# Synthesis and Cytoprotective Characterization of 8-Hydroxyquinoline Betti Products

**DOI:** 10.3390/molecules23081934

**Published:** 2018-08-02

**Authors:** Iván Kanizsai, Ramóna Madácsi, László Hackler Jr., Márió Gyuris, Gábor J. Szebeni, Orsolya Huzián, László G. Puskás

**Affiliations:** 1 Avidin Ltd., Alsó kikötő sor. 11D, H-6726 Szeged, Hungary; r.madacsi@avicorbiotech.com (R.M.); hackler@avidinbiotech.com (L.H.J.); gyuris.mario@gmail.com (M.G.); g.szebeni@avidinbiotech.com (G.J.S.); 2 Avicor Ltd., Alsó kikötő sor. 11D, H-6726 Szeged, Hungary; o.huzian@avicorbiotech.com

**Keywords:** 8-hydroxyquinoline, 8-HQ, Mannich-reaction, Betti-reaction, multicomponent reaction, cytoprotection, phenotypic screening, neurodegeneration, multitarget directed ligand, mitochondrial membrane potential, HIF1A

## Abstract

The 8-hydroxyquinoline pharmacophore scaffold has been shown to possess a range of activities as metal chelation, enzyme inhibition, cytotoxicity, and cytoprotection. Based on our previous findings we set out to optimize the scaffold for cytoprotective activity for its potential application in central nervous system related diseases. A 48-membered Betti-library was constructed by the utilization of formic acid mediated industrial-compatible coupling with sets of aromatic primary amines such as anilines, oxazoles, pyridines, and pyrimidines, with (hetero)aromatic aldehydes and 8-hydroxiquinoline derivatives. After column chromatography and re-crystallization, the corresponding analogues were obtained in yields of 13–90%. The synthesized analogs were optimized with the utilization of a cytoprotection assay with chemically induced oxidative stress, and the most active compounds were further tested in orthogonal assays, a real time cell viability method, a fluorescence-activated cell sorting (FACS)-based assay measuring mitochondrial membrane potential changes, and gene expression analysis. The best candidates showed potent, nanomolar activity in all test systems and support the need for future studies in animal models of central nervous system (CNS) disorders.

## 1. Introduction

In the last two decades, the 8-hydroxyquinoline (8-HQ) structure have emerged as a promising pharmacophore scaffold for medicinal chemists, due to a coded biological and synthetic potential owing to the active sites in the molecule on C-2, C-5, and C-7 carbons [1]. The presence of hydroxyl substituent on the C-8 carbon creates an *ortho* (C-7) and/or *para* (C-5) direction, providing active positions for electrophilic aromatic substitutions on C-5 (*para*) and for the accomplishment of aza Fiedel-Crafts-type reactions, including Mannich- or Betti-three components reactions (Mannich-3CR and Betti-3CR) predominantly on the C-7 (*ortho*) position. Higher molecular diversity and the formation of a new chiral carbon centre could be achieved by the use of Betti-3CR: treatment of several (aromatic) aldehydes with primary amines then 8-HQ, 5-nitro-, or 5-chloro-8-HQ. The application of Mannich-3CR of simple formaldehyde and secondary amines as an iminium source for addition, is highlighted for biological utilization [2,3]. Based on previously published biological studies, the structurally modified 8-HQs exert two main activities depending on the C-7 function and substitution pattern of the mother compound, 8-HQ. These compounds are either cytotoxic or active in cytoprotection assays. These studies describe a variety of 7-aminomethylated 8-HQ, 5-chloro-8-HQ and 5-nitro-8-HQ (nitroxoline) as products of different multicomponent reaction (MCR) techniques in different hit-to-lead optimizations.

### 1.1. Cytotoxicity

**Figure 1:**
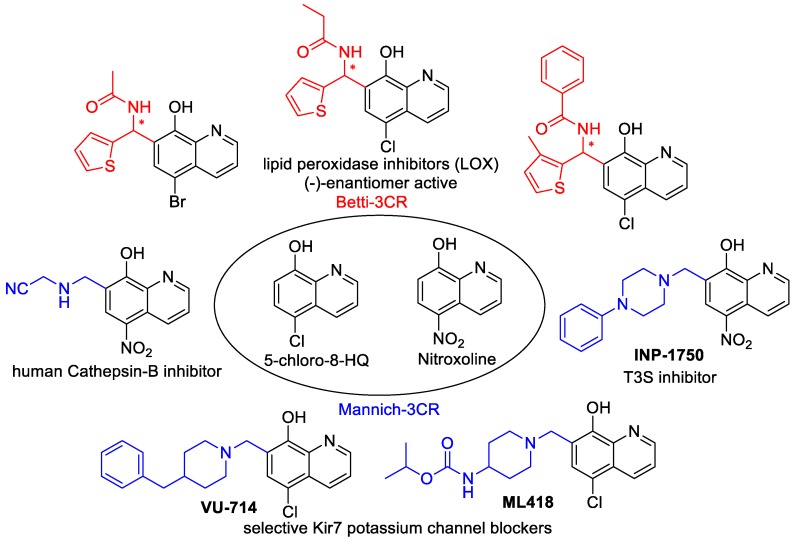
Cytotoxic 8-hydroxyquinoline (8-HQ) analogues.

The most promising cytotoxic 8-HQ analogues were depicted in Figure 1. They act through different mechanisms, namely the inhibition of Cathepsin B, KDM4 histone demethylases, 2-oxyglutarate oxygenase subtypes, and lipoxygenase (LOX)-enzymes [4,5,6,7,8,9,10]. Among the enzyme inhibitors, eutomer (−)-Betti products were presented with excellent, selective 12-LOX inhibition; moreover, these products were tested successfully in in vivo experiments. Additionally, INP1750 has a Gram-negative pathogen selective antibacterial effect [11]. Interestingly, if an *N*-sulfonated piperazine-1-yl unit was introduced in the C-7 position, the Mannich derivatives exerted an improved growth-inhibitory effect that was 26-fold more potent than that of 5-chloro-8-HQ against HeLa cells [12,13].

It is notable that the inhibitor activities were explained by an improved metal chelating ability compared with the parent compound 8-HQ, due to optimal copper and zinc(II)-binding in vivo [1,6,11,12,14].

### 1.2. Cytoprotection

In contrast with mentioned enzyme inhibition and antitumor activities, a number of 8-HQ derivatives possessed cytoprotective activities. 

As a suitable example, clioquinol (CQ, 5-Chloro-7-iodoquinolin-8-ol) has a selective, but low binding ability for Zn^2+^/Cu^2+^-ions, and it exerts potency against several neurodegenerative disorders (Huntington, Alzheimer’s, and Parkinson diseases) but its toxicity in long-term administration restricts its use in the clinic [15,16,17,18]. Structural modifications on the parent compound CQ have afforded second generation Zn^2+^/Cu^2+^ ionophores such as the PBT family (C-2 substituted 5,7-dichloro-8-HQ e.g. PBT2 (5,7-Dichloro-2-[(dimethylamino)methyl]quinolin-8-ol)) or their related structures, which have increased blood brain barrier permeability, more solubility, and less toxicity. In addition, PBT2 progressed to Phase IIa clinical trials as an anti-Alzheimer agent [19,20,21,22]. The multifunctional characteristics of these 8-HQ analogs provide the basis for the emerging use of multitarget-directed ligand (MTDL) treatment of neurogenerative diseases possessing a multifactorial nature e.g., Alzheimer’s disease.

**Figure 2:**
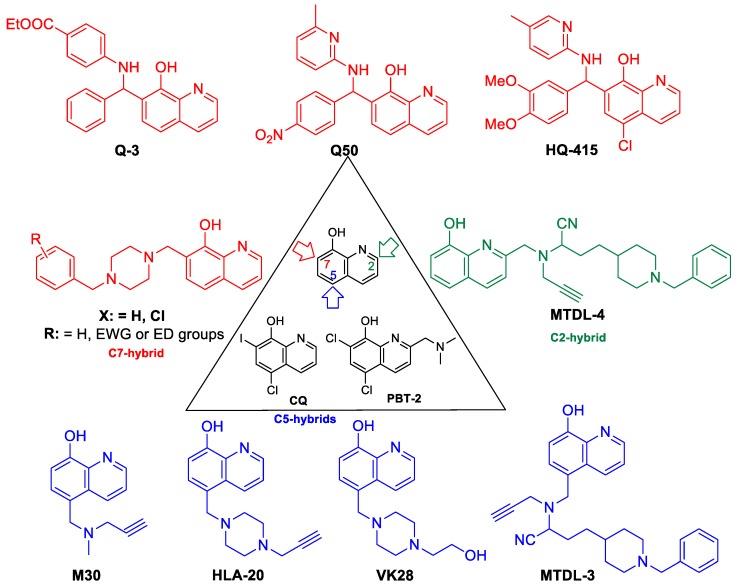
Cytoprotective 8-HQ derivatives.

An interesting medicinal chemistry concept is based on the formation of 8-HQ-hybrids (Figure 2). Known pharmacophores e.g. propargil or *N*-benzyl piperidine/piperazine moieties were substituted in C-2, C-5, or C-7 positions of 8-HQ creating analogues with an improved cytoprotective action. In fact, a series of 2- and 5-functionalized-8-hydroxyquinoline hybrid structures, HLA-20, M30, VK28, and MTDLs act as potent anti-neurodegenerative agents, exert significant inhibition of β-amyloid aggregation in vitro, decrease metal-driven oxidative damage and β-amyloid-mediated neurotoxicity. In addition, considerable cholinesterase inhibition, ROS scavenging, and an anti-aggregating effect on Aβ42 has been assessed [23,24,25,26,27,28,29,30,31,32,33,34,35,36]. These compounds incorporate known pharmacophore synthones from rasagiline (HLA20), donepezil (MTDL3 and MTDL4), or from rivastigmine (VK28). As a result of original framework combinations, a subclass of C-7-hybrides were also prepared by fusing *N*-benzyl piperazines/piperidines to 8-HQ, utilizing optimized Mannich-3CR conditions [37].

Interestingly, C-2 hydrazones, thiosemicarbazones, and semicarbazones modulate the Cu-Aβ peptide interactions as well. 8-HQ-2-hydrazones (INHHQ) were proved to be well-tolerated, stable derivatives which are able to cross the blood-brain barrier (BBB) and act as anti-Parkinson agents [38,39].

Phenotypic screening for functionally distinct 8-HQ derivatives on a yeast TAR DNA-binding protein 43 (TDP-43) toxicity model indicated a considerable reverse action for the expression of the TDP-43 protein in the case of compound HQ415 (Figure 2) [40]. Moreover, CQ can modulate amyloidogenic proteins/peptides and consequently it can be effective in Alzheimer’s disease, but its structural modifications towards Betti-bases (e.g., HQ415) resulted in significantly higher potency in neuronal proteotoxicity models, presumably due to having an effect on the process of oxidative stress and misregulation of iron metabolism besides enzyme inhibition [41].

Novel cytoprotective screening assays revealed Betti-bases as being a new subclass of potent cytoprotective agents; nevertheless, a few number of analogues had been analyzed up to now (Figure 2). During the utilization of a cell-microelectronic sensing technique (RT-CES), a small-membered Q-library, composed of seven commercially available 8-HQ analogues, was tested for cytoprotective activity in real-time to identify possible hit compound(s) against cardiac diseases. Notably, compound Q3 implicated 8-HQ, benzocain, and benzaldehyde motifs, emerging as a suitable candidate for a hit-to-lead optimization [42]. Systematic preparative efforts led to the analogue Q50, which was proven to be highly potent in improving cardiac functional recovery of ischemic/reperfused myocardium in rats [43].

The current work describes the optimization of the 8-HQ Betti products for cytoprotective activity in glioblastoma cells as a model for neuroprotection, as well as for possible application in CNS-related disorders.

## 2. Results and Discussion

**Scheme 1:**
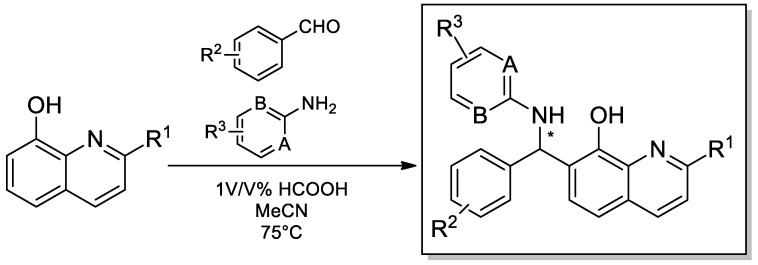
General protocol for the synthesis of compounds **1**–**48**.

Scheme 1 depicts the general synthetic procedures for the synthesis of products **1**–**48**. For a comprehensive biological evaluation, several primary aromatic amines, aromatic aldehydes and 8-hydroxyquinolines were subjected to the Betti-3CR. Notably, the achievement of a suitable optimal condition and access to a large number of Betti-compound libraries is a great challenge, owing to the fundamental Betti-conditions that proceed with very poor yields, and its improvement is strongly dependent on the applied amine and/or aldehyde components.

For a rapid synthetic method, a known industrial application was chosen to access a diverse chemical library. For the assemblies, 1 *v*/*v* % formic acid was exploited as Brönsted-acid mediator in acetonitrile at 75 °C. We focused on the preparation of Betti compounds with high purities regardless of the yields and optimal conditions.

At first, an optionally selected 15-membered basic library was prepared and assessed in a cytoprotection assay to reveal the possible next step for a synthetic strategy. Following a medicinal chemistry protocol, the 8-HQ was conducted by means of simple amine inputs such as aniline, 2-aminopyridine, 2-aminopyrimidine, and 3-aminoisoxazol in combination with unsubstituted benzaldehyde and 2-pyridinecarbaldehyde as a *N*-containing substrate. In addition, several structural modifications were accomplished to prepare some substituted variants by altering either the amine or aldehyde components. In accordance with that, 10 other examples were synthesized using substituted benzaldehydes, containing electron withdrawing (CF_3_, F, and NO_2_) or electron donating (*O*-alkyl) group in the *para* position, in combination with e.g., benzocain, *p*-benzoic acid (PABA), 5-alkyl (methyl or *tert*-butyl)-substituted isoxazoles, or 6-picoline.

For pyrimidine based analogues, only two derivatives were presented, consisting of unsubstituted pyrimidine units with phenyl (from benzaldehyde) or 4-nitrophenyl (from *p*-nitrobenzaldehyde) moieties. 

**Scheme 2:**
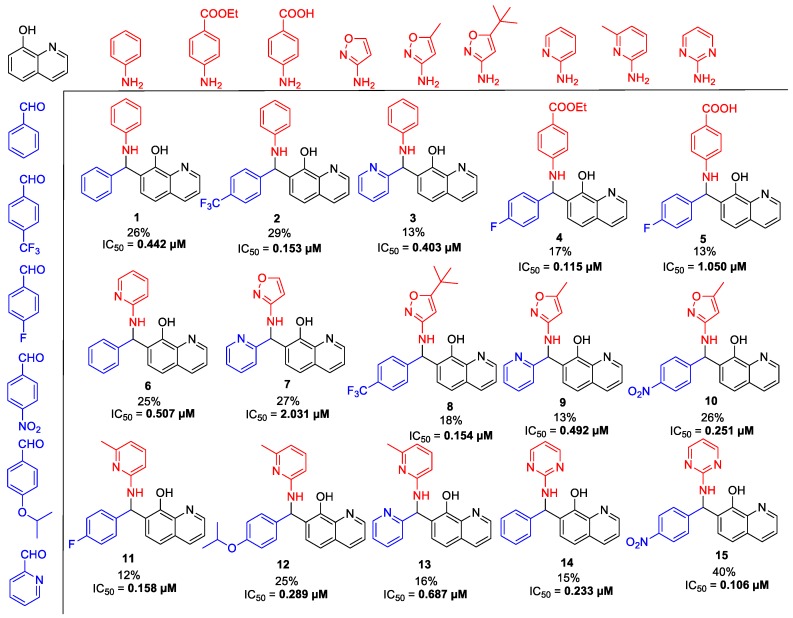
The tested basic chemical library with half maximal inhibitory concentration (IC_50_) values in a cytoprotection assay. Each analog was prepared with the combination of the shown aldehydes, substituted amines, and 8-HQ.

In all cases, the formic acid-mediated Betti-transformations were accomplished to furnish the pure desired products **1**–**15** in yields of 12–40% (Scheme 2).

### 2.1. Primary Assay

We have utilized a simple and inexpensive method to screen our 8-HQ analogs for cytoprotective activity. U251 MG glioblastoma cells were exposed to hydrogen peroxide to induce oxidative stress, and the effects of co-treatment with the synthesized compounds were investigated after a 24 h incubation by a fluorimetric endpoint assay. Cytoprotective compounds could be identified by resulting in more live cells after a peroxide challenge.

### 2.2. Structure Activity Relationship

Gratifyingly, the first assessment delineated a preliminary structure-activity relation (SAR), and demonstrated a high cytoprotective potential, due to the Betti-structure. In fact, we observed sub-micromolar activities in most cases, except for compounds **5** and **7** (1.05 µM and 2.03 µM). Consequently, application of PABA and non-substituted isoxazoles as an amine source is not suggested for further application. Despite that, presence of an alkyl function on C-5 position for the isoxazole ring significantly increases the efficiencies, as shown in case of compounds **8**–**10** (IC_50 (**8**–**10**)_ = 0.15, 0.49, and 0.25 µM). Interestingly, substitutions of the amine or aldehyde are capable of enhancing the cytoprotective activity as demonstrated by the changing IC_50_ values in the range from 2.03 µM towards 0.11 µM. Based on these preliminary results, we assumed that it was possible to increase the scaffold’s potential; however, the perfect substitution pattern could not be drawn due to similar sub-micromolar activities for differently oriented substitution patterns in starting substrates. 

Accordingly, further synthetic efforts could not be limited to one mainstream biological optimization process, with a focus on the variation of either the amine or the aldehyde reagents; rather, our strategy is based on the construction of a chemical library involving three subclasses (substituted anilines (Subclass 1), 2-aminopicolines (Subclass 2) and 2-aminopyrimidines (Subclass 3)) to demonstrate structure-activity relations and to give a chance to select lead-like compounds for the final assessment.

Coupling of benzocaine (presence of *para*-COOEt group) instead of aniline with 4-CF_3_–benzaldehyde and 8-HQ slightly attenuated the biological activity of derivative **16** from 0.15 µM (for **2**) to 0.19 µM (Table 1). On the other hand, slightly better results were obtained by the substitution of R″ = 4-F–C_6_H_4_ (analogues **4**; IC_50_ = 0.11 µM) with a 2-pyridyl group (analogues **17**; IC_50_ = 0.12 µM). Interestingly, the application of benzocaine for the construction of a 2-pyridyl-containing analogue **17** improved the cytoprotection potential significantly (compound **3** with IC_50_ = 0.40 µM in comparison with **17**, IC_50_ = 0.12 µM). Moreover, replacement of the carbetoxy group with a NO_2_ or CF_3_ moiety resulted in synthesized compounds with excellent efficiency in cytoprotection, with 0.09 µM (R′ = 4-CF_3_–C_6_H_4_ and R″ = 2-pyridyl; compound **18**) and 0.12 µM (R′ = 4-NO_2_–C_6_H_4_ and R″ = 2-pyridyl; compound **19**) IC_50_ values. 

From Subclass 1, three superior compounds **17** and **18** besides analogue **4** were selected as lead-like compounds.

For the picoline family, basic compounds **6**, **11**, **12**, and **13** highlighted the suitable reagent combination, such as *para*-substituted aldehydes, regardless of the electronic nature of the substituents and/or 6-picoline. Moreover, incorporation of the R″ = 2-pyridyl unit in compound **13** (IC_50_ = 0.687 µM) resulted in a weak cytoprotective potential in comparison with benzaldehyde-based analogues **11** (R″ = 4-F–C_6_H_4_; IC_50_ = 0.158 µM) and **12** (R″ = 4OCH(CH_3_)_2_–C_6_H_4_; IC_50_ = 0.289 µM). Although, both of electron withdrawing group (EWG)- or electron donating group (EDG)-substituted analogues possess higher activity than the unsubstituted such as compound **6** or the 2-pyridylform **13**, **11** with *para*-fluoro moiety on the R″ position demonstrated better efficiency.

In a pyridine-based optimization a tendency could be outlined by introducing a *para*–trifluoromethyl or *para*–nitrophenyl group on the R″ function, causing a remarkable positive effect. For a first improvement, the tested analogues **20** and **21** exerted excellent activity with IC_50_ = 0.087 µM and 0.086 µM values. Notably, their representative C-2 methylated variant **23** had diminished cytoprotection levels, thus the 8-hydroxyquinaldine proved to be an unsuitable component for the development process. Afterwards, we considered the in-vivo or in-use feasibilities of nitro and trifluoromethylated derivatives in future studies. For possible toxicity issues therefore, we decided that the trifluoromethylated structure **20** would be the favored scaffold for further modification, and that the development of nitro derivatives was terminated. In the next steps, the positions of the trifluoromethyl and the picolines methyl group were varied, resulting in a slightly decreased IC_50_ range: 0.156–0.166 µM were observed for cases **25**–**27**. Interestingly, application of di-substituted aldehydes involving trifluoromethyl and/or fluoro substituents in 2,4-; 3,4-, and 3,5-positions resulted in molecules **28**–**32**, which displayed a decreased biological activity with an IC_50_ = 0.204 µM at best (Table 2). Based on these observations, **20** and **21** picoline derivatives were selected for the final study.

Although the preliminary test defined high potential derivatives **14** and **15** with the introduction of a non-substituted pyrimidine moiety (IC_50_ values 0.233 µM and 0.106 µM), additional transformations should be accomplished that focus on the impact of replacing of CF_3_ with either EWG groups or ED functions. Also, the influence on the activity of further substituents on the phenyl ring was tested. In addition, further efforts were taken to determine the importance of introducing a methyl moiety on the pyrimidine heterocycle. Exploiting the earlier presented method, 16 analogues were prepared, with yields of up to 68% (Table 3). Except for **33** (besides the compound **14** and **15**), all Betti-products were derived from the 4-methyl-2-amino-1,3-pyrimidine as amine input. The firstly-prepared **33**, with a 4-CF_3_–C_6_H_4_ function on the R″ position, demonstrated a slightly better effect in comparison with R″ = C_6_H_5_ (**14**) or 4-NO_2_–C_6_H_4_ (**15**). Gratifyingly, the introduction of a methyl on the pyrimidine ring also increased the cytoprotection activity. For R″ = 4-NO_2_–C_6_H_4_ (**35**), a similar IC_50_ value (0.114 µM) was observed. Replacing CF_3_ with SF_5_ (**36**) or halogenides such as fluorine (**37**), bromine (**38**), or iodine (**39**) slightly attenuated the efficiency. Interestingly, the combination of 4-chlorobenzaldehydes, 4-methyl-2-amino-pyrimidine, and 8-HQ afforded one of the most potent derivatives, **40**, with a 73-nM IC_50_ value. Despite that, the 2,4- or 3,5-disubstituted phenyl moieties reduced the bioactivities; however, an acceptable cytoprotection level was provided by the incorporation of the 2,4-CF_3_–C_6_H_3_ group (compound **43**, IC_50_ = 0.119 µM). Modification on the pyrimidine structure, replacing methyl into a bromine unit decreased the activity to 0.183 µM, while a solubility issue also occurred in that case. The presence of an electron-donating group on the R″ position (analogue **47,** IC_50_ = 0.481 µM) or a C-2 methyl function (derivative **48**, IC_50_ = 0.887 µM) diminished the cytoprotective potential.

According to our results, compounds **34**, **35**, **40**, and **43** were proved to be the most potent pyrimidine derivatives and were selected for final assessment.

### 2.3. Evaluation of the Most Active Compounds in a Real-Time Cytoprotection Assay

In our primary assay, we have identified compounds that showed excellent activity. However, the derivatization of the basic scaffold with the combination of beneficial substitutions resulted in a handful of products with very similar activity and at the same time, significantly different modifications. Since the primary assay was unable to pinpoint one or two products that could be marked as leads, in an attempt to narrow down our selection of the most active compounds (compounds **4**, **17**, **18**, **20**, **21**, **34**, **35**, **40**, and **43** with IC_50_ between 0.08 and 0.12 µM) we have utilized an orthogonal assay to determine cytoprotective activity. Cell-based phenotypic screening, including end-point in vitro cellular screening protocols to identify cytoprotective compounds, are commonly used [44]; however, real-time cellular assays provide more information on the kinetics of drug action. Previously we have successfully adopted the RT-CES system to identify cytoprotective compounds [37]. Briefly, cells grown on golden electrodes in microplates were incubated with hydrogen peroxide alone or in the presence of our compounds. Using this system, cell viability can be monitored continuously with one-minute resolution through the measurement of impedance of the electrodes. The detected impedance is converted to an arbitrary measure (cell index) that is proportional to the number of attached cells, the strength of their attachment and cell morphology, since these properties influence electrode coverage.

**Figure 3:**
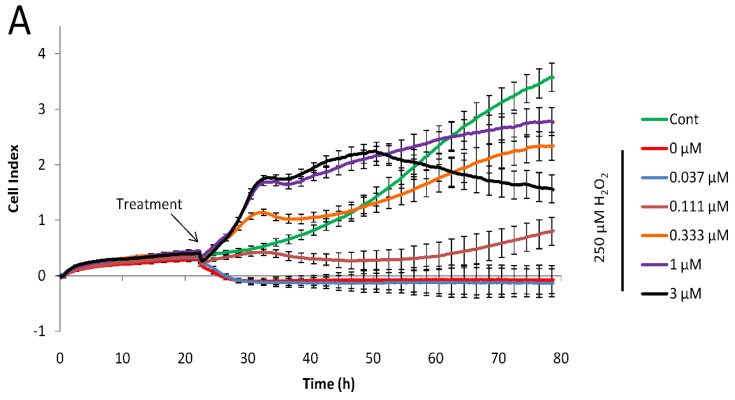

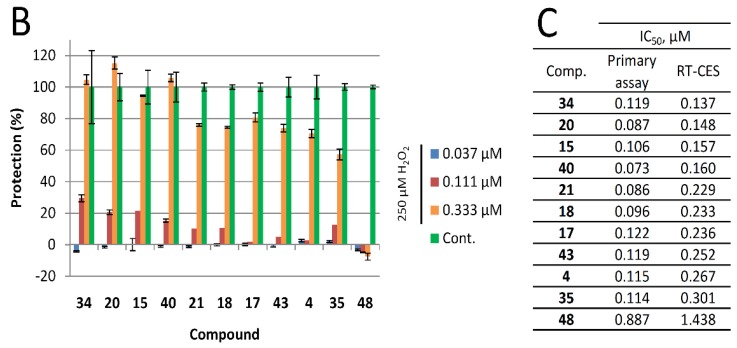
Real-time cytoprotection assay. (**A**): Real-time viability data traces of the most active **34** analog were followed for 60 h after treatment. (**B**): Cytoprotective activity of the tested compounds 24 h after treatment. Percentages were calculated in relation to non-treated and hydrogen peroxide-only treated cell data. (**C**): Comparison of IC_50_ values determined from the primary (resazurin, end-point) assay and the real time assay 24 h after treatment.

Using this real-time technique we have determined the IC_50_ values for each selected compound 24 h after treatment. The calculated values were comparable to the ones determined in the primary assay, hence this assay was also unable to pinpoint a lead. Interestingly, the 4-CF_3_ and 4-methyl pyrimidine sidechain containing **34** performed the best with the lowest IC_50_ value, and significant effect (29% protection) at 110 nM concentration. Our least active compound, **48**, bearing a methyl group in position 2 on the 8-HQ structure, also performed poorly in this assay. Results are summarized in Figure 3.

### 2.4. Mitochondrial Membrane Depolarization following Oxidative Stress is Reversed by Treatment with the Novel 8-HQ Analogs

Mitochondrial dysfunction is a prominent feature in neurodegenerative diseases, it can result in production and amplification of reactive oxygen species, and may be important in the pathophysiology of these diseases. Changes in the mitochondrial membrane potential (MMP) are detected in oxidative stress, and were suggested even as a biomarker for oxidative environmental stress [45].

**Figure 4:**
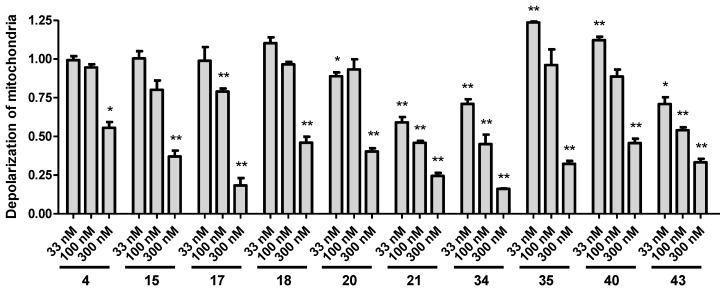
Mitochondrial membrane depolarization assay. Treatment with the synthesized analogs reversed the mitochondrial membrane potential changes caused by oxidative stress. All data was calculated by relation to hydrogen peroxide only treatment set to 1. Statistical significance (*t*-test): * *p* < 0.05 and ** *p* < 0.01.

The selected 10 most active analogues (compounds **4**, **15**, **17**, **18**, **20**, **21**, **34**, **35**, **40**, and **43**) were also tested as to whether they were able to reverse the mitochondrial membrane depolarization caused by the induced oxidative stress by hydrogen peroxide. The applied hydrogen peroxide treatment was higher in these experiments than in the previous assays (500 µM vs. 250 µM), in order to see a significant effect in the 2 h timeframe of this assay. All tested analogs effectively reversed the detected membrane depolarization. In this assay, the most active compounds (**21**, **34**, and **43**) were active even at the lowest applied 33 nM concentration. Figure 4 shows the effect of treatments compared to hydrogen peroxide treatment only (set by normalization as 1).

### 2.5. 8-HQ Analogues Induce Hypoxia Related Genes and Glucose Transporter Expression

The contribution of cerebrovascular deficiencies (such as cerebral ischemia/stroke) and the dysregulation of brain insulin signaling have been strongly implicated in neurodegenerative diseases in recent years [46]. Reduction of blood supply leading to a hypoxic condition is known to activate cellular responses through hypoxia-inducible factors (HIFs). The stabilization of HIF1A protein controlling the expression of stress adaptation related genes is a major factor in the oxidative stress response [47]. Since our original 8-HQ analog (Q50, **21**) showed potent activity in ischemic/reperfused myocardium in rats, it was selected along with a highly active (**34**) and less active compounds (**47**, **48**), and was investigated for its effect on oxidative stress response genes. Quantitative real-time PCR (qRT-PCR) analysis of the expression of oxidative stress-related genes HMOX-1, VEGF, and a glucose transporter GLUT1 was carried out.

**Figure 5:**
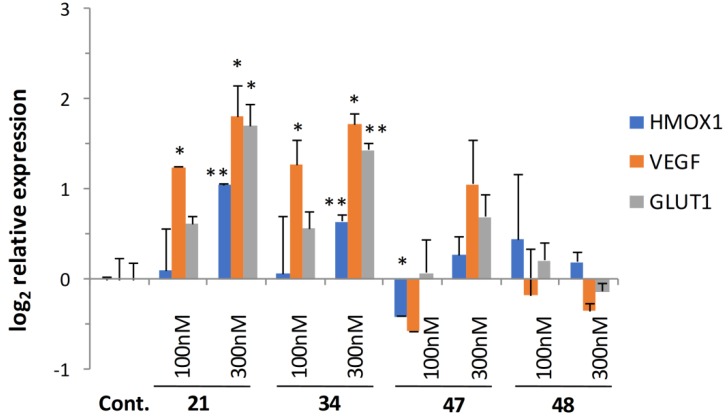
Induction of hypoxia-related gene and glucose transporter expression. HIF1A-regulated genes were activated following treatment with selected analogs in a dose- and cytoprotective activity-dependent manner. Statistical significance (*t*-test): * *p* < 0.05 and ** *p* < 0.01.

Results depicted in Figure 5 showed significant gene activation following treatment at 100 and 300 nM concentrations in the case of **21** and **34**, while gene-inducing capacity decreased parallel to cytoprotective activity with the less active compounds.

## 3. Materials and Methods

All NMR spectra were recorded in deuterated dimethyl sulfoxide (DMSO) at 298 K on a Bruker Avance 500 (Billerica, MA, USA) or Bruker Avance Neo 500 spectrometer (Billerica, MA, USA). All chemical shifts (δ) were reported in ppm relative to the residual solvent signal. HRMS spectra were recorded on a Thermo Scientific Q Exactive Plus mass spectrometer (Waltham, MA, USA) using a heated electrospray ionization (HESI-II) probe ion source. TLC was performed on aluminum sheets coated with silica gel 60 F254 (Merck, 1.05554, Budapest, Hungary). Visualization was done under UV light (254 nm). Column chromatography was carried out using silica gel (Merck, 60 Å, 0.063‒0.200 mm, Budapest, Hungary). Melting points were determined by a Stuart SMP10 device (Staffordshire, UK), and they were uncorrected. All chemicals and solvents were of commercial grade and were used without further purification. 

### 3.1. General Procedure for the Syntheses of Compounds ***1**–**48***

To a solution of 1 mmol aldehyde, 3× volume of acetonitrile and 1 equivalent volume of amine were added to 0.6 equivalent of quinoline derivatives and 1% (*v*/*v*) formic acid. The reaction mixture was stirred at higher temperature (75 °C). The reaction was monitored by TLC (eluent: hexane isomeric mixture:acetone). If the product precipitated, it was filtered, washed with hexane, and dried. If the reaction mixture was homogeneous, it was evaporated to dryness and was purified by column chromatography (eluent: hexane:acetone from 20:1 to 4:1, *v*/*v*). The crude product was crystallized from hexane/ethyl-acetate. The molecular structures were determined by means of 1D and 2D-NMR technologies (see Supplementary Materials)

*7-(Phenyl(phenylamino)methyl)quinolin-8-ol* (**1**): white solid, yield: 26% (51 mg), melting point (m.p.): 141–143 °C, C_22_H_18_N_2_O; ^1^H-NMR (500 MHz, DMSO) δ 9.99 (s, 1H), 8.82 (s, 1H), 8.24 (d, *J* = 7.0 Hz, 1H), 7.59 (d, *J* = 7.8 Hz, 1H), 7.53–7.48 (m, 1H), 7.43–7.38 (m, 2H), 7.35 (*d*, *J* = 7.9 Hz, 1H), 7.32–7.23 (m, 2H), 7.24–7.14 (m, 1H), 7.01–6.92 (m, 2H), 6.67–6.59 (m, 2H), 6.49–6.42 (m, 2H), 6.14 (d, *J* = 5.3 Hz, 1H); ^13^C-NMR (126 MHz, CDCl_3_) δ 149.77 (s), 148.25 (s), 147.97 (s), 142.87 (s), 138.11 (s), 136.04 (s), 128.75 (s), 128.34 (s), 127.51 (s), 127.36 (s), 126.82 (s), 126.29 (s), 125.45 (s), 121.69 (s), 117.54 (s), 118.06 (s), 112.94 (s), 54.16 (s); HRMS (ESI) *m*/*z* calcd. for C_22_H_18_N_2_O [M + H]^+^: 327.1492, found: 327.1493.

*7-((Phenylamino)(4-(trifluoromethyl)phenyl)methyl)quinolin-8-ol* (**2**): white solid, yield: 29% (69 mg), m.p.: 83–85 °C, C_23_H_17_F_3_N_2_O; ^1^H-NMR (500 MHz, DMSO) δ 10.14 (s, 1H), 8.84 (d, *J* = 2.2 Hz, 1H), 8.26 (d, *J* = 8.2 Hz, 1H), 7.67 (d, *J* = 7.9 Hz, 2H), 7.62 (d, *J* = 7.8 Hz, 2H), 7.56 (d, *J* = 8.5 Hz, 1H), 7.52 (dd, *J* = 7.7, 3.7 Hz, 1H), 7.38 (d, *J* = 8.4 Hz, 1H), 7.00 (t, *J* = 7.4 Hz, 2 H), 6.64 (d, *J* = 7.8 Hz, 2H), 6.55 (d, *J* = 6.9 Hz, 1H), 6.50 (t, *J* = 7.0 Hz, 1H), 6.23 (d, *J* = 6.9 Hz, 1H); ^13^C-NMR (126 MHz, CDCl_3_) δ 149.95 (s), 148.38 (s), 147.73 (s), 147.68 (s), 138.14 (s), 136.09 (s), 128.84 (s), 128.09 (s), 127.71 (s), 127.53 (q, *J* = 31.9 Hz), 126.16 (s), 125.29 (q, *J* = 3.3 Hz), 124.58 (s), 121.88 (s), 117.76 (s), 116.48 (s), 113.04 (s), 54.03 (s); HRMS (ESI) *m*/*z* calcd. for C_23_H_17_F_3_N_2_O [M + H]^+^: 395.1366, found: 395.1369.

*7-((Phenylamino)(pyridin-2-yl)methyl)quinolin-8-ol* (**3**): white solid, yield: 13% (26 mg), m.p.: 129–130 °C, C_21_H_17_N_3_O; ^1^H-NMR (500 MHz, DMSO) δ 10.16 (s, 1H), 8.86 (dd, *J* = 4.2, 1.5 Hz, 1H), 8.56 (dd, *J* = 4.8, 0.7 Hz, 1H), 8.25 (dd, *J* = 8.3, 1.5 Hz, 1H), 7.76 (td, *J* = 7.7, 1.8 Hz, 1H), 7.59–7.47 (m, 3H), 7.33 (d, *J* = 8.6 Hz, 1H), 7.27 (ddd, *J* = 7.4, 5.0, 0.8 Hz, 1H), 7.02 (dd, *J* = 8.1, 7.6 Hz, 2H), 6.70 (d, *J* = 7.8 Hz, 2H), 6.61 (d, *J* = 7.1 Hz, 1H), 6.50 (t, *J* = 7.3 Hz, 1H), 6.27 (d, *J* = 7.0 Hz, 1H); ^13^C-NMR (126 MHz, DMSO) δ 160.99 (s), 150.53 (s), 149.30 (s), 148.69 (s), 147.80 (s), 138.59 (s), 137.43 (s), 136.48 (s), 129.28 (s), 128.05 (s), 126.81 (s), 125.23 (s), 122.81 (s), 122.50 (s), 122.21 (s), 117.96 (s), 116.68 (s), 113.31 (s), 55.40 (s); HRMS (ESI) *m*/*z* calcd. for C_21_H_17_N_3_O [M + H]^+^: 328.1444, found: 328.1445.

*Ethyl 4-((4-fluorophenyl)(8-hydroxyquinolin-7-yl)methylamino)benzoate* (**4**): white solid, yield: 17% (42 mg), m.p.: 142–144 °C, C_25_H_21_FN_2_O_3_; ^1^H-NMR (500 MHz, DMSO) δ 10.17 (s, 1H), 8.87 (dd, *J* = 4.2, 1.5 Hz, 1H), 8.30 (dd, *J* = 8.3, 1.5 Hz, 1H), 7.64 (d, *J* = 8.9 Hz, 2H), 7.56 (dd, *J* = 8.3, 4.2 Hz, 1H), 7.51 (d, *J* = 8.6 Hz, 1H), 7.45–7.38 (m, 3H), 7.34 (d, *J* = 7.2 Hz, 1H), 7.20–7.14 (m, 2H), 6.69 (d, *J* = 8.8 Hz, 2H), 6.25 (d, *J* = 7.2 Hz, 1H), 4.18 (q, *J* = 7.0 Hz, 2H), 1.24 (t, *J* = 7.1 Hz, 3H).; ^13^C-NMR (126 MHz, CDCl_3_) δ 165.78 (s), 161.24 (d, *J* = 243.6 Hz), 151.80 (s), 150.01 (s), 148.42 (s), 138.11 (s), 136.11 (s), 130.78 (s), 129.38 (d, *J* = 8.1 Hz), 127.72 (s), 125.97 (s), 124.27 (s), 121.90 (s), 117.72 (s), 116.96 (s), 115.26 (s), 115.09 (s), 111.95 (s), 59.58 (s), 53.35 (s), 14.34 (s).

*4-((4-Fluorophenyl)(8-hydroxyquinolin-7-yl)methylamino)benzoic acid* (**5**): white solid, yield: 13% (30 mg), m.p.: 185–187 °C, C_23_H_17_FN_2_O_3_; ^1^H-NMR (500 MHz, DMSO) δ 12.01 (s, 1H), 10.22 (s, 1H), 8.85 (d, *J* = 2.2 Hz, 1H), 8.28 (d, *J* = 8.1 Hz, 1H), 7.69 (d, *J* = 7.9 Hz, 2H), 7.61 (d, *J* = 8.9 Hz, 2H), 7.59 (d, *J* = 8.4 Hz, 2H), 7.54 (dd, *J* = 8.0, 3.9 Hz, 1H), 7.48 (d, *J* = 8.5 Hz, 1H), 7.39 (d, *J* = 8.5 Hz, 1H), 7.31 (d, *J* = 6.9 Hz, 1H), 6.66 (d, *J* = 8.3 Hz, 2H), 6.31 (d, *J* = 6.7 Hz, 1H); ^13^C-NMR (126 MHz, CDCl_3_) δ 167.37 (s), 151.48 (s), 150.16 (s), 149.13 (d, *J* = 297.5 Hz), 148.49 (s), 146.83 (s), 138.14 (s), 136.14 (s), 131.02 (s), 128.20 (s), 127.85 (s), 126.06 (s), 125.41 (d, *J* = 2.8 Hz), 123.73 (s), 122.01 (s), 118.04 (s), 117.84 (s), 111.92 (s), 53.82 (s).

*7-(Phenyl(pyridin-2-ylamino)methyl)quinolin-8-ol* (**6**): white solid, yield: 25% (49 mg), m.p.: 172–174 °C, C_21_H_17_N_3_O; ^1^H-NMR (500 MHz, DMSO) δ 9.92 (s, 1H), 8.85 (dd, *J* = 4.0, 1.3 Hz, 1H), 8.29 (dd, *J* = 8.3, 1.1 Hz, 1H), 7.92 (d, *J* = 4.1 Hz, 1H), 7.62 (d, *J* = 8.5 Hz, 1H), 7.54 (dd, *J* = 8.3, 4.2 Hz, 1H), 7.42–7.34 (m, 5 H), 7.29 (t, *J* = 7.6 Hz, 2 H), 7.20 (t, *J* = 7.3 Hz, 1 H), 6.86 (d, *J* = 8.5 Hz, 1 H), 6.68 (d, *J* = 8.4 Hz, 1 H), 6.55–6.37 (m, 1H); ^13^C-NMR (126 MHz, DMSO) δ 158.43 (s), 150.00 (s), 148.69 (s), 147.90 (s), 144.00 (s), 138.57 (s), 137.12 (s), 136.47 (s), 128.62 (s), 127.86 (s), 127.66 (s), 127.04 (s), 126.95 (s), 126.36 (s), 122.05 (s), 117.73 (s), 112.51 (s), 109.26 (s), 52.08 (s); HRMS (ESI) *m*/*z* calcd. for C_21_H_17_N_3_O [M + H]^+^: 328.1444, found: 328.1447.

*7-(Isoxazol-3-ylamino)(pyridin-2-yl)methyl)quinolin-8-ol* (**7**): white solid, yield: 27% (52 mg), m.p.: 145–146 °C, C_18_H_14_N_4_O_2_; ^1^H-NMR (500 MHz, DMSO) δ 10.02 (s, 1H), 8.82 (d, *J* = 2.1 Hz, 1H), 8.49 (d, *J* = 3.4 Hz, 1H), 8.31 (bs, 1H), 8.24 (d, *J* = 8.1 Hz, 1H), 7.73 (t, *J* = 7.5 Hz, 1H), 7.54 (d, *J* = 8.5 Hz, 1H), 7.52–7.46 (m, 2H), 7.33 (d, *J* = 8.5 Hz, 1H), 7.27–7.12 (m, 2H), 6.35 (d, *J* = 7.9 Hz, 1H), 6.09 (bs, 1H); ^13^C-NMR (126 MHz, CDCl_3_) δ 162.79 (s), 160.56 (s), 158.17 (s), 149.88 (s), 148.84 (s), 148.23 (s), 138.14 (s), 136.83 (s), 136.00 (s), 127.57 (s), 126.24 (s), 124.61 (s), 122.27 (s), 121.81 (s), 121.73 (s), 117.23 (s), 97.02 (s), 55.73 (s); HRMS (ESI) *m*/*z* calcd. for C_18_H_14_N_4_O_2_ [M + H]^+^: 319.1190, found: 319.1189.

*7-((5-tert-Butylisoxazol-3-ylamino)(4-(trifluoromethyl)phenyl)methyl)quinolin-8-ol* (**8**): white solid, yield: 18% (48 mg), m.p.: 185–187 °C, C_24_H_22_F_3_N_3_O_2_; ^1^H-NMR (500 MHz, DMSO) δ 10.08 (s, 1H), 8.84 (bs, 1H), 8.28 (d, *J* = 8.2 Hz, 1H), 7.66 (d, *J* = 7.8 Hz, 2H), 7.59–7.50 (m, 4H), 7.40 (d, *J* = 8.4 Hz, 1H), 7.10 (d, *J* = 7.8 Hz, 1H), 6.31 (d, *J* = 7.7 Hz, 1H), 5.66 (s, 1H), 1.18 (s, 9H); ^13^C-NMR (126 MHz, DMSO) δ 179.26 (s), 163.57 (s), 150.17 (s), 148.89 (s), 148.27 (s), 138.57 (s), 136.54 (s), 128.11 (s), 127.84 (q, *J* = 31.9 Hz), 126.38 (s), 125.69 (q, *J* = 3.9 Hz), 124.93 (s), 124.78 (q, *J* = 272.2 Hz), 122.35 (s), 118.05 (s), 91.12 (s), 54.54 (s), 32.63 (s), 28.89 (s); HRMS (ESI) *m*/*z* calcd. for C_24_H_22_F_3_N_3_O_2_ [M + H]^+^: 442.1737, found: 442.1739.

*7-((4-Fluorophenyl)(5-methylisoxazol-3-ylamino)methyl)quinolin-8-ol* (**9**): white solid, yield: 13% (27 mg), m.p.: 153–155 °C, C_20_H_16_FN_3_O_2_; ^1^H-NMR (500 MHz, DMSO) δ 9.98 (s, 1H), 8.86 (dd, *J* = 3.9, 1.1 Hz, 1H), 8.30 (d, *J* = 8.3 Hz, 1H), 7.59 (d, *J* = 8.5 Hz, 1H), 7.55 (dd, *J* = 8.3, 4.2 Hz, 1H), 7.41 (d, *J* = 8.7 Hz, 1H), 7.38 (dd, *J* = 8.4, 5.9 Hz, 2H), 7.13 (t, *J* = 8.8 Hz, 2H), 7.01 (d, *J* = 8.3 Hz, 1H), 6.23 (d, *J* = 8.3 Hz, 1H), 5.73 (s, 1H), 2.20 (s, 3H); ^13^C-NMR (126 MHz, DMSO) δ 167.92 (s), 164.03 (s), 161.48 (d, *J* = 242.6 Hz), 149.95 (s), 148.76 (s), 139.56 (s), 138.55 (s), 136.49 (s), 129.32 (d, *J* = 8.1 Hz), 127.96 (s), 126.27 (s), 125.66 (s), 122.18 (s), 117.87 (s), 115.36 (d, *J* = 21.2 Hz), 94.33 (s), 54.21 (s), 12.45 (s); HRMS (ESI) *m*/*z* calcd. for C_20_H_16_FN_3_O_2_ [M + H]^+^: 350.1299, found: 350.1300.

*7-((5-Methylisoxazol-3-ylamino)(4-nitrophenyl)methyl)quinolin-8-ol* (**10**): white solid, yield: 26% (59 mg), o.p.: 138–140 °C, C_20_H_16_N_4_O_4_; ^1^H-NMR (500 MHz, DMSO) δ 10.16 (s, 1H), 8.84 (s, 1H), 8.28 (d, *J* = 8.1 Hz, 1H), 8.16 (d, *J* = 8.2 Hz, 2H), 7.61 (d, *J* = 8.1 Hz, 2H), 7.56–7.48 (m, 2H), 7.40 (d, *J* = 8.4 Hz, 1H), 7.15 (d, *J* = 7.7 Hz, 1H), 6.34 (d, *J* = 7.7 Hz, 1H), 5.74 (s, 1H), 2.18 (s, 3H); ^13^C-NMR (126 MHz, CDCl_3_) δ 167.76 (s), 163.53 (s), 150.90 (s), 149.77 (s), 148.49 (s), 146.35 (s), 138.15 (s), 136.10 (s), 128.01 (s), 127.77 (s), 125.93 (s), 124.02 (s), 123.56 (s), 122.00 (s), 117.73 (s), 93.91 (s), 54.18 (s), 12.01 (s); HRMS (ESI) *m*/*z* calcd. for C_20_H_16_N_4_O_4_ [M + H]^+^: 377.1244, found: 377.1250.

*7-((4-Fluorophenyl)(6-methylpyridin-2-ylamino)methyl)quinolin-8-ol* (**11**): white solid, yield: 12% (26 mg), m.p.: 157–159 °C, C_22_H_18_FN_3_O; ^1^H-NMR (500 MHz, DMSO) δ 9.97 (s, 1H), 8.82 (d, *J* = 2.5 Hz, 1H), 8.26 (d, *J* = 8.0 Hz, 1H), 7.63 (d, *J* = 8.5 Hz, 1H), 7.50 (dd, *J* = 8.1, 4.0 Hz, 1H), 7.41–7.31 (m, 3H), 7.30–7.17 (m, 2H), 7.08 (t, *J* = 8.7 Hz, 2H), 6.80 (d, *J* = 8.4 Hz, 1H), 6.40 (t, *J* = 12.7 Hz, 1H), 6.33 (d, *J* = 6.9 Hz, 1H), 2.19 (s, 3H); ^13^C-NMR (126 MHz, CDCl_3_) δ 160.92 (d, *J* = 242.0 Hz), 157.50 (s), 155.70 (s), 149.53 (s), 148.29 (s), 139.82 (s), 138.13 (s), 137.24 (s), 136.04 (s), 128.95 (d, *J* = 8.0 Hz), 127.48 (s), 126.60 (s), 125.68 (s), 121.68 (s), 117.43 (s), 114.84 (d, *J* = 21.2 Hz), 111.29 (s), 105.17 (s), 51.11 (s), 24.23 (s); HRMS (ESI) *m*/*z* calcd. for C_22_H_18_FN_3_O [M + H]^+^: 360.1507, found: 360.1508.

*7-((4-Isopropoxyphenyl)(6-methylpyridin-2-ylamino)methyl)quinolin-8-ol* (**12**): white solid, yield: 25% (60 mg), m.p.: 132–134 °C, C_25_H_25_N_3_O_2_; ^1^H-NMR (500 MHz, DMSO) δ 9.80 (d, *J* = 117.5 Hz, 1H), 8.85 (dd, *J* = 4.1, 1.4 Hz, 1H), 8.29 (dd, *J* = 8.3, 1.3 Hz, 1H), 7.67 (d, *J* = 8.5 Hz, 1H), 7.53 (dd, *J* = 8.3, 4.2 Hz, 1H), 7.40 (d, *J* = 8.6 Hz, 1H), 7.26 (t, *J* = 8.2 Hz, 3H), 7.16 (d, *J* = 8.9 Hz, 1H), 6.82 (d, *J* = 8.7 Hz, 2H), 6.73 (d, *J* = 8.7 Hz, 1H), 6.41 (d, *J* = 8.3 Hz, 1H), 6.34 (d, *J* = 7.1 Hz, 1H), 4.53 (h, *J* = 6.0 Hz, 1H), 2.22 (s, 3H), 1.22 (d, *J* = 6.0 Hz, 6H); ^13^C-NMR (126 MHz, DMSO) δ 158.05 (s), 156.55 (s), 156.12 (s), 149.85 (s), 148.66 (s), 138.57 (s), 137.64(s), 136.45 (s), 135.71 (s), 128.73 (s), 127.80 (s), 127.12 (s), 126.66 (s), 122.00 (s), 117.73 (s), 115.62 (s), 111.54 (s), 105.42 (s), 69.45 (s), 51.58 (s), 24.68 (s), 22.33 (s); HRMS (ESI) *m*/*z* calcd. for C_25_H_25_N_3_O_2_ [M + H]^+^: 400.2020, found: 400.2018.

*7-((6-Methylpyridin-2-ylamino)(pyridin-2-yl)methyl)quinolin-8-ol* (**13**): white solid, yield: 16% (33 mg), m.p.: 170–173 °C, C_21_H_18_N_4_O; ^1^H-NMR (500 MHz, DMSO) δ 10.12 (s, 1H), 8.82 (s, 1H), 8.49 (s, 1H), 8.23 (d, *J* = 7.9 Hz, 1H), 7.71 (t, *J* = 7.1 Hz, 1H), 7.55–7.44 (m, 3H), 7.31 (d, *J* = 8.3 Hz, 1H), 7.27–7.18 (m, 3H), 6.76 (d, *J* = 7.1 Hz, 1H), 6.40 (t, *J* = 17.7 Hz, 1H), 6.33 (d, *J* = 6.7 Hz, 1H), 2.19 (s, 3H); ^13^C-NMR (126 MHz, CDCl_3_) δ 161.06 (s), 157.24 (s), 155.73 (s), 149.93 (s), 148.71 (s), 148.25 (s), 138.24 (s), 137.33 (s), 136.80 (s), 135.99 (s), 127.55 (s), 126.75 (s), 125.22 (s), 122.13 (s), 121.96 (s), 121.68 (s), 117.34 (s), 111.37 (s), 104.91 (s), 53.29 (s), 24.15 (s); HRMS (ESI) *m*/*z* calcd. for C_21_H_18_N_4_O [M + H]^+^: 343.1553, found: 343.1555.

*7-(Phenyl(pyrimidin-2-ylamino)methyl)quinolin-8-ol* (**14**): white solid, yield: 15% (30 mg), m.p.: 192–195 °C, C_20_H_16_N_4_O; ^1^H-NMR (500 MHz, DMSO) δ 9.98 (s, 1H), 8.85 (d, *J* = 2.9 Hz, 1H), 8.33–8.27 (m, 3H), 8.08 (d, *J* = 9.4 Hz, 1H), 7.76 (d, *J* = 8.5 Hz, 1H), 7.54 (dd, *J* = 8.2, 4.1 Hz, 1H), 7.44–7.36 (m, 3H), 7.28 (t, *J* = 7.6 Hz, 2H), 7.19 (t, *J* = 7.3 Hz, 1H), 7.00 (d, *J* = 9.4 Hz, 1H), 6.60 (t, *J* = 4.7 Hz, 1H); ^13^C-NMR (126 MHz, DMSO) δ 162.17 (s), 158.51 (s), 149.91 (s), 148.72 (s), 143.59 (s), 138.51 (s), 136.49 (s), 128.63 (s), 127.92 (s), 127.49 (s), 127.33 (s), 127.01 (s), 125.81 (s), 122.13 (s), 117.79 (s), 111.07 (s), 52.31 (s); HRMS (ESI) *m*/*z* calcd. for C_20_H_16_N_4_O [M + H]^+^: 329.1397, found: 329.1397.

*7-((4-Nitrophenyl)(pyrimidin-2-ylamino)methyl)quinolin-8-ol* (**15**): yellow solid, yield: 40% (90 mg), m.p.: 121–123 °C, C_20_H_15_N_5_O_3_; ^1^H-NMR (500 MHz, DMSO) δ 10.17 (s, 1H), 8.84 (d, *J* = 2.4 Hz, 1H), 8.29 (dd, *J* = 8.7, 6.1 Hz, 3H), 8.23 (d, *J* = 9.0 Hz, 1H), 8.15 (d, *J* = 8.4 Hz, 2H), 7.69 (d, *J* = 8.5 Hz, 1H), 7.62 (d, *J* = 8.4 Hz, 2H), 7.53 (dd, *J* = 8.0, 3.9 Hz, 1H), 7.40 (d, *J* = 8.5 Hz, 1H), 7.07 (d, *J* = 9.0 Hz, 1H), 6.62 (t, *J* = 4.3 Hz, 1H); ^13^C-NMR (126 MHz, CDCl_3_) δ 161.62 (s), 158.17 (s), 151.08 (s), 149.79 (s), 148.46 (s), 146.32 (s), 138.11 (s), 136.11 (s), 128.16 (s), 127.79 (s), 126.68 (s), 123.95 (s), 123.56 (s), 121.96 (s), 117.69 (s), 111.07 (s), 51.78 (s); HRMS (ESI) *m*/*z* calcd. for C_20_H_15_N_5_O_3_ [M + H]^+^: 374.1248, found: 374.1250.

*Ethyl 4-((8-hydroxyquinolin-7-yl)(4-(trifluoromethyl)phenyl)methylamino)benzoate* (**16**): white solid, yield: 30% (84 mg), m.p.: 95–98 °C, C_26_H_21_F_3_N_2_O_3_; ^1^H-NMR (500 MHz, DMSO) δ 10.27 (s, 1H), 8.88 (dd, *J* = 4.1, 1.2 Hz, 1H), 8.31 (dd, *J* = 8.3, 1.1 Hz, 1H), 7.72 (d, *J* = 8.3 Hz, 2H), 7.66 (d, *J* = 8.7 Hz, 2H), 7.61 (d, *J* = 8.2 Hz, 2H), 7.57 (dd, *J* = 8.3, 4.2 Hz, 1H), 7.50 (d, *J* = 8.6 Hz, 1H), 7.44–7.39 (m, 2H), 6.71 (d, *J* = 8.7 Hz, 2H), 6.35 (d, *J* = 7.1 Hz, 1H), 4.18 (q, *J* = 7.1 Hz, 2H), 1.24 (t, *J* = 7.1 Hz, 3H); ^13^C-NMR (126 MHz, DMSO) δ 166.21 (s), 152.16 (s), 150.63 (s), 148.95 (s), 147.18 (s), 138.58 (s), 136.59 (s), 131.27 (s), 128.63 (s), 128.30 (s), 128.12–127.65(m), 126.48 (s), 125.87 (q, *J* = 3.2 Hz), 124.07 (s), 122.47 (s), 118.28 (s), 117.62 (s), 112.46 (s), 60.06 (s), 54.22 (s), 14.78 (s); HRMS (ESI) *m*/*z* calcd. for C_26_H_21_F_3_N_2_O_3_ [M + H]^+^: 467.1577, found: 467.1584.

*Ethyl 4-((8-hydroxyquinolin-7-yl)(pyridin-2-yl)methylamino)benzoate* (**17**): white solid, yield: 60% (144 mg), m.p.: 157–159 °C, C_24_H_21_N_3_O_3_; ^1^H-NMR (500 MHz, DMSO) δ 10.22 (s, 1H), 8.84 (s, 1H), 8.54 (s, 1H), 8.23 (d, *J* = 8.1 Hz, 1H), 7.75 (t, *J* = 7.5 Hz, 1H), 7.62 (d, *J* = 8.0 Hz, 2H), 7.53–7.50 (m, 1H), 7.48 (d, *J* = 8.3 Hz, 2H), 7.42 (d, *J* = 6.5 Hz, 1H), 7.32 (d, *J* = 8.4 Hz, 1H), 7.28–7.22 (m, 1H), 6.73 (d, *J* = 7.9 Hz, 2H), 6.34 (d, *J* = 6.5 Hz, 1H), 4.14 (q, *J* = 6.8 Hz, 2H), 1.20 (t, *J* = 6.6 Hz, 3H); ^13^C-NMR (126 MHz, CDCl_3_) δ 165.77 (s), 159.85 (s), 151.43 (s), 150.19 (s), 148.99 (s), 148.35 (s), 138.14 (s), 137.11 (s), 136.06 (s), 130.79 (s), 127.73(s), 126.24 (s), 123.92 (s), 122.56 (s), 122.11 (s), 121.89 (s), 117.63 (s), 116.94 (s), 111.95 (s), 59.58 (s), 54.84 (s), 14.33 (s); HRMS (ESI) *m*/*z* calcd. for C_24_H_21_N_3_O_3_ [M + H]^+^: 400.1656, found: 400.1661.

*7-(Pyridin-2-yl(4-(trifluoromethyl)phenylamino)methyl)quinolin-8-ol* (**18**): white solid, yield: 33% (78 mg), m.p.: 162–164 °C, C_22_H_16_F_3_N_3_O; ^1^H-NMR (500 MHz, DMSO) δ 10.22 (s, 1H), 8.84 (d, *J* = 3.4 Hz, 1H), 8.55 (d, *J* = 4.0 Hz, 1H), 8.23 (d, *J* = 8.2 Hz, 1H), 7.75 (t, *J* = 7.5 Hz, 1H), 7.54–7.47 (m, 3H), 7.34–7.30 (m, 4H), 7.29–7.23 (m, 1H), 6.79 (d, *J* = 8.2 Hz, 2H), 6.31 (d, *J* = 6.8 Hz, 1H); ^13^C-NMR (126 MHz, CDCl_3_) δ 160.33 (s), 150.95 (s), 150.80 (s), 149.54 (s), 148.80 (s), 138.55 (s), 137.57 (s), 136.53 (s), 128.18 (s), 126.65 (s), 126.61 (s), 125.71 (q, *J* = 269.9 Hz), 124.33 (s), 123.01 (s), 122.57 (s), 122.33 (s), 118.10 (s), 116.35 (q, *J* = 31.8 Hz), 112.68 (s), 55.33 (s); HRMS (ESI) *m*/*z* calcd. for C_22_H_16_F_3_N_3_O [M + H]^+^: 396.1318, found: 396.1323.

*7-((3-Fluoro-4-(trifluoromethyl)phenyl)(morpholino)methyl)quinolin-8-ol7-((4-nitrophenylamino) (pyridin-2-yl)methyl)quinolin-8-ol* (**19**): yellow-green (viridian) solid, yield: 14% (31 mg), m.p.: 128–131 °C, C_21_H_16_N_4_O_3_; ^1^H-NMR (500 MHz, DMSO) δ 10.32 (s, 1H), 8.87–8.83 (m, 1H), 8.57 (d, *J* = 3.1 Hz, 1H), 8.25 (d, *J* = 8.2 Hz, 1H), 8.12 (d, *J* = 6.6 Hz, 1H), 7.94 (d, *J* = 8.6 Hz, 2H), 7.77 (t, *J* = 7.6 Hz, 1H), 7.53 (dd, *J* = 7.8, 3.8 Hz, 1H), 7.50–7.41 (m, 2H), 7.34 (d, *J* = 8.5 Hz, 1H), 7.31–7.25 (m, 1H), 6.78 (bs, 2H), 6.39 (d, *J* = 6.7 Hz, 1H); ^13^C-NMR (126 MHz, CDCl_3_) δ 159.21 (s), 153.27 (s), 150.30 (s), 149.11 (s), 148.46 (s), 138.14 (s), 137.25 (s), 136.43 (s), 136.11 (s), 127.87 (s), 126.09 (s), 126.03 (s), 123.23 (s), 122.76 (s), 122.21 (s), 122.03 (s), 117.77 (s), 55.15 (s); HRMS (ESI) *m*/*z* calcd. for C_21_H_16_N_4_O_3_ [M + H]^+^: 373.1295, found: 373.1299.

*7-((6-Methylpyridin-2-ylamino)(4-(trifluoromethyl)phenyl)methyl)quinolin-8-ol* (**20**): white solid, yield: 33% (81 mg), m.p.: 147–150 °C, C_23_H_18_F_3_N_3_O; ^1^H-NMR (500 MHz, DMSO) δ 10.06 (s, 1H), 8.83 (bs, 1H), 8.27 (d, *J* = 8.0 Hz, 1H), 7.66–7.59 (m, 3H), 7.56 (d, *J* = 7.4 Hz, 2H), 7.52 (d, *J* = 2.7 Hz, 1H), 7.40 (d, *J* = 8.3 Hz, 1H), 7.34 (d, *J* = 8.4 Hz, 1H), 7.26 (t, *J* = 7.1 Hz, 1H), 6.93 (d, *J* = 8.1 Hz, 1H), 6.46 (d, *J* = 8.0 Hz, 1H), 6.35 (d, *J* = 6.6 Hz, 1H), 2.19 (s, 3H); ^13^C-NMR (126 MHz, CDCl_3_) δ 157.41 (s), 155.70 (s), 149.70 (s), 148.68 (s), 148.38 (s), 138.15 (s), 137.27 (s), 136.07 (s), 127.79 (s), 127.62 (s), 127.16 (q, *J* = 31.7 Hz), 126.67 (s), 125.11 (dd, *J* = 6.9, 3.8 Hz), 124.96 (s), 124.39 (q, *J* = 271.7 Hz), 121.81 (s), 117.57 (s), 111.44 (s), 105.41 (s), 52.20 (s), 24.24 (s); HRMS (ESI) *m*/*z* calcd. for C_23_H_18_F_3_N_3_O [M + H]^+^: 410.1475, found: 410.1478.

*7-((6-Methylpyridin-2-ylamino)(4-nitrophenyl)methyl)quinolin-8-ol* (**21**): pale yellow solid, yield: 51% (118 mg), m.p.: 159–161 °C, C_22_H_18_N_4_O_3_; ^1^H-NMR (500 MHz, DMSO) δ 10.13 (s, 1H), 8.84 (d, *J* = 2.8 Hz, 1H), 8.28 (d, *J* = 8.1 Hz, 1H), 8.15 (d, *J* = 8.5 Hz, 2H), 7.64–7.56 (m, 3H), 7.53 (dd, *J* = 8.1, 4.0 Hz, 1H), 7.40 (t, *J* = 8.4 Hz, 2H), 7.33–7.19 (m, 1H), 6.96 (d, *J* = 8.3 Hz, 1H), 6.47 (d, *J* = 8.2 Hz, 1H), 6.36 (d, *J* = 7.1 Hz, 1H), 2.19 (s, 3H); ^13^C-NMR (126 MHz, CDCl_3_) δ 157.28 (s), 155.70 (s), 151.99 (s), 149.79 (s), 148.45 (s), 146.17 (s), 138.16 (s), 137.30 (s), 136.10 (s), 128.14 (s), 127.72 (s), 126.66 (s), 124.48 (s), 123.48 (s), 121.92 (s), 117.67 (s), 111.56 (s), 105.57 (s), 51.47 (s), 24.24 (s); HRMS (ESI) *m*/*z* calcd. for C_22_H_18_N_4_O_3_ [M + H]^+^: 387.1452, found: 387.1451.

*2-Methyl-7-((6-methylpyridin-2-ylamino)(4-nitrophenyl)methyl)quinolin-8-ol* (**22**): ivory-white solid, yield: 23% (55 mg), m.p.: 134–136 °C, C_23_H_20_N_4_O_3_; ^1^H-NMR (500 MHz, DMSO) δ 9.64 (s, 1H), 8.20–8.10 (m, 3H), 7.62 (d, *J* = 8.7 Hz, 2H), 7.53 (d, *J* = 8.5 Hz, 1H), 7.44 (d, *J* = 8.4 Hz, 1H), 7.41 (d, *J* = 8.6 Hz, 1H), 7.37 (d, *J* = 8.5 Hz, 1H), 7.30 (dd, *J* = 8.0, 7.4 Hz, 1H), 6.97 (d, *J* = 8.5 Hz, 1H), 6.50 (d, *J* = 8.3 Hz, H), 6.39 (d, *J* = 7.2 Hz, 1 H), 2.69 (d, *J* = 10.1 Hz, 3H), 2.22 (s, 3H); ^13^C-NMR (126 MHz, DMSO) δ 157.73 (s), 157.60 (s), 156.12 (s), 152.53 (s), 149.34 (s), 146.59 (s), 137.87 (s), 137.74 (s), 136.58 (s), 128.52 (s), 126.23 (s), 125.95 (s), 124.69 (s), 123.91 (s), 123.17 (s), 117.96 (s), 111.99 (s), 106.04 (s), 51.87 (s), 25.16 (s), 24.69 (s); HRMS (ESI) *m*/*z* calcd. for C_23_H_20_N_4_O_3_ [M + H]^+^: 401.1608, found: 401.1609.

*2-Methyl-7-((6-methylpyridin-2-ylamino)(4-(trifluoromethyl)phenyl)methyl)quinolin-8-ol* (**23**): beige solid, yield: 71% (180 mg), m.p.: 122–124 °C, C_24_H_20_F_3_N_3_O; ^1^H-NMR (500 MHz, DMSO) δ 9.54 (s, 1H), 8.15 (d, *J* = 8.4 Hz, 1H), 7.82 (d, *J* = 3.9 Hz, 1H), 7.65–7.59 (m, 3H), 7.55 (d, *J* = 7.9 Hz, 2H), 7.39 (d, *J* = 8.4 Hz, 1H), 7.33 (d, *J* = 8.5 Hz, 1H), 7.27 (d, *J* = 6.7 Hz, 1H), 7.07 (t, *J* = 18.0 Hz, 1H), 6.54–6.43 (m, 2H), 2.67 (s, 3H), 2.19 (s, 3H); ^13^C-NMR (126 MHz, CDCl_3_) δ 157.02 (s), 155.79 (s), 149.05 (s), 148.68 (s), 144.77 (s), 137.48 (s), 136.92 (s), 136.10 (s), 127.85 (s), 127.08 (dd, *J* = 63.3, 31.6 Hz), 126.25 (s), 125.75 (s), 124.97 (dd, *J* = 6.8, 3.8 Hz), 124.64 (s), 124.42 (q, *J* = 271.9 Hz), 122.59 (s), 117.34 (s), 117.14 (s), 112.75 (s), 52.45 (s), 24.69 (s), 16.92 (s); HRMS (ESI) *m*/*z* calcd. for C_24_H_20_F_3_N_3_O [M + H]^+^: 424.1631, found: 424.1631.

*7-((6-Methylpyridin-2-ylamino)(4-(trifluoromethoxy)phenyl)methyl)quinolin-8-ol* (**24**): white solid, yield: 34% (87 mg), m.p.: 99–101 °C, C_23_H_18_F_3_N_3_O_2_; ^1^H-NMR (500 MHz, DMSO) δ 10.00 (s, 1H), 8.82 (d, *J* = 2.0 Hz, 1H), 8.27 (d, *J* = 8.1 Hz, 1H), 7.63 (d, *J* = 8.4 Hz, 1H), 7.51 (dd, *J* = 7.6, 3.6 Hz, 1H), 7.45 (d, *J* = 8.1 Hz, 2H), 7.39 (d, *J* = 8.4 Hz, 1H), 7.30 (d, *J* = 8.6 Hz, 1H), 7.28–7.21 (m, 3H), 6.86 (d, *J* = 8.2 Hz, 1H), 6.43 (d, *J* = 8.1 Hz, 1H), 6.34 (d, *J* = 6.9 Hz, 1H), 2.19 (s, 3H); ^13^C-NMR (126 MHz, CDCl_3_) δ 157.44 (s), 155.70 (s), 149.71 (s), 148.31 (s), 146.90 (s), 143.21 (s), 138.17 (s), 137.25 (s), 136.05 (s), 128.89 (s), 127.57 (s), 126.57 (s), 125.30 (s), 121.73 (s), 120.78 (s), 117.42 (s), 116.02 (d, *J* = 260.6 Hz), 111.35 (s), 105.28 (s), 51.14 (s), 24.24 (s); HRMS (ESI) *m*/*z* calcd. for C_23_H_18_F_3_N_3_O_2_ [M + H]^+^: 426.1424, found: 426.1423.

*7-((6-Methylpyridin-2-ylamino)(3-(trifluoromethyl)phenyl)methyl)quinolin-8-ol* (**25**): white solid, yield: 62% (152 mg), m.p.: 148–152 °C, C_23_H_18_F_3_N_3_O; ^1^H-NMR (500 MHz, DMSO) δ 10.07 (s, 1H), 8.83 (s, 1H), 8.27 (d, *J* = 8.2 Hz, 1H), 7.71 (s, 1H), 7.69–7.60 (m, 2H), 7.56–7.46 (m, 3H), 7.44–7.35 (m, 2H), 7.27 (t, *J* = 7.6 Hz, 1H), 6.93 (d, *J* = 8.6 Hz, 1H), 6.45 (d, *J* = 8.2 Hz, 1H), 6.35 (d, *J* = 6.9 Hz, 1H), 2.19 (s, 3H); ^13^C-NMR (126 MHz, CDCl_3_) δ 157.37 (s), 155.70 (s), 149.67 (s), 148.41 (s), 145.29 (s), 138.12 (s), 137.32 (s), 136.08 (s), 131.32 (s), 129.29 (s), 128.88 (d, *J* = 31.4 Hz), 127.60 (s), 126.44 (s), 125.22 (q, *J* = 48.0 Hz), 125.03 (s), 123.34 (s), 121.82 (s), 117.62 (s), 111.49 (s), 105.40 (s), 51.40 (s), 24.21 (s); HRMS (ESI) *m*/*z* calcd. for C_23_H_18_F_3_N_3_O [M + H]^+^: 410.1475, found: 410.1476.

*7-((3-Methylpyridin-2-ylamino)(4-(trifluoromethyl)phenyl)methyl)quinolin-8-ol* (**26**): white solid, yield: 18% (44 mg), m.p.: 148–151 °C, C_23_H_18_F_3_N_3_O; ^1^H-NMR (500 MHz, DMSO) δ 10.09 (s, 1H), 8.83 (d, *J* = 3.1 Hz, 1H), 8.28 (d, *J* = 8.2 Hz, 1H), 7.81 (d, *J* = 4.1 Hz, 1H), 7.69 (d, *J* = 8.4 Hz, 1H), 7.62 (d, *J* = 7.9 Hz, 2 H), 7.58–7.48 (m, 3H), 7.39 (d, *J* = 8.4 Hz, 1H), 7.27 (d, *J* = 6.7 Hz, 1H), 7.11 (d, *J* = 8.1 Hz, 1H), 6.53–6.45 (m, 2H), 2.19 (s, 3H); ^13^C-NMR (126 MHz, CDCl_3_) δ 155.75 (s), 149.93 (s), 148.57 (s), 148.34 (s), 144.73 (s), 138.18 (s), 136.95 (s), 136.06 (s), 127.93 (s), 127.67 (s), 127.32 (s), 127.10 (q, *J* = 31.3 Hz), 125.00 (q, *J* = 2.9 Hz), 124.88 (s), 124.42 (q, *J* = 272.0 Hz), 121.79 (s), 117.48 (s), 117.17 (s), 112.75 (s), 52.33 (s), 16.95 (s); HRMS (ESI) *m*/*z* calcd. for C_23_H_18_F_3_N_3_O [M + H]^+^: 410.1475, found: 410.1479.

*7-((4-Methylpyridin-2-ylamino)(4-(trifluoromethyl)phenyl)methyl)quinolin-8-ol* (**27**): mulatto solid, yield: 15% (37 mg), m.p.: 151–153 °C, C_23_H_18_F_3_N_3_O; ^1^H-NMR (500 MHz, DMSO) δ 10.06 (s, 1H), 8.86 (dd, *J* = 4.1, 1.4 Hz, 1H), 8.30 (dd, *J* = 8.3, 1.3 Hz, 1H), 7.80 (d, *J* = 5.2 Hz, 1H), 7.66 (d, *J* = 8.2 Hz, 2H), 7.59 (d, *J* = 8.5 Hz, 1H), 7.57–7.53 (m, 3H), 7.42 (d, *J* = 8.6 Hz, 1H), 7.37 (d, *J* = 8.4 Hz, 1H), 6.97 (d, *J* = 8.4 Hz, 1H), 6.54 (s, 1H), 6.36 (d, *J* = 5.1 Hz, 1H), 2.15 (s, H); ^13^C-NMR (126 MHz, DMSO) δ 158.49 (s), 150.16 (s), 149.13 (s), 148.81 (s), 147.58 (s), 147.46 (s), 138.59 (s), 136.52 (s), 128.27 (s), 128.05 (s), 127.60 (q, *J* = 31.5 Hz), 127.03 (s), 125.58(q, *J* = 3.5 Hz), 125.49 (s), 124.83 (q, *J* = 271.6 Hz), 122.24 (s), 117.96 (s), 114.48 (s), 109.35 (s), 51.91 (s), 21.09 (s); HRMS (ESI) *m*/*z* calcd. for C_23_H_18_F_3_N_3_O [M + H]^+^: 410.1475, found: 410.1477.

*7-((3-Fluoro-5-(trifluoromethyl)phenyl)(6-methylpyridin-2-ylamino)methyl)quinolin-8-ol* (**28**): white solid, yield: 35% (90 mg), m.p.: 163–165 °C, C_23_H_17_F_4_N_3_O; ^1^H-NMR (500 MHz, DMSO) δ 10.19 (s, 1H), 8.87 (dd, *J* = 4.0, 1.2 Hz, 1H), 8.31 (dd, *J* = 8.3, 1.1 Hz, 1H), 7.67 (d, *J* = 8.6 Hz, 1H), 7.60 (s, 1H), 7.56 (dd, *J* = 8.3, 4.2 Hz, 1H), 7.51 (d, *J* = 9.2 Hz, 2H), 7.45 (d, *J* = 8.4 Hz, 2H), 7.31 (t, *J* = 7.7 Hz, 1H), 6.95 (d, *J* = 8.7 Hz, 1H), 6.50 (d, *J* = 8.3 Hz, 1H), 6.40 (d, *J* = 7.2 Hz, 1H), 2.22 (s, 3H); ^13^C NMR (126 MHz, DMSO) δ 162.40 (d, *J* = 246.4 Hz), 157.62 (s), 156.15 (s), 150.19 (s), 149.34 (d, *J* = 6.8 Hz), 148.95 (s), 138.57 (s), 137.84 (s), 136.57 (s), 131.17 (qd, *J* = 32.7, 8.7 Hz), 128.16 (s), 126.69 (s), 124.86 (s), 123.82 (dq, *J* = 272.8, 2.6 Hz), 122.40 (s), 120.30–120.02 (m), 118.39 (d, *J* = 21.8 Hz), 118.22 (s), 112.16 (s), 111.39 (dq, *J* = 25.1, 3.4 Hz), 106.01 (s), 51.75 (s), 24.64 (s); HRMS (ESI) *m*/*z* calcd. for C_23_H_17_F_4_N_3_O [M + H]^+^: 428.1381, found: 428.1388.

*7-((3,5-bis(Trifluoromethyl)phenyl)(6-methylpyridin-2-ylamino)methyl)quinolin-8-ol* (**29**): white solid, yield: 43% (123 mg), m.p.: 144–146 °C, C_24_H_17_F_6_N_3_O; ^1^H-NMR (500 MHz, DMSO) δ 10.20 (s, 1H), 8.84 (bs, 1H), 8.28 (d, *J* = 8.1 Hz, 1H), 8.03 (s, 2H), 7.92 (s, 1H), 7.69 (d, *J* = 8.4 Hz, 1H), 7.58–7.48 (m, 2H), 7.43 (d, *J* = 8.4 Hz, 1H), 7.29 (t, *J* = 7.6 Hz, 1H), 6.98 (d, *J* = 8.3 Hz, 1H), 6.48 (d, *J* = 8.1 Hz, 1H), 6.38 (d, *J* = 6.9 Hz, 1H), 2.19 (s, 3H); ^13^C-NMR (126 MHz, CDCl_3_) δ 157.12 (s), 155.69 (s), 149.81 (s), 148.56 (s), 147.44 (s), 138.11 (s), 137.44 (s), 136.13 (s), 130.08 (q, *J* = 32.3 Hz), 127.78 (s), 127.68 (s), 126.08 (s), 124.14 (s), 123.39 (q, *J* = 272.5 Hz), 122.01 (s), 120.48 (s), 117.87 (s), 111.83 (s), 105.67 (s), 51.55 (s), 24.13 (s); HRMS (ESI) *m*/*z* calcd. for C_24_H_17_F_6_N_3_O [M + H]^+^: 478.1349, found: 478.1353.

*7-((4-Fluoro-3-(trifluoromethyl)phenyl)(6-methylpyridin-2-ylamino)methyl)quinolin-8-ol* (**30**): white solid, yield: 90% (231 mg), m.p.: 148–151 °C, C_23_H_17_F_4_N_3_O; ^1^H-NMR (500 MHz, DMSO) δ 10.10 (s, 1H), 8.83 (s, 1H), 8.27 (d, *J* = 8.1 Hz, 1H), 7.73 (d, *J* = 6.2 Hz, 1H), 7.65 (d, *J* = 8.7 Hz, 2H), 7.52 (dd, *J* = 8.0, 3.9 Hz, 1H), 7.45–7.36 (m, 3H), 7.27 (t, *J* = 7.7 Hz, 1H), 6.87 (d, *J* = 8.5 Hz, 1H), 6.44 (t, *J* = 9.4 Hz, 1H), 6.34 (t, *J* = 15.0 Hz, 1H), 2.19 (s, 3H); ^13^C-NMR (126 MHz, DMSO) δ 159.53–156.77 (m), 157.71 (s), 156.16 (s), 150.13 (s), 148.90 (s), 141.39 (d, *J* = 3.4 Hz), 138.55 (s), 137.76 (d, *J* = 13.0 Hz), 136.55 (s), 134.27 (d, *J* = 8.5 Hz), 129.47–121.35 (m), 128.10(s), 126.68 (s), 125.77 (q, *J* = 4.7 Hz), 125.26 (s), 122.31 (s), 118.12 (s), 117.48 (d, *J* = 20.4 Hz), 116.78–116.78 (m), 112.03 (s), 105.88 (s), 51.42 (s), 24.65 (s); HRMS (ESI) *m*/*z* calcd. for C_23_H_17_F_4_N_3_O [M + H]^+^: 428.1381, found: 428.1385.

*7-((2,4-bis(Trifluoromethyl)phenyl)(6-methylpyridin-2-ylamino)methyl)quinolin-8-*ol (**31**): light rose solid, yield: 22% (63 mg), m.p.: decomposed 230 °C, C_24_H_17_F_6_N_3_O; ^1^H-NMR (500 MHz, DMSO) δ 9.83 (s, 1H), 8.87 (d, *J* = 2.9 Hz, 1H), 8.33 (d, *J* = 8.0 Hz, 1H), 8.16 (d, *J* = 8.2 Hz, 1H), 8.11 (d, *J* = 8.2 Hz, 1H), 8.01 (s, 1H), 7.63 (dd, *J* = 8.6, 4.1 Hz, 1H), 7.59 (d, *J* = 7.8 Hz, 1H), 7.36 (d, *J* = 7.6 Hz, 1H), 7.25 (t, *J* = 7.7 Hz, 1H), 6.92 (d, *J* = 8.0 Hz, 1H), 6.63 (d, *J* = 8.0 Hz, 1H), 6.37 (d, *J* = 7.2 Hz, 1H), 2.17 (s, 3H); HRMS (ESI) *m*/*z* calcd. for C_24_H_17_F_6_N_3_O [M + H]^+^: 478.1349, found: 478.1356.

*7-((3,4-Difluorophenyl)(6-methylpyridin-2-ylamino)methyl)quinolin-8-ol* (**32**): white solid, yield: 53% (120 mg), m.p.: 175–178 °C, C_22_H_17_F_2_N_3_O; ^1^H-NMR (500 MHz, DMSO) δ 10.10 (s, 1H), 8.86 (dd, *J* = 4.0, 1.2 Hz, 1H), 8.30 (dd, *J* = 8.2, 1.1 Hz, 1H), 7.63 (d, *J* = 8.5 Hz, 1H), 7.55 (dd, *J* = 8.3, 4.2 Hz, 1H), 7.42 (d, *J* = 8.6 Hz, 1H), 7.40–7.27 (m, 4 H), 7.24–7.15 (m, 1H), 6.83 (d, *J* = 8.8 Hz, 1H), 6.46 (d, *J* = 8.3 Hz, 1H), 6.38 (d, *J* = 7.2 Hz, 1H), 2.26 (d, *J* = 40.6 Hz, 3H); ^13^C-NMR (126 MHz, CDCl_3_) δ 157.32 (s), 155.72 (s), 149.61 (s), 149.19 (dd, *J* = 245.7, 13.0 Hz), 148.38 (s), 148.09 (dd, *J* = 244.0, 13.1 Hz), 141.68 (s), 138.14 (s), 137.30 (s), 136.07 (s),127.59 (s), 126.43 (s), 125.08 (s), 123.70 (s), 121.80 (s), 117.58 (s), 117.16 (d, *J* = 16.9 Hz), 115.74 (d, *J* = 17.3 Hz), 111.50 (s), 105.33 (s), 50.96 (s), 24.22 (s); HRMS (ESI) *m*/*z* calcd. for C_22_H_17_F_2_N_3_O [M + H]^+^: 378.1412, found: 378.1413.

*7-((Pyrimidin-2-ylamino)(4-(trifluoromethyl)phenyl)methyl)quinolin-8-ol* (**33**): white solid, yield: 32% (76 mg), m.p.: 160–163 °C, C_21_H_15_F_3_N_4_O; ^1^H-NMR (500 MHz, DMSO) δ 10.10 (s, 1H), 8.83 (d, *J* = 2.3 Hz, 1H), 8.33–8.23 (m, 3H), 8.18 (d, *J* = 9.1 Hz, 1H), 7.70 (d, *J* = 8.5 Hz, 1H), 7.64 (d, *J* = 7.9 Hz, 2H), 7.57 (d, *J* = 7.8 Hz, 2H), 7.52 (dd, *J* = 8.0, 3.9 Hz, 1H), 7.39 (d, *J* = 8.5 Hz, 1H), 7.04 (d, *J* = 9.1 Hz, 1H), 6.60 (t, *J* = 4.3 Hz, 1H); ^13^C-NMR (126 MHz, CDCl_3_) δ 162.48 (s), 158.95 (s), 150.50 (s), 149.22 (s), 148.76 (s), 138.91 (s), 136.90 (s), 128.58 (s), 128.51 (s), 128.14 (q, *J* = 31.7 Hz), 127.53 (s), 126.01 (q, *J* = 3.7 Hz), 125.23 (s), 125.17 (q, *J* = 272.1 Hz), 122.68 (s), 118.42 (s), 111.75 (s), 52.59 (s); HRMS (ESI) *m*/*z* calcd. for C_21_H_15_F_3_N_4_O [M + H]^+^: 397.1271, found: 397.1273.

*7-((4-Methylpyrimidin-2-ylamino)(4-(trifluoromethyl)phenyl)methyl)quinolin-8-ol* (**34**): white solid, yield: 46% (113 mg), m.p.: 147–149 °C, C_22_H_17_F_3_N_4_O; ^1^H-NMR (500 MHz, DMSO) δ 10.12 (s, 1H), 8.86 (dd, *J* = 4.1, 1.5 Hz, 1H), 8.31 (dd, *J* = 8.3, 1.3 Hz, 1H), 8.16 (d, *J* = 5.0 Hz, 1H), 8.11 (d, *J* = 9.4 Hz, 1H), 7.75 (d, *J* = 8.6 Hz, 1H), 7.66 (d, *J* = 8.3 Hz, 2H), 7.60 (d, *J* = 8.2 Hz, 2H), 7.55 (dd, *J* = 8.3, 4.2 Hz, 1H), 7.42 (d, *J* = 8.6 Hz, 1H), 7.09 (d, *J* = 9.4 Hz, 1H), 6.52 (d, *J* = 5.0 Hz, 1H), 2.26 (s, 3H); ^13^C-NMR (126 MHz, DMSO) δ 167.94 (s), 162.02 (s), 158.11 (s), 150.04 (s), 148.83 (s), 148.65 (s), 138.53 (s), 136.53 (s), 128.15 (s), 128.10 (s), 127.67 (q, *J* = 31.5 Hz), 127.22 (s), 125.61 (q, *J* = 3.5 Hz), 125.01 (s), 124.81 (q, *J* = 271.6 Hz), 122.29 (s), 118.03 (s), 110.84 (s), 52.09 (s), 24.11 (s).

*7-((4-Methylpyrimidin-2-ylamino)(4-nitrophenyl)methyl)quinolin-8-ol* (**35**): white solid, yield: 19% (44 mg), m.p.: 136–138 °C, C_21_H_17_N_5_O_3_; ^1^H-NMR (500 MHz, DMSO) δ 10.15 (s, 1H), 8.84 (d, *J* = 2.5 Hz, 1H), 8.28 (d, *J* = 7.9 Hz, 1H), 8.18–8.09 (m, 4H), 7.71 (d, *J* = 8.5 Hz, 1H), 7.62 (d, *J* = 8.4 Hz, 2H), 7.53 (dd, *J* = 8.1, 4.0 Hz, 1H), 7.40 (d, *J* = 8.5 Hz, 1H), 7.09 (d, *J* = 9.2 Hz, 1H), 6.51 (d, *J* = 4.8 Hz, 1H), 2.24 (s, 3 H); ^13^C-NMR (126 MHz, CDCl_3_) δ 168.00 (s), 161.51 (s), 158.09 (s), 151.33 (s), 149.69 (s), 148.45 (s), 146.26 (s), 138.10 (s), 136.10 (s), 128.10 (s), 127.75 (s), 126.73 (s), 124.09 (s), 123.53 (s), 121.94 (s), 117.68 (s), 110.53 (s), 51.69 (s), 23.69 (s); HRMS (ESI) *m*/*z* calcd. for C_21_H_17_N_5_O_3_ [M + H]^+^: 388.1404, found: 388.1409.

*7-((4-Methylpyrimidin-2-ylamino)(4-(pentafluorothio)phenyl)methyl)quinolin-8-ol* (**36**): white solid, yield: 68% (191 mg), m.p.: 164–165 °C, C_21_H_17_F_5_N_4_OS; ^1^H-NMR (500 MHz, DMSO) δ 10.16 (s, 1H), 8.87 (dd, *J* = 4.1, 1.5 Hz, 1H), 8.31 (dd, *J* = 8.3, 1.5 Hz, 1H), 8.17 (d, *J* = 5.0 Hz, 1H), 8.12 (d, *J* = 9.4 Hz, 1H), 7.84 (d, *J* = 8.9 Hz, 2H), 7.75 (d, *J* = 8.6 Hz, 1H), 7.59 (d, *J* = 8.5 Hz, 2H), 7.55 (dt, *J* = 12.4, 6.2 Hz, 1H), 7.43 (d, *J* = 8.6 Hz, 1H), 7.07 (d, *J* = 9.4 Hz, 1H), 6.52 (t, *J* = 6.2 Hz, 1H), 2.26 (s, 3H); ^13^C-NMR (126 MHz, DMSO) δ 168.2–167.74 (m), 161.98 (s), 158.28–157.83 (m), 152.05–151.32 (m), 150.08 (s), 148.86 (s), 148.60 (s), 138.53 (s), 136.54 (s), 128.25(s), 128.16 (s), 127.13 (s), 126.36–126.19 (m), 124.73 (s), 122.34 (s), 118.09 (s), 110.92 (s), 51.90 (s), 24.11 (s); HRMS (ESI) *m*/*z* calcd. for C_21_H_17_F_5_N_4_OS [M + H]^+^: 469.1116, found: 469.1118.

*7-((4-Fluorophenyl)(4-methylpyrimidin-2-ylamino)methyl)quinolin-8-ol* (**37**): white solid, yield: 16% (35 mg), m.p.: 156–158 °C, C_21_H_17_FN_4_O; ^1^H-NMR (500 MHz, DMSO) δ 9.97 (s, 1H), 8.82 (d, *J* = 2.8 Hz, 1H), 8.27 (d, *J* = 8.2 Hz, 1H), 8.12 (d, *J* = 4.9 Hz, 1H), 7.98 (d, *J* = 9.5 Hz, 1H), 7.74 (d, *J* = 8.5 Hz, 1H), 7.51 (dd, *J* = 8.2, 4.1 Hz, 1H), 7.42–7.31 (m, 3H), 7.12–7.03 (m, 2H), 6.98 (t, *J* = 12.1 Hz, 1H), 6.47 (d, *J* = 4.9 Hz, 1H), 2.22 (s, H); ^13^C-NMR (126 MHz, CDCl_3_) δ 167.90 (s), 161.58 (s), 158.06 (s), 160.95 (d, *J* = 242.0 Hz), 149.37 (s), 148.30 (s), 139.53 (s), 138.07 (s), 136.05 (s), 128.87 (d, *J* = 8.1 Hz), 127.51 (s), 126.74 (s), 125.34 (s), 121.71 (s), 117.43 (s), 114.85 (d, *J* = 21.2 Hz), 110.20 (s), 51.25 (s), 23.63 (s); HRMS (ESI) *m*/*z* calcd. for C_21_H_17_FN_4_O [M + H]^+^: 361.1459, found: 361.1460.

*7-((4-Iodophenyl)(4-methylpyrimidin-2-ylamino)methyl)quinolin-8-ol* (**38**): white solid, yield: 18% (51 mg), m.p.: 139–142 °C, C_21_H_17_IN_4_O; ^1^H-NMR (500 MHz, DMSO) δ 9.99 (s, 1 H), 8.82 (s, 1 H), 8.27 (d, *J* = 8.2 Hz, 1 H), 8.12 (d, *J* = 4.3 Hz, 1 H), 7.96 (d, *J* = 9.3 Hz, 1 H), 7.70 (d, *J* = 8.4 Hz, 1 H), 7.61 (d, *J* = 7.8 Hz, 2 H), 7.52 (dd, *J* = 7.8, 3.8 Hz, 1 H), 7.37 (d, *J* = 8.4 Hz, 1 H), 7.15 (d, *J* = 7.6 Hz, 2 H), 6.91 (d, *J* = 9.3 Hz, 1 H), 6.48 (d, *J* = 4.5 Hz, 1 H), 2.22 (s, 3 H) ^13^C-NMR (126 MHz, DMSO) δ 167.89 (s), 162.01 (s), 158.01 (s), 149.90 (s), 148.77 (s), 143.75 (s), 138.51 (s), 137.34 (s), 136.50 (s), 129.86 (s), 128.00 (s), 127.23 (s), 125.35 (s), 122.20 (s), 117.91 (s), 110.69 (s), 92.81 (s), 51.91 (s), 24.06 (s); HRMS (ESI) *m*/*z* calcd. for C_21_H_17_IN_4_O [M + H]^+^: 469.0520, found: 469.0525.

*7-((4-Bromophenyl)(4-methylpyrimidin-2-ylamino)methyl)quinolin-8-ol* (**39**): ivory-white solid, yield: 29% (73 mg), m.p.: 143–145 °C, C_21_H_17_BrN_4_O; ^1^H-NMR (500 MHz, DMSO) δ 10.00 (s, 1H), 8.82 (s, 1H), 8.27 (d, *J* = 7.9 Hz, 1H), 8.12 (d, *J* = 4.5 Hz, 1H), 7.98 (d, *J* = 9.2 Hz, 1H), 7.71 (d, *J* = 8.5 Hz, 1H), 7.52 (dd, *J* = 7.8, 3.8 Hz, 1H), 7.44 (d, *J* = 8.1 Hz, 2H), 7.38 (d, *J* = 8.4 Hz, 1H), 7.30 (d, *J* = 8.0 Hz, 2H), 6.94 (d, *J* = 9.3 Hz, 1H), 6.48 (d, *J* = 4.6 Hz, 1H), 2.22 (s, 3H); ^13^C-NMR (126 MHz, CDCl_3_) δ 167.85 (s), 161.57 (s), 149.46 (s), 148.34 (s), 142.86 (s), 138.07 (s), 136.06 (s), 131.04 (s), 129.24 (s), 127.57 (s), 126.77 (s), 124.91 (s), 121.77 (s), 119.59 (s), 117.48 (s), 110.27 (s), 51.39 (s), 24.39 (s); HRMS (ESI) *m*/*z* calcd. for C_21_H_17_BrN_4_O [M + H]^+^: 421.0659, found: 421.0664.

*7-((4-Chlorophenyl)(4-methylpyrimidin-2-ylamino)methyl)quinolin-8-ol* (**40**): white solid, yield: 40% (90 mg), m.p.: 139–141 °C, C_21_H_17_ClN_4_O; ^1^H-NMR (500 MHz, DMSO) δ 10.04 (s, 1H), 8.86 (dd, *J* = 3.9, 1.3 Hz, 1H), 8.30 (d, *J* = 8.3 Hz, 1H), 8.15 (d, *J* = 4.9 Hz, 1H), 8.02 (d, *J* = 9.4 Hz, 1H), 7.75 (d, *J* = 8.5 Hz, 1H), 7.55 (dd, *J* = 8.2, 4.1 Hz, 1H), 7.44–7.37 (m, 3H), 7.34 (d, *J* = 8.5 Hz, 2H), 6.98 (d, *J* = 9.4 Hz, 1H), 6.51 (d, *J* = 5.0 Hz, 1H), 2.25 (s, 3H); ^13^C-NMR (126 MHz, DMSO) δ 167.90 (s), 162.01 (s), 158.05 (s), 149.90 (s), 148.78 (s), 142.86 (s), 138.51 (s), 136.51 (s), 131.53 (s), 129.29 (s), 128.57 (s), 128.00 (s), 127.19 (s), 125.43 (s), 122.21 (s), 117.92 (s), 110.70 (s), 51.74 (s), 24.09 (s); HRMS (ESI) *m*/*z* calcd. for C_21_H_17_ClN_4_O [M + H]^+^: 377.1164, found: 377.1167.

*7-((4-Methylpyrimidin-2-ylamino)(3-(trifluoromethyl)phenyl)methyl)quinolin-8-ol* (**41**): white solid, yield: 31% (76 mg), m.p.: 160–163 °C, C_22_H_17_F_3_N_4_O; ^1^H-NMR (500 MHz, DMSO) δ 10.08 (s, 1H), 8.91–8.73 (m, 1H), 8.27 (d, *J* = 8.2 Hz, 1H), 8.21–8.06 (m, 2H), 7.81–7.70 (m, 2H), 7.67 (d, *J* = 7.0 Hz, 1H), 7.59–7.44 (m, 3H), 7.40 (d, *J* = 8.5 Hz, 1H), 7.07 (d, *J* = 9.4 Hz, 1H), 6.49 (d, *J*= 4.7 Hz, 1H), 2.23 (s, 3H); ^13^C-NMR (126 MHz, DMSO) δ 168.00 (s), 161.96 (s), 149.96 (s), 148.87 (s), 145.32 (s), 138.50 (s), 136.54 (s), 131.70 (s), 129.79 (s), 129.36 (q, *J* = 31.4 Hz), 128.07 (s), 126.93 (s), 125.18 (s), 124.74 (q, *J* = 272.43 Hz), 123.87 (q, *J* = 3.6 Hz), 123.68 (q, *J* = 3.7 Hz), 122.30 (s), 118.09 (s), 110.85 (s), 51.95 (s), 24.16 (s); HRMS (ESI) *m*/*z* calcd. for C_22_H_17_F_3_N_4_O [M + H]^+^: 411.1427, found: 411.1428.

*7-((3,5-bis(Trifluoromethyl)phenyl)(4-methylpyrimidin-2-ylamino)methyl)quinolin-8-ol* (**42**): white solid, yield: 59% (169 mg), m.p.: 128–130 °C, C_23_H_16_F_6_N_4_O; ^1^H-NMR (500 MHz, DMSO) δ 10.24 (s, 1H), 8.84 (d, *J* = 2.8 Hz, 1H), 8.36–8.23 (m, 2H), 8.15 (d, *J* = 4.8 Hz, 1H), 8.08 (s, 2H), 7.93 (s, 1H), 7.77 (d, *J* = 8.5 Hz, 1H), 7.53 (dd, *J* = 8.2, 4.1 Hz, 1H), 7.42 (d, *J* = 8.5 Hz, 1H), 7.13 (d, *J* = 9.4 Hz, 1H), 6.52 (d, *J* = 4.9 Hz, 1H), 2.24 (s, 3H); ^13^C-NMR (126 MHz, CDCl_3_) δ 167.63 (s), 161.38 (s), 157.64 (s), 149.65 (s), 148.57 (s), 146.83 (s), 138.06 (s), 136.14 (s), 130.19 (q, *J* = 32.9 Hz), 127.80 (s), 127.63 (s), 125.96 (s), 123.97 (s), 123.35 (q, *J* = 272.8 Hz), 122.02 (s), 120.80–120.55 (m), 117.93 (s), 110.72 (s), 51.60 (s), 23.62 (s); HRMS (ESI) *m*/*z* calcd. for C_23_H_16_F_6_N_4_O [M + H]^+^: 479.1301, found: 479.1304.

*7-((2,4-bis(Trifluoromethyl)phenyl)(4-methylpyrimidin-2-ylamino)methyl)quinolin-8-ol* (**43**): white solid, yield: 16% (46 mg), m.p.: 199–201 °C, C_23_H_16_F_6_N_4_O; ^1^H-NMR (500 MHz, DMSO) δ 9.97 (s, 1H), 8.81 (d, *J* = 2.4 Hz, 1H), 8.28 (d, *J* = 7.8 Hz, 1H), 8.08 (s, 1H), 8.03 (d, *J* = 6.0 Hz, 1H), 7.99–7.95 (m, 2H), 7.80 (bs, 1H), 7.52 (dd, *J* = 8.1, 3.9 Hz, 1H), 7.33 (dd, *J* = 11.7, 9.2 Hz, 2H), 7.21 (d, *J* = 4.7 Hz, 1H), 6.47 (d, *J* = 4.7 Hz, 1H), 2.19 (s, 3H); ^13^C-NMR (126 MHz, DMSO) δ 169.04 (s), 161.45 (s), 158.49 (s), 150.79 (s), 148.79 (s), 138.45 (s), 136.53 (s), 131.63 (s), 129.90–129.72 (m), 128.66 (q, *J* = 31.4 Hz), 128.55 (q, *J* = 32.7 Hz), 128.26 (s), 127.05–126.81 (m), 124.01 (q, *J* = 275.4 Hz), 123.97 (q, *J* = 272.2 Hz), 123.57–123.27 (m), 122.34 (s), 117.30 (s), 116.90 (s), 110.80 (s), 49.76 (s), 24.05 (s); HRMS (ESI) *m*/*z* calcd. for C_23_H_16_F_6_N_4_O [M + H]^+^: 479.1301, found: 479.1307.

*7-((4-Fluoro-3-(trifluoromethyl)phenyl)(4-methylpyrimidin-2-ylamino)methyl)quinolin-8-ol* (**44**): white solid, yield: 25% (64 mg), m.p.: 89–92 °C, C_22_H_16_F_4_N_4_O; ^1^H-NMR (500 MHz, DMSO) δ 10.10 (s, 1H), 8.82 (bs, 1H), 8.26 (d, *J* = 5.6 Hz, 1H), 8.19–8.08 (m, 2H), 7.83–7.64 (m, 3H), 7.51 (dd, *J* = 5.6, 3.6 Hz, 1 H), 7.43–7.31 (m, 2H), 7.01 (d, *J* = 7.7 Hz, 1H), 6.49 (bs, 1H), 2.22 (s, 3H); ^13^C-NMR (126 MHz, CDCl_3_) δ 167.49 (bs), 161.44 (s), 157.95–157.18 (m), 157.63 (d, *J* = 252.6 Hz), 149.50 (s), 148.44 (s), 140.49 (s), 138.05 (s), 136.09 (s), 133.81 (d, *J* = 8.5 Hz), 127.67 (s), 126.23 (s), 125.27 (d, *J* = 3.4 Hz), 124.56 (s), 123.77 (s), 121.87 (s), 117.70 (s), 117.08 (d, *J* = 20.5 Hz), 110.49 (s), 51.15 (s), 23.62 (s); HRMS (ESI) *m*/*z* calcd. for C_22_H_16_F_4_N_4_O [M + H]^+^: 429.1333, found: 429.1337.

*7-((3-Fluoro-5-(trifluoromethyl)phenyl)(4-methylpyrimidin-2-ylamino)methyl)quinolin-8-ol* (**45**): ivory-white solid, yield: 15% (39 mg), m.p.: decomposed 92 °C, C_22_H_16_F_4_N_4_O; ^1^H-NMR (500 MHz, DMSO) δ 10.22 (s, 1H), 8.87 (d, *J* = 3.0 Hz, 1H), 8.31 (d, *J* = 8.1 Hz, 1H), 8.21 (d, *J* = 9.5 Hz, 1H), 8.18 (d, *J* = 4.8 Hz, 1H), 7.77 (d, *J* = 8.5 Hz, 1H), 7.65 (s, 1H), 7.58–7.52 (m, 3H), 7.44 (d, *J* = 8.5 Hz, 1H), 7.09 (d, *J* = 9.4 Hz, 1H), 6.55 (d, *J* = 4.9 Hz, 1H), 2.27 (s, 3H); ^13^C-NMR (126 MHz, DMSO) δ 162.39 (d, *J* = 246.5 Hz), 161.86 (s), 150.03 (s), 148.97 (s), 148.74 (d, *J* = 6.8 Hz), 138.51 (s), 136.57 (s), 131.72–131.06 (m), 128.19 (s), 126.64 (s), 124.61 (s), 123.80 (dq, *J* = 272.7, 4.2 Hz), 122.42 (s), 120.28–119.83 (m), 118.40 (d, *J* = 22.0 Hz), 118.26 (s), 111.74–111.36 (m), 111.08 (s), 51.86 (s), 24.10 (s); HRMS (ESI) *m*/*z* calcd. for C_22_H_16_F_4_N_4_O [M + H]^+^: 429.1333, found: 429.1336.

*7-((5-Bromopyrimidin-2-ylamino)(4-(trifluoromethyl)phenyl)methyl)quinolin-8-ol* (**46**): white solid, yield: 15% (43 mg), m.p.: 146–148 °C, C_21_H_14_BrF_3_N_4_O; ^1^H-NMR (500 MHz, DMSO) δ 10.12 (s, 1H), 8.84 (d, *J* = 2.2 Hz, 1H), 8.50 (d, *J* = 8.9 Hz, 1H), 8.41 (s, 2H), 8.28 (d, *J* = 7.9 Hz, 1H), 7.70–7.61 (m, 3H), 7.59–7.49 (m, 3H), 7.39 (d, *J* = 8.4 Hz, 1H), 6.96 (d, *J* = 8.8 Hz, 1H); ^13^C-NMR (126 MHz, CDCl_3_) δ 160.07 (s), 149.81 (s), 148.45 (s), 147.38 (s), 138.07 (s), 136.11 (s), 127.82 (s), 127.75 (s), 127.19 (q, *J* = 31.8 Hz), 126.52 (s), 125.26 (q, *J* = 4.0 Hz), 124.77 (q, *J* = 272.0 Hz), 123.91 (s), 121.93 (s), 117.62 (s), 106.09 (s), 52.12 (s); HRMS (ESI) *m*/*z* calcd. for C_21_H_14_BrF_3_N_4_O [M + H]^+^: 475.0376, found: 475.0384.

*7-((2-Hydroxyphenyl)(4-methylpyrimidin-2-ylamino)methyl)quinolin-8-ol* (**47**): caesious solid, yield: 15% (32 mg), m.p.: decomposed 179 °C, C_21_H_18_N_4_O_2_; ^1^H-NMR (500 MHz, DMSO) δ 9.81 (s, 1 H), 9.49 (s, 1 H), 8.82 (d, *J* = 2.9 Hz, 1 H), 8.28 (d, *J* = 7.7 Hz, 1 H), 8.11 (d, *J* = 4.9 Hz, 1H), 7.59 (d, *J* = 8.5 Hz, 1H), 7.51 (dd, *J* = 8.3, 4.2 Hz, 1H), 7.49 (d, *J* = 9.2 Hz, 1H), 7.34 (d, *J* = 8.5 Hz, 1H), 7.24 (d, *J* = 7.2 Hz, 1H), 7.04 (t, *J* = 7.1 Hz, 1H), 6.99 (d, *J* = 9.0 Hz, 1H), 6.77 (d, *J* = 7.9 Hz, 1H), 6.72 (t, *J* = 7.4 Hz, 1H), 6.46 (d, *J* = 4.9 Hz, 1H), 2.23 (s, 3H); ^13^C-NMR (126 MHz, DMSO) δ 167.64 (s), 161.85 (s), 157.94 (s), 155.36 (s), 150.18 (s), 148.47 (s), 138.47(s), 136.39 (s), 129.22 (s), 128.76 (s), 128.12 (s), 127.85 (s), 127.80 (s), 125.59 (s), 121.89 (s), 119.01 (s), 116.93 (s), 115.63 (s), 110.29 (s), 48.89 (s), 24.13 (s); HRMS (ESI) *m*/*z* calcd. for C_21_H_18_N_4_O_2_ [M + H]^+^: 359.1503, found: 359.1509.

*2-Methyl-7-((4-methylpyrimidin-2-ylamino)(4-(trifluoromethyl)phenyl)methyl)quinolin-8-ol* (**48**): white solid, yield: 25% (64 mg), m.p.: 123–125 °C, C_23_H_19_F_3_N_4_O; ^1^H-NMR (500 MHz, DMSO) δ 9.59 (s, 1H), 8.19–8.15 (m, 2H), 8.09 (d, *J* = 9.4 Hz, 1H), 7.69–7.64 (m, 3H), 7.59 (d, *J* = 8.3 Hz, 2H), 7.42 (d, *J* = 8.4 Hz, 1H), 7.36 (d, *J* = 8.6 Hz, 1H), 7.07 (d, *J* = 9.4 Hz, 1H), 6.52 (d, *J* = 5.0 Hz, 1H), 2.70 (s, 3H), 2.26 (s, 3H); ^13^C-NMR (126 MHz, DMSO) δ 167.96 (s), 162.04 (s), 158.23 (s), 157.51 (s), 149.14 (s), 148.73 (s), 137.80 (s), 136.56 (s), 128.09 (s), 127.52 (q, *J* = 31.6 Hz), 126.17 (s), 126.09 (s), 125.57 (q, *J* = 3.5 Hz), 124.81 (q, *J* = 271.9 Hz), 124.77 (s), 123.09 (s), 117.87 (s), 110.83 (s), 52.08 (s), 25.14 (s), 24.12 (s); HRMS (ESI) *m*/*z* calcd. for C_23_H_19_F_3_N_4_O [M + H]^+^: 425.1584, found: 425.1585.

### 3.2. Cell Culture

U251 MG cell line was a gift from Szabolcs Bellyei, the University of Pecs (originally obtained from American Type Culture Collection, Manassas, VA, USA). Cells were grown at 37 °C under 5% CO_2_ and 100% humidity in Roswell Park Memorial Institute (RPMI) medium supplemented with 10% fetal calf serum (FCS) (Sigma-Aldrich, Budapest, Hungary), and penicillin-streptomycin antibiotics.

### 3.3. End Point Cytoprotection Assay

For cytoprotection assays, 10^4^ cells were seeded into each well of 96-well cell culture plates (Costar, Corning Inc., Corning, NY, USA) in culture medium containing 10% FCS. The following day, cells were treated with an increasing concentration of test compounds in the presence of 250 μM H_2_O_2_ (Sigma-Aldrich, Budapest, Hungary). The H_2_O_2_ concentration to elicit cell injury was predetermined in a pilot experiment (data not shown) and was set to induce a decrease in viability of 65–75% after 24 h Plate-to-plate variation was monitored using a dilution series of a known cytoprotective compound that was tested on each plate. Cell viability was recorded 24 h after treatment. Resazurin reagent (Sigma-Aldrich) was dissolved in phosphate buffered saline (PBS, pH 7.4) at 0.15 mg/mL concentration, sterile filtered (0.22 µm, Merck Millipore, Budapest, Hungary), aliquoted and stored at −20 °C. Samples were treated with a final concentration of 25 µg/mL resazurin. After 2 h of incubation at 37 °C 5% CO_2_, fluorescence (530 nm excitation/580 nm emission) was recorded on a multimode microplate reader (Cytofluor4000, PerSeptive Biosystems, Foster City, CA, USA). Viability was calculated in relation to untreated control cells and blank wells containing media without cells. The presented IC_50_ values (half maximal inhibitory concentration) were determined based on dose-response curves plotted in GraphPad Prism^®^ 5.

### 3.4. Real-Time Cell Electronic Sensing (RT-CES) Cytoprotection Assay

A RT cytoprotection assay was performed as previously described [42,48]. Briefly, RT-CES 96-well E-plate (Roche, Hungary) was coated with gelatin solution (0.2% in PBS) for 20 min at 37 °C, then gelatin was washed twice with PBS solution. Growth media (50 μL) was then gently dispensed into each well of the 96-well E-plate for background readings by the RT-CES system prior to addition of 50 μL of the cell suspension containing 10^4^ U251 MG cells. Plates were kept at room temperature in a tissue culture hood for 30 min prior to insertion into the RT-CES device in the incubator to allow cells to settle. Cell growth was monitored overnight by measurements of electrical impedance every 15 min. Continuous recording of impedance in cells was reflected by cell index value. Next day, cells were co-treated with 250 μM H_2_O_2_ and test compounds. Treated and control wells were dynamically monitored over 48 h by measurements of electrical impedance every 5 min. The raw plate reads for each titration point were normalized relative to the cell index status right before treatment. Each treatment was repeated in three wells per plate during the experiments.

### 3.5. Detection of Mitochondrial Membrane Potential

Mitochondrial membrane potential was measured as described previously [49] U251 cells (6 × 10^4^) were plated in 24-well tissue culture plates (Corning Life Sciences, Corning, NY, USA) in RPMI 10% FCS and were treated in 500 µL media containing 500 µM H_2_O_2_ with or without test compounds. Untreated controls cells were supplemented with 500 µL cell culture media. After 2 h, the supernatants were harvested. Cells were washed with PBS, trypsinized, pooled with the corresponding supernatant, and centrifuged (2000 rpm, 5 min). The pellet was resuspended and incubated for 15 min in 5 µg/mL JC-1 (5,5′,6,6′-tetrachloro-1,1′,3,3′-tetraethylbenzimidazolocarbocyanine iodide, Chemodex) containing media in final volume 300 µL at 37 °C. Finally, using FL2 (585/42 nm) -FL1 (530/30 nm) channels, the red–green fluorescence intensity of 6 × 10^3^ events was acquired immediately on a FACSCalibur flow cytometer. Data were analyzed using CellQuest^TM^ software (CellQuest Pro v5.1, Becton Dickinson, Franklin Lakes, NJ, USA) gating out debris. Bar graphs show the percentage of FL1 positive cells visualized by GraphPad Prism^®^ 5. For significance analysis, a Student’s *t*-test was applied in Microsoft Excel.

### 3.6. Gene Expression Analysis

The day before measurement, U-251 MG cells were seeded in 24-well microtiter plates with 100,000 cells/well density. Cells were treated with either vehicle (solvent control; ≤0.01% DMSO) or with 8-HQ analogues: **21**, **34**, **47**, and **48** at two different concentrations: 100 nM or 300 nM. After 6 hours of incubation the medium was removed, and cells were rinsed with PBS. Cells were collected for RNA isolation in RA1 Lysis Buffer (Macherey-Nagel, Germany) supplemented with 1% beta-mercaptoethanol. Total RNA was purified with the Direct-zol kit (Zymo Research, Irvine, CA, USA) according to the manufacturer’s protocol. Total RNA was converted into complementary DNA (cDNA) using the High-Capacity cDNA Archive Kit (Applied Biosystems, Foster City, CA, USA) in a volume of 10 µL. 0.75 µL cDNA template was used (19 ng) in each PCR reaction. Quantitative real-time PCR was performed on the LightCycler 96 instrument (Roche) with gene-specific primers and the SYBRGreen protocol. NormFinder software [50] was used to calculate the most suitable housekeeping genes (TUBB and PPIA) for relative expression ratio calculation. For significance analysis, a Student’s *t*-test was applied in Microsoft Excel. Table 4 lists the primer sequences used.

## 4. Conclusions

Our synthesized 8HQ analogs were tested for cytoprotective activity in different assays and a handful of compounds were selected for further test. As expected, maintenance of mitochondrial membrane potential in oxidative stress was a key factor for their activity. Induction of hypoxia related genes under the regulation of HIF1A may be a crucial mechanistic step for cytoprotective activity. Based on our results, lead compounds will be further tested in animal models of different CNS diseases where oxidative stress is involved.

## 5. Patents

Compounds described in the current study are protected by patent #WO2011148208.

## Figures and Tables

**Table 1:**
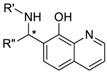
Betti-three components reactions (3CR) results, use of several anilines and aldehydes (Subclass 1).

Compounds	Amine (R′)	Aldehyde (R″)	Yield (%) ^a^	IC_50_ (µM)	SD
**16**	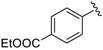	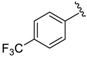	30	0.195	0.143
**17**	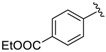	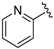	60	0.122	0.029
**18**	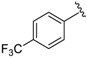	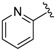	33	0.096	0.033
**19**	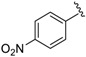	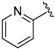	14	0.125	0.024

^a^ after column chromatography and crystallization.

**Table 2:**
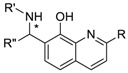
Functionalization with several picolines (Subclass 2).

Compounds	Amine (R′)	Aldehyde (R″)	R	Yield (%) ^a^	IC_50_ (µM)	SD
**20**	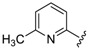	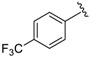	H	33	0.087	0.028
**21**	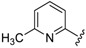	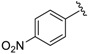	H	51	0.086	0.046
**22**	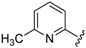	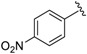	Me	23	0.562	0.136
**23**	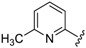	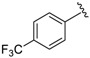	Me	71	0.824	0.390
**24**	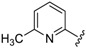	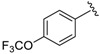	H	34	0.135	0.073
**25**	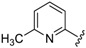	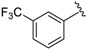	H	62	0.156	0.052
**26**	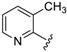	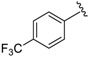	H	18	0.166	0.040
**27**	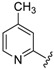	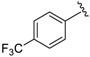	H	15	0.156	0.078
**28**	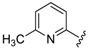	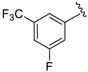	H	35	0.206	0.168
**29**	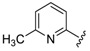	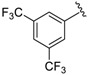	H	43	0.379	0.217
**30**	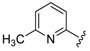	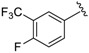	H	90	0.246	0.171
**31**	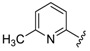	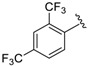	H	22	0.460	0.199
**32**	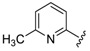	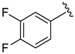	H	53	0.204	0.111

^a^ after column chromatography and crystallization.

**Table 3:**
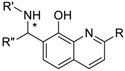
Functionalization with several pyrimidines (Subclass 3).

Compounds	Amine (R′)	Aldehyde (R″)	R	Yield (%) ^a^	IC_50_ (µM)	SD
**33**	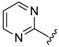	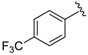	H	32	0.138	0.077
**34**	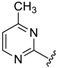	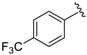	H	46	0.119	0.080
**35**	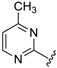	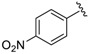	H	19	0.114	0.095
**36**	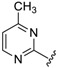	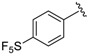	H	68	0.140	0.152
**37**	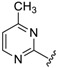	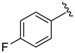	H	16	0.163	0.109
**38**	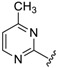	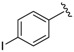	H	18	0.149	0.067
**39**	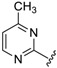	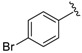	H	29	0.135	0.098
**40**	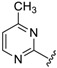	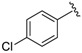	H	40	0.073	0.021
**41**	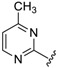	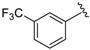	H	31	0.169	0.129
**42**	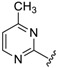	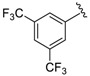	H	59	0.413	0.240
**43**	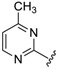	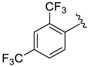	H	16	0.119	0.050
**44**	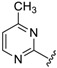	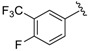	H	25	0.139	0.064
**45**	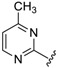	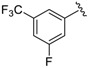	H	15	0.313	0.067
**46**	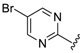	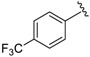	H	15	0.183	0.157
**47**	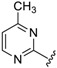	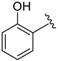	H	15	0.481	0.082
**48**	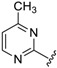	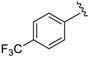	Me	25	0.887	0.226

^a^ after column chromatography and crystallization.

**Table 4:** Primer sequences for Quantitative real-time PCR (qRT-PCR) analysis.

Gene Name	Abbreviation	Forward_Sequence	Reverse_Sequence
Tubulin beta class I	TUBB	ataccttgaggcgagcaaaa	ctgatcacctcccagaacttg
Peptidylprolyl isomerase A	PPIA	atgctggacccaacacaaat	tctttcactttgccaaacacc
Heme oxygenase 1	HMOX1	ggcagagggtgatagaagagg	agctcctgcaactcctcaaa
Vascular endothelial growth factor	VEGF	gcagcttgagttaaacgaacg	ggttcccgaaaccctgag
Solute carrier family 2 member 1	GLUT1	ccccatcccatggttcatc	tgaggtccagttggagaagc

## Supplementary Materials

The following are available online (^1^H, ^13^C-NMR and HRMS spectra of **1**–**48**).
