# Supplementary material for: Synthesis and Cytoprotective Characterization of 8-Hydroxyquinoline Betti Products
Source: Molecules. 2018 Aug 2;23(8):1934. doi: 10.3390/molecules23081934 (PMC6222637; doi:10.3390/molecules23081934)
Supplement: Supplementary file 1 [file molecules-23-01934-s001.pdf]

molecules MDPI

Article

Synthesis and Cytoprotective Characterization of 8-Hydroxyquinoline Betti Products

Iván Kanizsai [1,*], Ramóna Madácsi [1], László Hackler Jr.[1], Márió Gyuris [1], Gábor J. Szebeni [1], Orsolya Huzián [2] and László G. Puskás [1,2,*]

[1] Avidin Ltd., Alsó kikötő sor. 11D, H-6726 Szeged, Hungary; r.madacsi@avicorbiotech.com (R.M.); hackler@avidinbiotech.com (L.H., Jr.); gyuris.mario@gmail.com (M.G.); g.szebeni@avidinbiotech.com (G.J.S.)
[2] Avicor Ltd., Alsó kikötő sor. 11D, H-6726 Szeged, Hungary; o.huzian@avicorbiotech.com
* Correspondence: i.kanizsai@avidinbiotech.com (I.K.); laszlo@avidinbiotech.com (L.G.P)

I. NMR spectra:

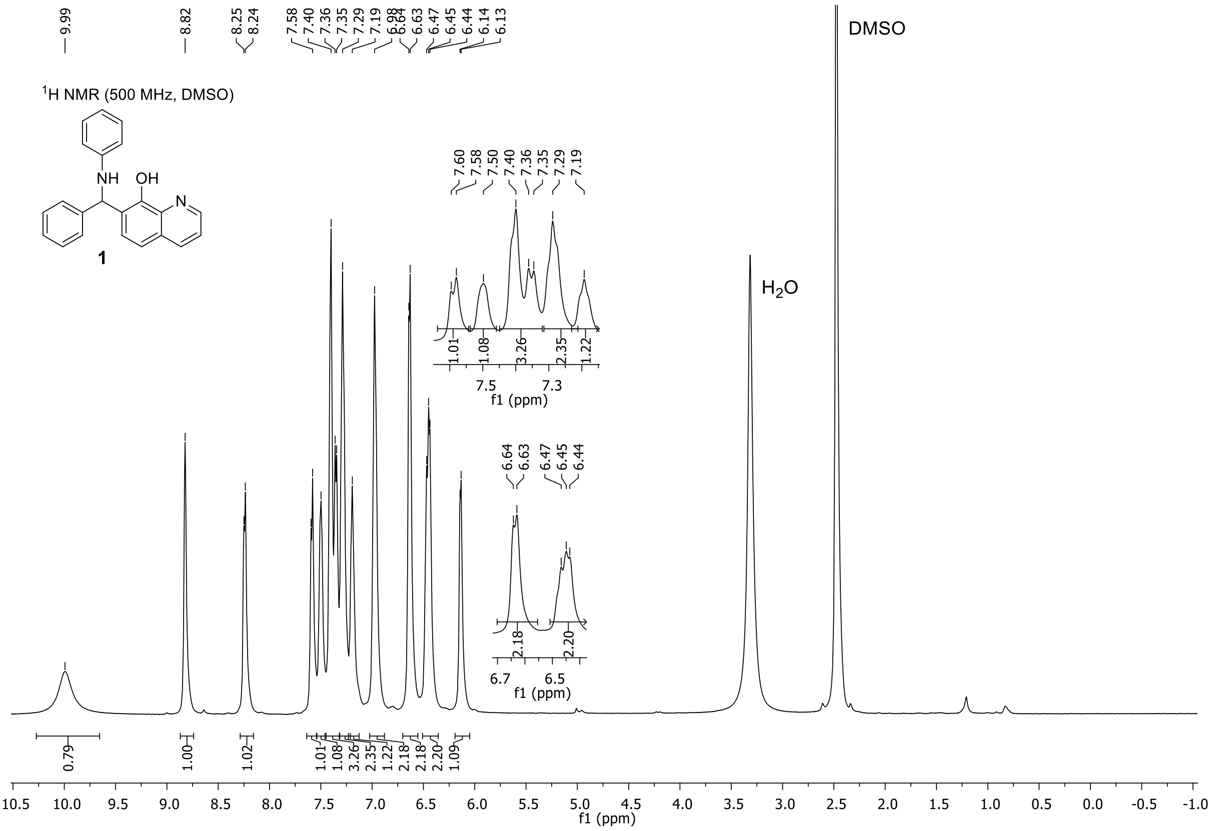

DMSO

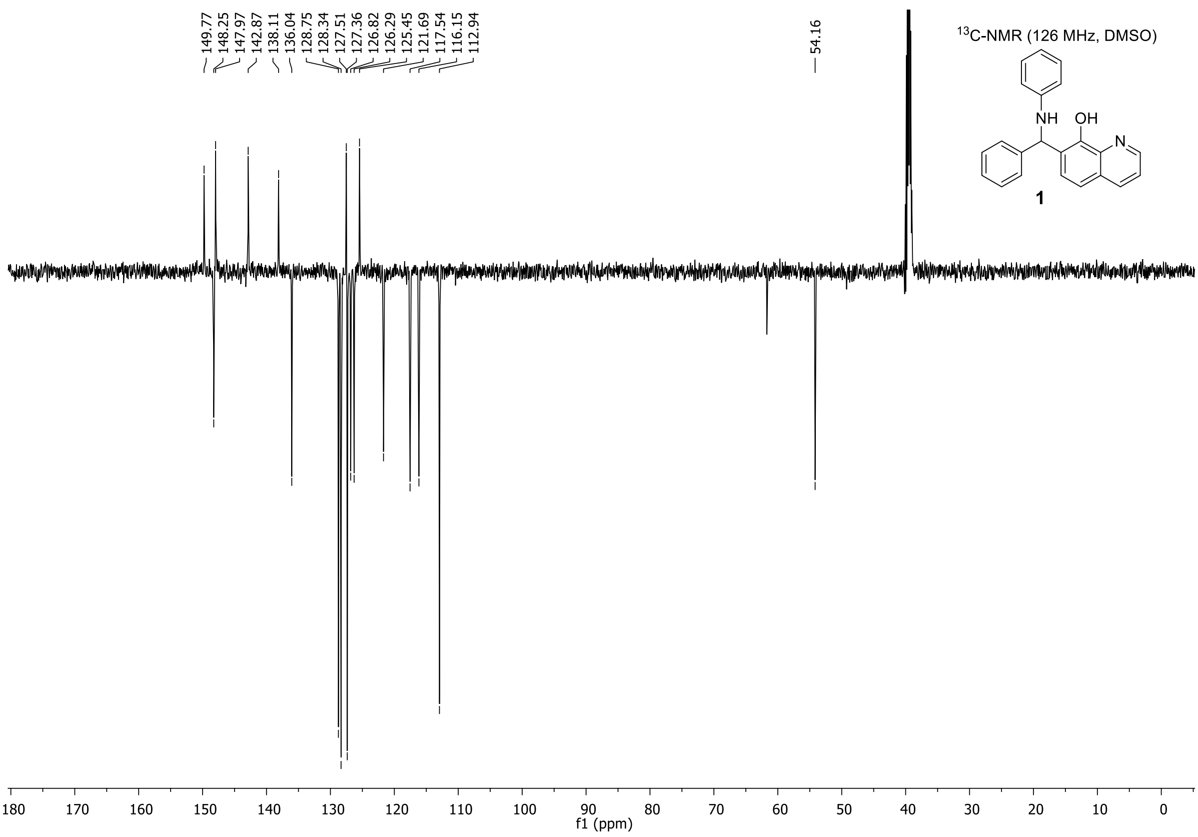

149.77
148.25
147.97
142.87
138.11
136.04
128.75
128.34
127.51
127.36
126.82
126.29
125.45
121.69
117.54
116.15
112.94
54.16
^{13}C-NMR (126 MHz, DMSO)
NH
OH
N
1
180 170 160 150 140 130 120 110 100 90 80 70 60 50 40 30 20 10 0
f1 (ppm)

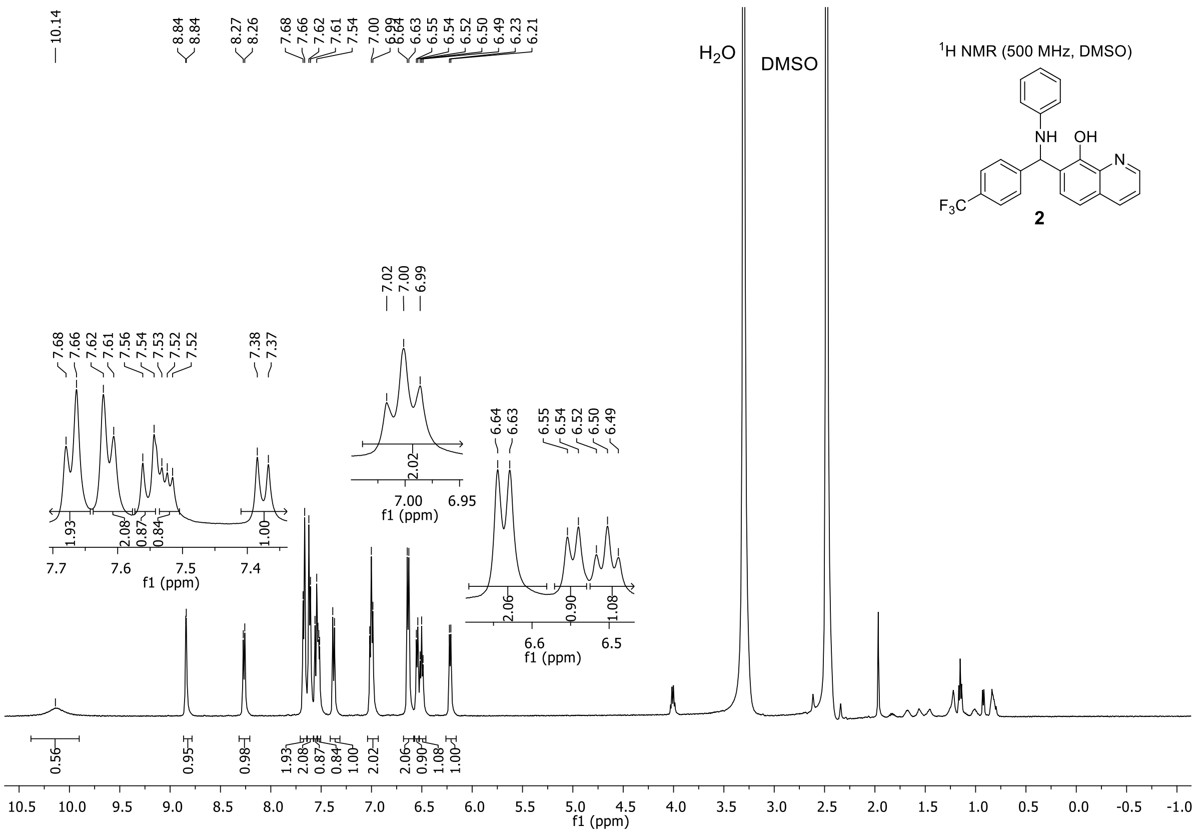

10.14
8.84
8.84
8.27
8.26
7.68
7.66
7.62
7.61
7.54
7.00
6.99
6.64
6.63
6.55
6.54
6.52
6.50
6.49
6.23
6.21
H_2O
DMSO
^{1}H NMR (500 MHz, DMSO)
NH
OH
N
F_3C
2
7.02
7.00
6.99
7.68
7.66
7.62
7.61
7.56
7.54
7.53
7.52
7.52
7.38
7.37
1.93
2.08
0.87
0.84
1.00
7.7 7.6 7.5 7.4
f1 (ppm)
2.02
7.00 6.95
f1 (ppm)
6.64
6.63
6.55
6.54
6.52
6.50
6.49
2.06
0.90
1.08
6.6 6.5
f1 (ppm)
0.56
0.95
0.98
1.93
2.08
0.87
0.84
1.00
2.02
2.06
0.90
1.08
1.00
10.5 10.0 9.5 9.0 8.5 8.0 7.5 7.0 6.5 6.0 5.5 5.0 4.5 4.0 3.5 3.0 2.5 2.0 1.5 1.0 0.5 0.0 -0.5 -1.0
f1 (ppm)

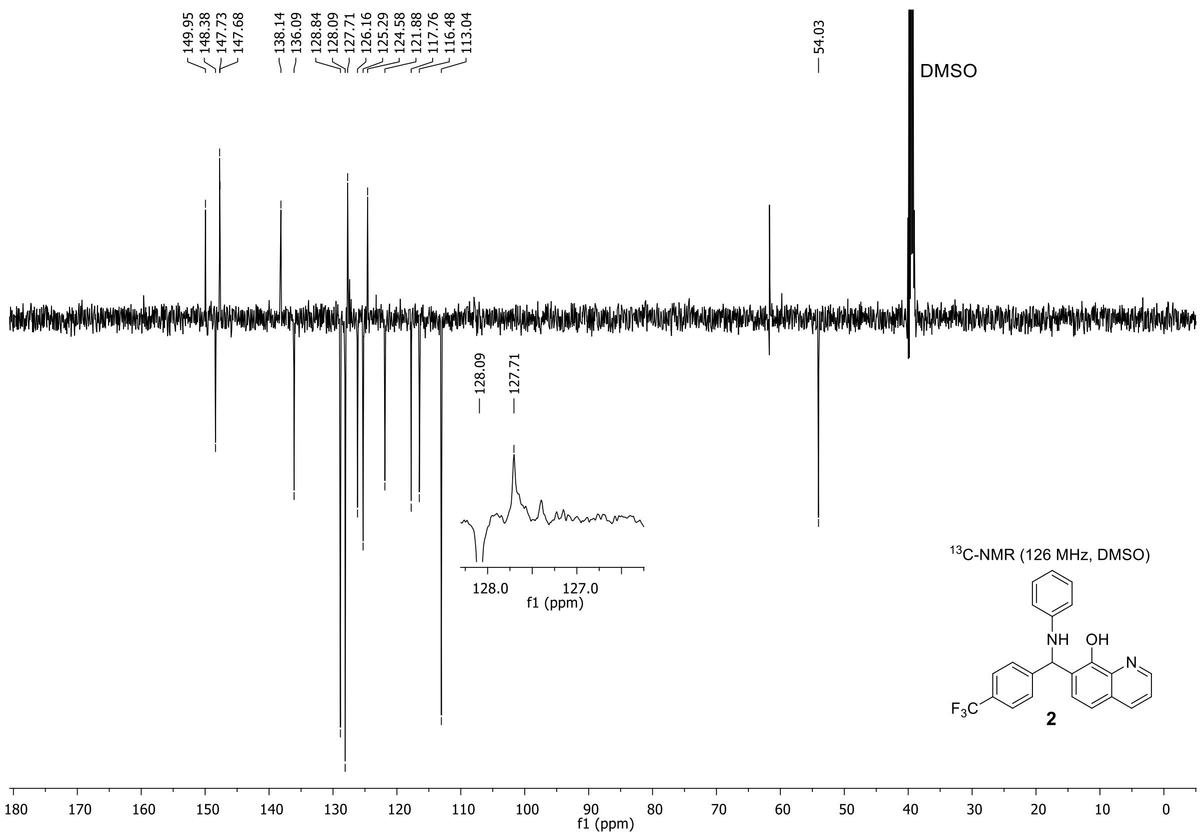
149.95
148.38
147.73
147.68
138.14
136.09
128.84
128.09
127.71
126.16
125.29
124.58
121.88
117.76
116.48
113.04
54.03
DMSO
128.09
127.71
128.0
127.0
f1 (ppm)
13C-NMR (126 MHz, DMSO)
NH OH
N
F3C
2
180 170 160 150 140 130 120 110 100 90 80 70 60 50 40 30 20 10 0
f1 (ppm)

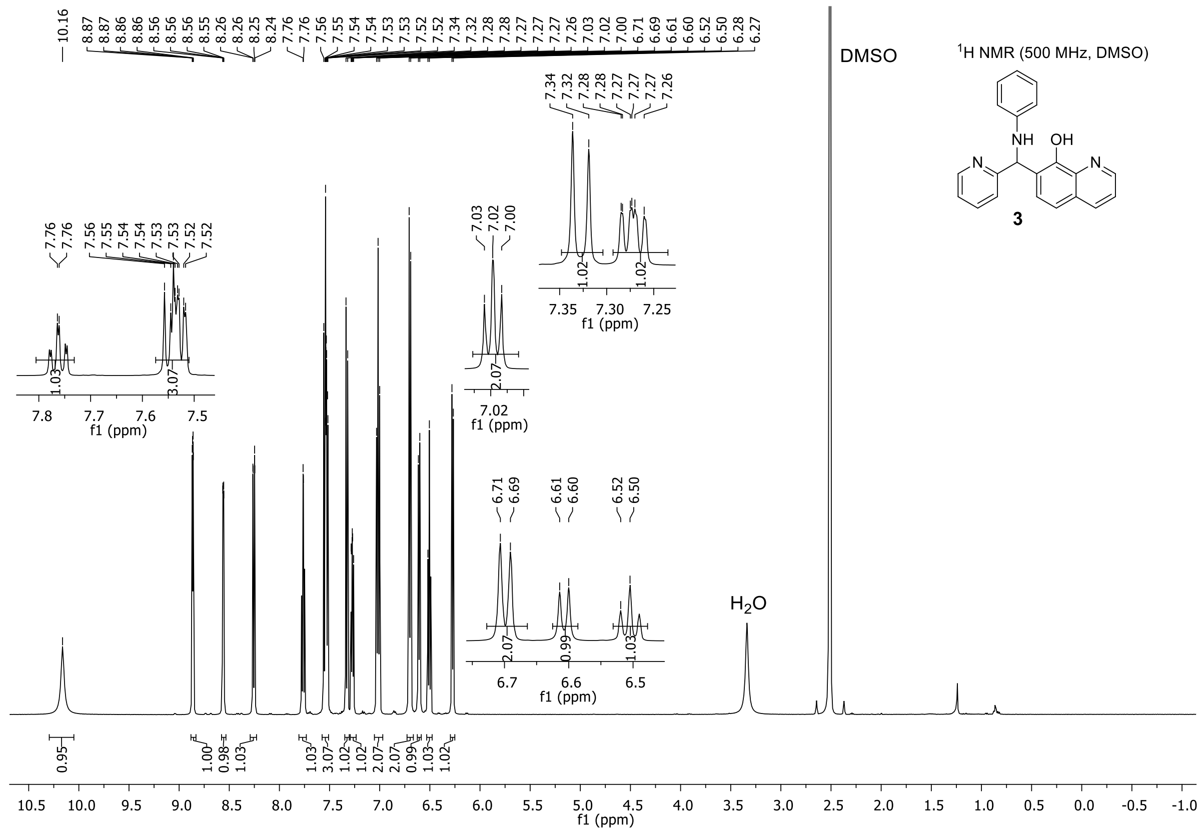
10.16
DMSO
1H NMR (500 MHz, DMSO)
NH OH
N
N
3
H2O
7.8 7.7 7.6 7.5
f1 (ppm)
7.02
f1 (ppm)
7.35 7.30 7.25
f1 (ppm)
6.7 6.6 6.5
f1 (ppm)
10.5 10.0 9.5 9.0 8.5 8.0 7.5 7.0 6.5 6.0 5.5 5.0 4.5 4.0 3.5 3.0 2.5 2.0 1.5 1.0 0.5 0.0 -0.5 -1.0
f1 (ppm)

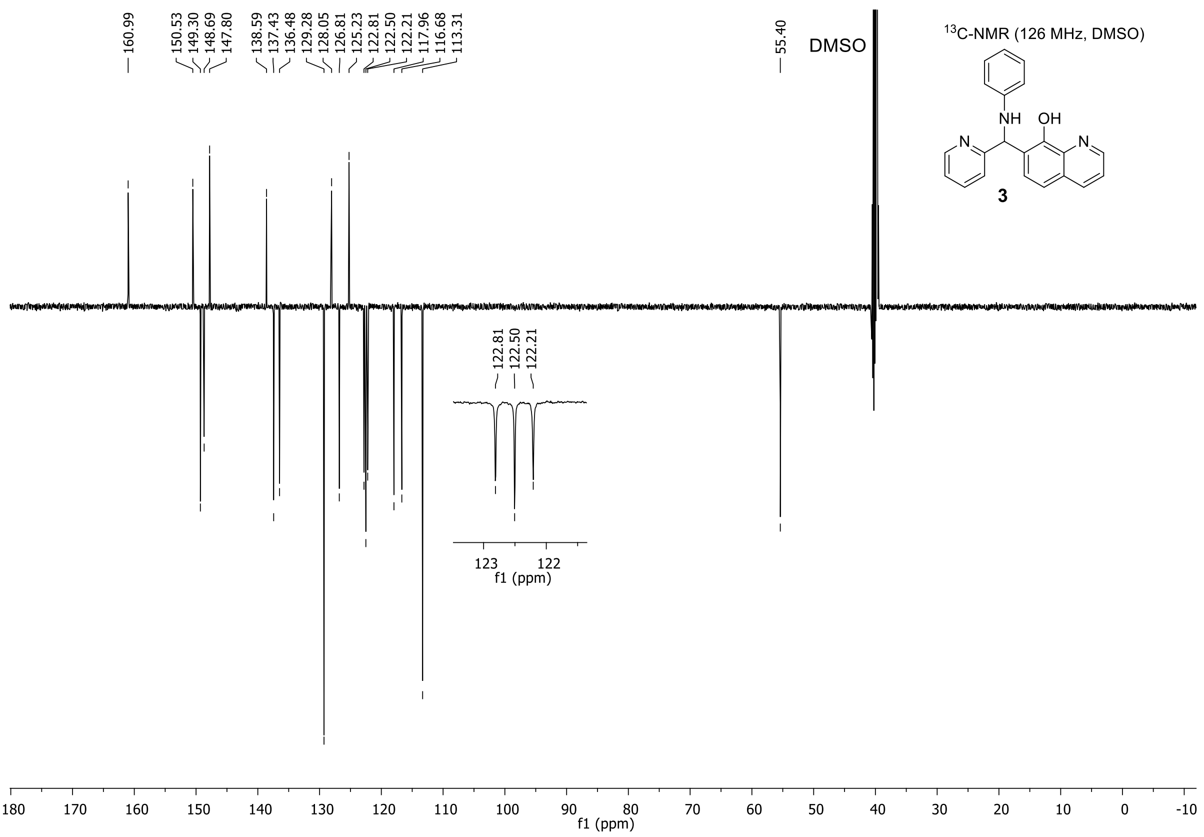

160.99
150.53
149.30
148.69
147.80
138.59
137.43
136.48
129.28
128.05
126.81
125.23
122.81
122.50
122.21
117.96
116.68
113.31
55.40
DMSO
^{13}C-NMR (126 MHz, DMSO)
NH OH
N
N
3
122.81
122.50
122.21
123 122
f1 (ppm)
180 170 160 150 140 130 120 110 100 90 80 70 60 50 40 30 20 10 0 -10
f1 (ppm)

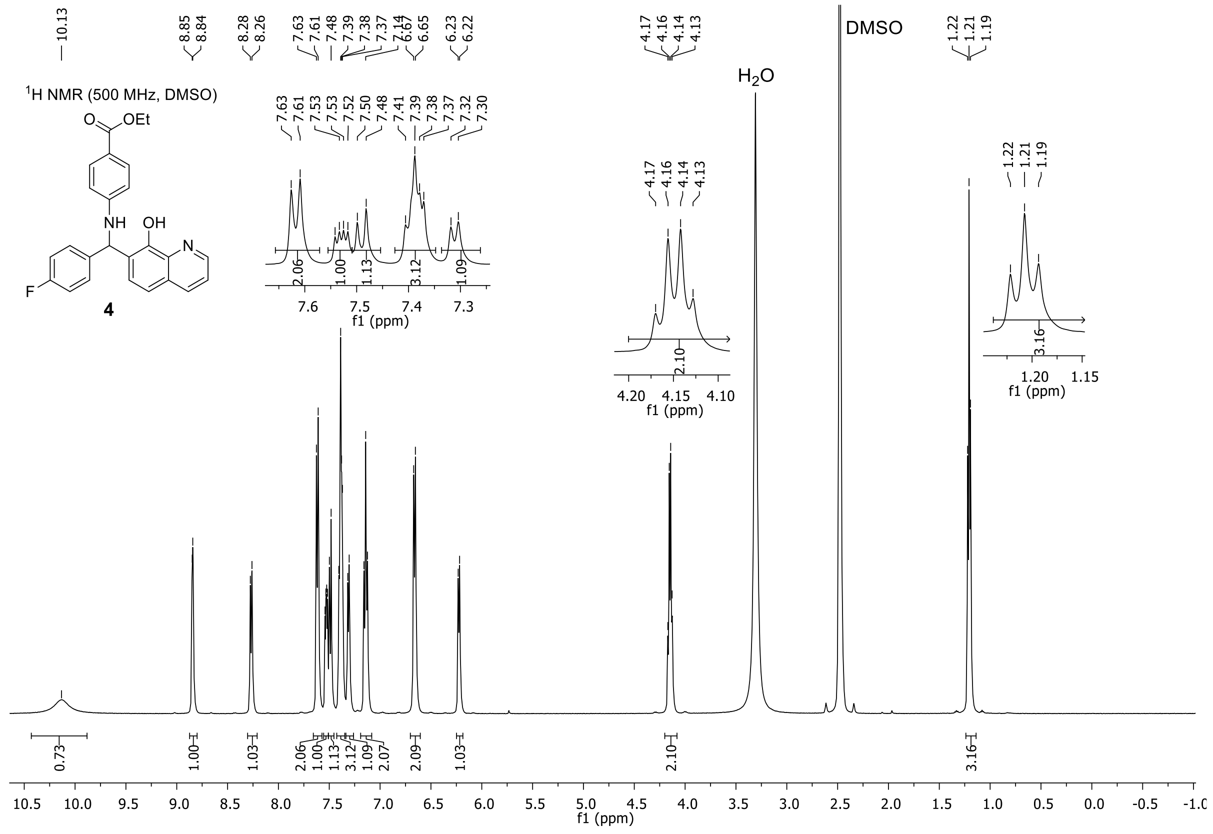

10.13
8.85
8.84
8.28
8.26
7.63
7.61
7.48
7.39
7.38
7.37
7.14
6.67
6.65
6.23
6.22
4.17
4.16
4.14
4.13
DMSO
1.22
1.21
1.19
H_2O
^{1}H NMR (500 MHz, DMSO)
O OEt
NH OH
N
F
4
7.63
7.61
7.53
7.53
7.52
7.50
7.48
7.41
7.39
7.38
7.37
7.32
7.30
2.06
1.00
1.13
3.12
1.09
7.6 7.5 7.4 7.3
f1 (ppm)
4.17
4.16
4.14
4.13
2.10
4.20 4.15 4.10
f1 (ppm)
1.22
1.21
1.19
3.16
1.20 1.15
f1 (ppm)
0.73
1.00
1.03
2.06
1.00
1.13
3.12
1.09
2.07
2.09
1.03
2.10
3.16
10.5 10.0 9.5 9.0 8.5 8.0 7.5 7.0 6.5 6.0 5.5 5.0 4.5 4.0 3.5 3.0 2.5 2.0 1.5 1.0 0.5 0.0 -0.5 -1.0
f1 (ppm)

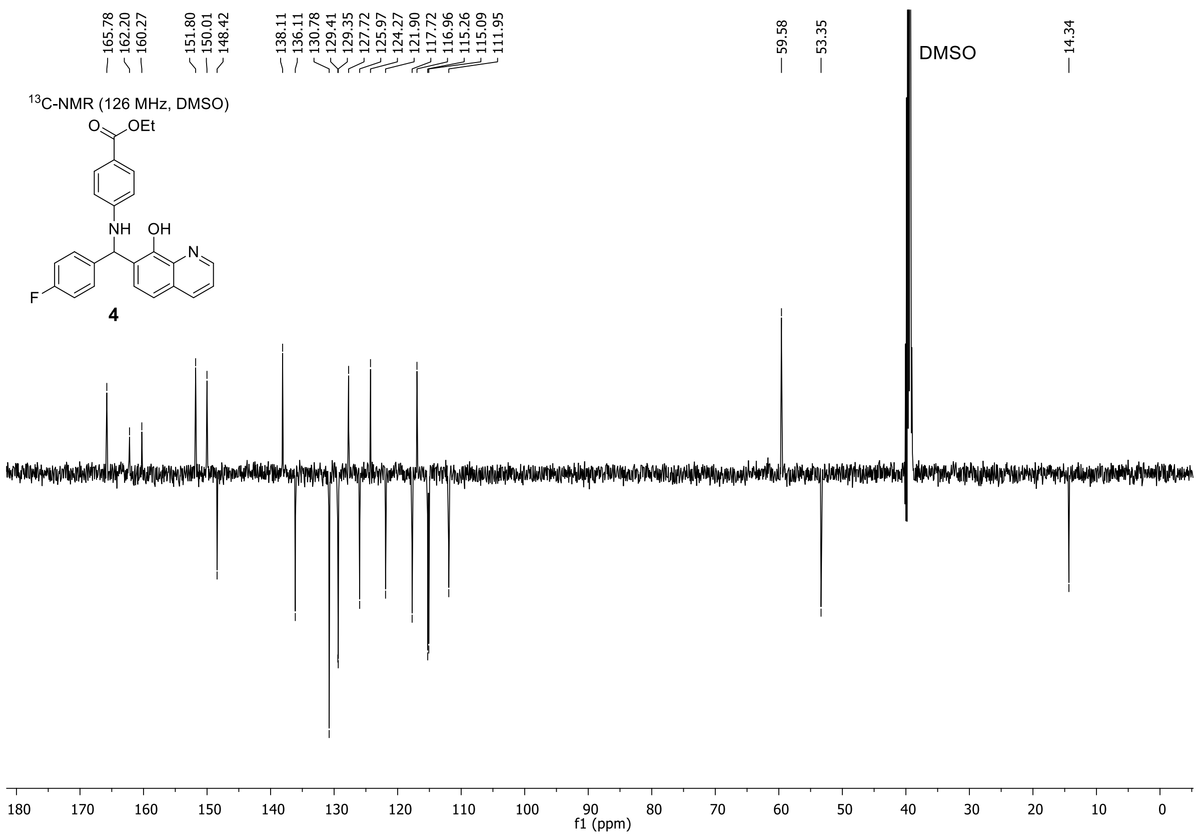

165.78
162.20
160.27
151.80
150.01
148.42
138.11
136.11
130.78
129.41
129.35
127.72
125.97
124.27
121.90
117.72
116.96
115.26
115.09
111.95
59.58
53.35
DMSO
14.34
13C-NMR (126 MHz, DMSO)
O
OEt
NH
OH
N
F
4
f1 (ppm)

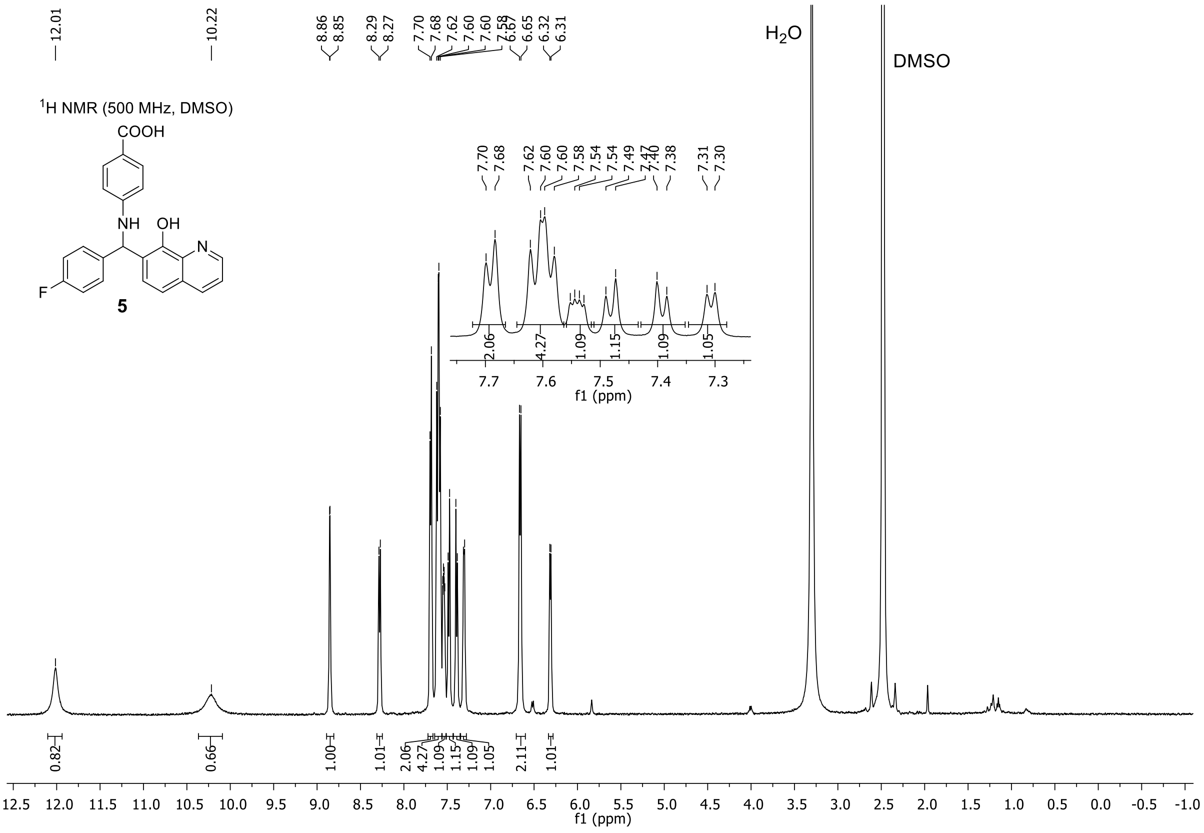

12.01
10.22
8.86
8.85
8.29
8.27
7.70
7.68
7.62
7.60
6.65
6.32
6.31
H2O
DMSO
1H NMR (500 MHz, DMSO)
COOH
NH
OH
N
F
5
7.70
7.68
7.62
7.60
7.60
7.58
7.54
7.49
7.47
7.40
7.38
7.31
7.30
2.06
4.27
1.09
1.15
1.09
1.05
f1 (ppm)
0.82
0.66
1.00
1.01
2.11
1.01
f1 (ppm)

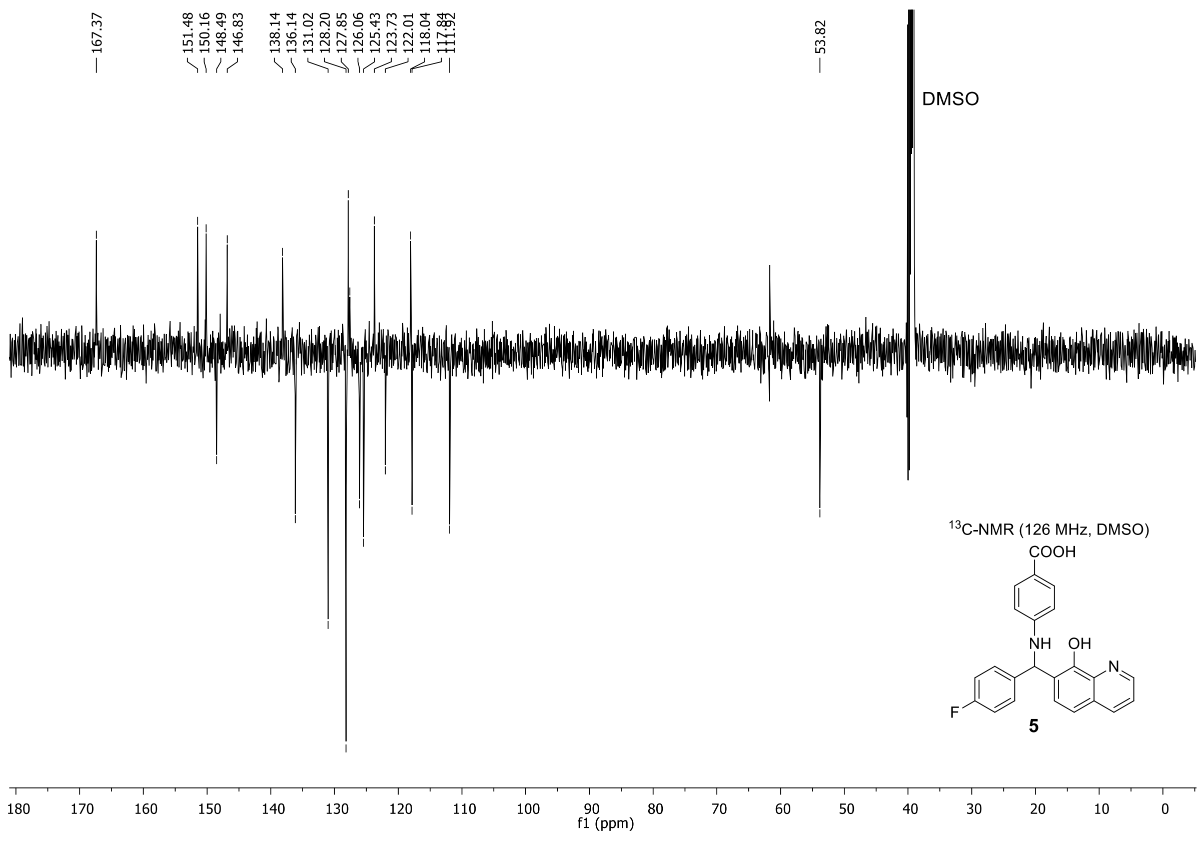
167.37
151.48
150.16
148.49
146.83
138.14
136.14
131.02
128.20
127.85
126.06
125.43
123.73
122.01
118.04
117.84
111.92
53.82
DMSO
^{13}C-NMR (126 MHz, DMSO)
COOH
NH
OH
N
F
5
f1 (ppm)

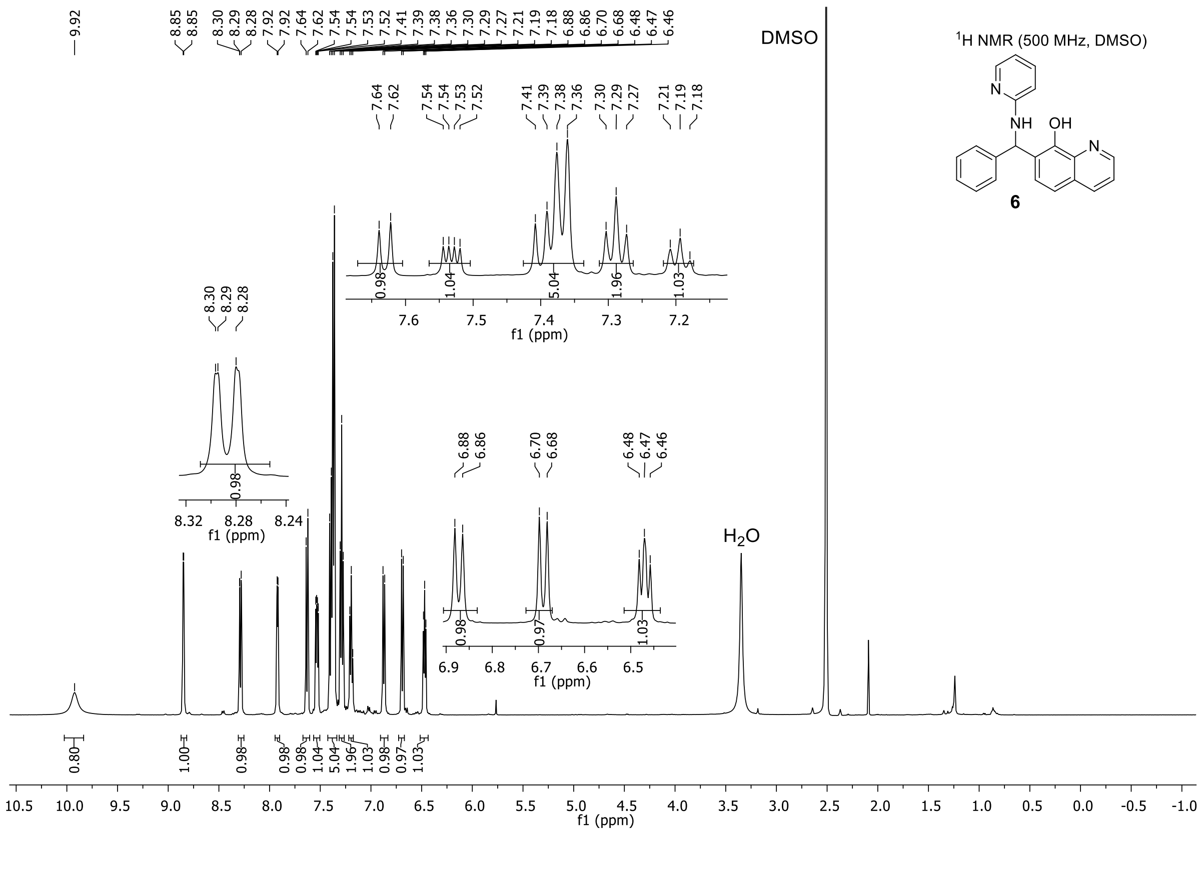
9.92
8.85
8.85
8.30
8.29
8.28
7.92
7.92
7.64
7.62
7.54
7.54
7.53
7.52
7.41
7.39
7.38
7.36
7.30
7.29
7.27
7.21
7.19
7.18
6.88
6.86
6.70
6.68
6.48
6.47
6.46
DMSO
^{1}H NMR (500 MHz, DMSO)
N
NH
OH
N
6
H$_2$O
f1 (ppm)

S6

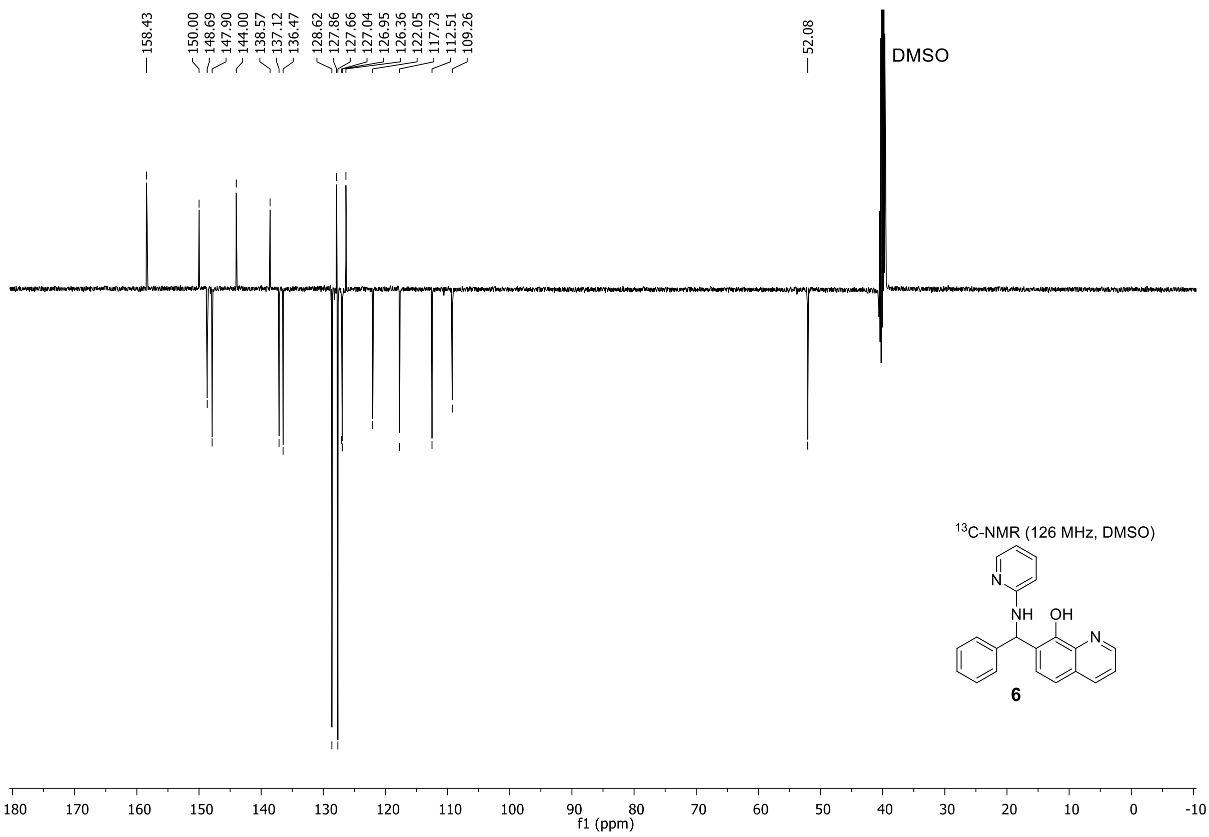

158.43
150.00
148.69
147.90
144.00
138.57
137.12
136.47
128.62
127.86
127.66
127.04
126.95
126.36
122.05
117.73
112.51
109.26
52.08
DMSO
13C-NMR (126 MHz, DMSO)
N
NH OH
N
6
f1 (ppm)

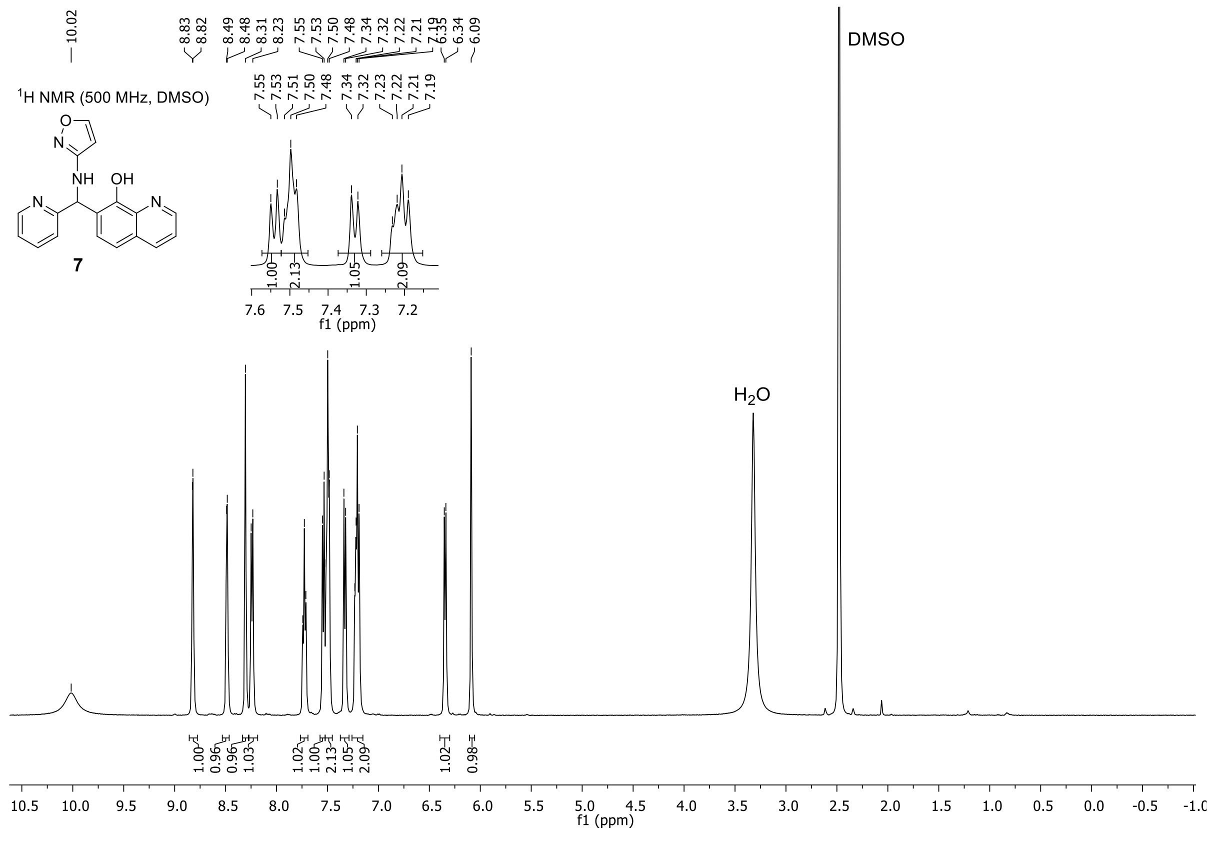

10.02
8.83
8.82
8.49
8.48
8.31
8.23
7.55
7.53
7.50
7.48
7.34
7.32
7.22
7.21
7.19
6.35
6.34
6.09
DMSO
1H NMR (500 MHz, DMSO)
O
N
NH OH
N
N
7
H2O
f1 (ppm)

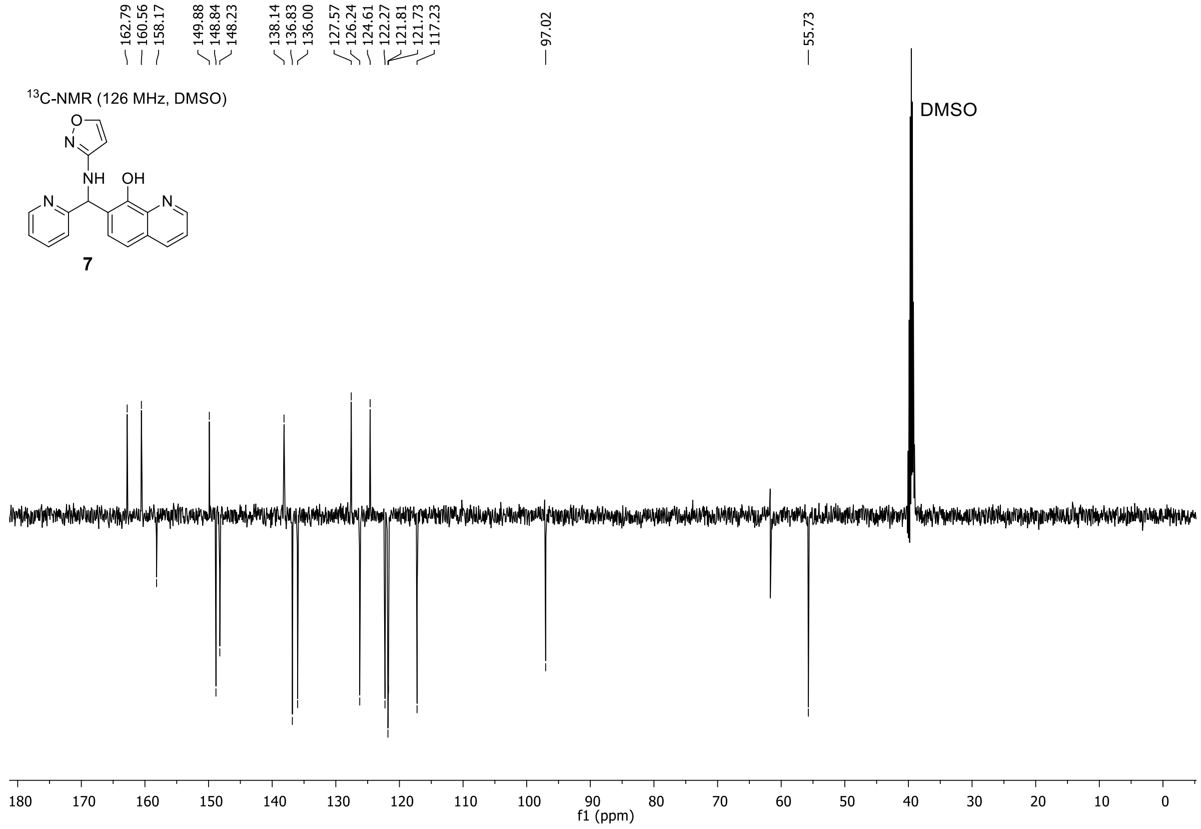
162.79
160.56
158.17
149.88
148.84
148.23
138.14
136.83
136.00
127.57
126.24
124.61
122.27
121.81
121.73
117.23
97.02
55.73
13C-NMR (126 MHz, DMSO)
DMSO
O
N
NH
OH
N
N
7
180 170 160 150 140 130 120 110 100 90 80 70 60 50 40 30 20 10 0
f1 (ppm)

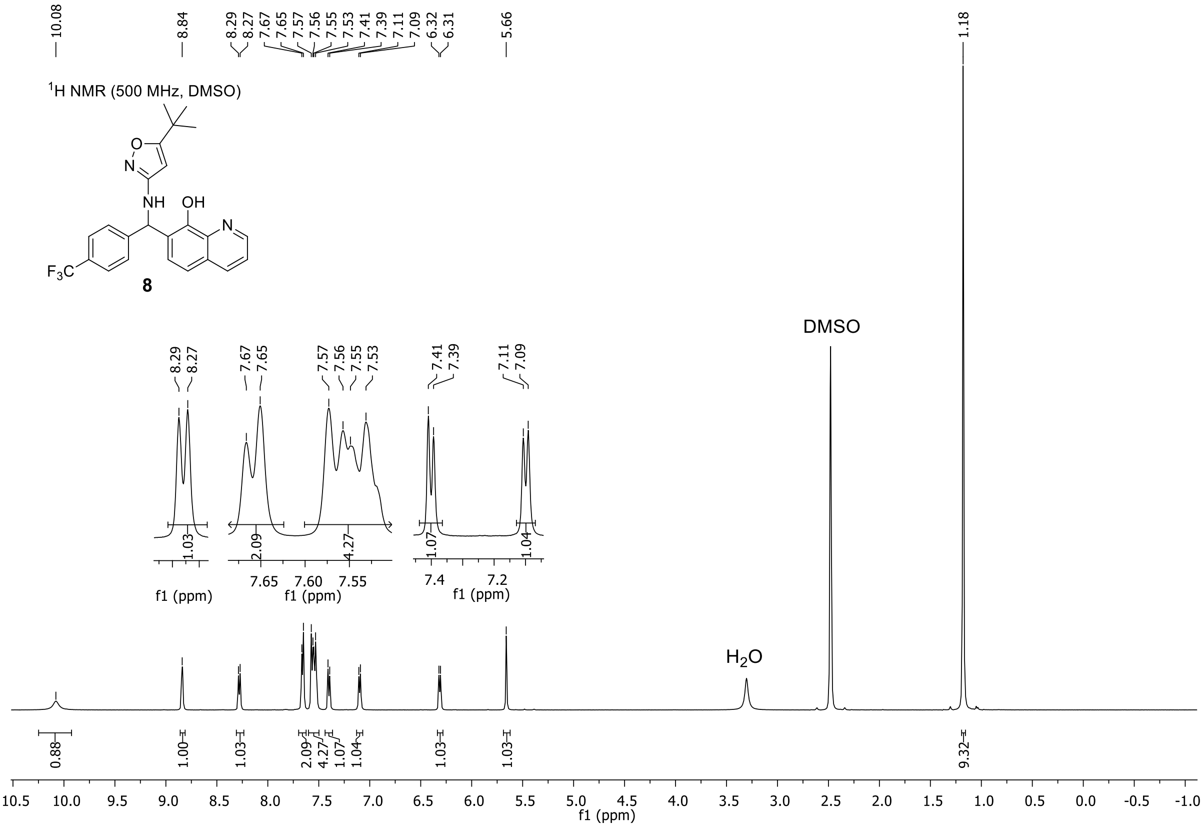
10.08
8.84
8.29
8.27
7.67
7.65
7.57
7.56
7.55
7.53
7.41
7.39
7.11
7.09
6.32
6.31
5.66
1.18
1H NMR (500 MHz, DMSO)
O
N
NH
OH
N
F3C
8
DMSO
H2O
1.03
2.09
4.27
1.07
1.04
7.65 7.60 7.55
7.4 7.2
f1 (ppm)
0.88
1.00
1.03
2.09
4.27
1.07
1.04
1.03
1.03
9.32
10.5 10.0 9.5 9.0 8.5 8.0 7.5 7.0 6.5 6.0 5.5 5.0 4.5 4.0 3.5 3.0 2.5 2.0 1.5 1.0 0.5 0.0 -0.5 -1.0
f1 (ppm)

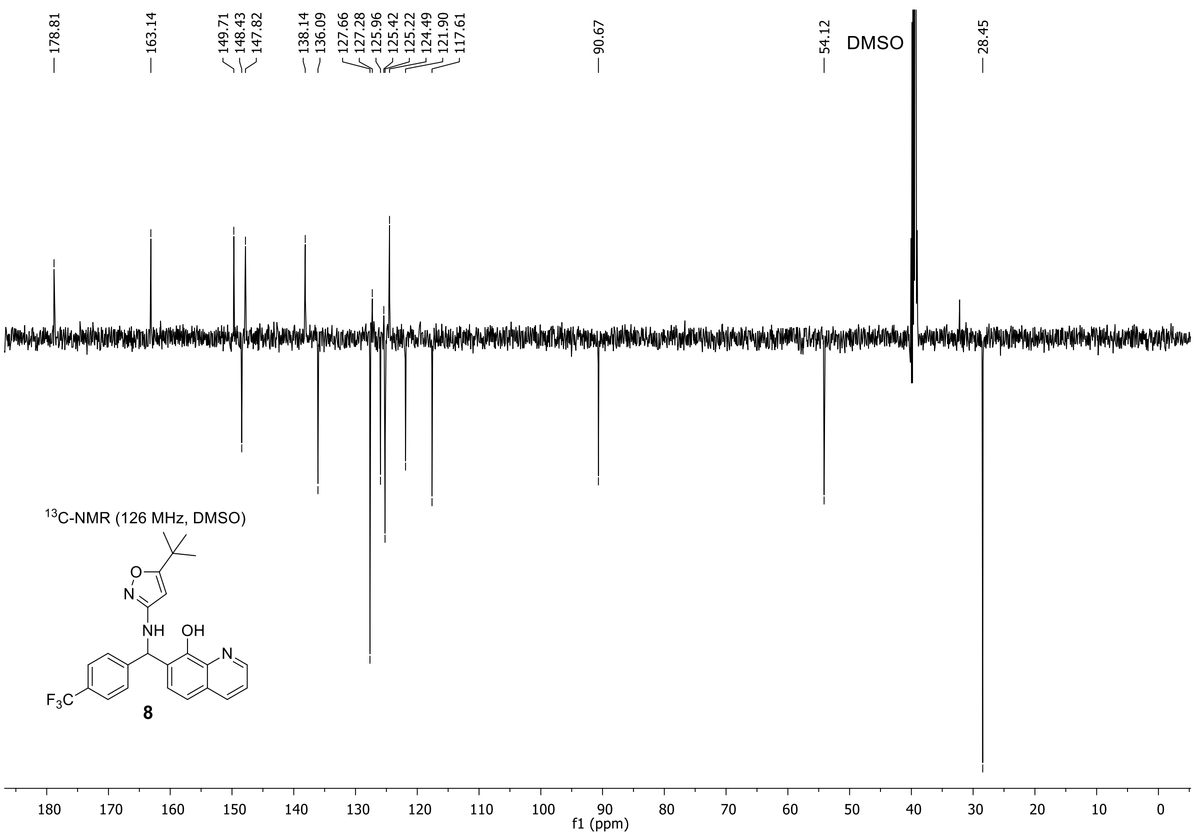

178.81
163.14
149.71
148.43
147.82
138.14
136.09
127.66
127.28
125.96
125.42
125.22
124.49
121.90
117.61
90.67
54.12
DMSO
28.45
13C-NMR (126 MHz, DMSO)
NH
OH
N
O
F3C
8
f1 (ppm)

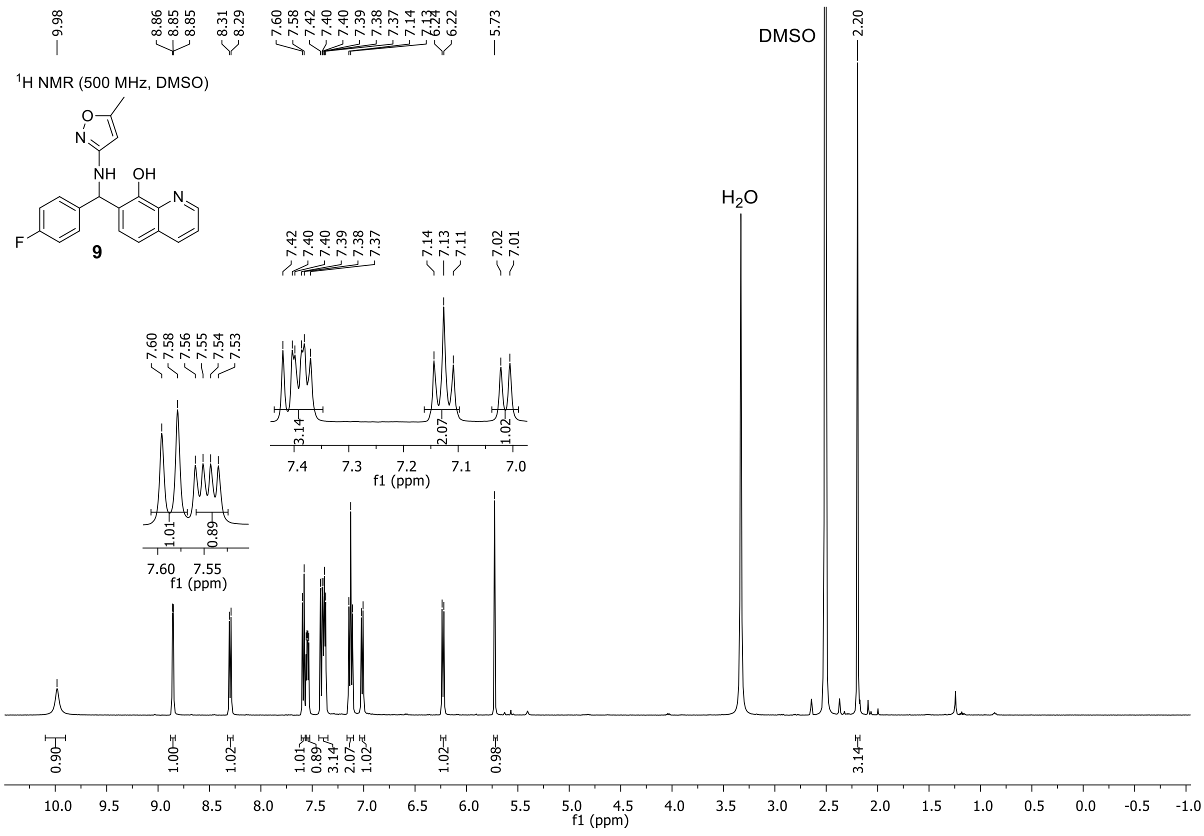

9.98
8.86
8.85
8.85
8.31
8.29
7.60
7.58
7.42
7.40
7.40
7.39
7.38
7.37
7.14
7.13
6.24
6.22
5.73
DMSO
2.20
1H NMR (500 MHz, DMSO)
NH
OH
F
9
H2O
f1 (ppm)
0.90
1.00
1.02
1.01
0.89
3.14
2.07
1.02
1.02
0.98
3.14

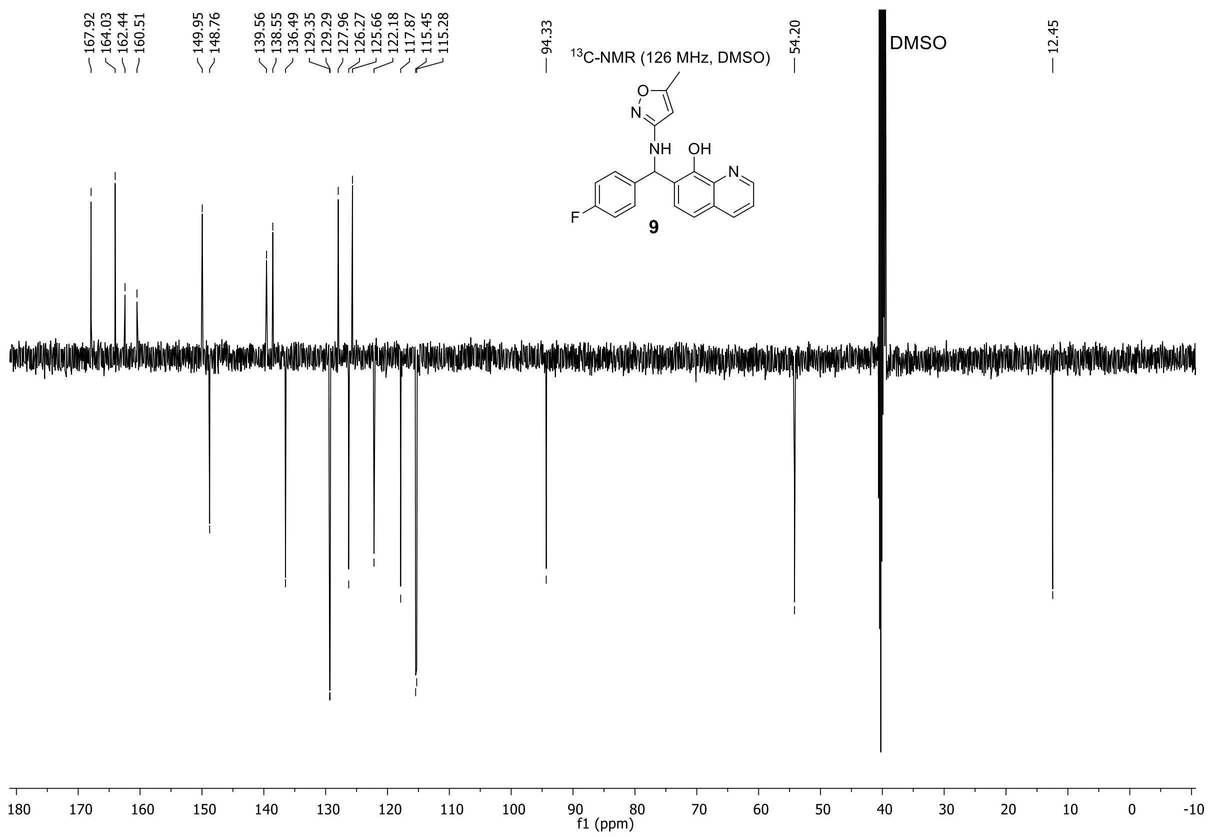
167.92
164.03
162.44
160.51
149.95
148.76
139.56
138.55
136.49
129.35
129.29
127.96
126.27
125.66
122.18
117.87
115.45
115.28
94.33
^{13}C-NMR (126 MHz, DMSO)
54.20
DMSO
12.45
9
f1 (ppm)

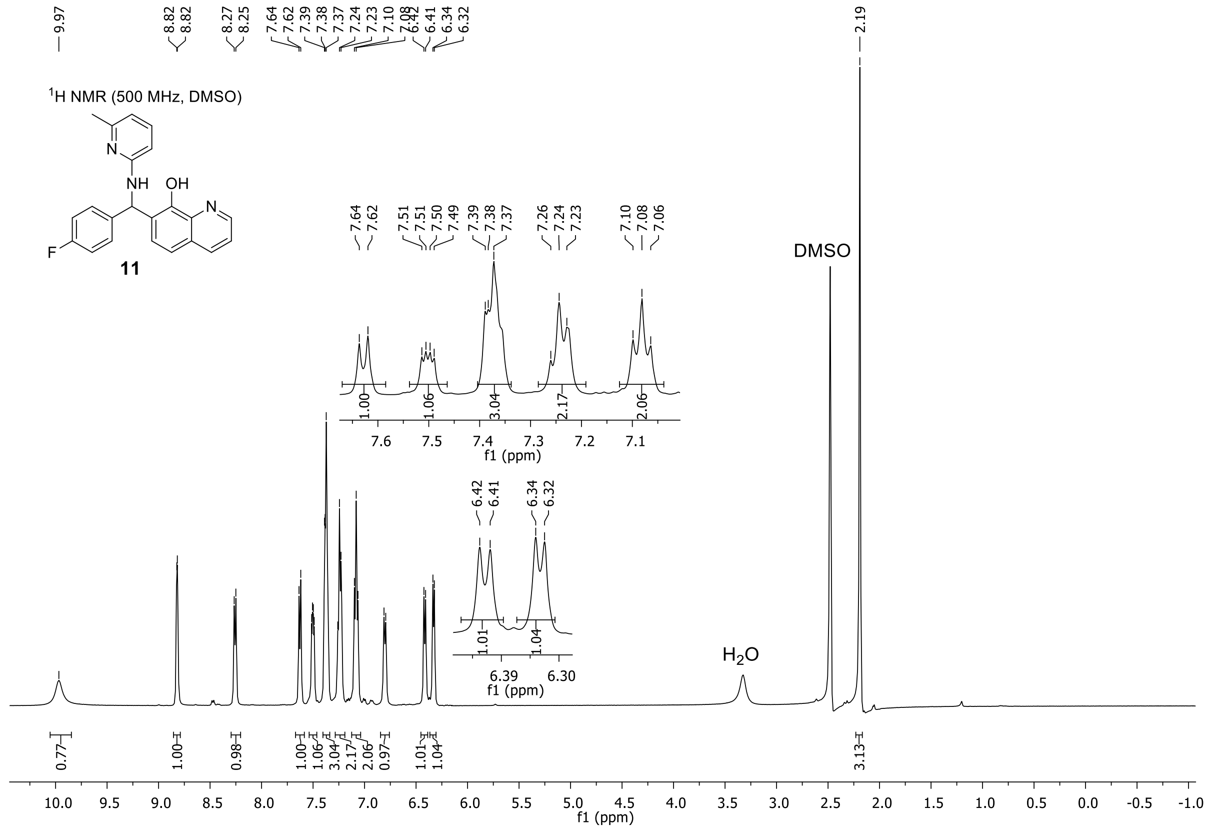
9.97
8.82
8.82
8.27
8.25
7.64
7.62
7.39
7.38
7.37
7.24
7.23
7.10
7.08
6.42
6.41
6.34
6.32
2.19
^{1}H NMR (500 MHz, DMSO)
11
DMSO
H_2O
f1 (ppm)

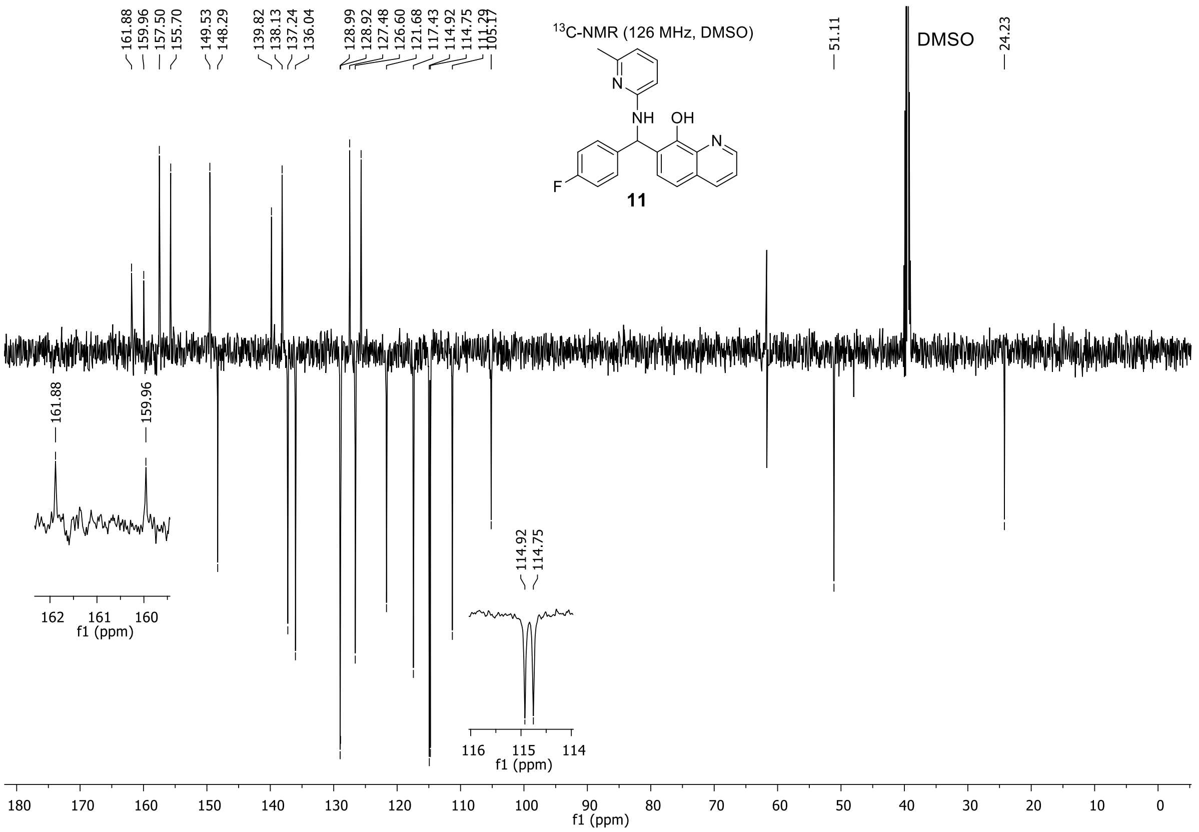
13C-NMR (126 MHz, DMSO)
DMSO
11
f1 (ppm)

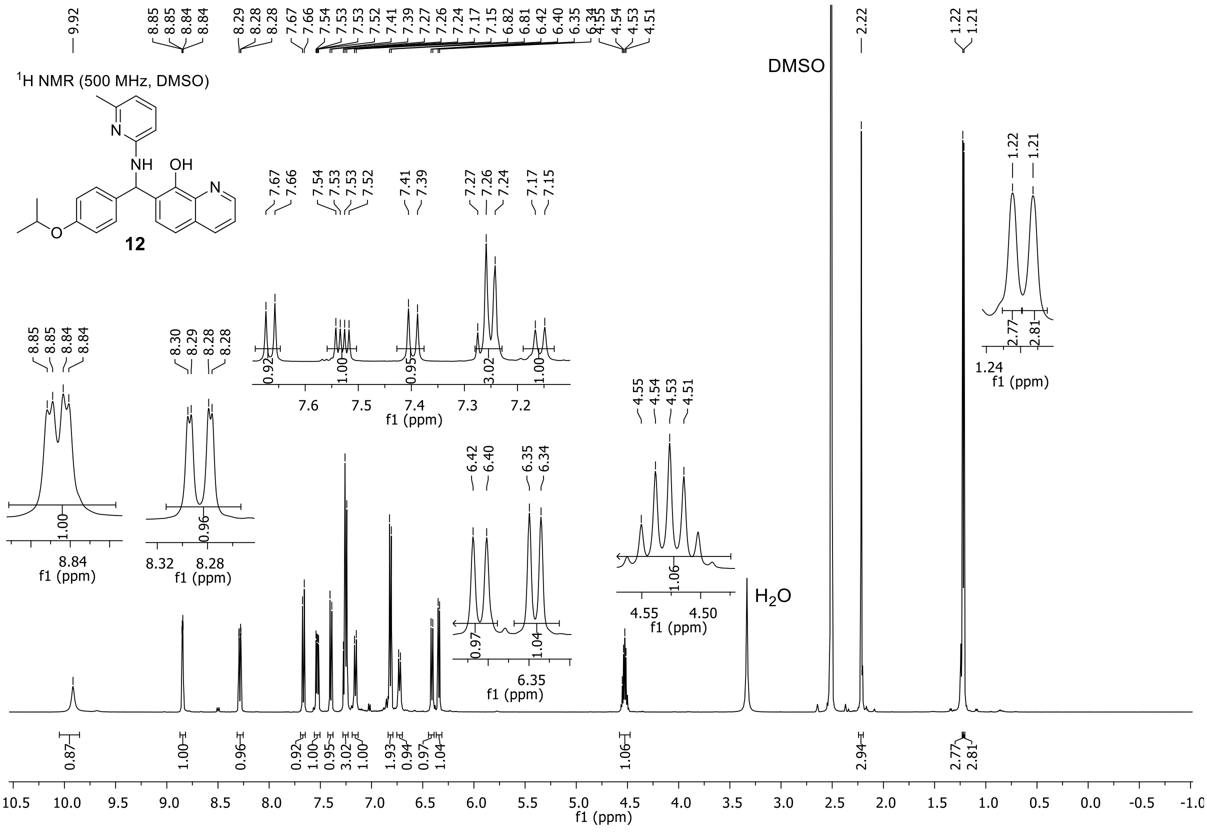
1H NMR (500 MHz, DMSO)
DMSO
H2O
12
f1 (ppm)

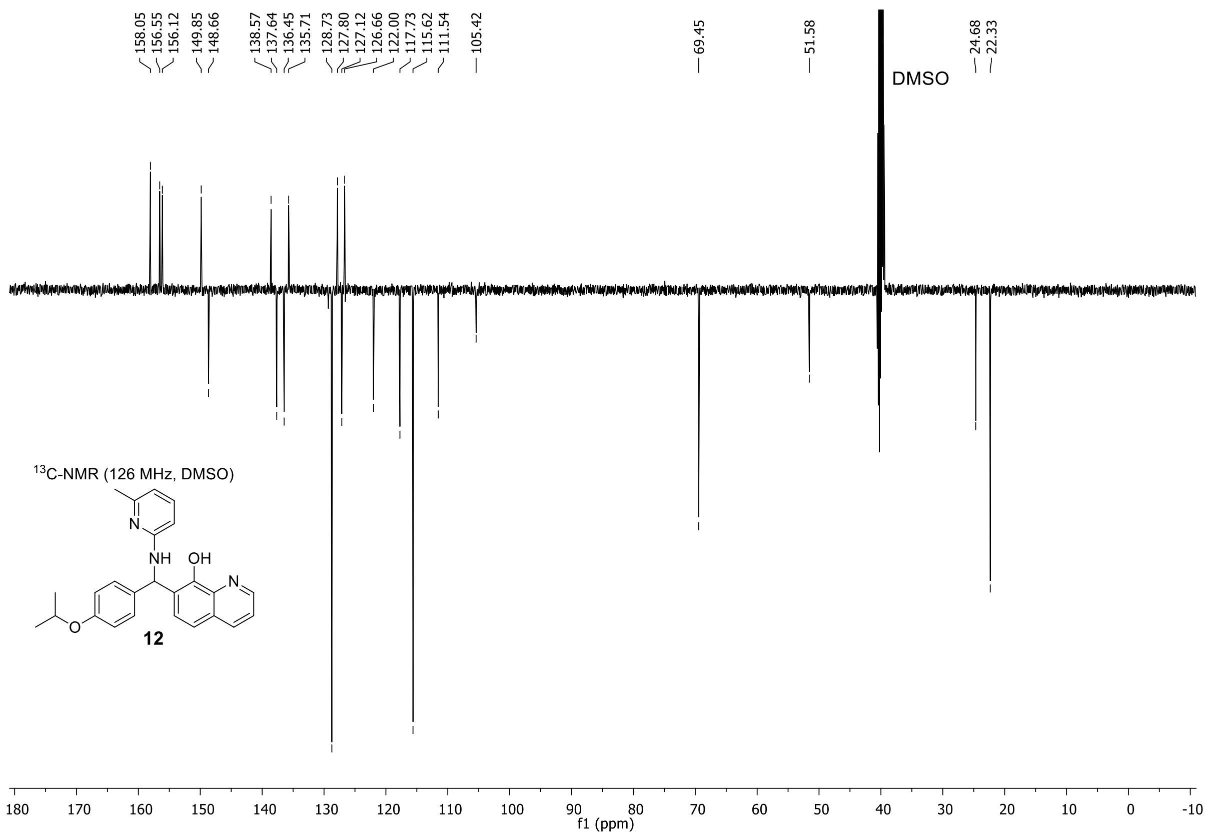
158.05
156.55
156.12
149.85
148.66
138.57
137.64
136.45
135.71
128.73
127.80
127.12
126.66
122.00
117.73
115.62
111.54
105.42
69.45
51.58
24.68
22.33
DMSO
^{13}C-NMR (126 MHz, DMSO)
N
NH OH
N
O
12
f1 (ppm)

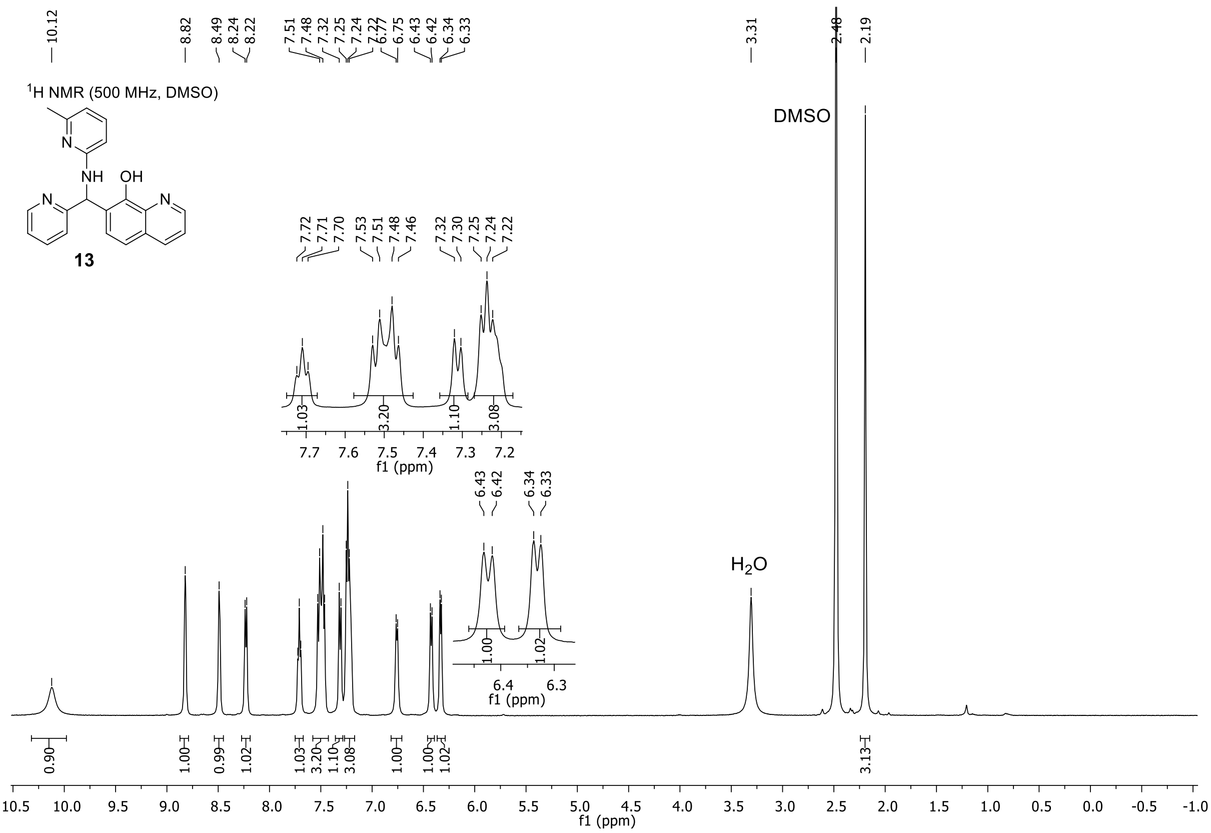
10.12
8.82
8.49
8.24
8.22
7.51
7.48
7.32
7.25
7.24
7.22
6.77
6.75
6.43
6.42
6.34
6.33
3.31
2.48
2.19
^{1}H NMR (500 MHz, DMSO)
N
NH OH
N
N
13
DMSO
H2O
f1 (ppm)

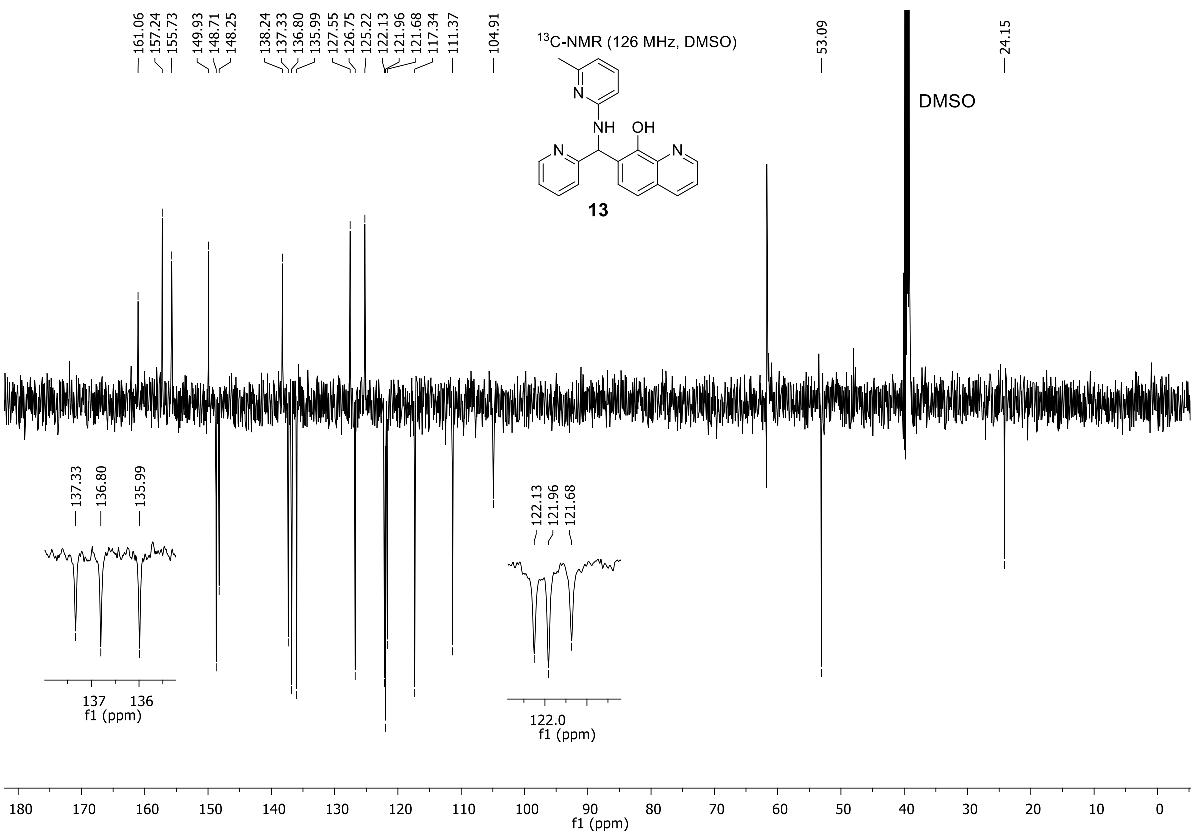
161.06
157.24
155.73
149.93
148.71
148.25
138.24
137.33
136.80
135.99
127.55
126.75
125.22
122.13
121.96
121.68
117.34
111.37
104.91
^{13}C-NMR (126 MHz, DMSO)
N
NH
OH
N
N
13
53.09
24.15
DMSO
137.33
136.80
135.99
137
136
f1 (ppm)
122.13
121.96
121.68
122.0
f1 (ppm)
f1 (ppm)

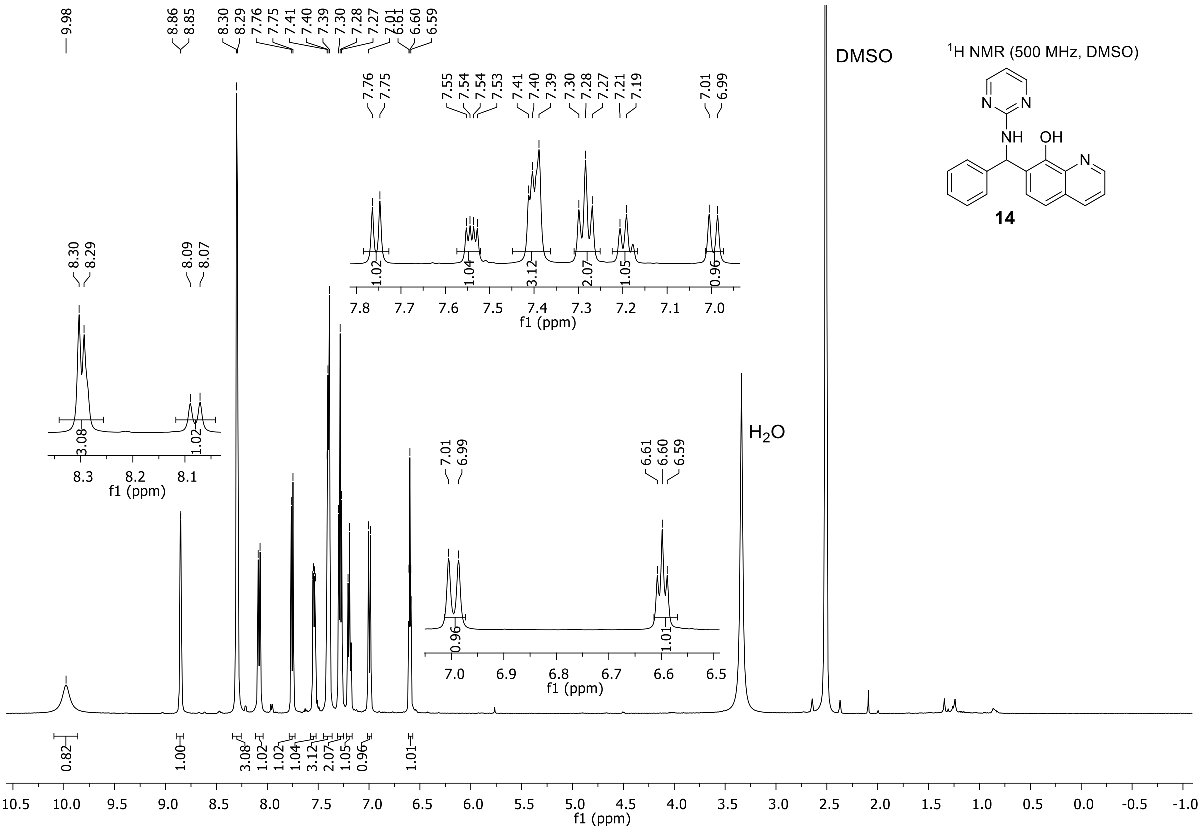
9.98
8.86
8.85
8.30
8.29
7.76
7.75
7.41
7.40
7.39
7.30
7.28
7.27
7.01
6.61
6.60
6.59
DMSO
^{1}H NMR (500 MHz, DMSO)
N
N
NH
OH
N
14
7.76
7.75
7.55
7.54
7.54
7.53
7.41
7.40
7.39
7.30
7.28
7.27
7.21
7.19
7.01
6.99
8.30
8.29
8.09
8.07
3.08
1.02
8.3
8.2
8.1
f1 (ppm)
1.02
1.04
3.12
2.07
1.05
0.96
f1 (ppm)
H_2O
7.01
6.99
6.61
6.60
6.59
0.96
1.01
f1 (ppm)
0.82
1.00
3.08
1.02
1.02
1.04
3.12
2.07
1.05
0.96
1.01
f1 (ppm)

S13

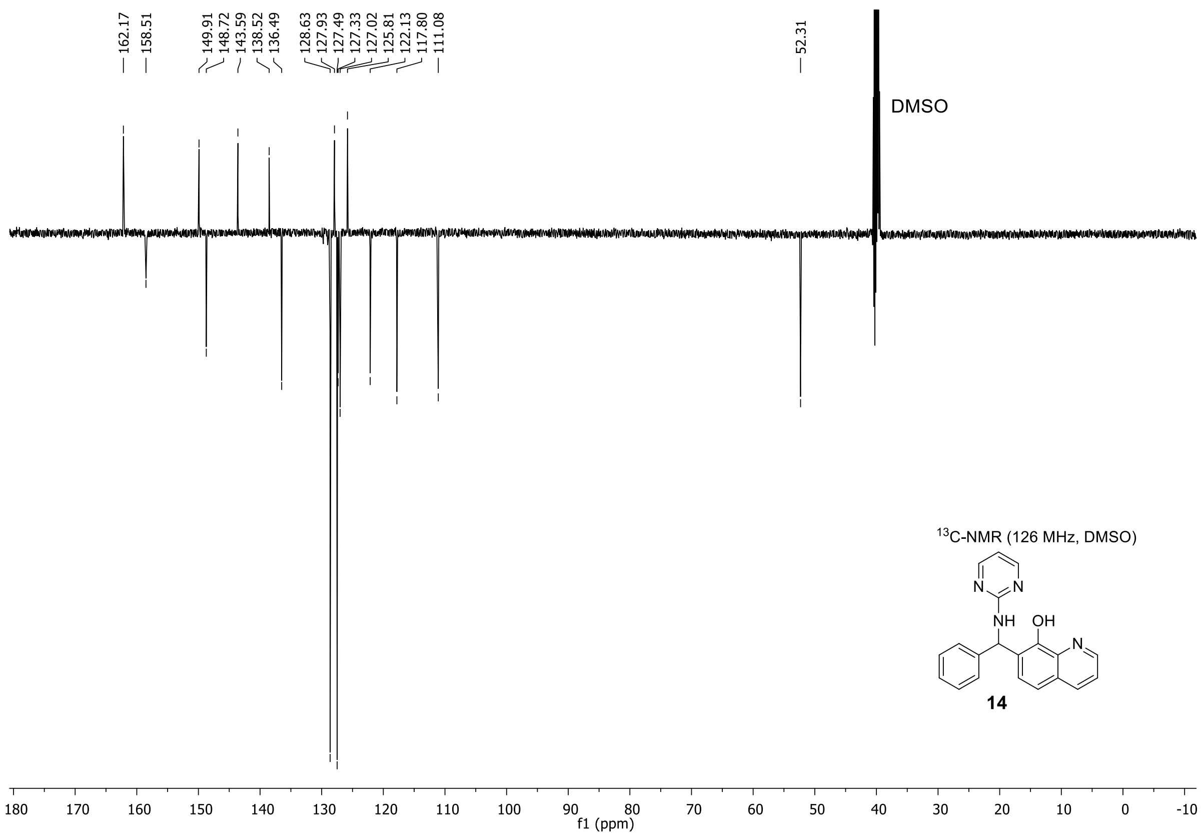
162.17
158.51
149.91
148.72
143.59
138.52
136.49
128.63
127.93
127.49
127.33
127.02
125.81
122.13
117.80
111.08
52.31
DMSO
^{13}C-NMR (126 MHz, DMSO)
N
NH
OH
14
f1 (ppm)

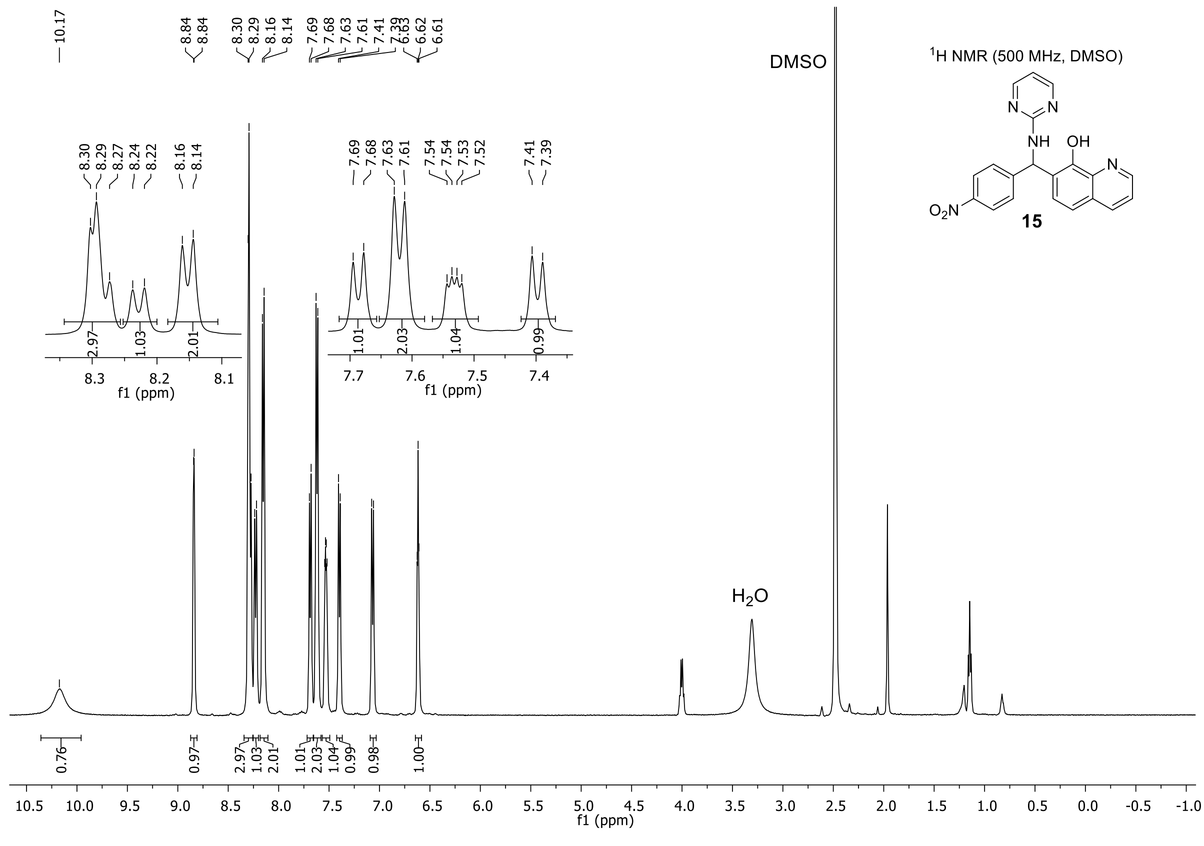
10.17
8.84
8.84
8.30
8.29
8.16
8.14
7.69
7.68
7.63
7.61
7.41
7.39
6.63
6.62
6.61
DMSO
^{1}H NMR (500 MHz, DMSO)
N
NH
OH
O_2N
15
H_2O
f1 (ppm)

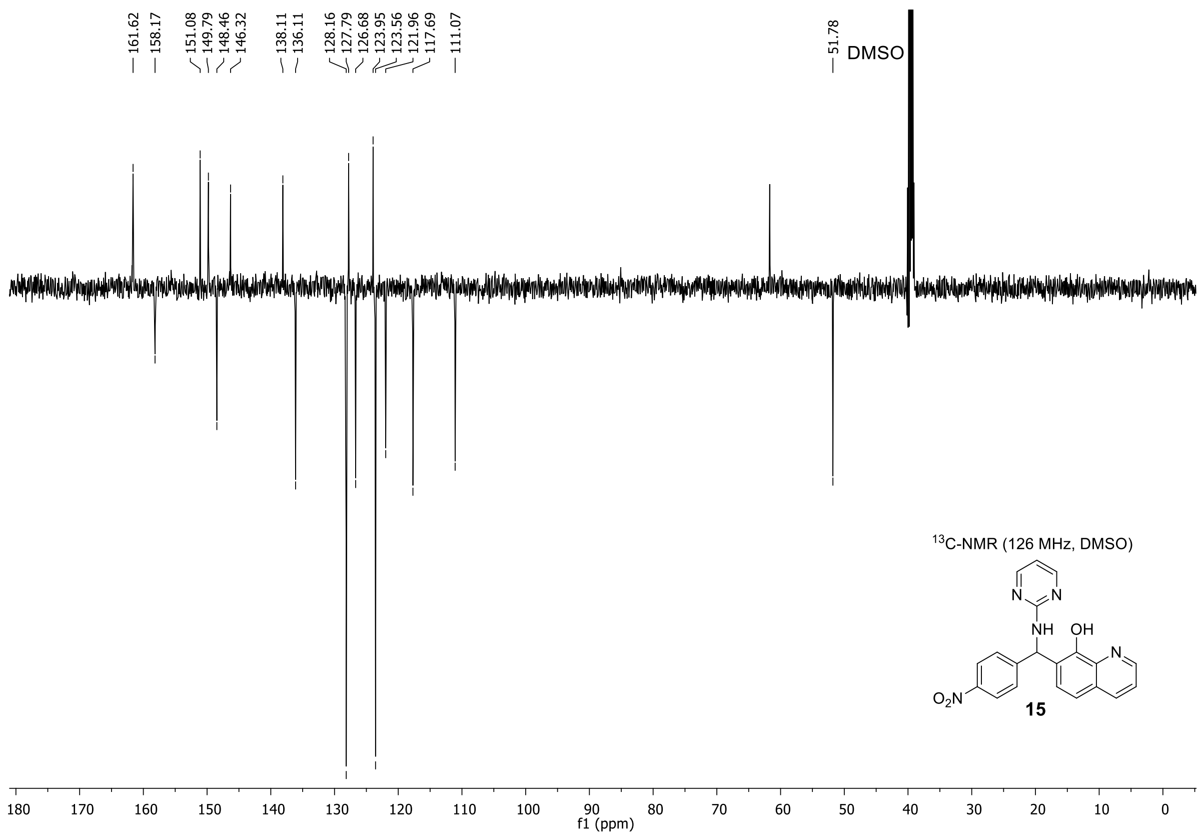
161.62
158.17
151.08
149.79
148.46
146.32
138.11
136.11
128.16
127.79
126.68
123.95
123.56
121.96
117.69
111.07
51.78
DMSO
13C-NMR (126 MHz, DMSO)
N
N
NH
OH
N
O2N
15
180 170 160 150 140 130 120 110 100 90 80 70 60 50 40 30 20 10 0
f1 (ppm)

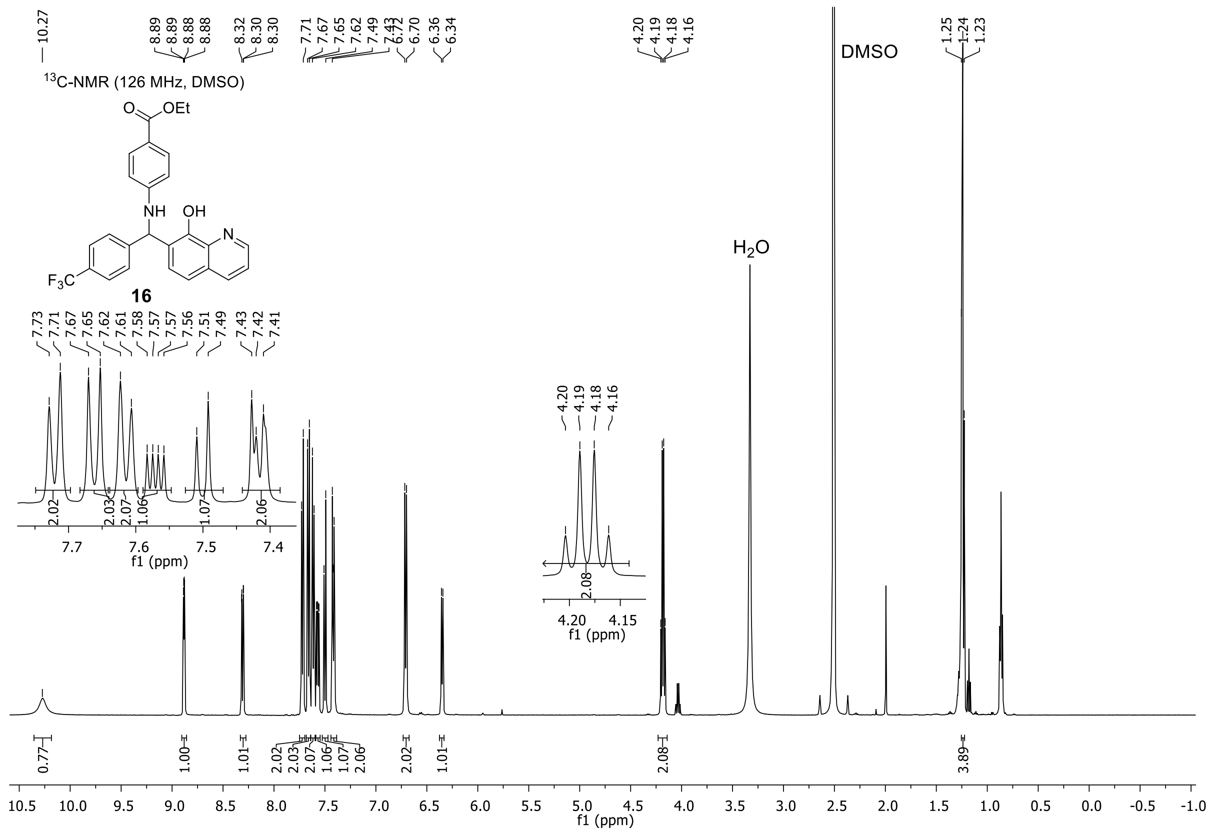
10.27
8.89
8.89
8.88
8.88
8.32
8.30
8.30
7.71
7.67
7.65
7.62
7.49
7.43
6.72
6.70
6.36
6.34
4.20
4.19
4.18
4.16
1.25
1.24
1.23
DMSO
13C-NMR (126 MHz, DMSO)
O
OEt
NH
OH
N
F3C
16
H2O
7.73
7.71
7.67
7.65
7.62
7.61
7.58
7.57
7.57
7.56
7.51
7.49
7.43
7.42
7.41
2.02
2.03
2.07
1.06
1.07
2.06
7.7 7.6 7.5 7.4
f1 (ppm)
2.08
4.20 4.15
f1 (ppm)
0.77
1.00
1.01
2.02
2.03
2.07
1.06
1.07
2.06
2.02
1.01
2.08
3.89
10.5 10.0 9.5 9.0 8.5 8.0 7.5 7.0 6.5 6.0 5.5 5.0 4.5 4.0 3.5 3.0 2.5 2.0 1.5 1.0 0.5 0.0 -0.5 -1.0
f1 (ppm)

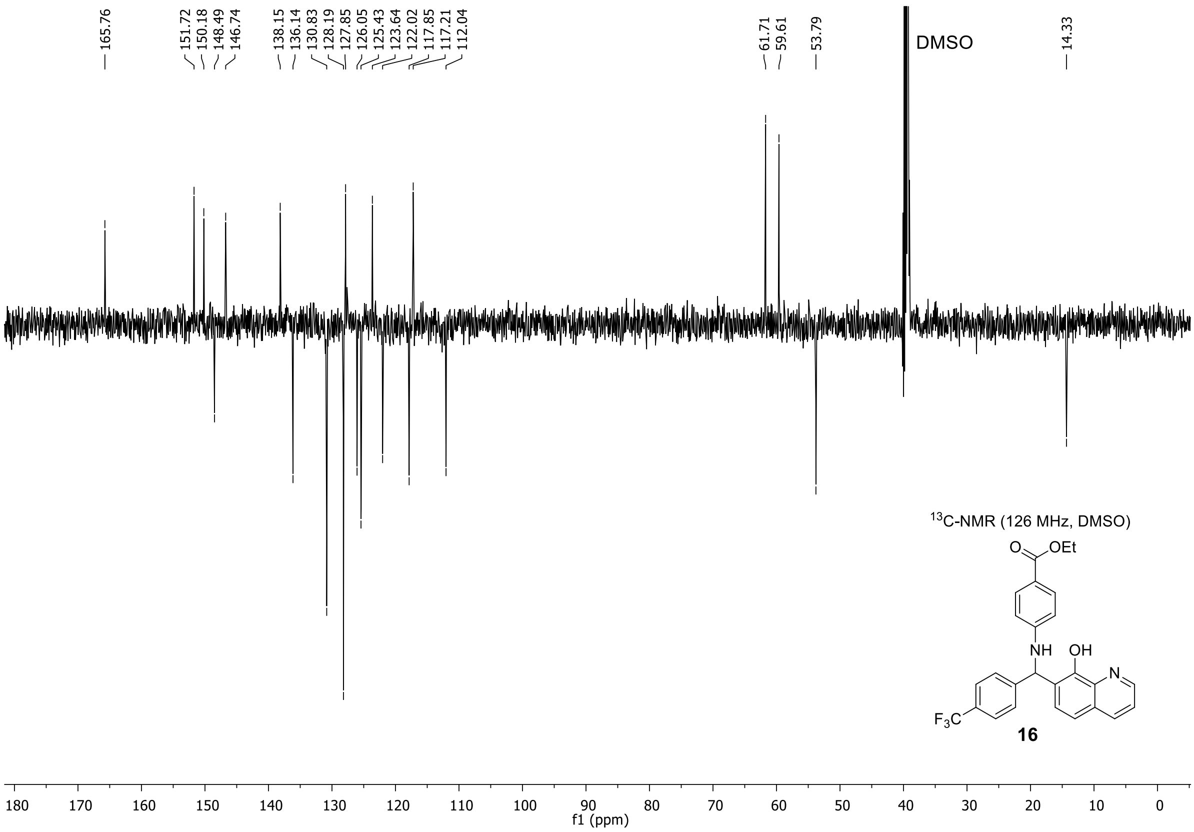
165.76
151.72
150.18
148.49
146.74
138.15
136.14
130.83
128.19
127.85
126.05
125.43
123.64
122.02
117.85
117.21
112.04
61.71
59.61
53.79
DMSO
14.33
13C-NMR (126 MHz, DMSO)
O
OEt
NH
OH
N
F3C
16
f1 (ppm)

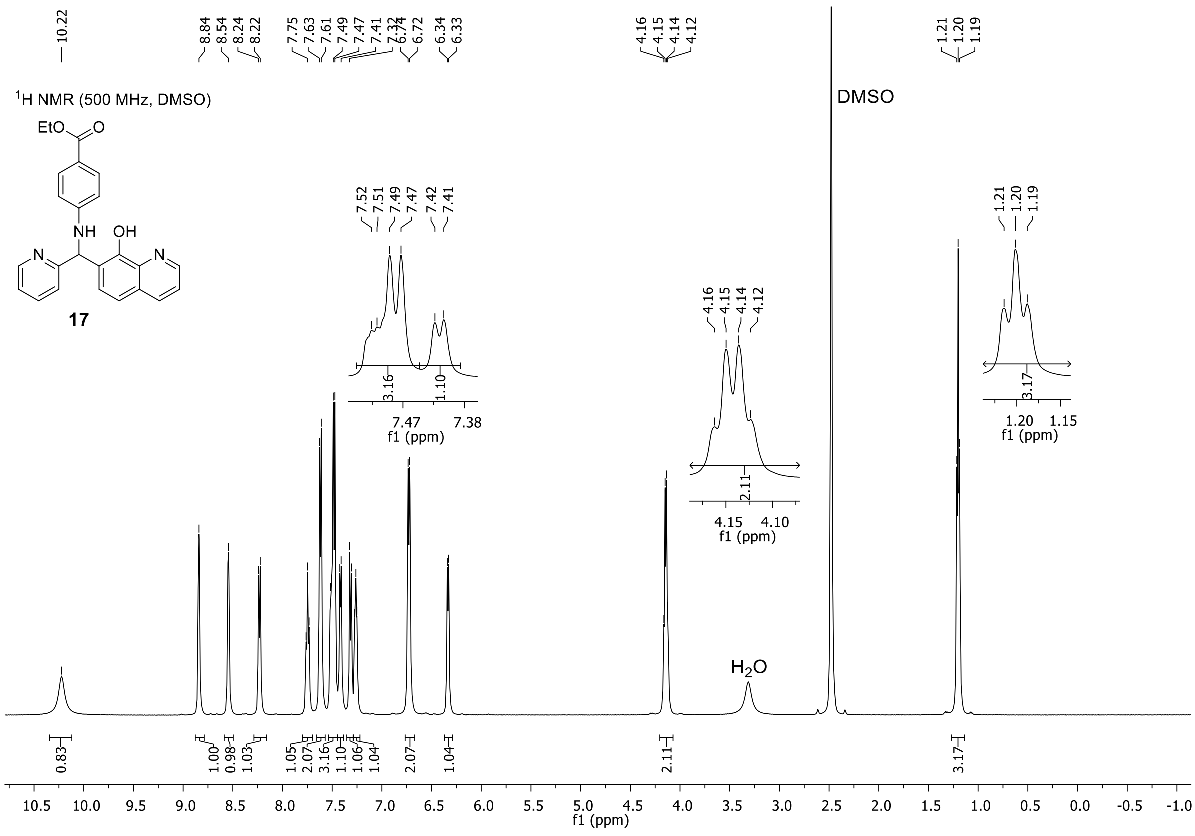
10.22
8.84
8.54
8.24
8.22
7.75
7.63
7.61
7.49
7.47
7.41
7.32
6.74
6.72
6.34
6.33
4.16
4.15
4.14
4.12
1.21
1.20
1.19
1H NMR (500 MHz, DMSO)
EtO
O
NH
OH
N
N
17
DMSO
H2O
0.83
1.00
0.98
1.03
1.05
2.07
3.16
1.10
1.06
1.04
2.07
1.04
2.11
3.17
f1 (ppm)

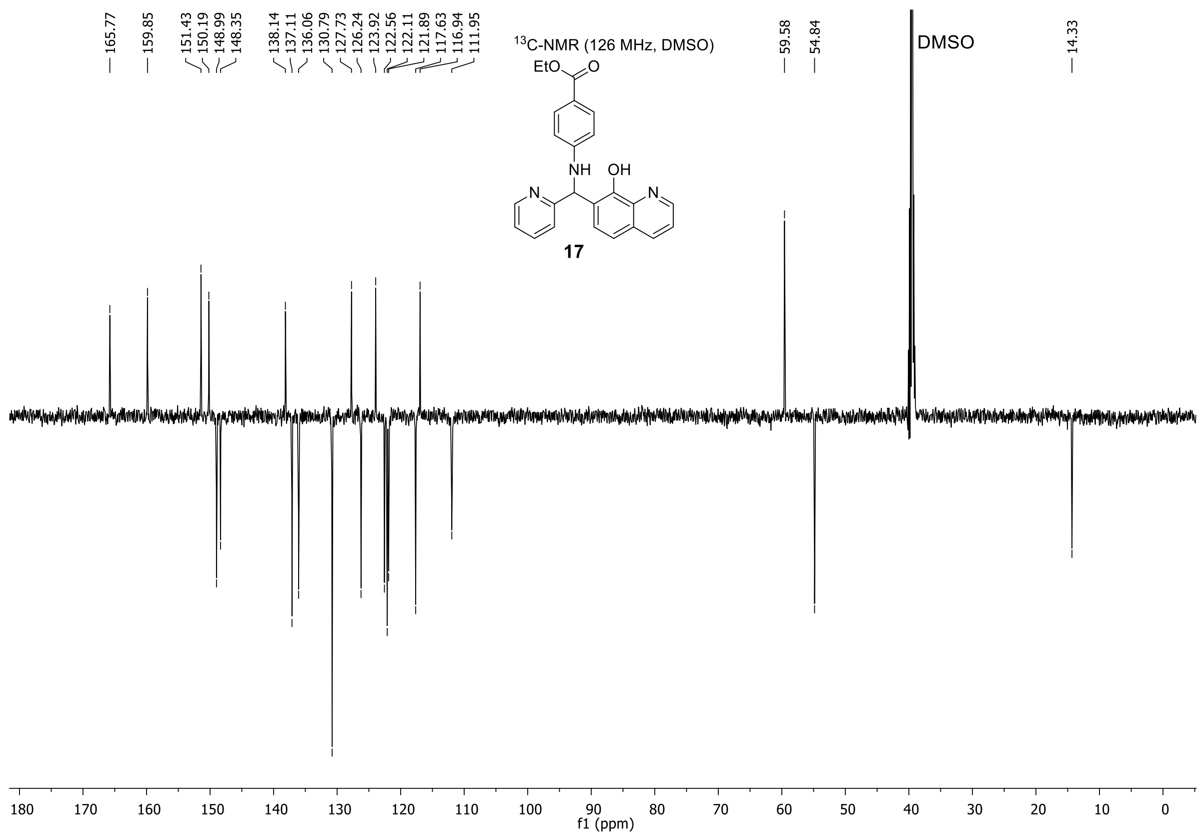
165.77
159.85
151.43
150.19
148.99
148.35
138.14
137.11
136.06
130.79
127.73
126.24
123.92
122.56
122.11
121.89
117.63
116.94
111.95
^{13}C-NMR (126 MHz, DMSO)
EtO
O
NH
OH
N
N
17
59.58
54.84
DMSO
14.33
180 170 160 150 140 130 120 110 100 90 80 70 60 50 40 30 20 10 0
f1 (ppm)

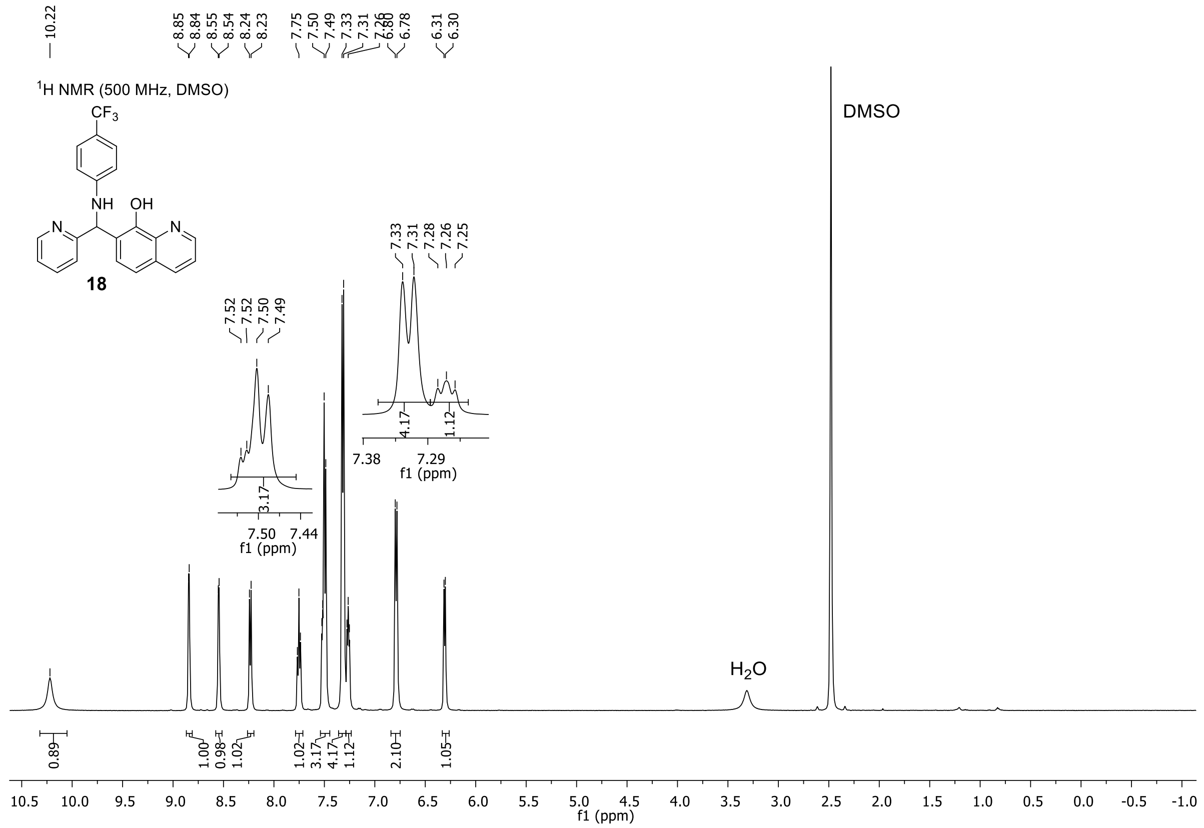
10.22
8.85
8.84
8.55
8.54
8.24
8.23
7.75
7.50
7.49
7.33
7.31
6.80
6.78
6.31
6.30
^{1}H NMR (500 MHz, DMSO)
CF_3
NH
OH
N
N
18
DMSO
7.52
7.52
7.50
7.49
3.17
7.50 7.44
f1 (ppm)
7.33
7.31
7.28
7.26
7.25
4.17
1.12
7.38 7.29
f1 (ppm)
H_2O
0.89
1.00
0.98
1.02
1.02
3.17
4.17
1.12
2.10
1.05
10.5 10.0 9.5 9.0 8.5 8.0 7.5 7.0 6.5 6.0 5.5 5.0 4.5 4.0 3.5 3.0 2.5 2.0 1.5 1.0 0.5 0.0 -0.5 -1.0
f1 (ppm)

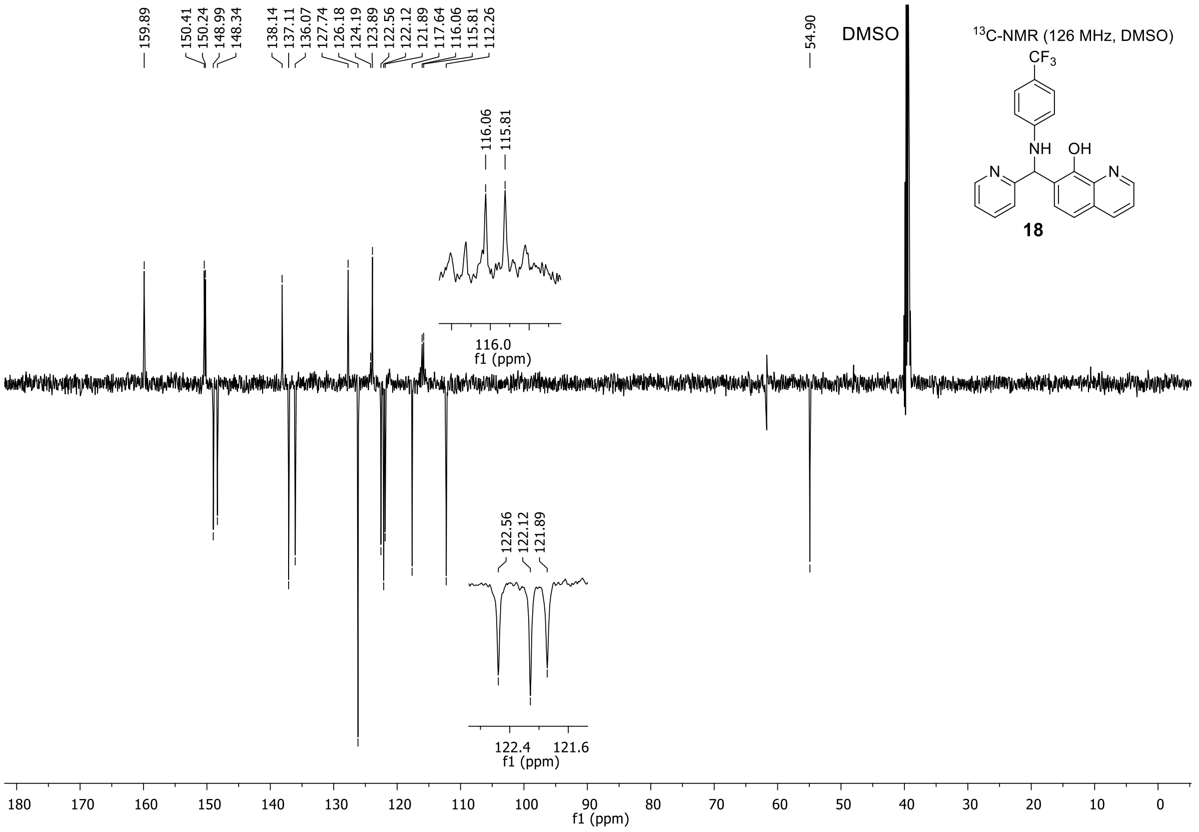
13C-NMR (126 MHz, DMSO)
CF3
NH OH
N N
18
DMSO
f1 (ppm)

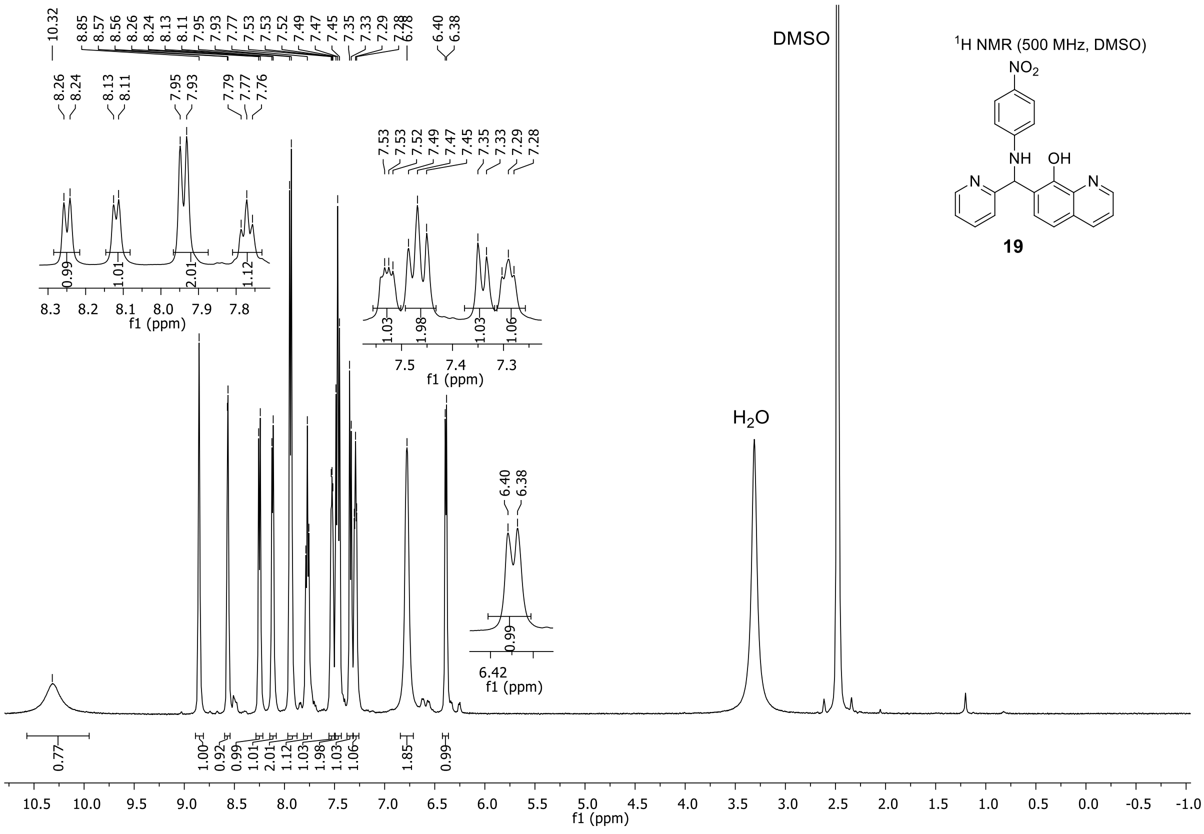
1H NMR (500 MHz, DMSO)
NO2
NH OH
N N
19
DMSO
H2O
f1 (ppm)

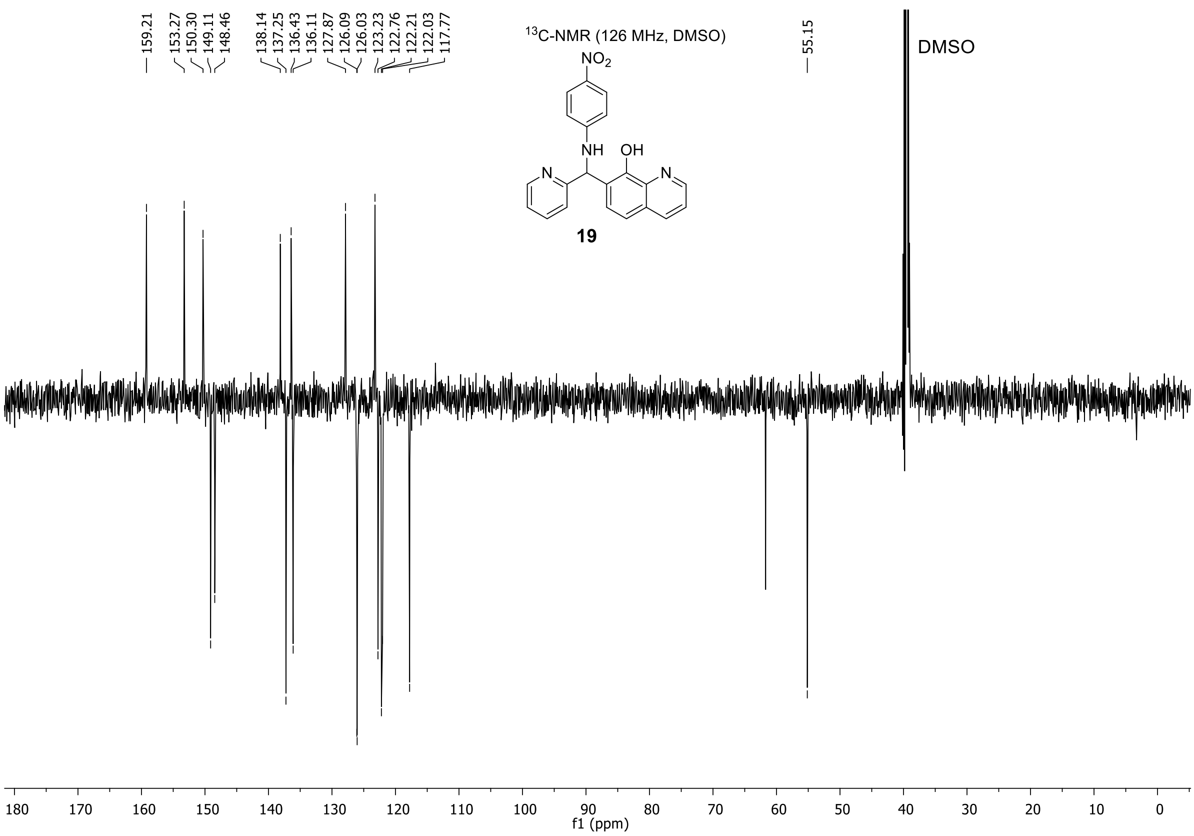

159.21
153.27
150.30
149.11
148.46
138.14
137.25
136.43
136.11
127.87
126.09
126.03
123.23
122.76
122.21
122.03
117.77
13C-NMR (126 MHz, DMSO)
55.15
DMSO
NO2
NH
OH
N
N
19
180 170 160 150 140 130 120 110 100 90 80 70 60 50 40 30 20 10 0
f1 (ppm)

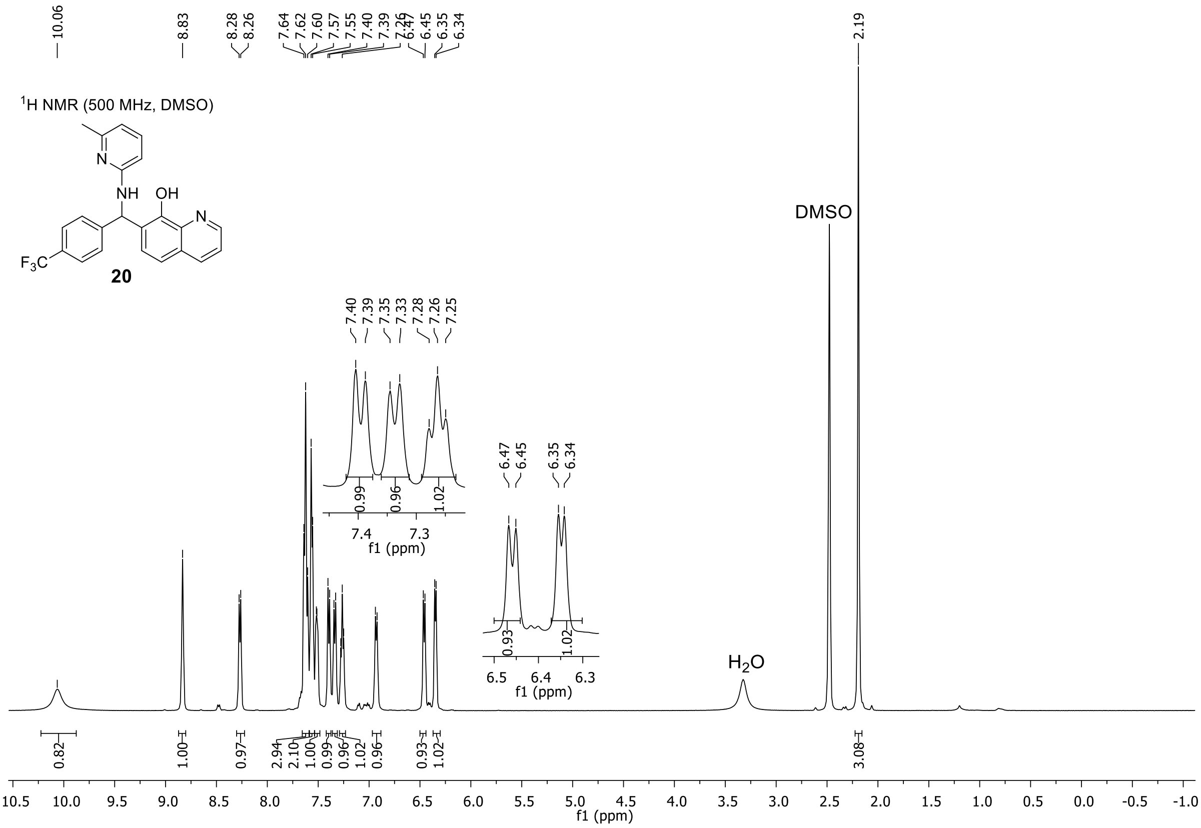

10.06
8.83
8.28
8.26
7.64
7.62
7.60
7.57
7.55
7.40
7.39
7.26
6.47
6.45
6.35
6.34
2.19
1H NMR (500 MHz, DMSO)
N
NH
OH
N
F3C
20
DMSO
H2O
0.82
1.00
0.97
2.94
2.10
1.00
0.99
0.96
1.02
0.96
0.93
1.02
3.08
10.5 10.0 9.5 9.0 8.5 8.0 7.5 7.0 6.5 6.0 5.5 5.0 4.5 4.0 3.5 3.0 2.5 2.0 1.5 1.0 0.5 0.0 -0.5 -1.0
f1 (ppm)

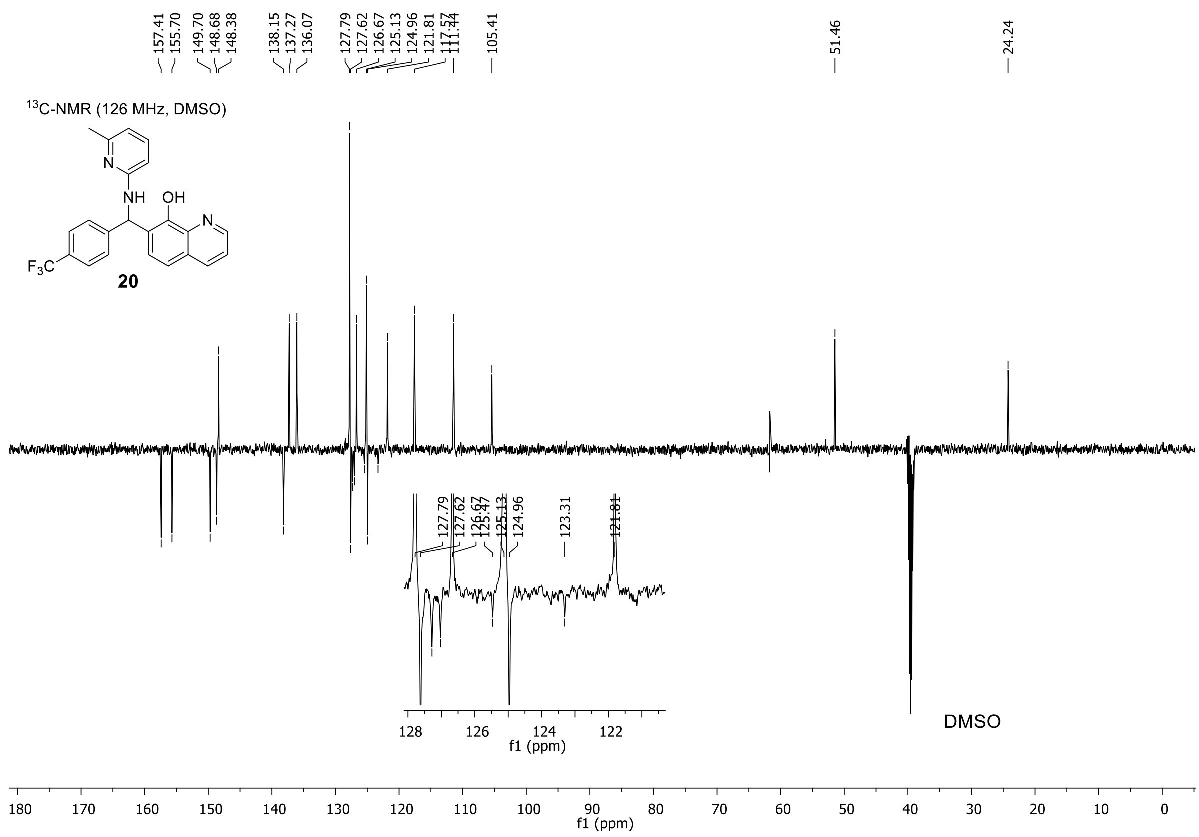
13C-NMR (126 MHz, DMSO)
157.41
155.70
149.70
148.68
148.38
138.15
137.27
136.07
127.79
127.62
126.67
125.13
124.96
121.81
117.57
111.44
105.41
51.46
24.24
NH OH
N
F3C
20
123.31
DMSO
f1 (ppm)

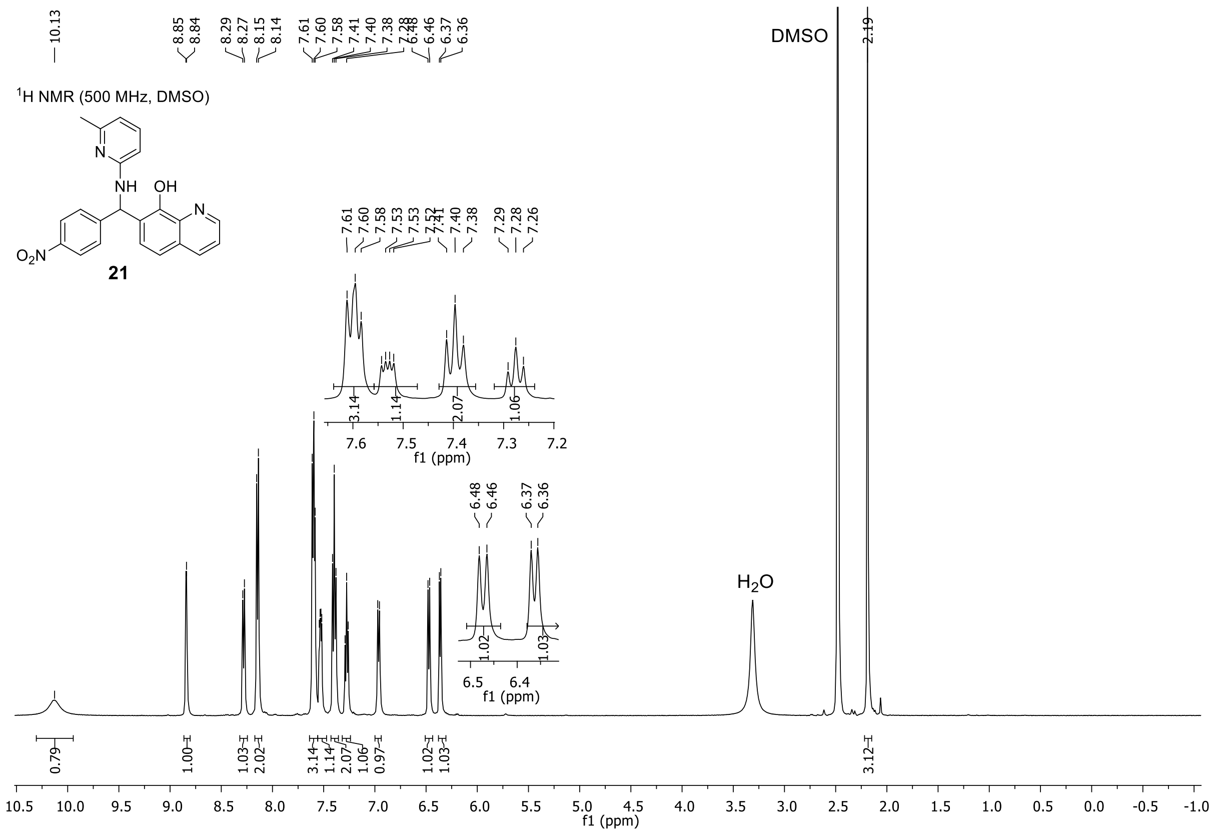
1H NMR (500 MHz, DMSO)
10.13
8.85
8.84
8.29
8.27
8.15
8.14
7.61
7.60
7.58
7.41
7.40
7.38
7.28
6.48
6.46
6.37
6.36
DMSO
2.19
NH OH
N
O2N
21
H2O
f1 (ppm)

S20

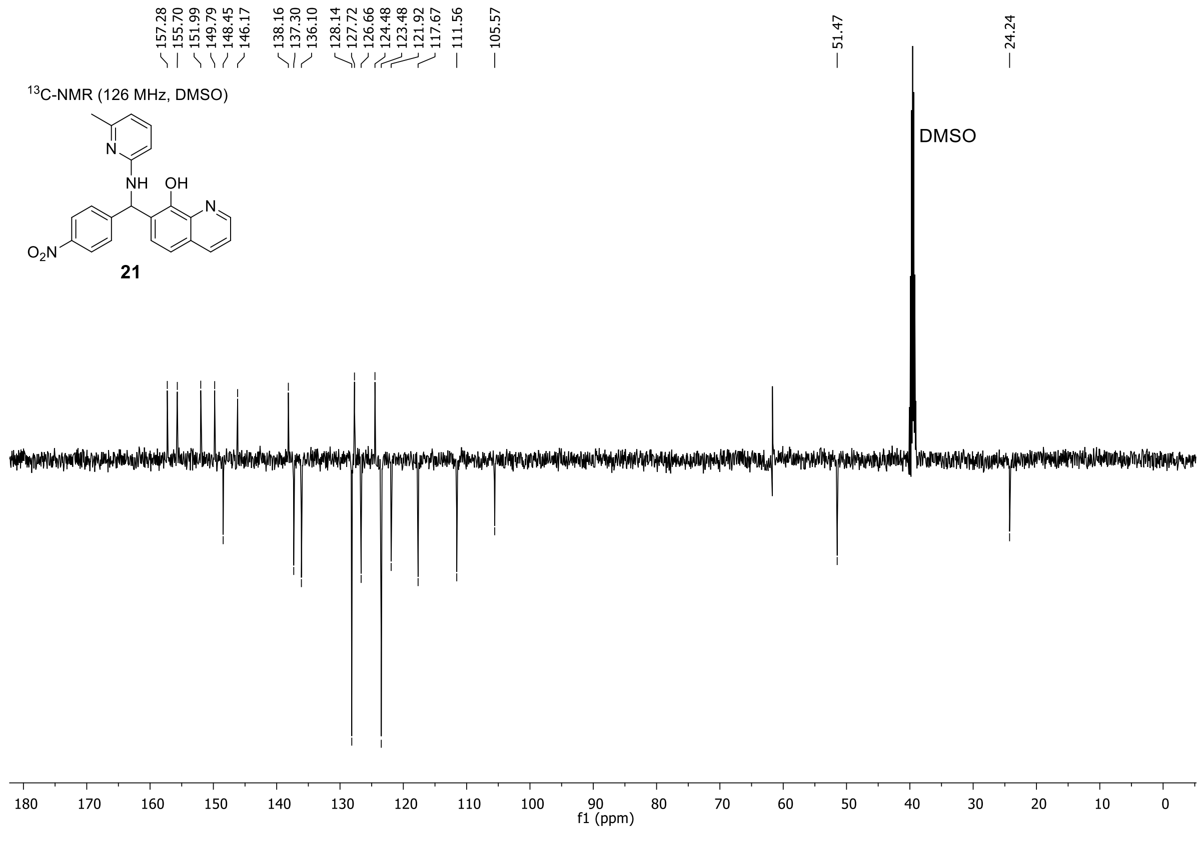

13C-NMR (126 MHz, DMSO)
21
DMSO
f1 (ppm)

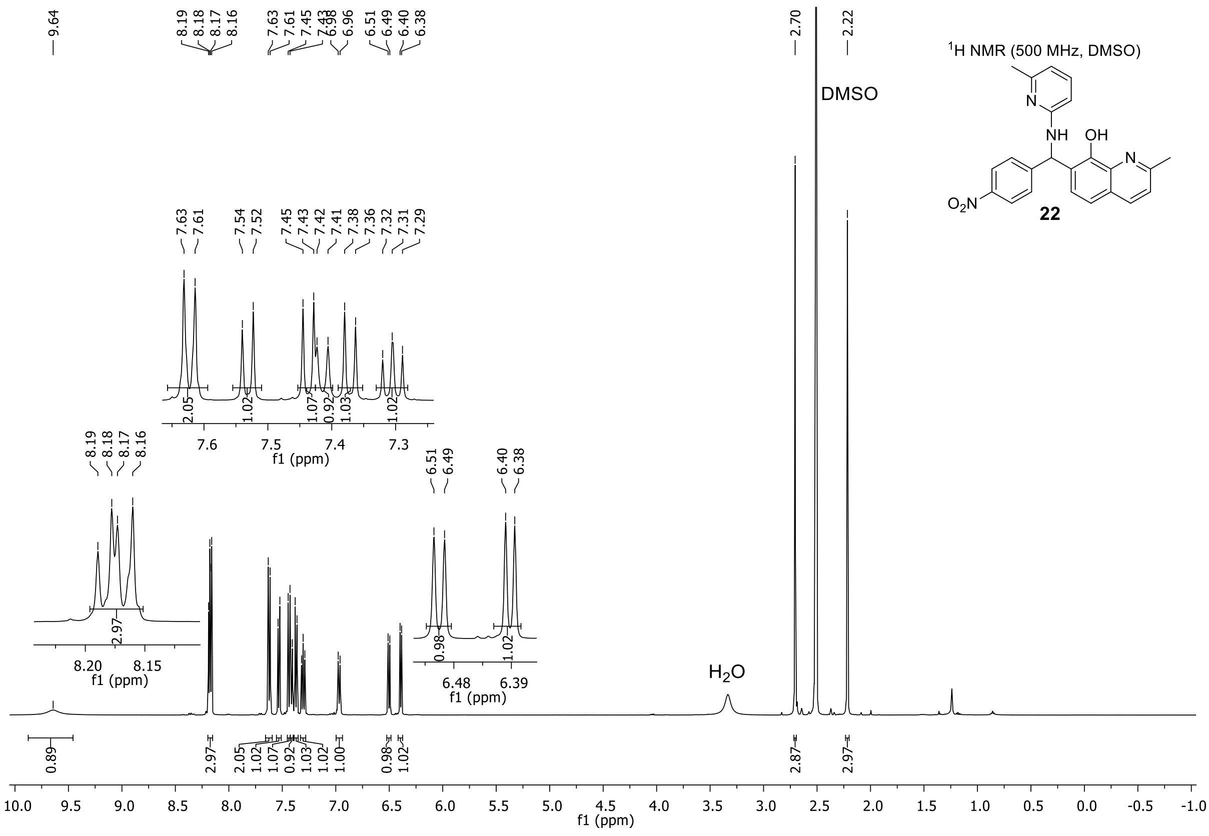

1H NMR (500 MHz, DMSO)
22
DMSO
H2O
f1 (ppm)

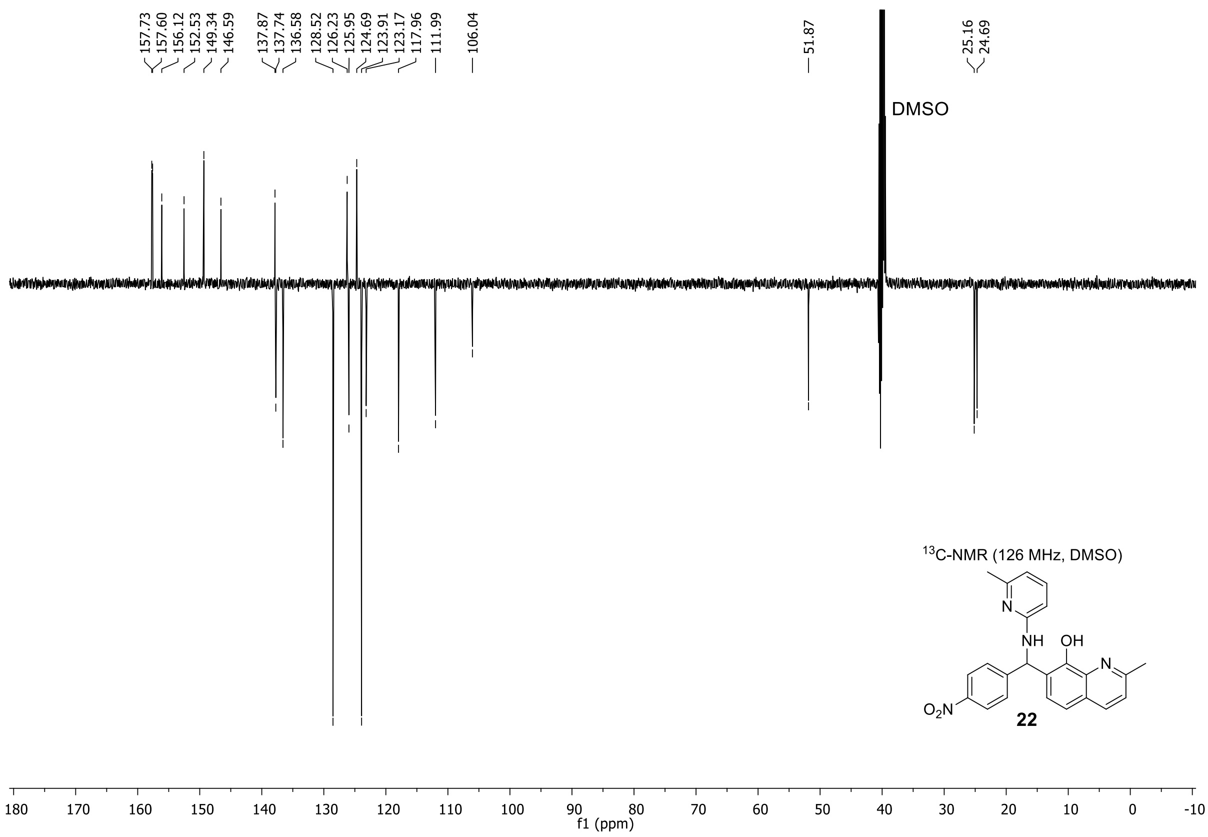
157.73
157.60
156.12
152.53
149.34
146.59
137.87
137.74
136.58
128.52
126.23
125.95
124.69
123.91
123.17
117.96
111.99
106.04
51.87
25.16
24.69
DMSO
13C-NMR (126 MHz, DMSO)
N
NH
OH
O2N
22
f1 (ppm)

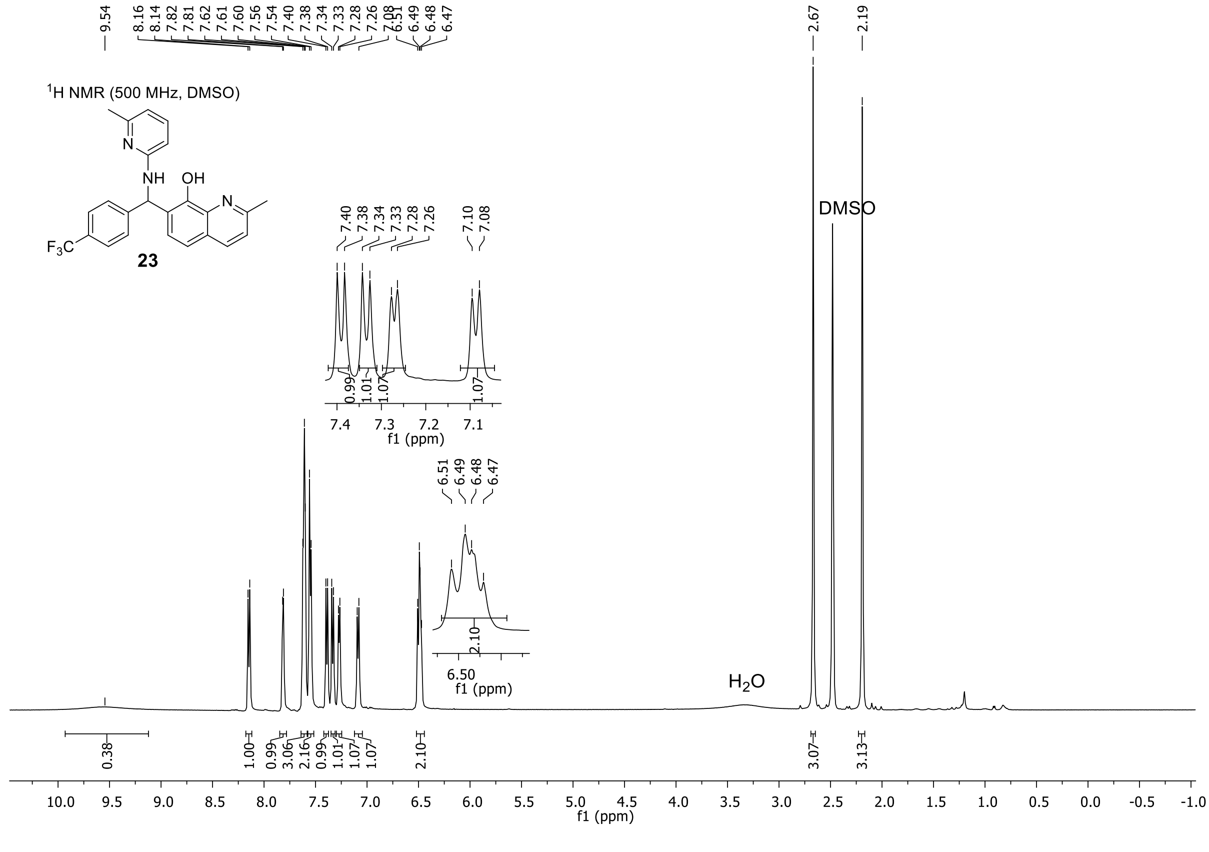
1H NMR (500 MHz, DMSO)
N
NH
OH
F3C
23
DMSO
H2O
f1 (ppm)

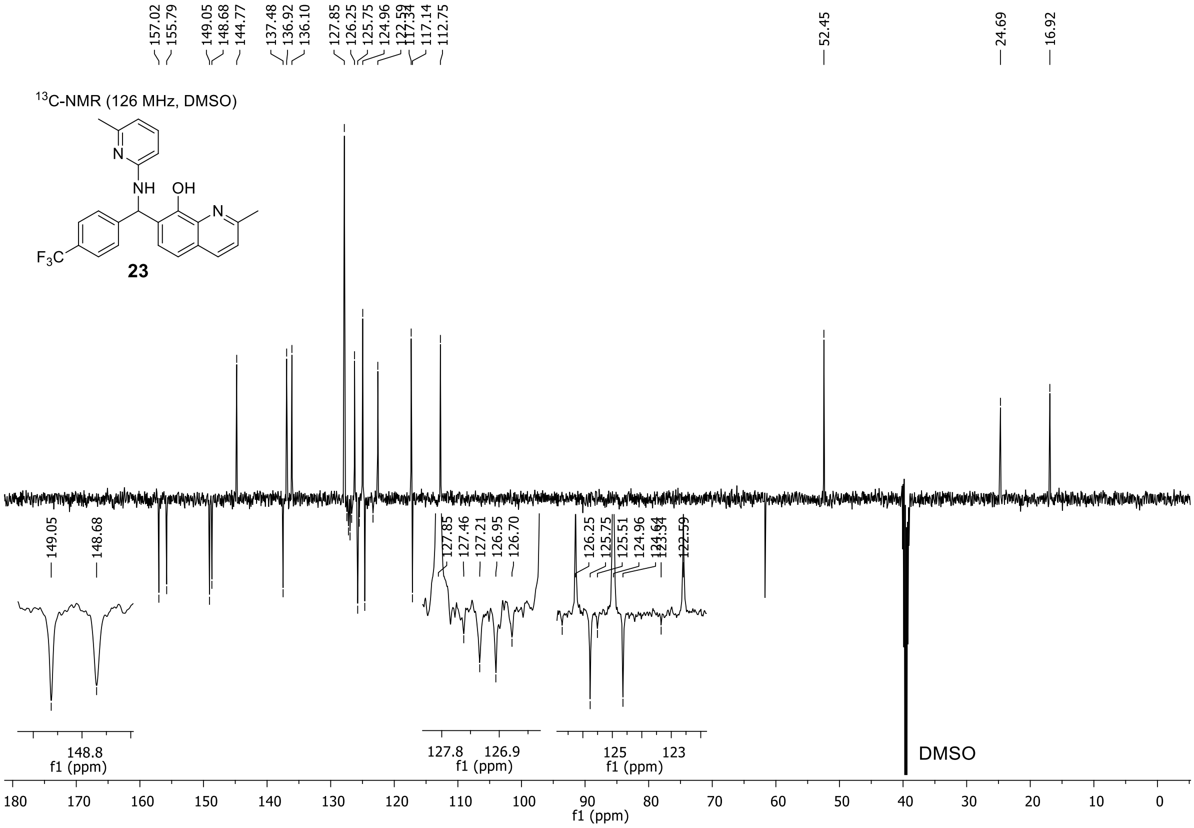

157.02
155.79
149.05
148.68
144.77
137.48
136.92
136.10
127.85
126.25
125.75
124.96
122.59
117.34
117.14
112.75
52.45
24.69
16.92
13C-NMR (126 MHz, DMSO)
NH
OH
N
F3C
23
149.05
148.68
127.85
127.46
127.21
126.95
126.70
126.25
125.75
125.51
124.96
122.59
148.8
f1 (ppm)
127.8
126.9
f1 (ppm)
125
123
f1 (ppm)
DMSO
f1 (ppm)

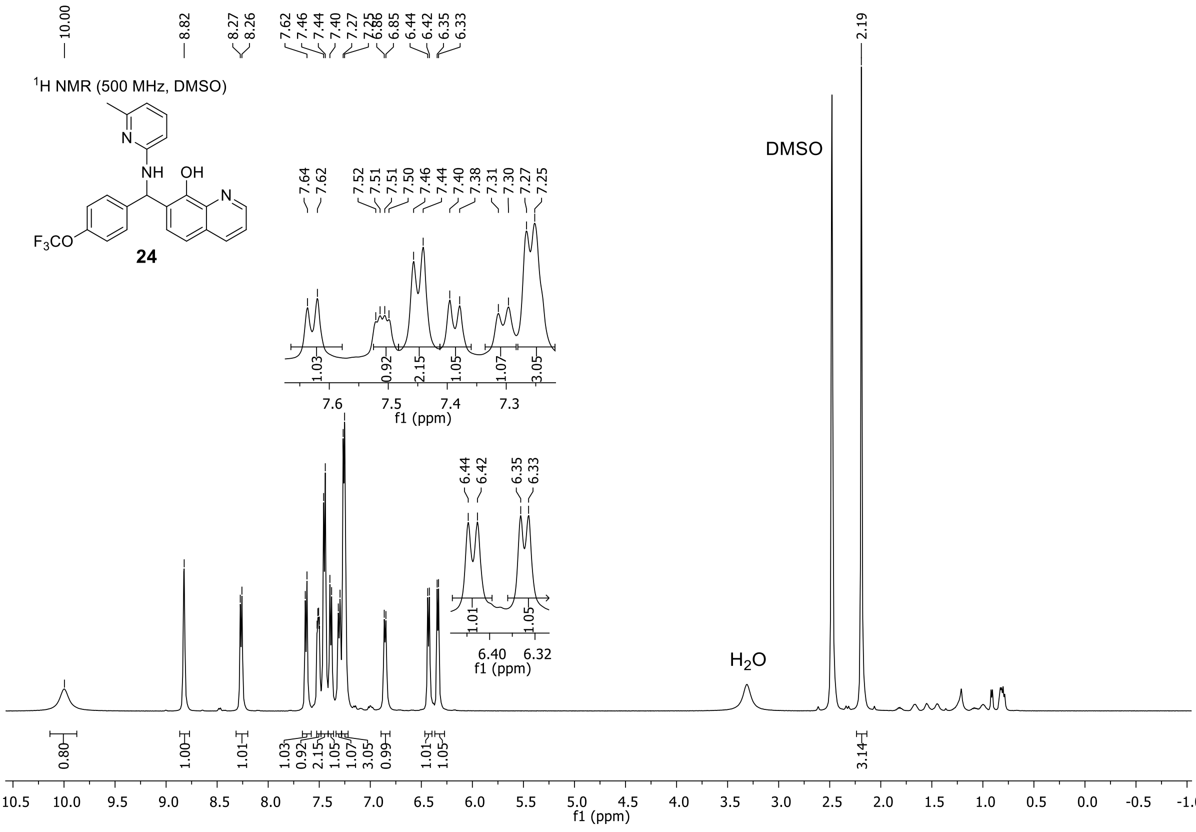

10.00
8.82
8.27
8.26
7.62
7.46
7.44
7.40
7.27
7.25
6.85
6.44
6.42
6.35
6.33
2.19
1H NMR (500 MHz, DMSO)
NH
OH
N
F3CO
24
DMSO
7.64
7.62
7.52
7.51
7.51
7.50
7.46
7.44
7.40
7.38
7.31
7.30
7.27
7.25
1.03
0.92
2.15
1.05
1.07
3.05
7.6
7.5
7.4
7.3
f1 (ppm)
6.44
6.42
6.35
6.33
1.01
1.05
6.40
6.32
f1 (ppm)
H2O
0.80
1.00
1.01
0.99
3.14
f1 (ppm)

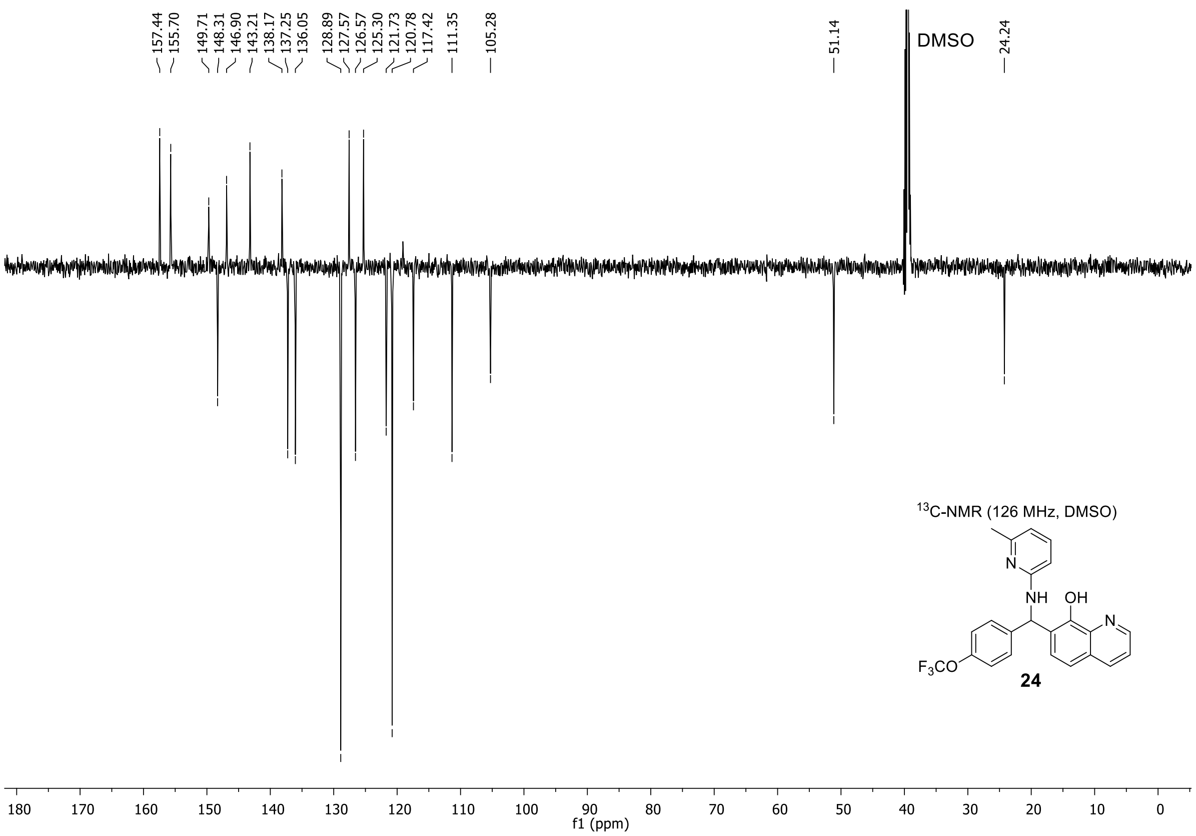

157.44
155.70
149.71
148.31
146.90
143.21
138.17
137.25
136.05
128.89
127.57
126.57
125.30
121.73
120.78
117.42
111.35
105.28
51.14
DMSO
24.24
^{13}C-NMR (126 MHz, DMSO)
NH
OH
N
N
F_3CO
24
f1 (ppm)

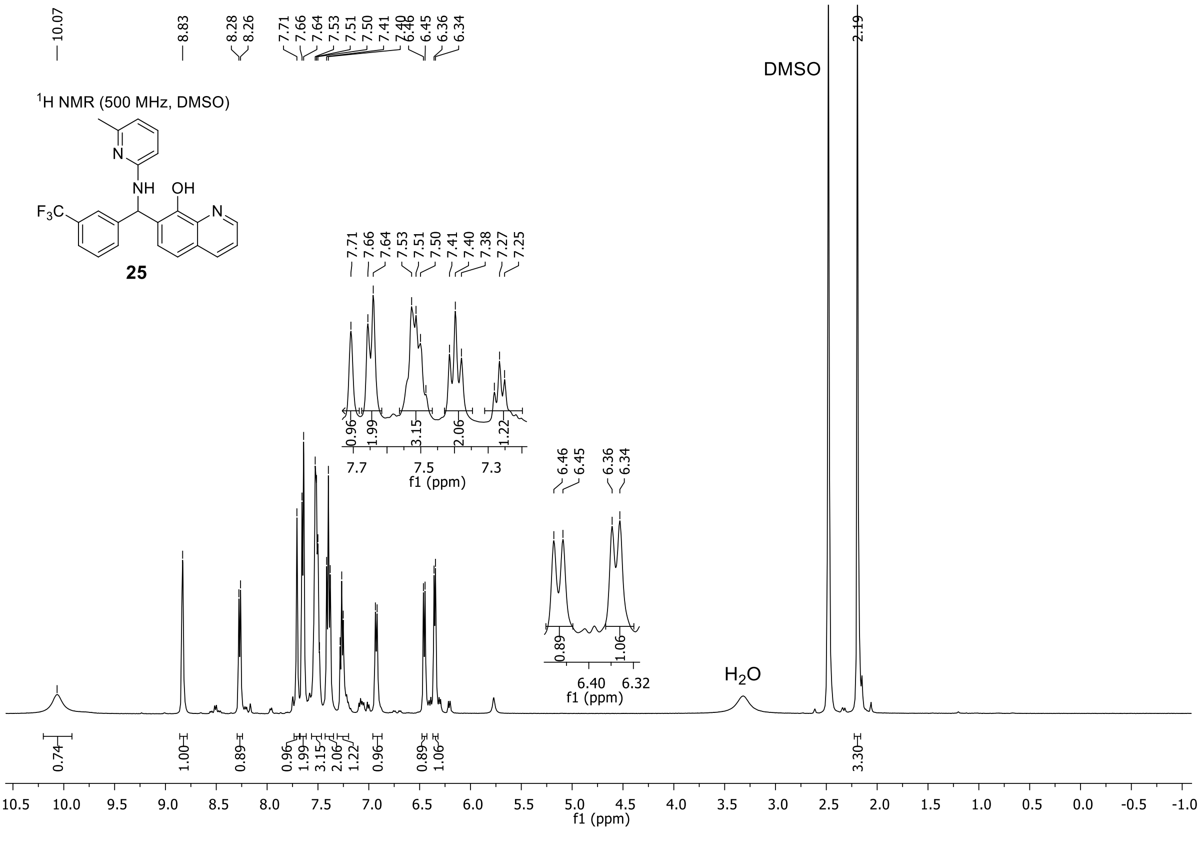

10.07
8.83
8.28
8.26
7.71
7.66
7.64
7.53
7.51
7.50
7.41
7.40
6.46
6.45
6.36
6.34
2.19
DMSO
^{1}H NMR (500 MHz, DMSO)
N
NH
OH
N
F_3C
25
7.38
7.27
7.25
0.96
1.99
3.15
2.06
1.22
0.89
1.06
H_2O
0.74
1.00
0.89
0.96
3.30
f1 (ppm)

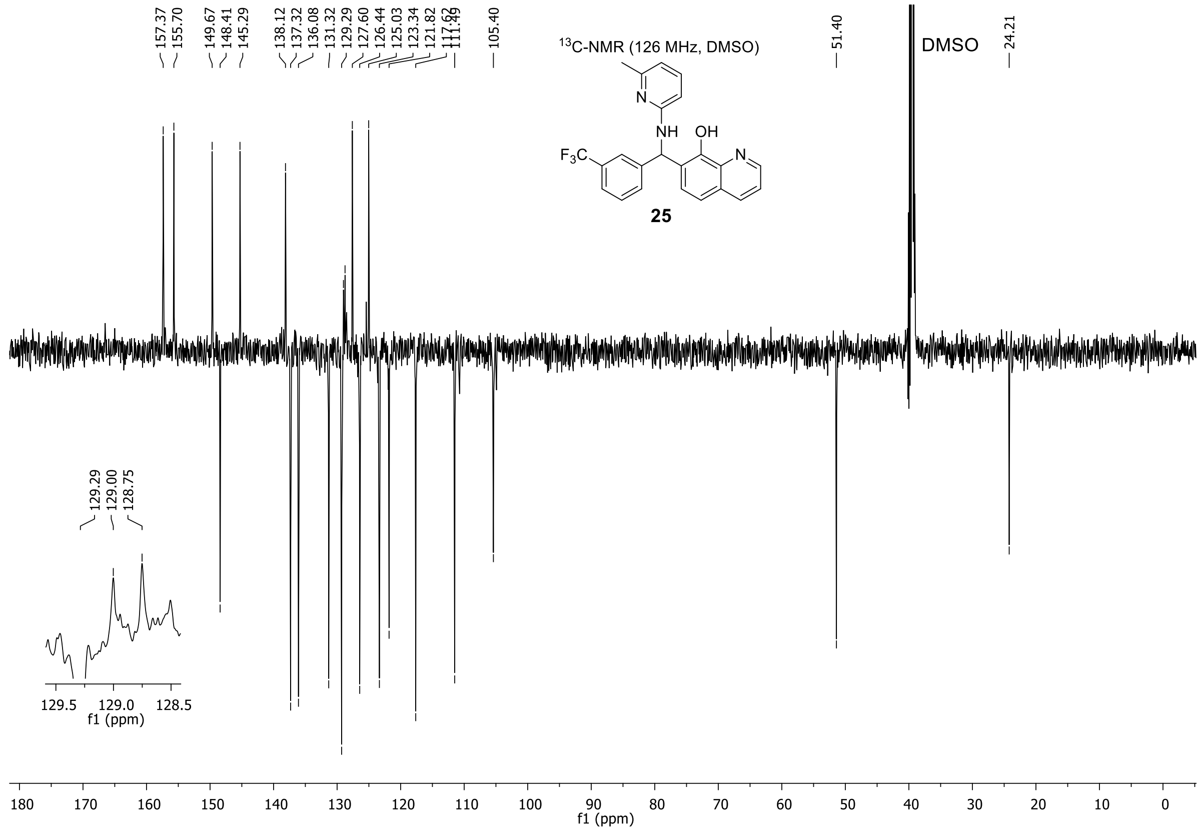

157.37
155.70
149.67
148.41
145.29
138.12
137.32
136.08
131.32
129.29
127.60
126.44
125.03
123.34
121.82
117.62
111.49
105.40
^{13}C-NMR (126 MHz, DMSO)
51.40
DMSO
24.21
F_3C
NH OH
N
25
129.29
129.00
128.75
129.5 129.0 128.5
f1 (ppm)
f1 (ppm)

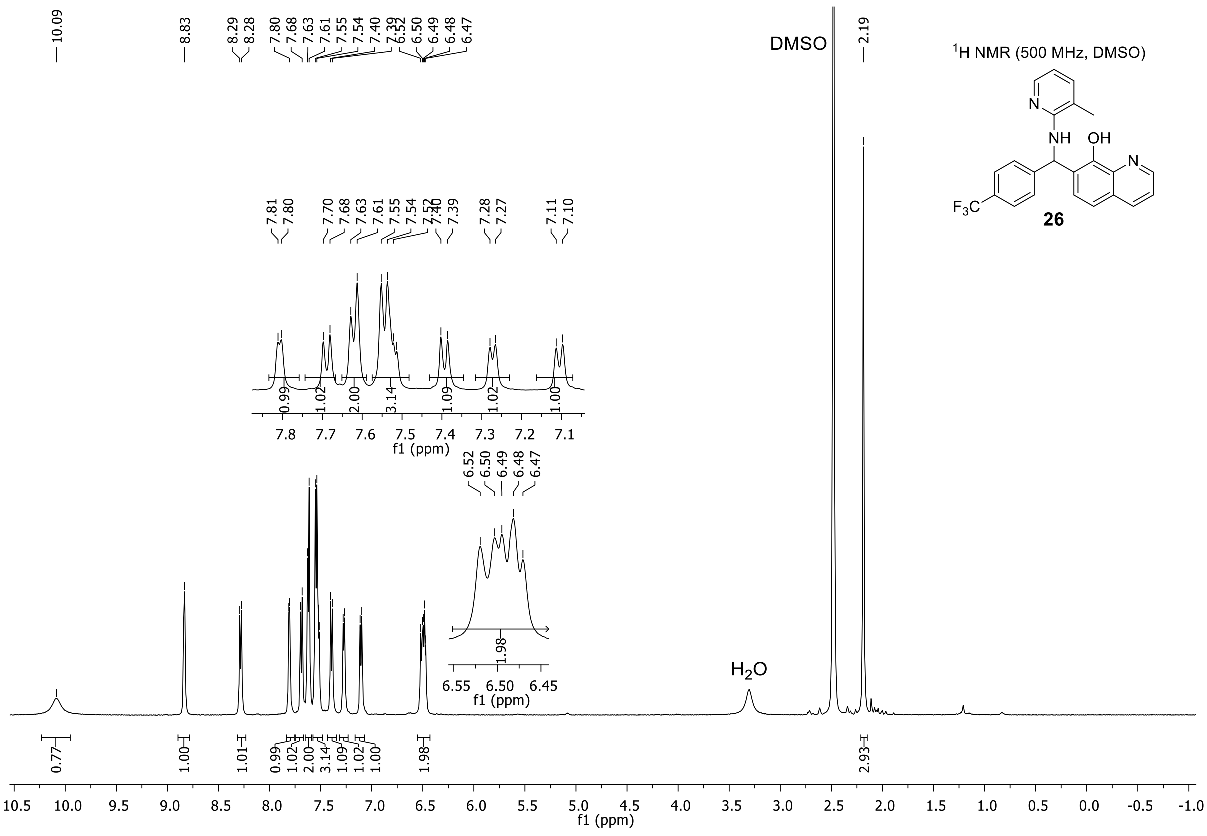

10.09
8.83
8.29
8.28
7.80
7.68
7.63
7.61
7.55
7.54
7.40
7.39
6.52
6.50
6.49
6.48
6.47
DMSO
2.19
^{1}H NMR (500 MHz, DMSO)
NH OH
F_3C
26
7.81
7.80
7.70
7.68
7.63
7.61
7.55
7.54
7.40
7.39
7.28
7.27
7.11
7.10
0.99
1.02
2.00
3.14
1.09
1.02
1.00
7.8 7.7 7.6 7.5 7.4 7.3 7.2 7.1
f1 (ppm)
1.98
6.55 6.50 6.45
f1 (ppm)
H_2O
0.77
1.00
1.01
2.93
f1 (ppm)

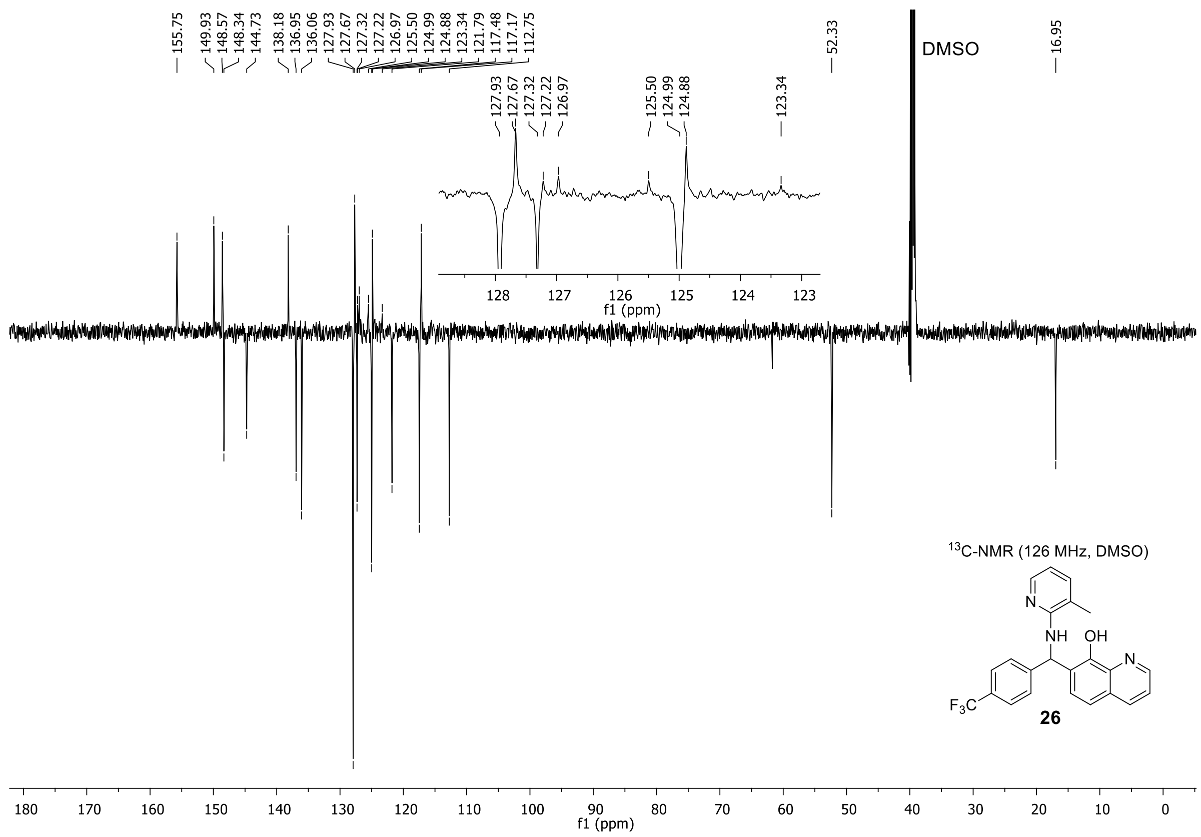
DMSO
^{13}C-NMR (126 MHz, DMSO)
26
f1 (ppm)

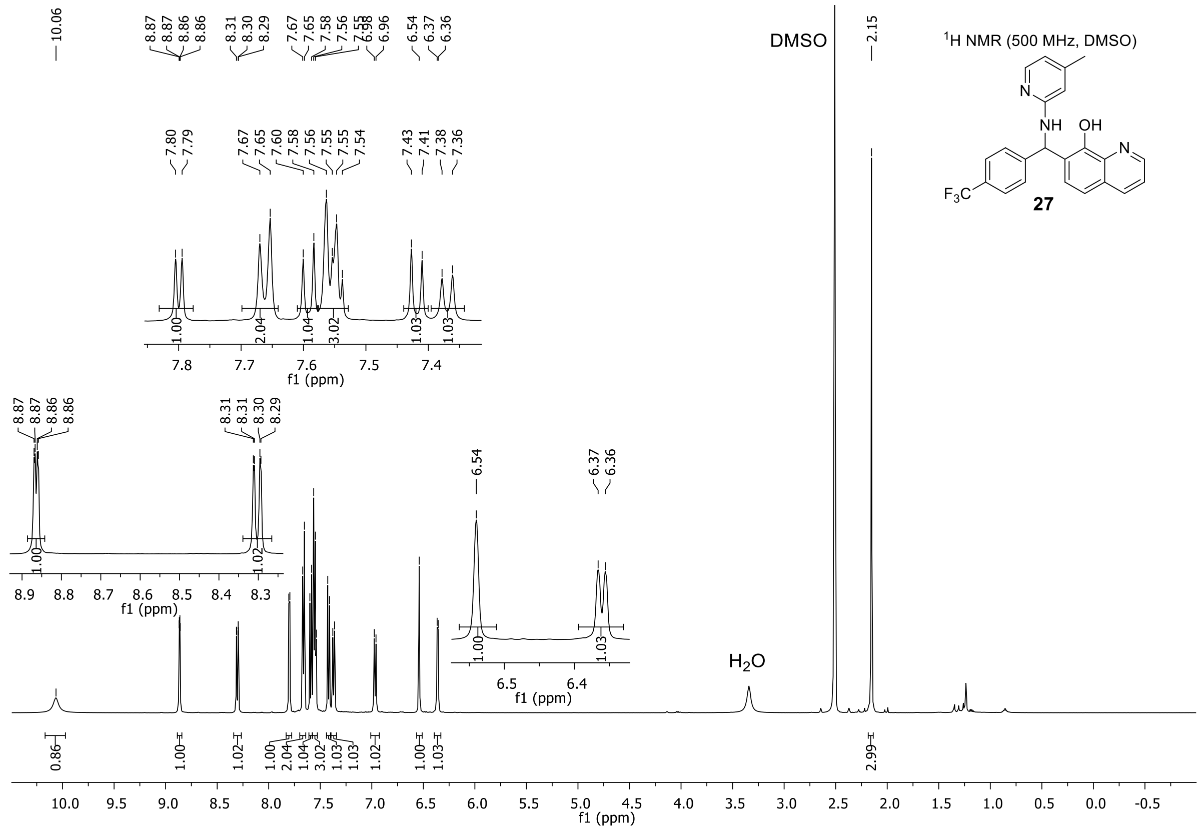
DMSO
^{1}H NMR (500 MHz, DMSO)
27
H_2O
f1 (ppm)

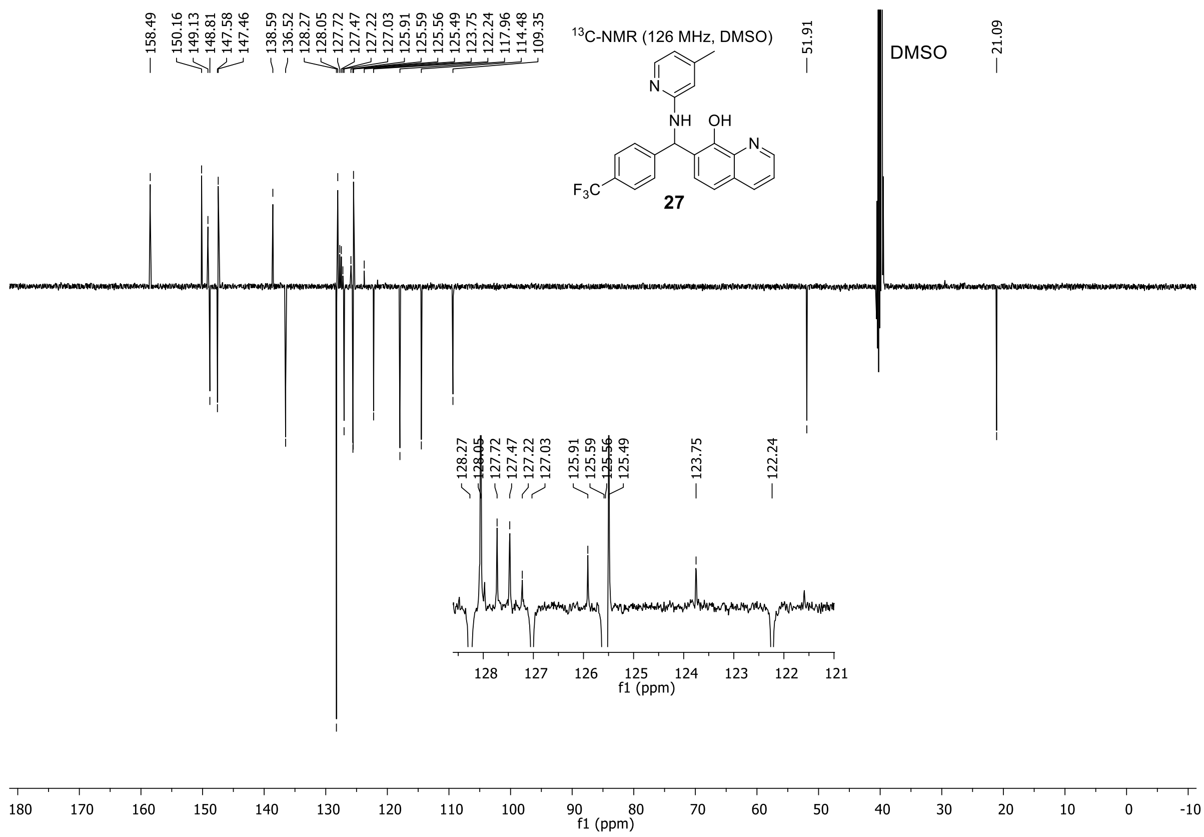
158.49
150.16
149.13
148.81
147.58
147.46
138.59
136.52
128.27
128.05
127.72
127.47
127.22
127.03
125.91
125.59
125.56
125.49
123.75
122.24
117.96
114.48
109.35
13C-NMR (126 MHz, DMSO)
51.91
DMSO
21.09
N
NH
OH
F3C
27
f1 (ppm)

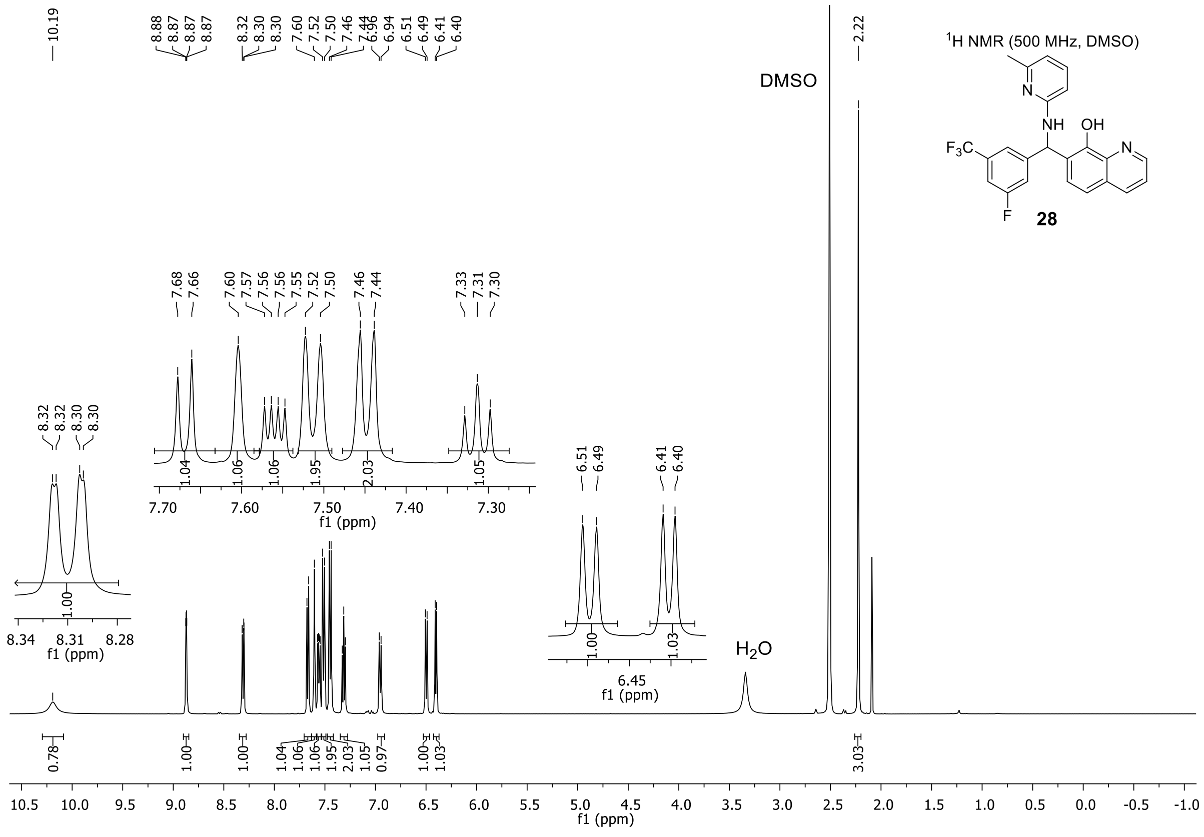
10.19
8.88
8.87
8.87
8.87
8.32
8.30
8.30
7.60
7.52
7.50
7.46
7.44
6.96
6.94
6.51
6.49
6.41
6.40
2.22
DMSO
1H NMR (500 MHz, DMSO)
N
NH
OH
F3C
F
28
H2O
f1 (ppm)

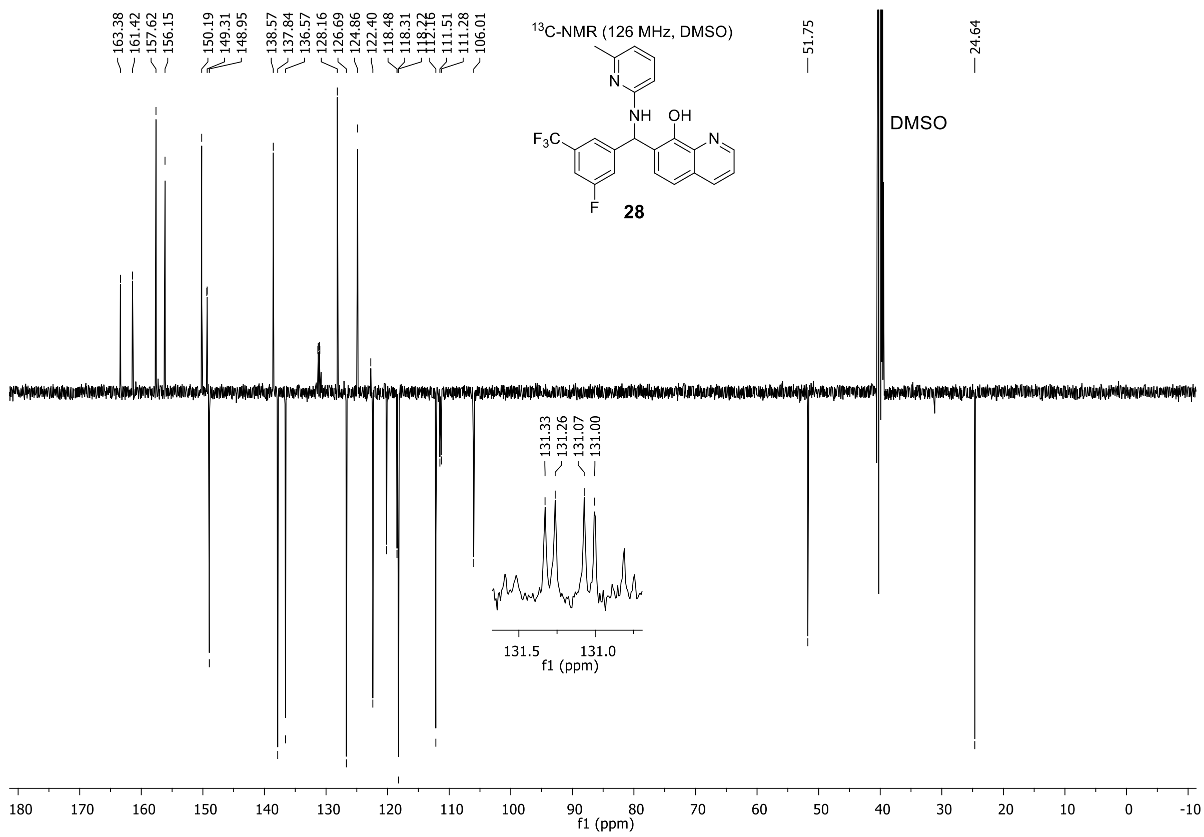
13C-NMR (126 MHz, DMSO)
163.38
161.42
157.62
156.15
150.19
149.31
148.95
138.57
137.84
136.57
128.16
126.69
124.86
122.40
118.48
118.31
118.22
118.16
111.51
111.28
106.01
51.75
24.64
DMSO
28
131.33
131.26
131.07
131.00
131.5
131.0
f1 (ppm)
f1 (ppm)

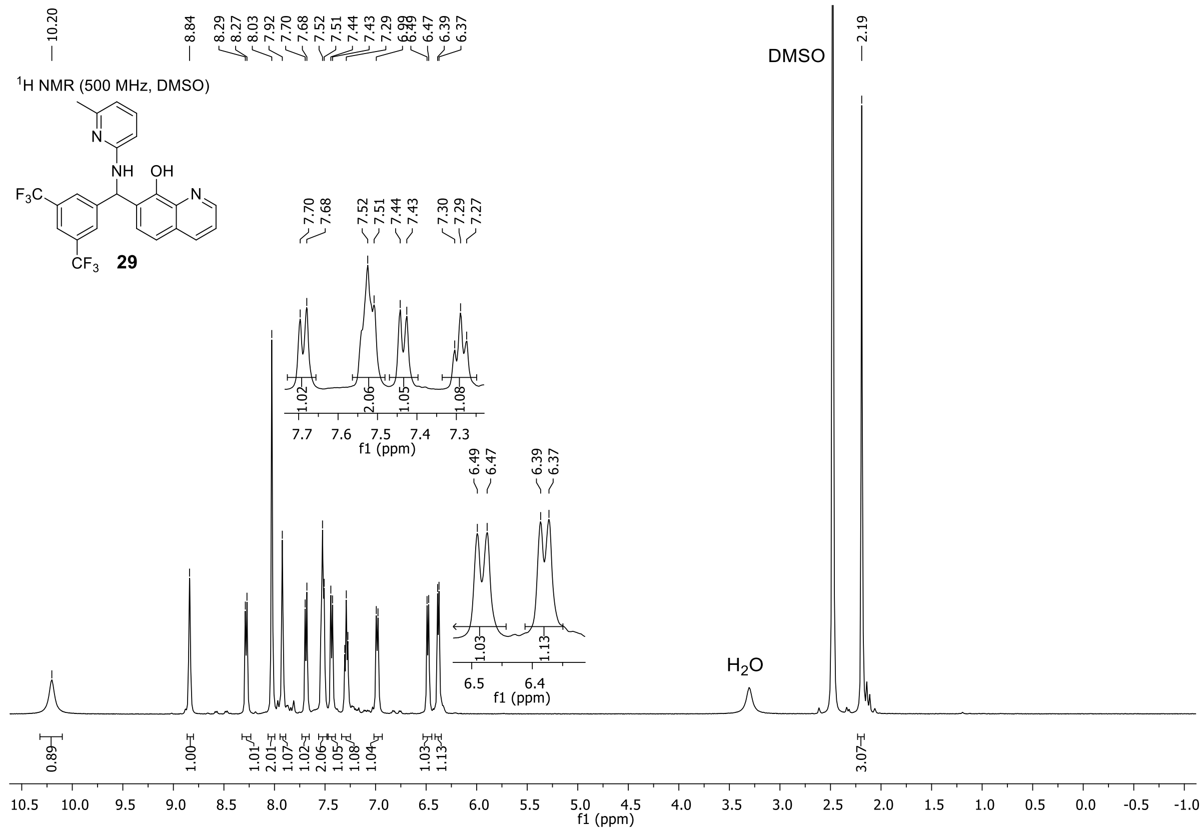
1H NMR (500 MHz, DMSO)
10.20
8.84
8.29
8.27
8.03
7.92
7.70
7.68
7.52
7.51
7.44
7.43
7.29
6.99
6.49
6.47
6.39
6.37
2.19
DMSO
29
H2O
0.89
1.00
1.01
2.01
1.07
1.02
2.06
1.05
1.08
1.04
1.03
1.13
3.07
f1 (ppm)

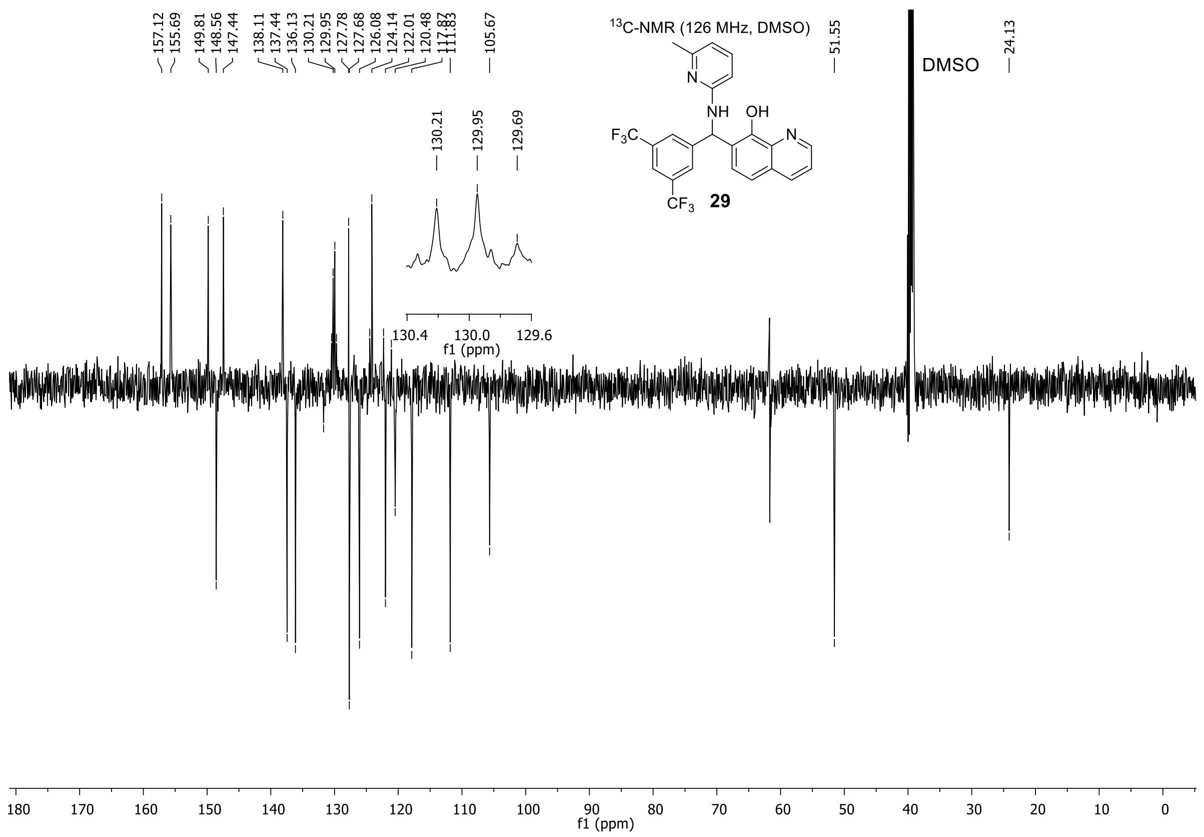

157.12
155.69
149.81
148.56
147.44
138.11
137.44
136.13
130.21
129.95
127.78
127.68
126.08
124.14
122.01
120.48
117.82
111.83
105.67
^{13}C-NMR (126 MHz, DMSO)
51.55
DMSO
24.13
130.21
129.95
129.69
130.4
130.0
129.6
f1 (ppm)
F_3C
CF_3
NH
OH
N
29
180
170
160
150
140
130
120
110
100
90
80
70
60
50
40
30
20
10
0
f1 (ppm)

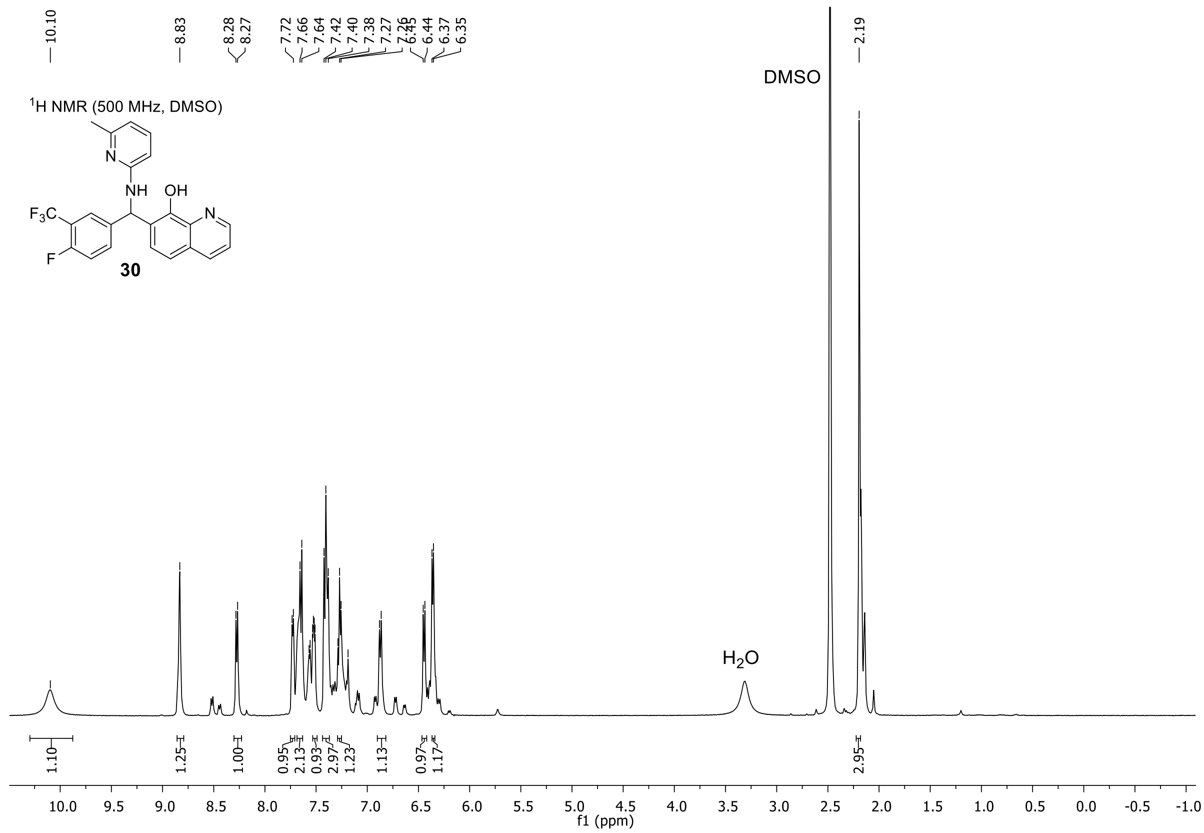

10.10
8.83
8.28
8.27
7.72
7.66
7.64
7.42
7.40
7.38
7.27
6.45
6.44
6.37
6.35
2.19
^{1}H NMR (500 MHz, DMSO)
DMSO
F_3C
F
NH
OH
N
30
H_2O
1.10
1.25
1.00
0.95
2.13
0.93
2.97
1.23
1.13
0.97
1.17
2.95
10.0
9.5
9.0
8.5
8.0
7.5
7.0
6.5
6.0
5.5
5.0
4.5
4.0
3.5
3.0
2.5
2.0
1.5
1.0
0.5
0.0
-0.5
-1.0
f1 (ppm)

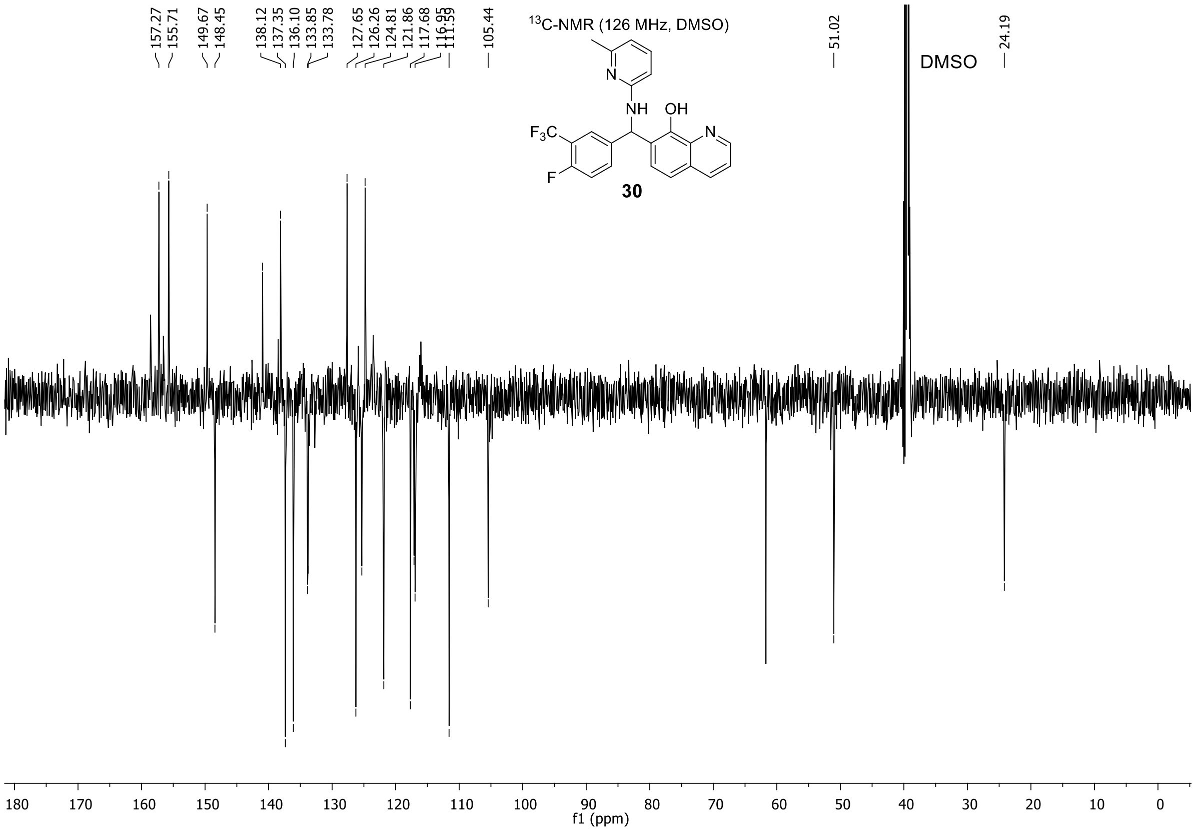
157.27
155.71
149.67
148.45
138.12
137.35
136.10
133.85
133.78
127.65
126.26
124.81
121.86
117.68
116.95
111.59
105.44
^{13}C-NMR (126 MHz, DMSO)
N
NH OH
N
F_3C
F
30
51.02
DMSO
24.19
180 170 160 150 140 130 120 110 100 90 80 70 60 50 40 30 20 10 0
f1 (ppm)

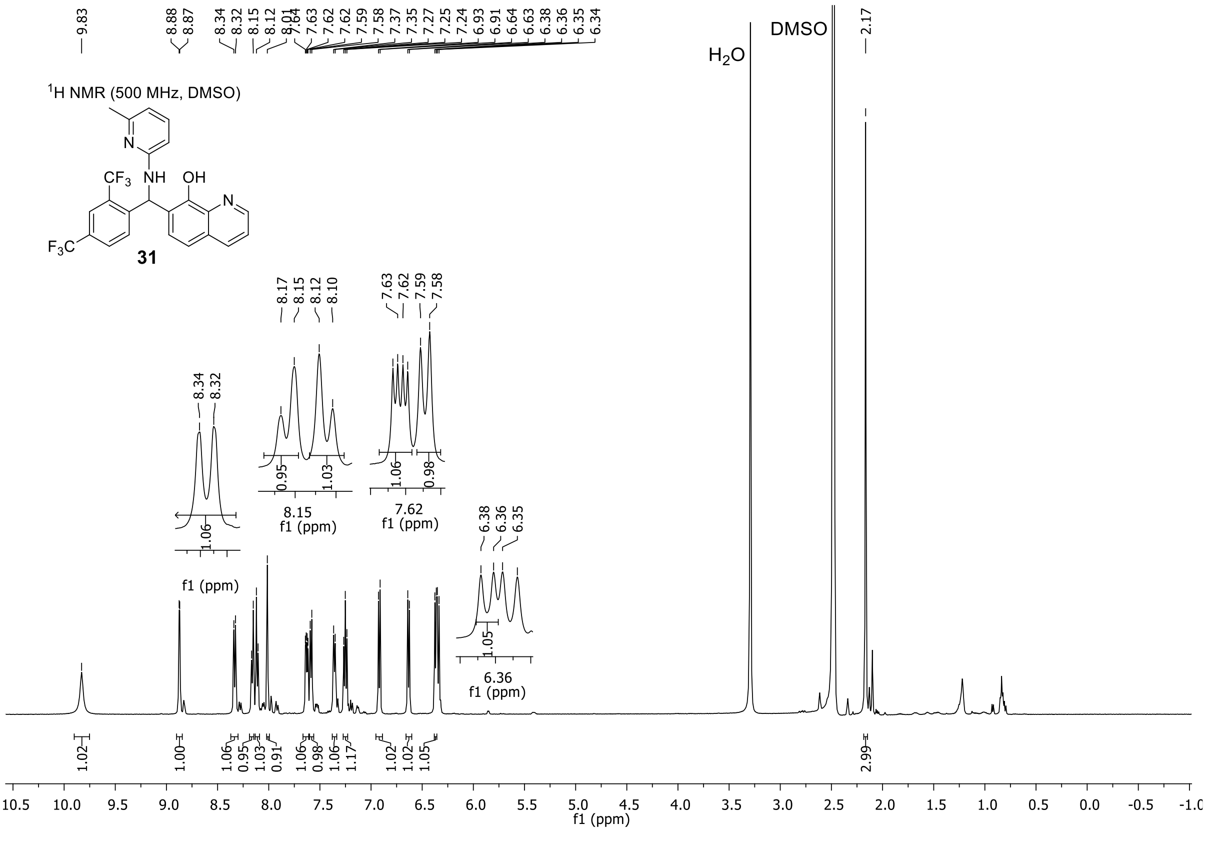
9.83
8.88
8.87
8.34
8.32
8.15
8.12
8.01
7.64
7.63
7.62
7.62
7.59
7.58
7.37
7.35
7.27
7.25
7.24
6.93
6.91
6.64
6.63
6.38
6.36
6.35
6.34
H_2O
DMSO
2.17
^{1}H NMR (500 MHz, DMSO)
N
CF_3 NH OH
N
F_3C
31
8.17
8.15
8.12
8.10
7.63
7.62
7.59
7.58
8.34
8.32
0.95
1.03
1.06
0.98
8.15
f1 (ppm)
7.62
f1 (ppm)
1.06
f1 (ppm)
6.38
6.36
6.35
1.05
6.36
f1 (ppm)
1.02
1.00
1.06
0.95
1.03
0.91
1.06
0.98
1.06
1.17
1.02
1.02
1.05
2.99
10.5 10.0 9.5 9.0 8.5 8.0 7.5 7.0 6.5 6.0 5.5 5.0 4.5 4.0 3.5 3.0 2.5 2.0 1.5 1.0 0.5 0.0 -0.5 -1.0
f1 (ppm)

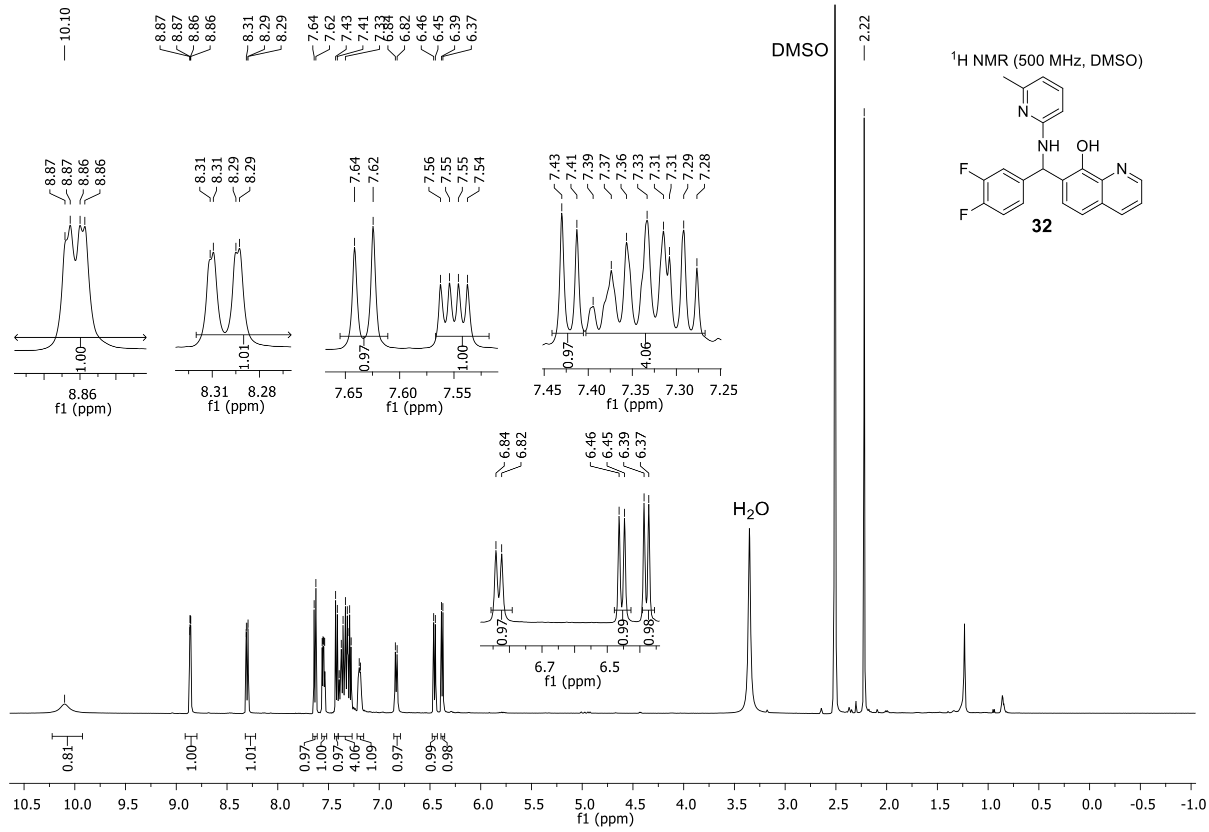
1H NMR (500 MHz, DMSO)
DMSO
H2O
32
f1 (ppm)

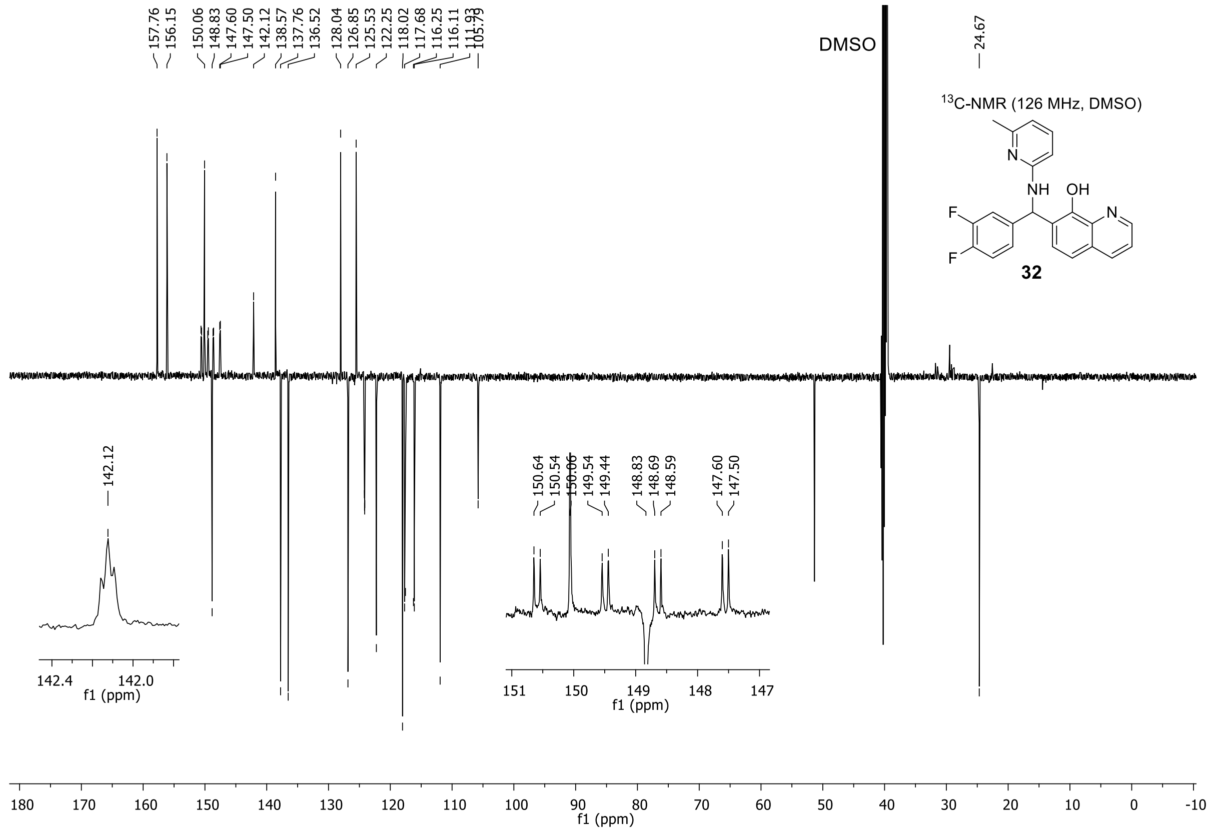
13C-NMR (126 MHz, DMSO)
DMSO
32
f1 (ppm)

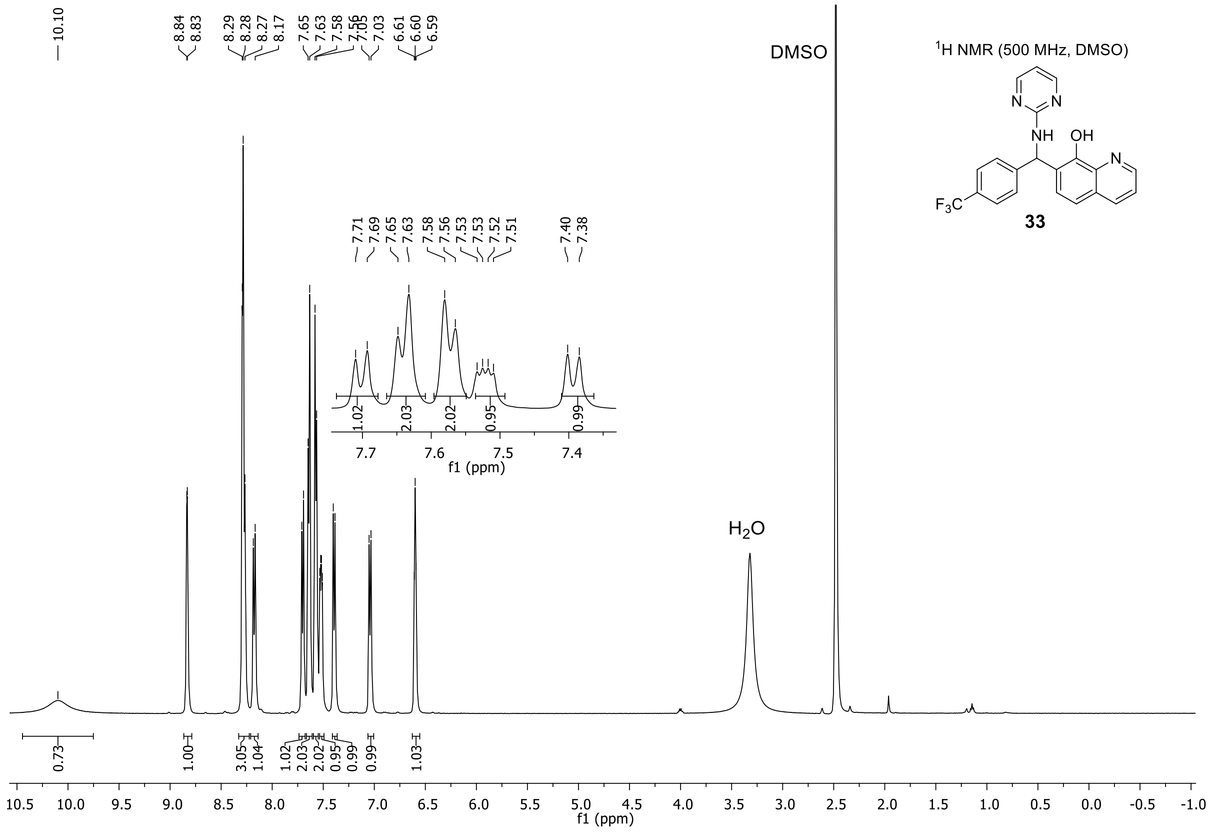

DMSO
^{1}H NMR (500 MHz, DMSO)
H$_2$O
NH OH
F$_3$C
33
f1 (ppm)

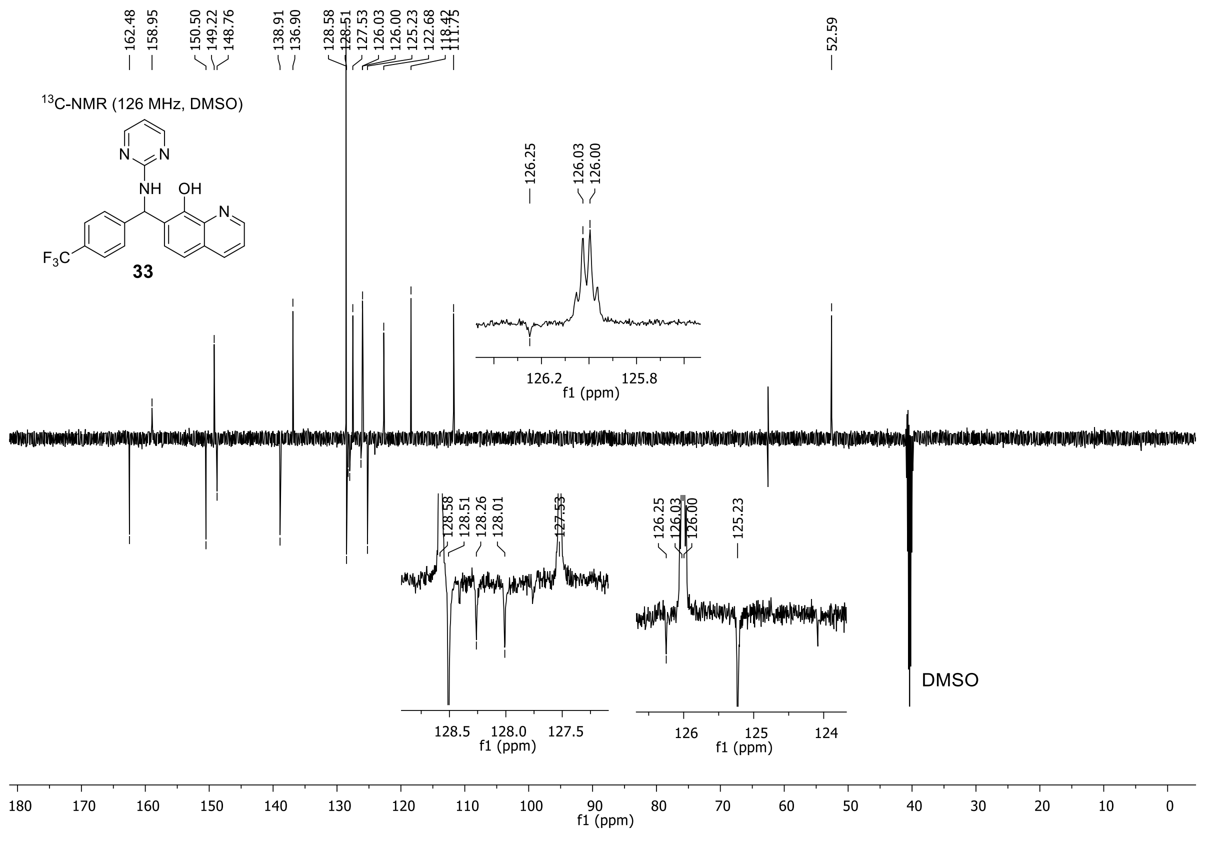

^{13}C-NMR (126 MHz, DMSO)
NH OH
F$_3$C
33
DMSO
f1 (ppm)

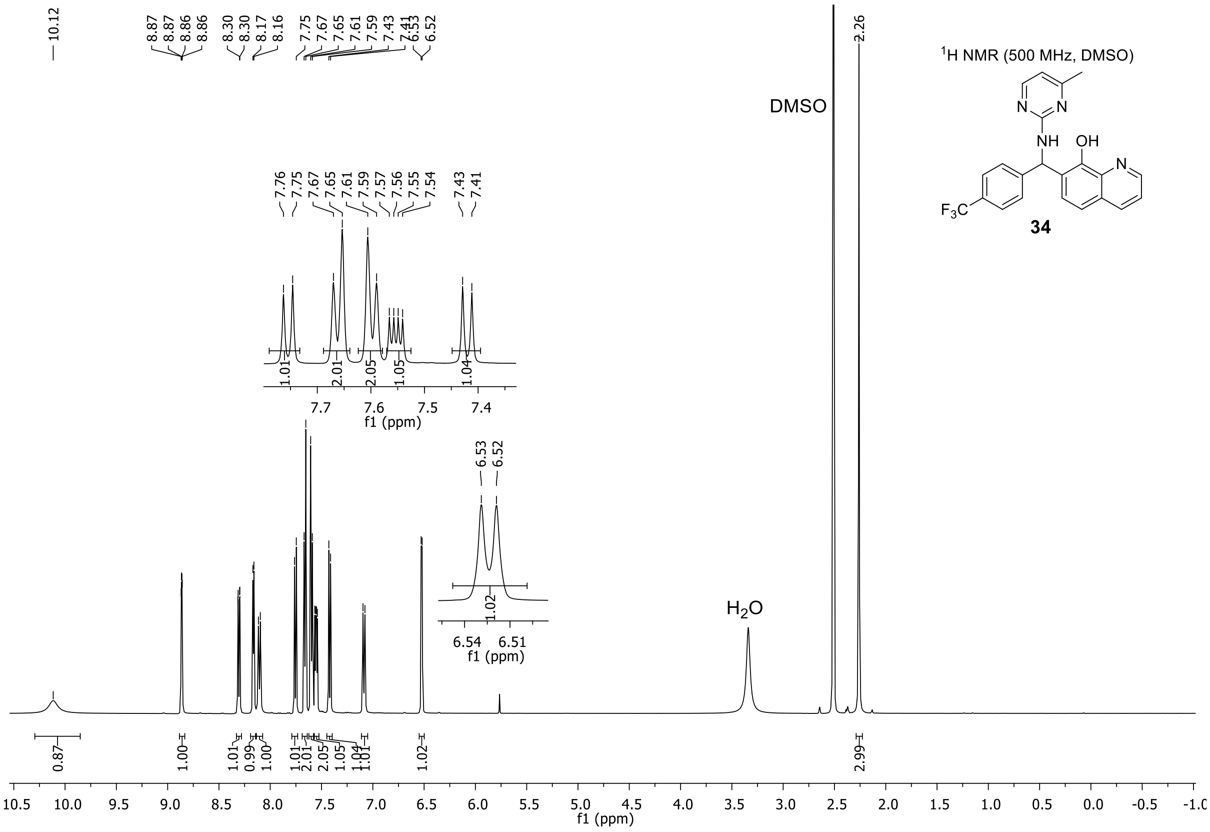
^{1}H NMR (500 MHz, DMSO)
34
DMSO
H2O
f1 (ppm)

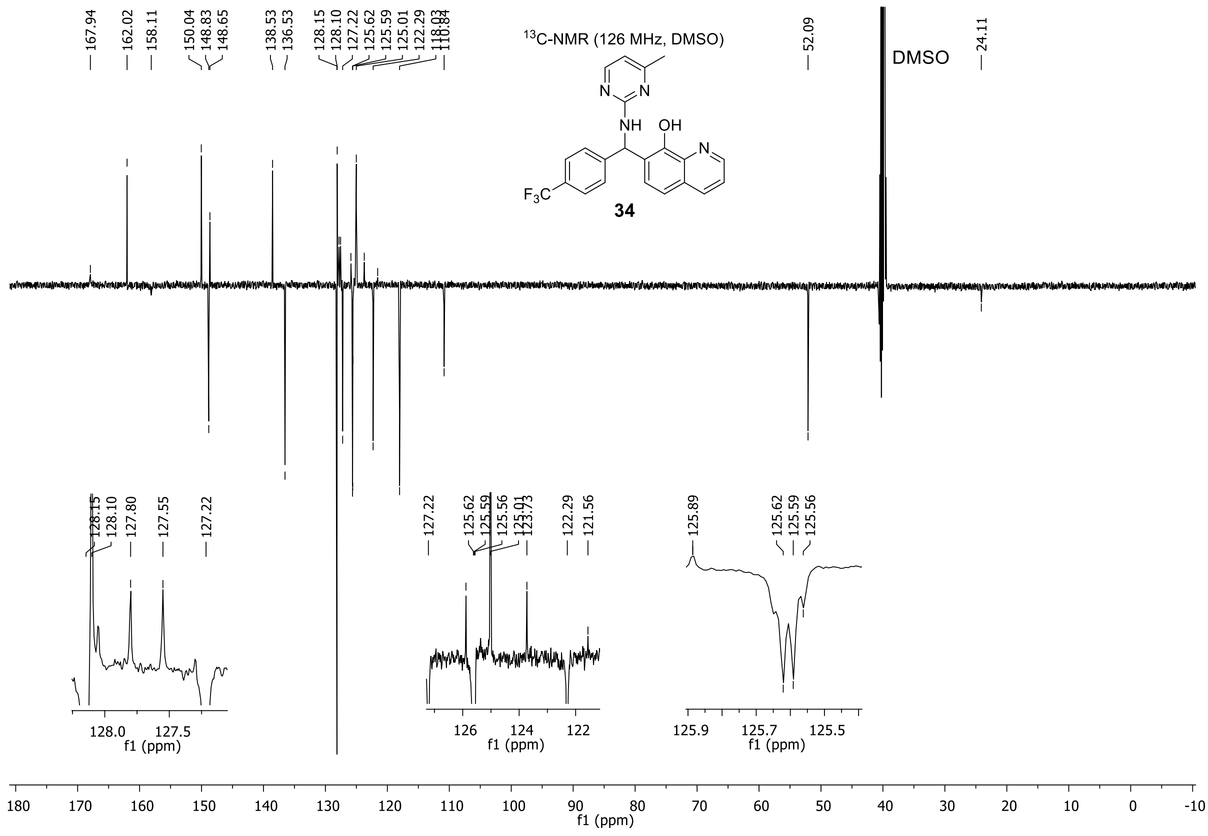
^{13}C-NMR (126 MHz, DMSO)
34
DMSO
f1 (ppm)

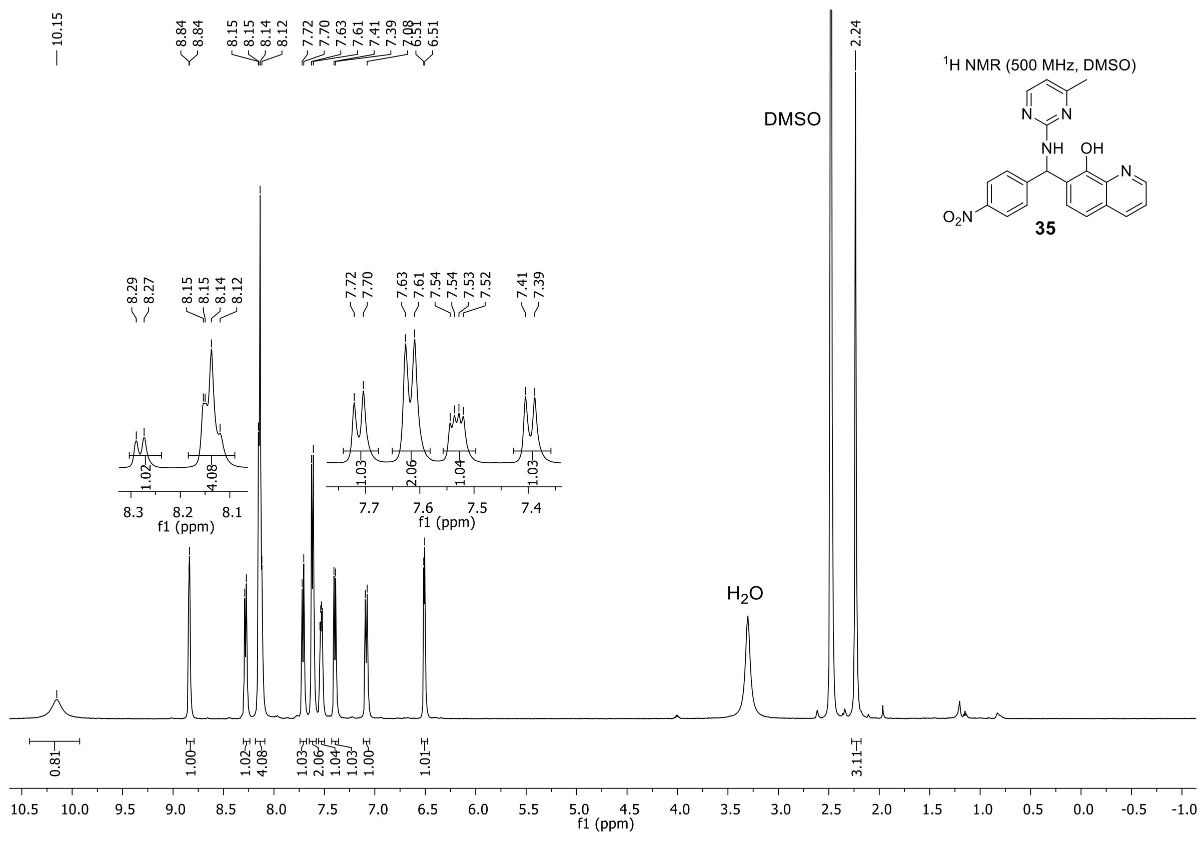

^{1}H NMR (500 MHz, DMSO)
DMSO
H2O
35
f1 (ppm)

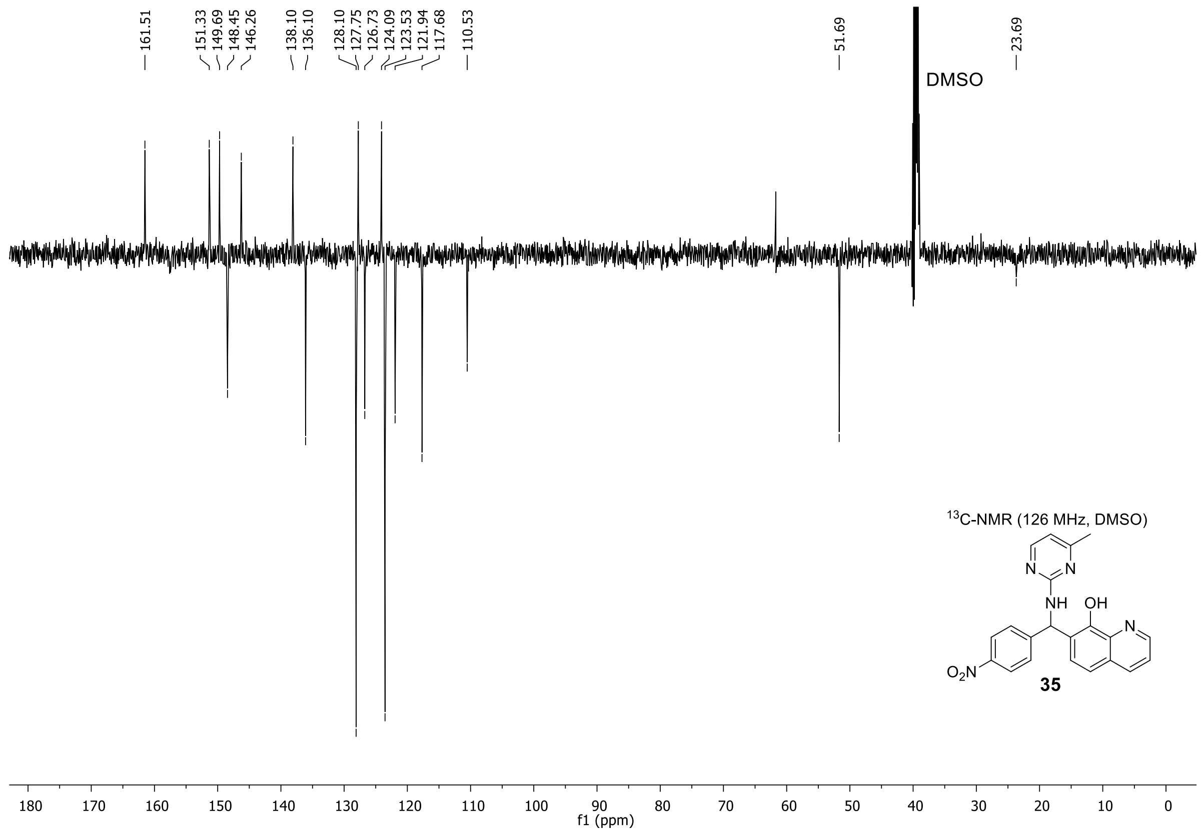

^{13}C-NMR (126 MHz, DMSO)
DMSO
35
f1 (ppm)

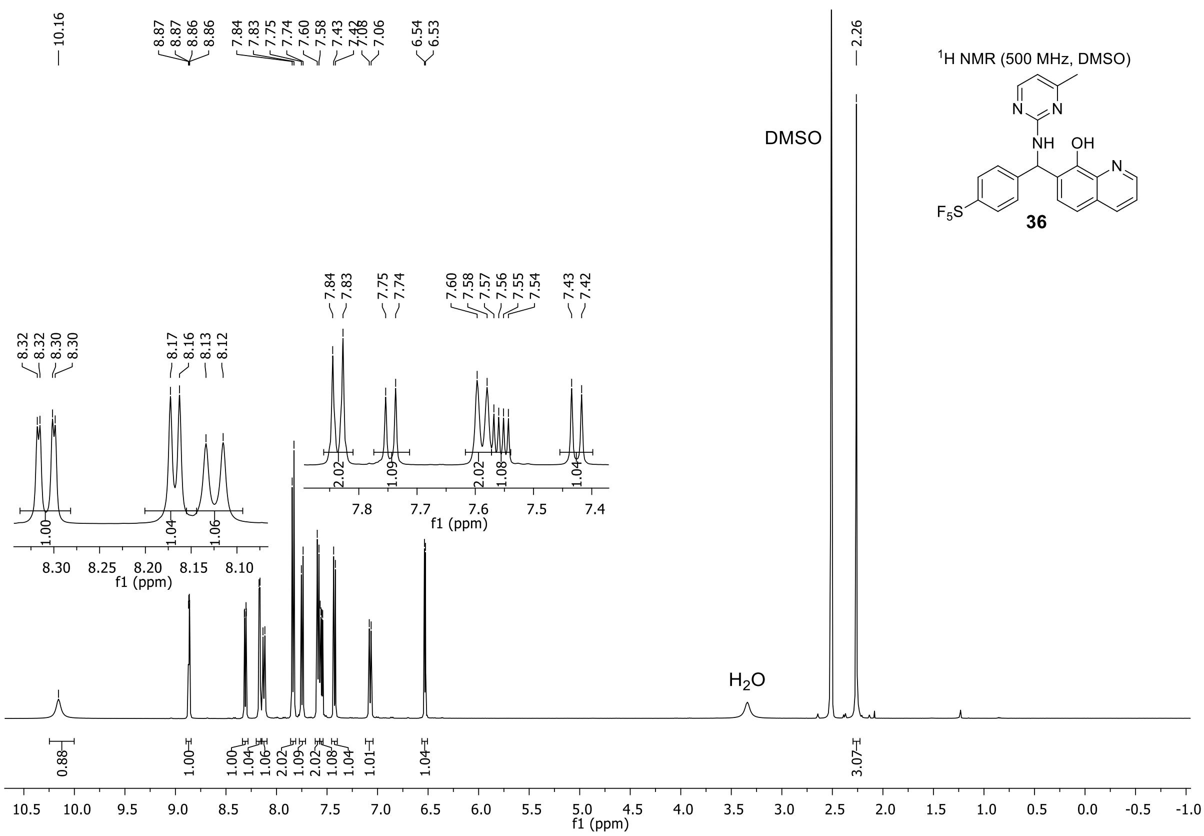
1H NMR (500 MHz, DMSO)
36
F5S
NH OH
DMSO
H2O
10.16
8.87 8.87 8.86 8.86
7.84 7.83 7.75 7.74 7.60 7.58 7.43 7.42 7.08 7.06
6.54 6.53
2.26
8.32 8.32 8.30 8.30
8.17 8.16 8.13 8.12
7.84 7.83 7.75 7.74 7.60 7.58 7.57 7.56 7.55 7.54 7.43 7.42
f1 (ppm)

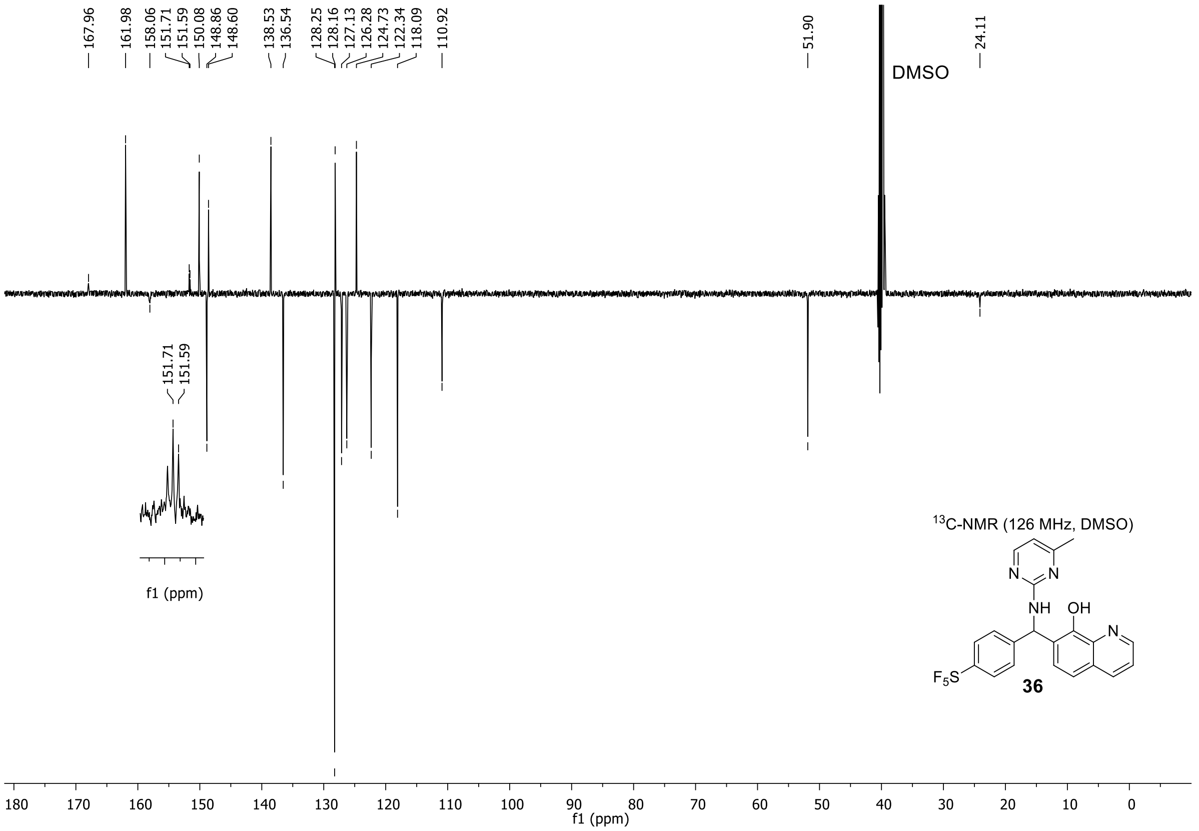
167.96 161.98 158.06 151.71 151.59 150.08 148.86 148.60 138.53 136.54 128.25 128.16 127.13 126.28 124.73 122.34 118.09 110.92
51.90
24.11
DMSO
151.71 151.59
13C-NMR (126 MHz, DMSO)
36
F5S
NH OH
f1 (ppm)

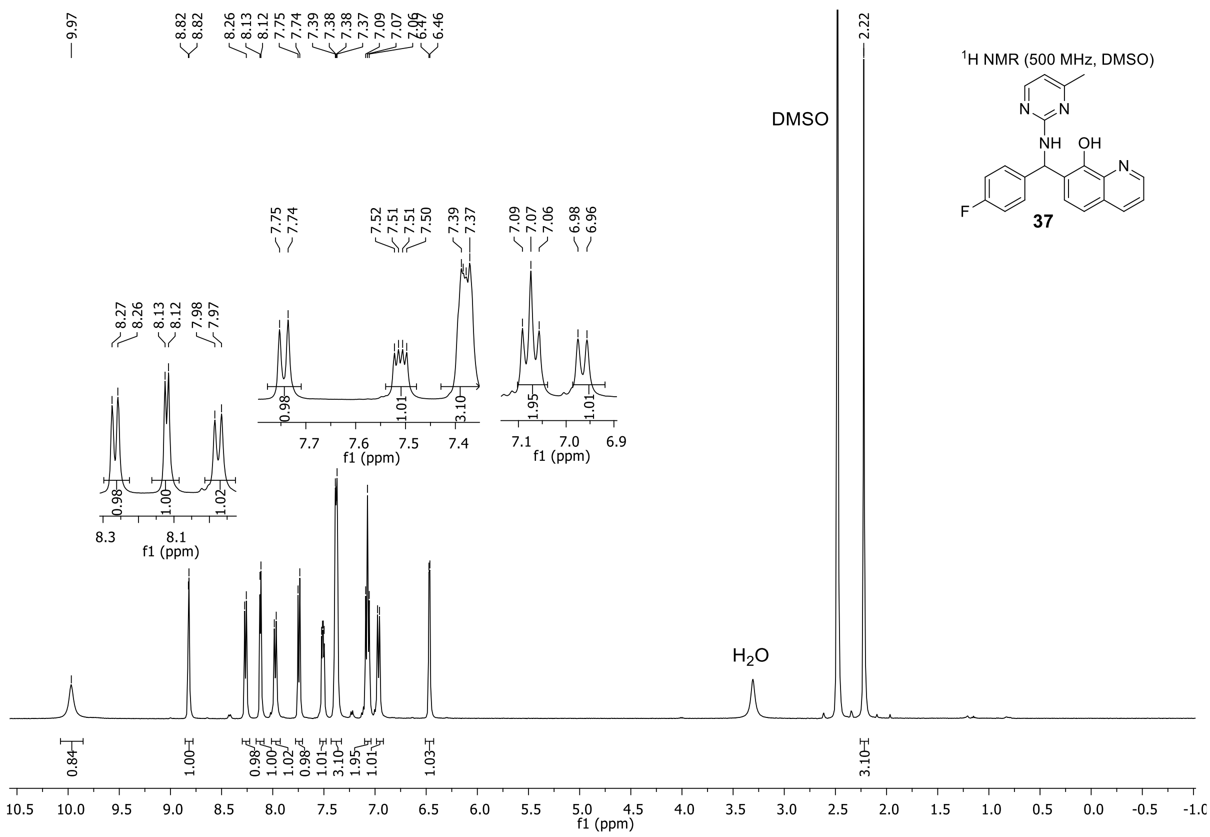
^{1}H NMR (500 MHz, DMSO)
DMSO
H_2O
37
f1 (ppm)

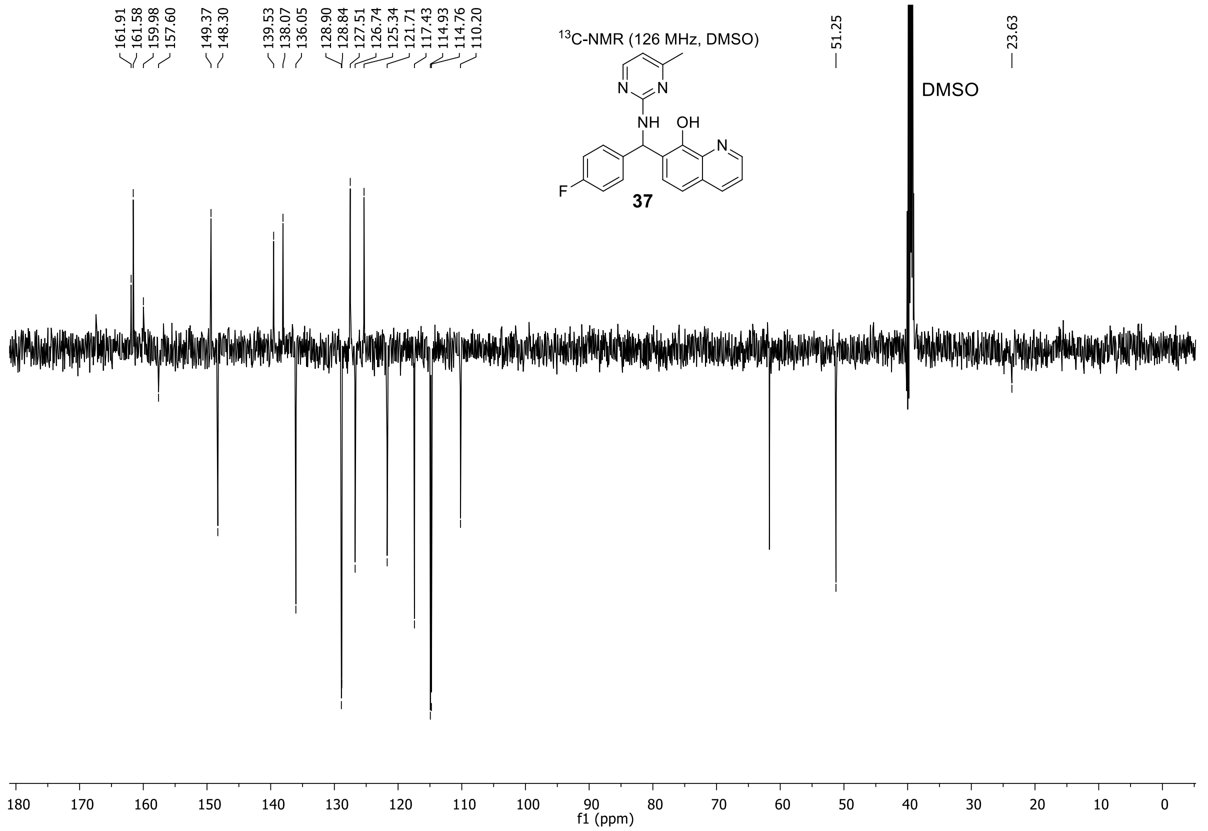
^{13}C-NMR (126 MHz, DMSO)
DMSO
37
f1 (ppm)

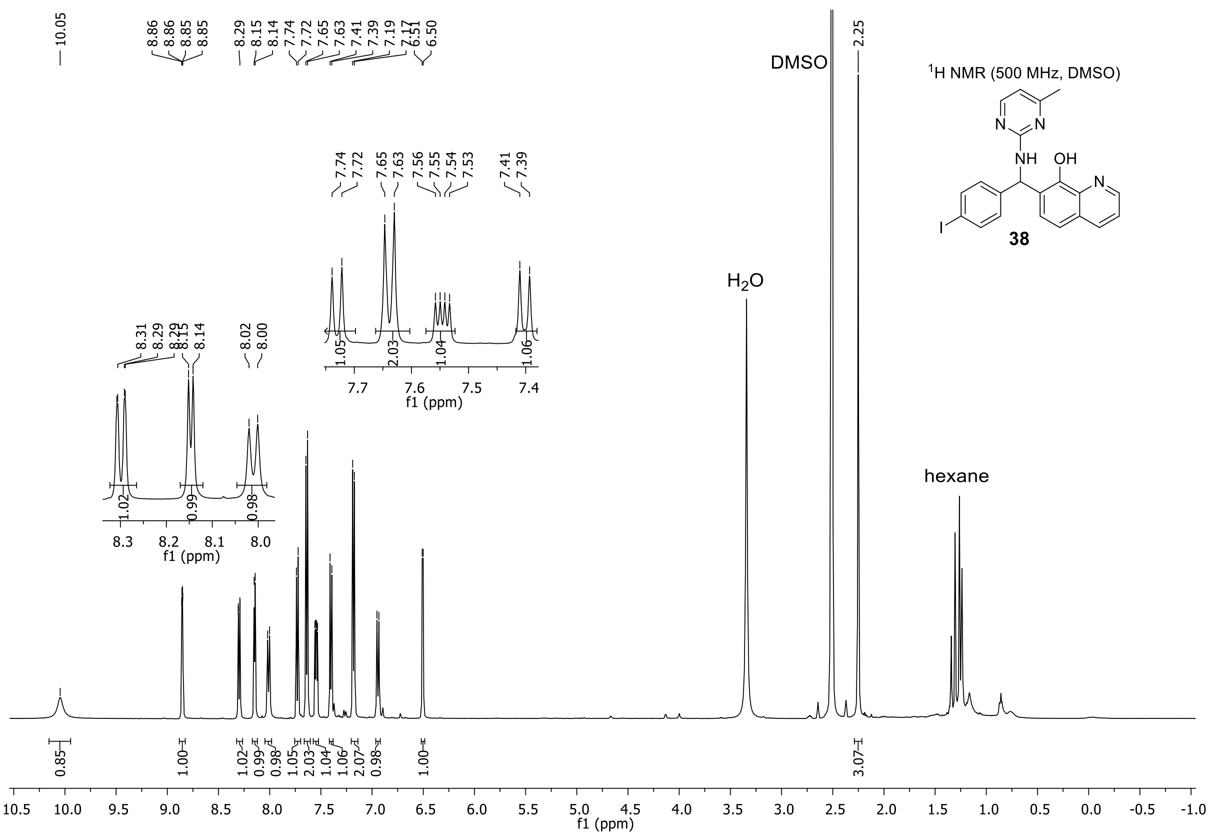
10.05
8.86 8.86 8.85 8.85
8.29 8.15 8.14 7.74 7.72 7.65 7.63 7.41 7.39 7.19 7.17 6.51 6.50
DMSO
2.25
1H NMR (500 MHz, DMSO)
N
N
NH
OH
I
38
H2O
hexane
f1 (ppm)

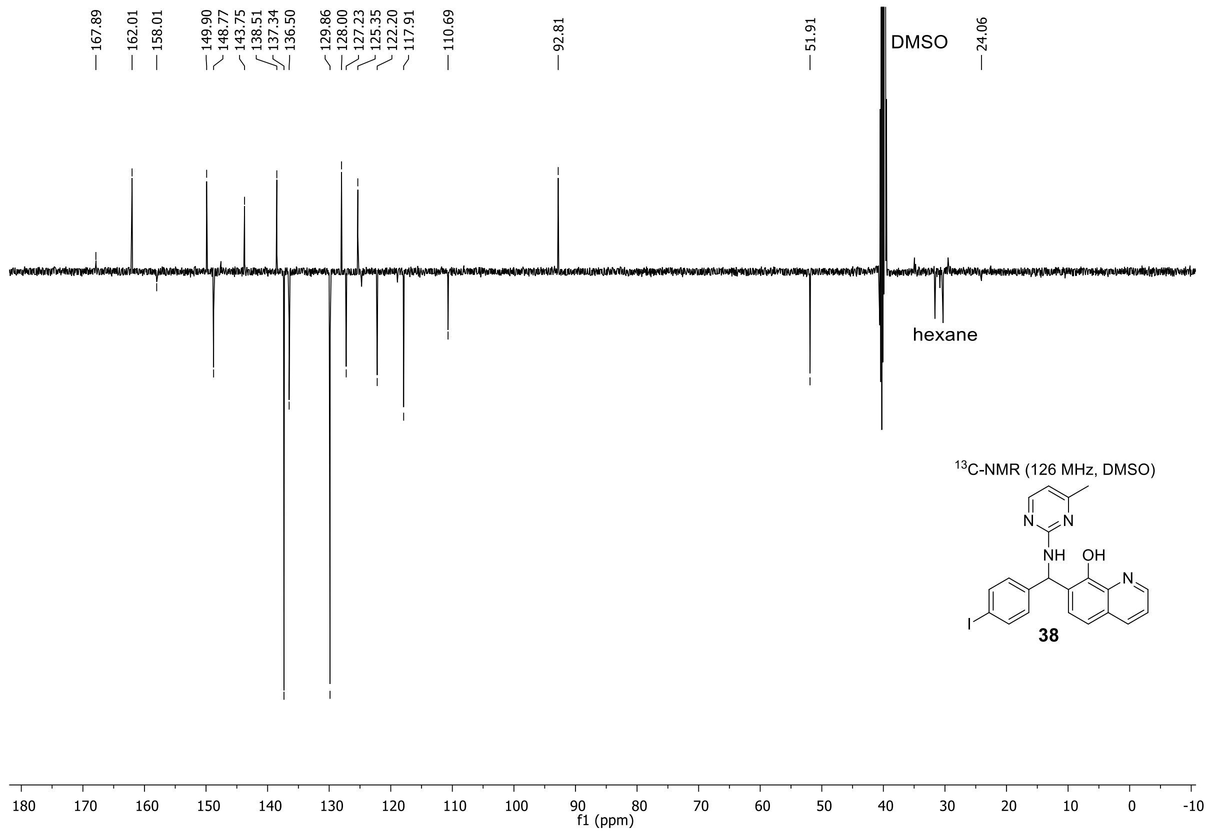
167.89 162.01 158.01 149.90 148.77 143.75 138.51 137.34 136.50 129.86 128.00 127.23 125.35 122.20 117.91 110.69 92.81 51.91 24.06
DMSO
hexane
13C-NMR (126 MHz, DMSO)
NH
OH
I
38
f1 (ppm)

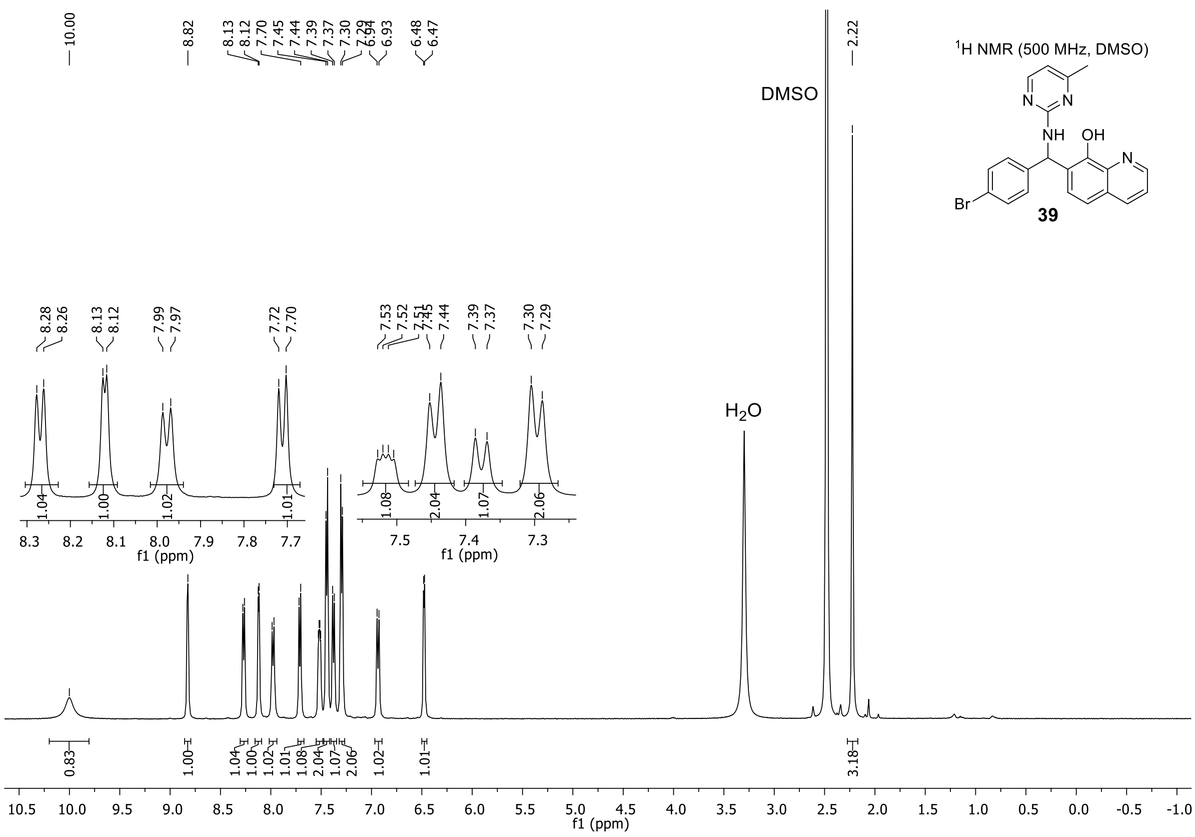

1H NMR (500 MHz, DMSO)
DMSO
H2O
39
Br
NH OH
f1 (ppm)

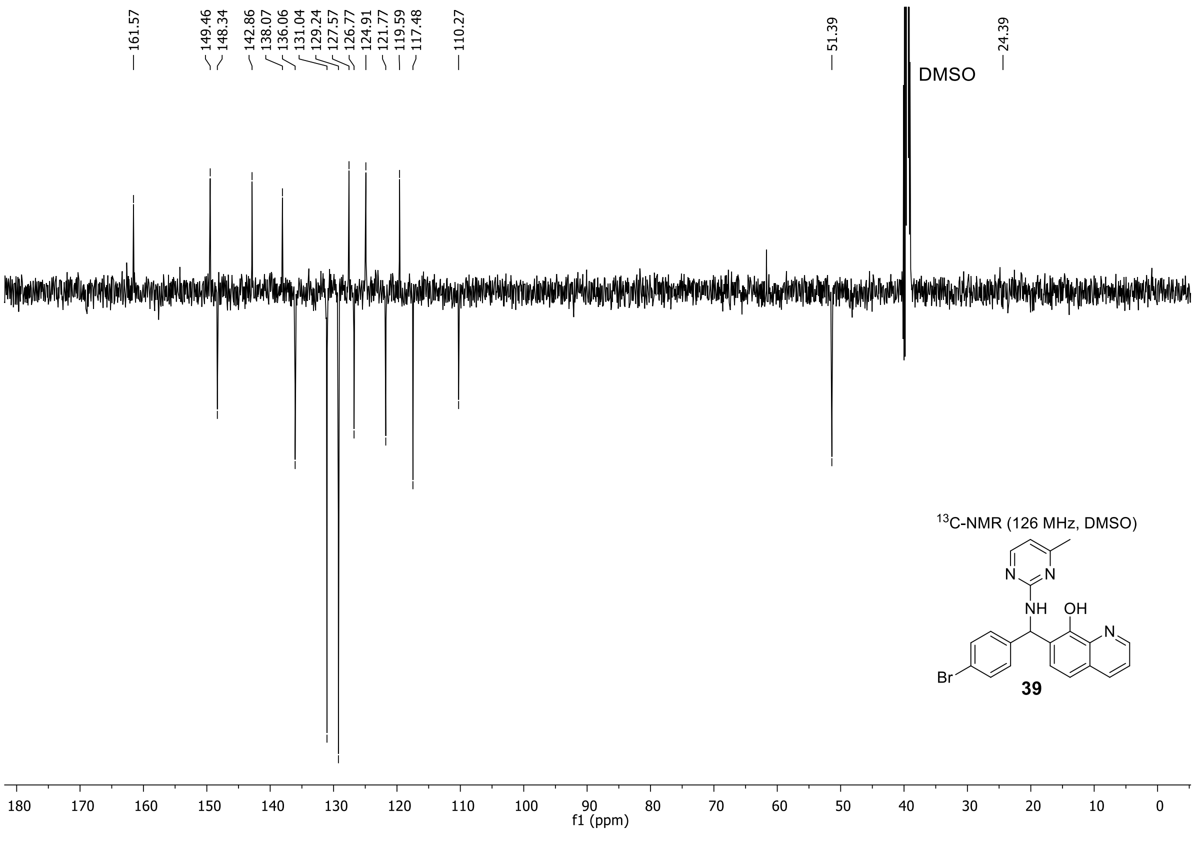

13C-NMR (126 MHz, DMSO)
DMSO
39
Br
NH OH
f1 (ppm)

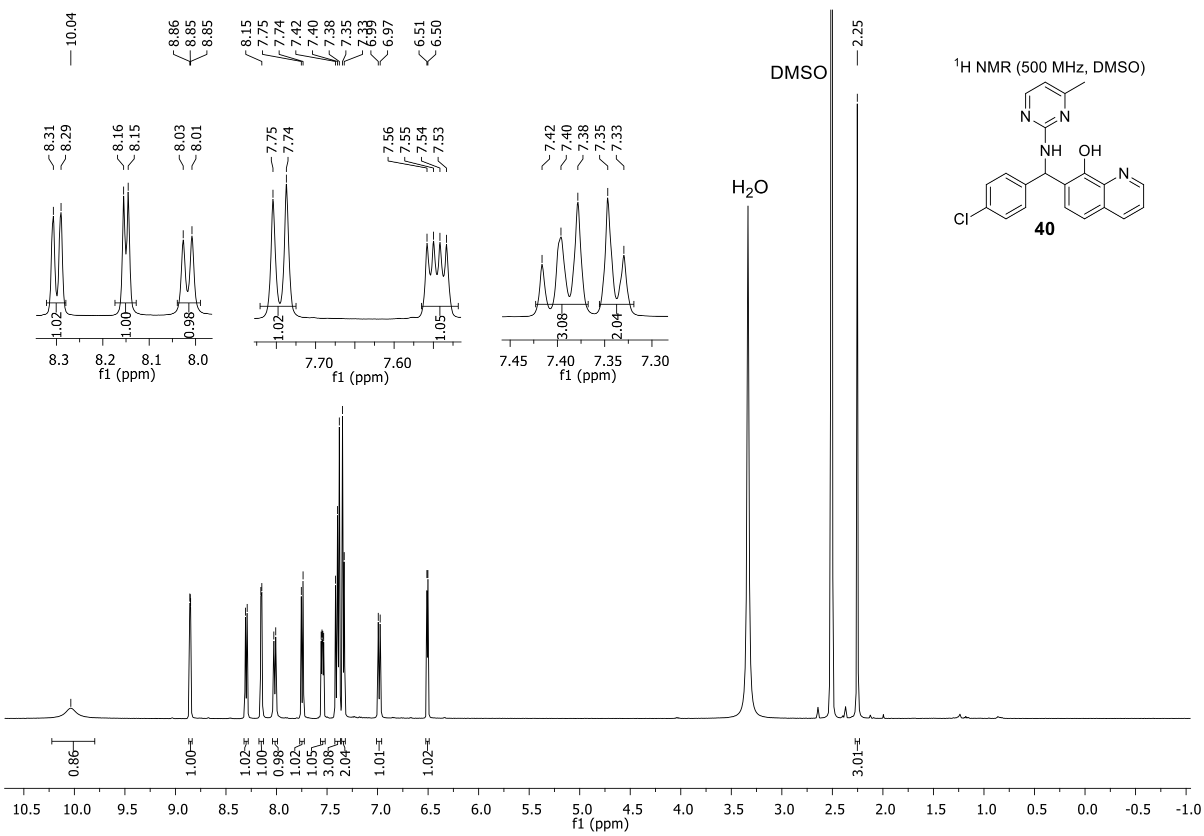

10.04
8.86 8.85 8.85
8.15 7.75 7.74 7.42 7.40 7.38 7.35 7.33 6.99 6.97
6.51 6.50
DMSO
2.25
¹H NMR (500 MHz, DMSO)
N N
NH OH
N
Cl
40
8.31 8.29 8.16 8.15 8.03 8.01
7.75 7.74 7.56 7.55 7.54 7.53
7.42 7.40 7.38 7.35 7.33
H2O
1.02 1.00 0.98
1.02 1.05
3.08 2.04
f1 (ppm)
0.86 1.00 1.02 1.00 0.98 1.02 1.05 3.08 2.04 1.01 1.02 3.01
10.5 10.0 9.5 9.0 8.5 8.0 7.5 7.0 6.5 6.0 5.5 5.0 4.5 4.0 3.5 3.0 2.5 2.0 1.5 1.0 0.5 0.0 -0.5 -1.0
f1 (ppm)

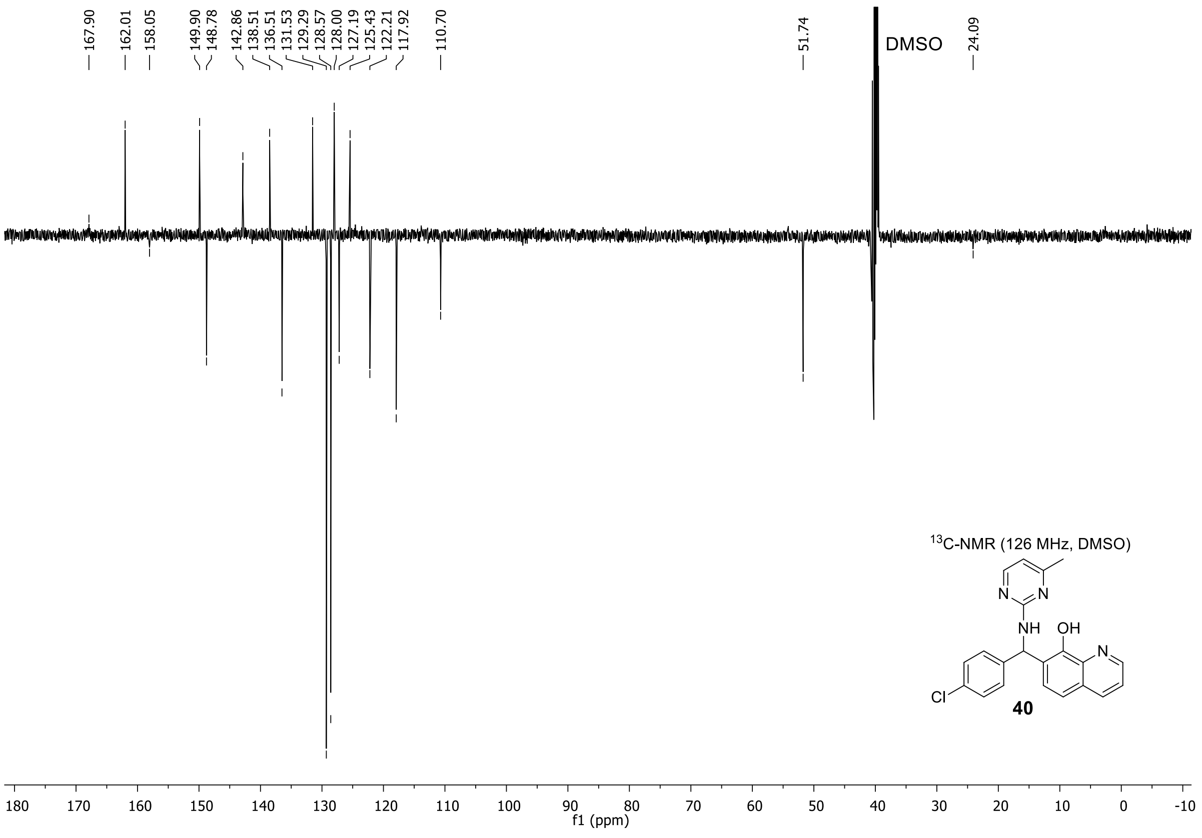

167.90 162.01 158.05 149.90 148.78 142.86 138.51 136.51 131.53 129.29 128.57 128.00 127.19 125.43 122.21 117.92 110.70
51.74
DMSO
24.09
¹³C-NMR (126 MHz, DMSO)
N N
NH OH
N
Cl
40
180 170 160 150 140 130 120 110 100 90 80 70 60 50 40 30 20 10 0 -10
f1 (ppm)

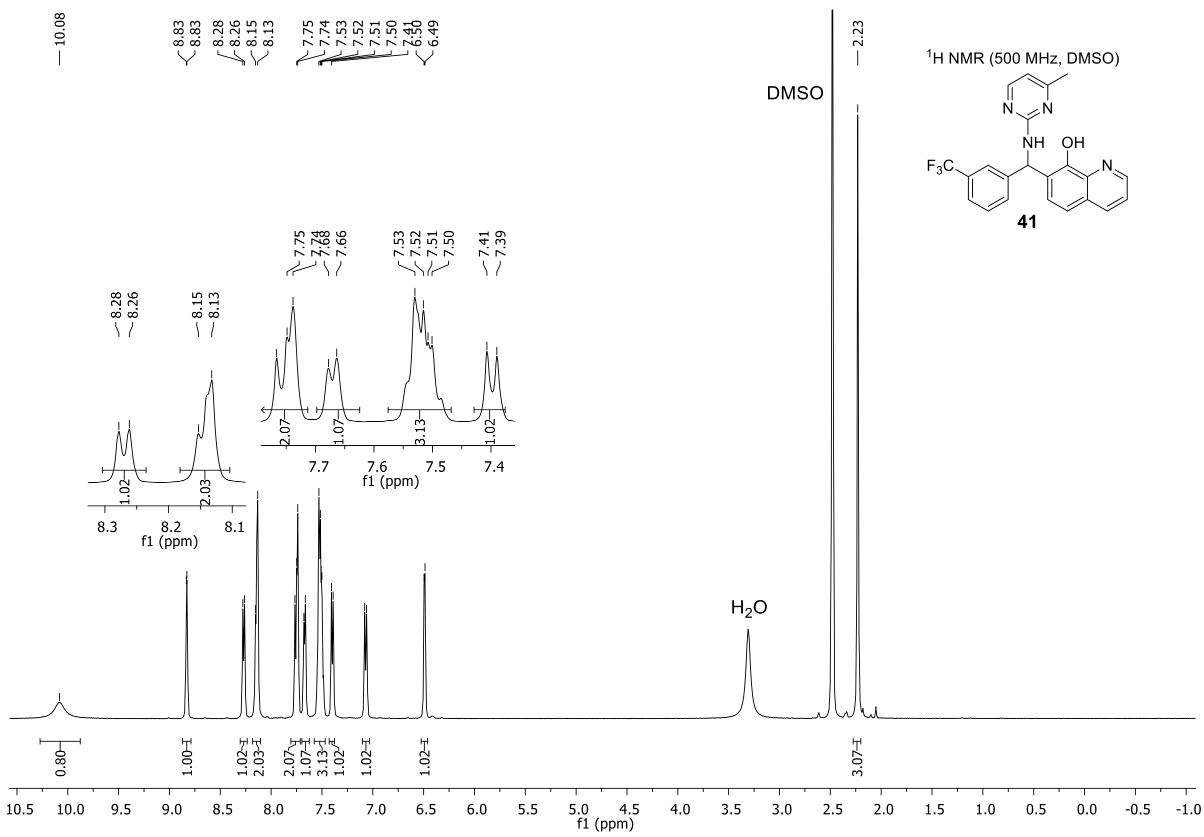

10.08
8.83
8.83
8.28
8.26
8.15
8.13
7.75
7.74
7.53
7.52
7.51
7.50
7.41
6.50
6.49
2.23
DMSO
H2O
1H NMR (500 MHz, DMSO)
N
N
NH
OH
F3C
N
41
0.80
1.00
1.02
2.03
2.07
1.07
3.13
1.02
1.02
1.02
3.07
f1 (ppm)

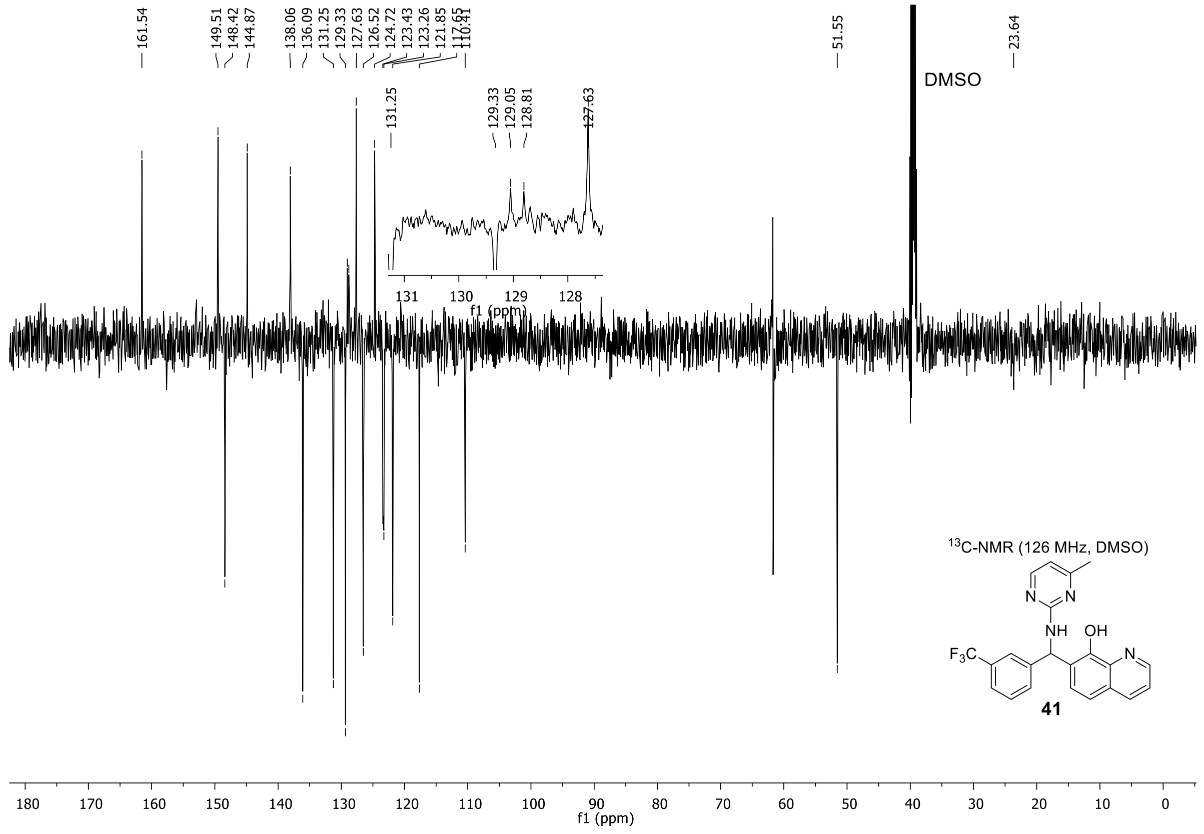

161.54
149.51
148.42
144.87
138.06
136.09
131.25
129.33
127.63
126.52
124.72
123.43
123.26
121.85
110.41
51.55
23.64
DMSO
131.25
129.33
129.05
128.81
127.63
13C-NMR (126 MHz, DMSO)
N
N
NH
OH
F3C
N
41
f1 (ppm)

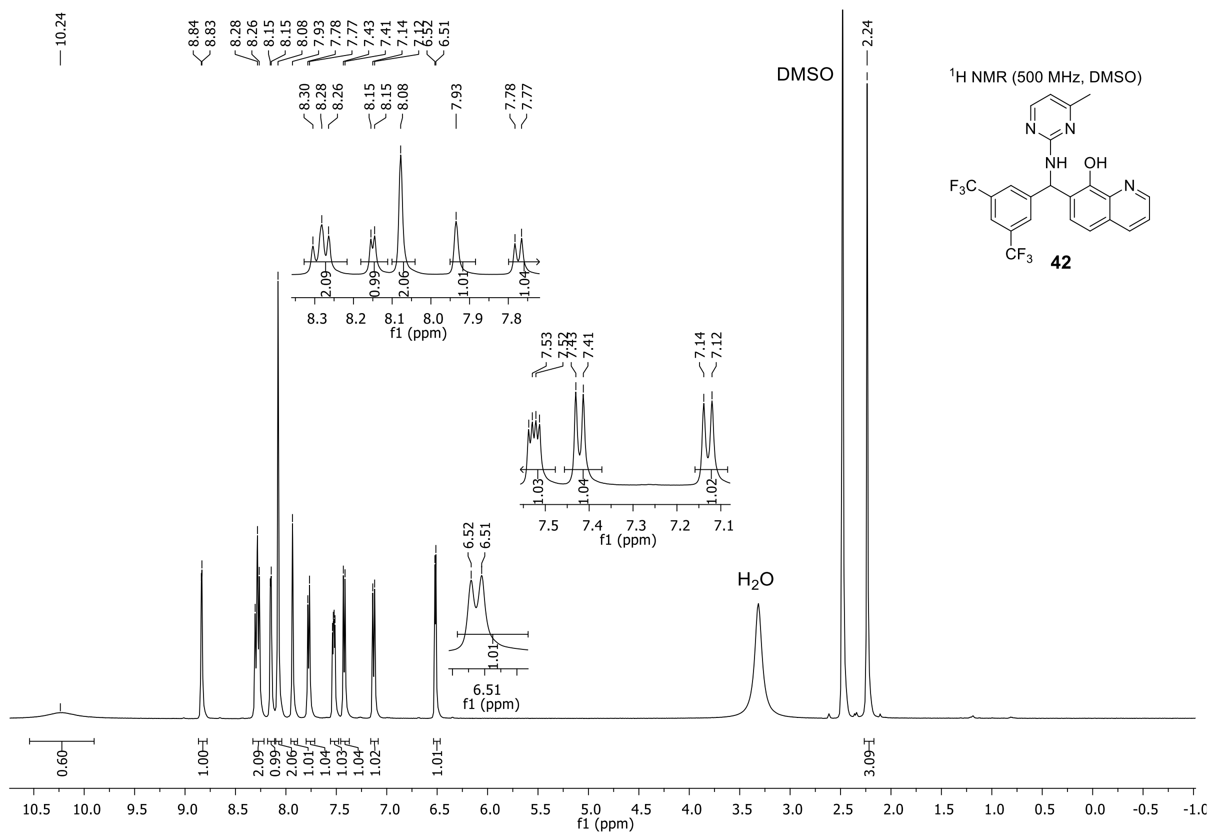
10.24
8.84
8.83
8.28
8.26
8.15
8.15
8.08
7.93
7.78
7.77
7.43
7.41
7.14
7.12
6.52
6.51
2.24
DMSO
^{1}H NMR (500 MHz, DMSO)
NH
OH
N
F_3C
CF_3
42
H_2O
0.60
1.00
2.09
0.99
2.06
1.01
1.04
1.03
1.04
1.02
1.01
3.09
f1 (ppm)

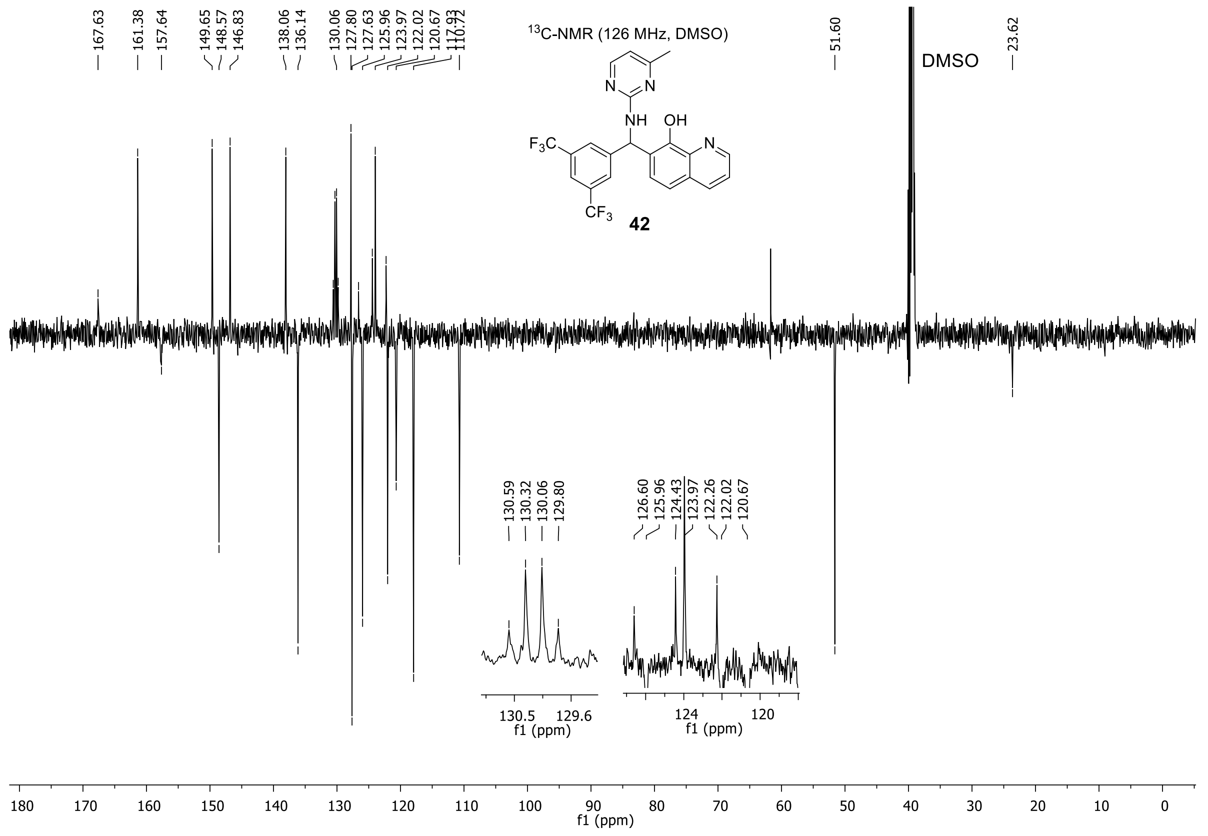
167.63
161.38
157.64
149.65
148.57
146.83
138.06
136.14
130.06
127.80
127.63
125.96
123.97
122.02
120.67
117.93
110.72
^{13}C-NMR (126 MHz, DMSO)
51.60
DMSO
23.62
NH
OH
F_3C
CF_3
42
130.59
130.32
130.06
129.80
126.60
125.96
124.43
123.97
122.26
122.02
120.67
f1 (ppm)

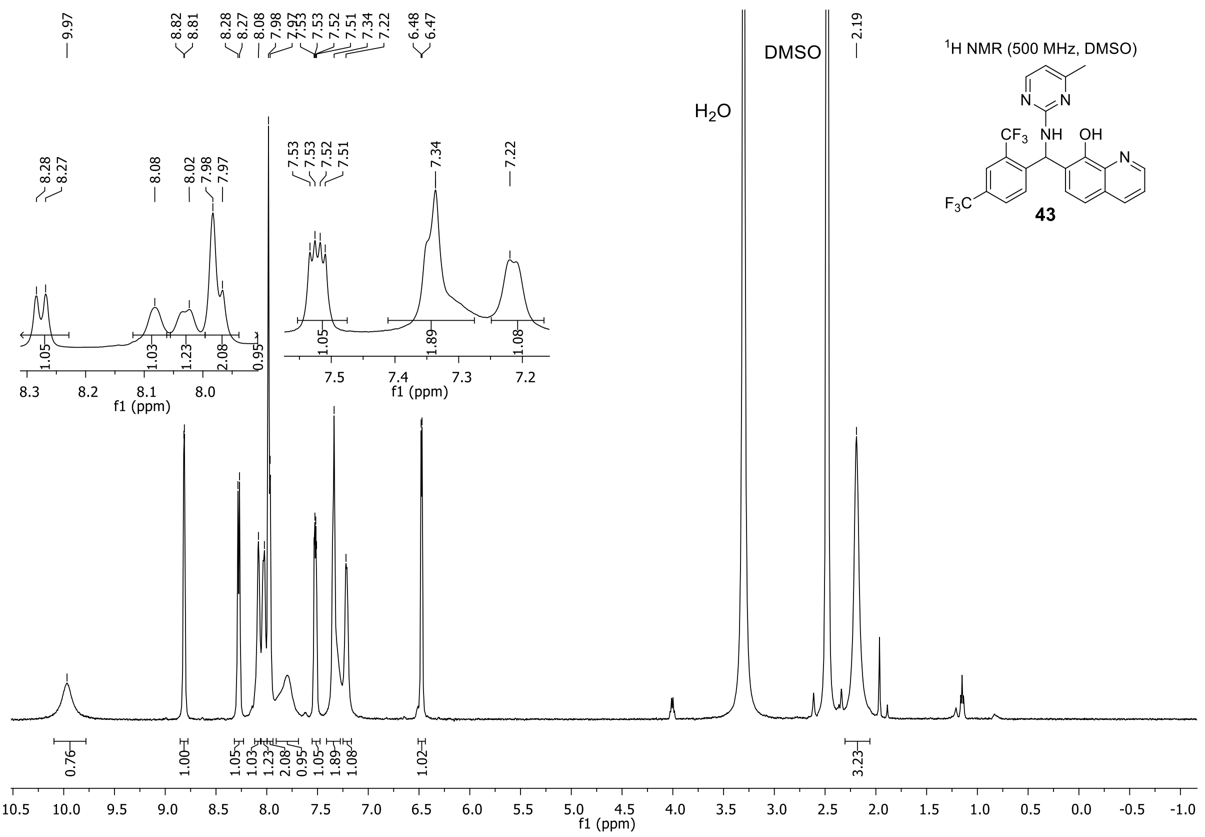

^{1}H NMR (500 MHz, DMSO)
43
DMSO
H_2O
f1 (ppm)

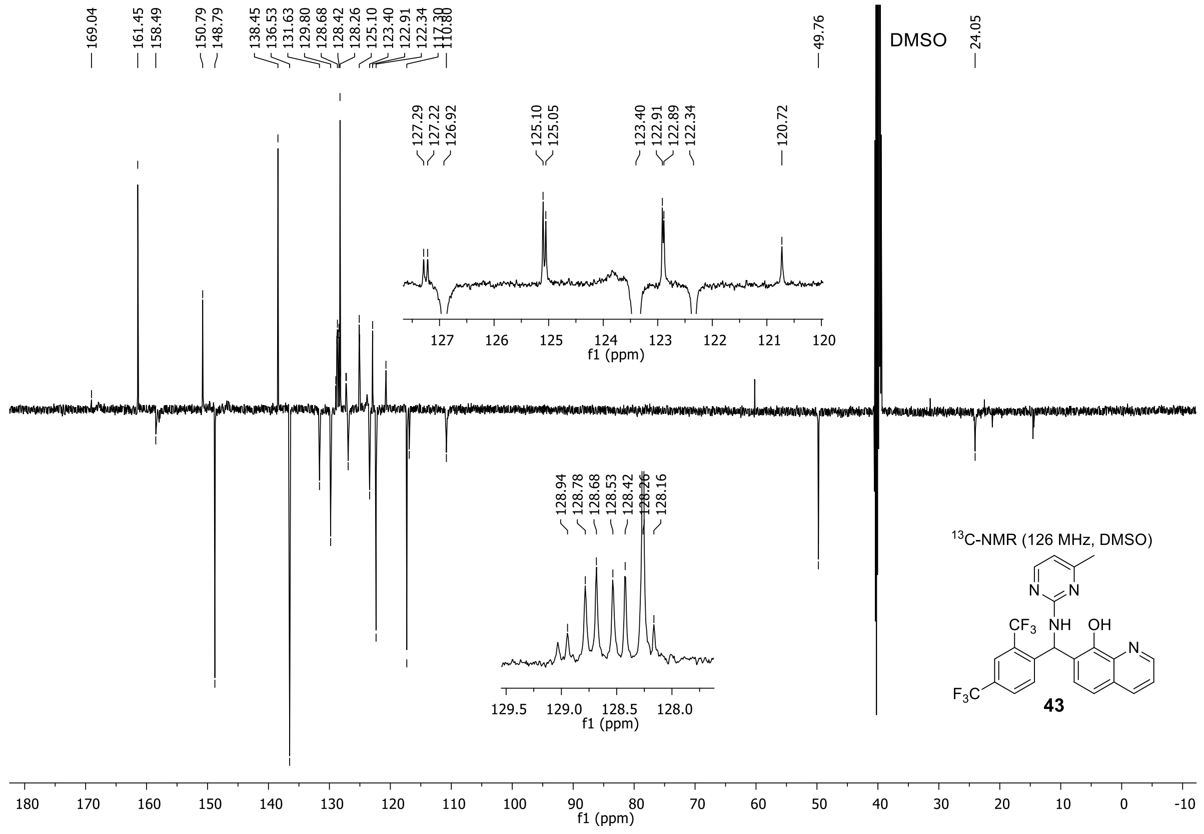

^{13}C-NMR (126 MHz, DMSO)
43
DMSO
f1 (ppm)

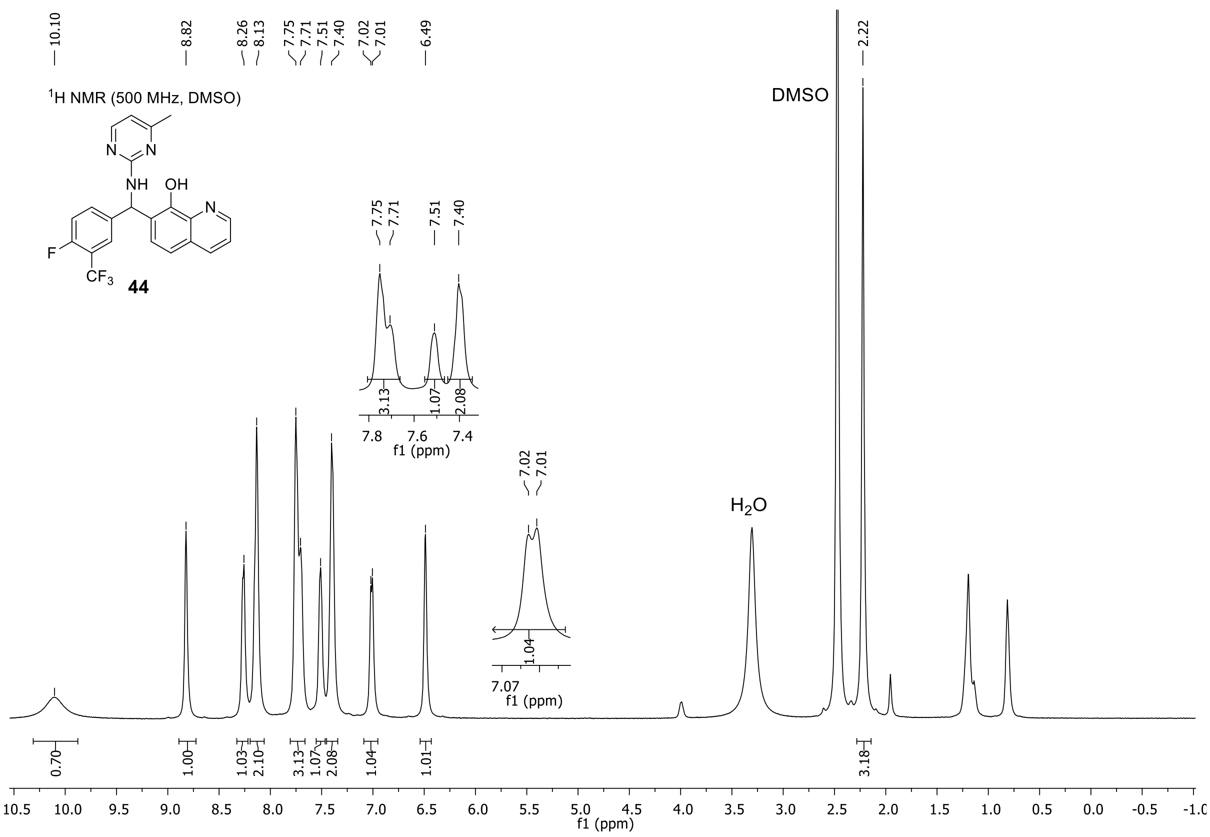
^{1}H NMR (500 MHz, DMSO)
44
DMSO
H_2O
f1 (ppm)

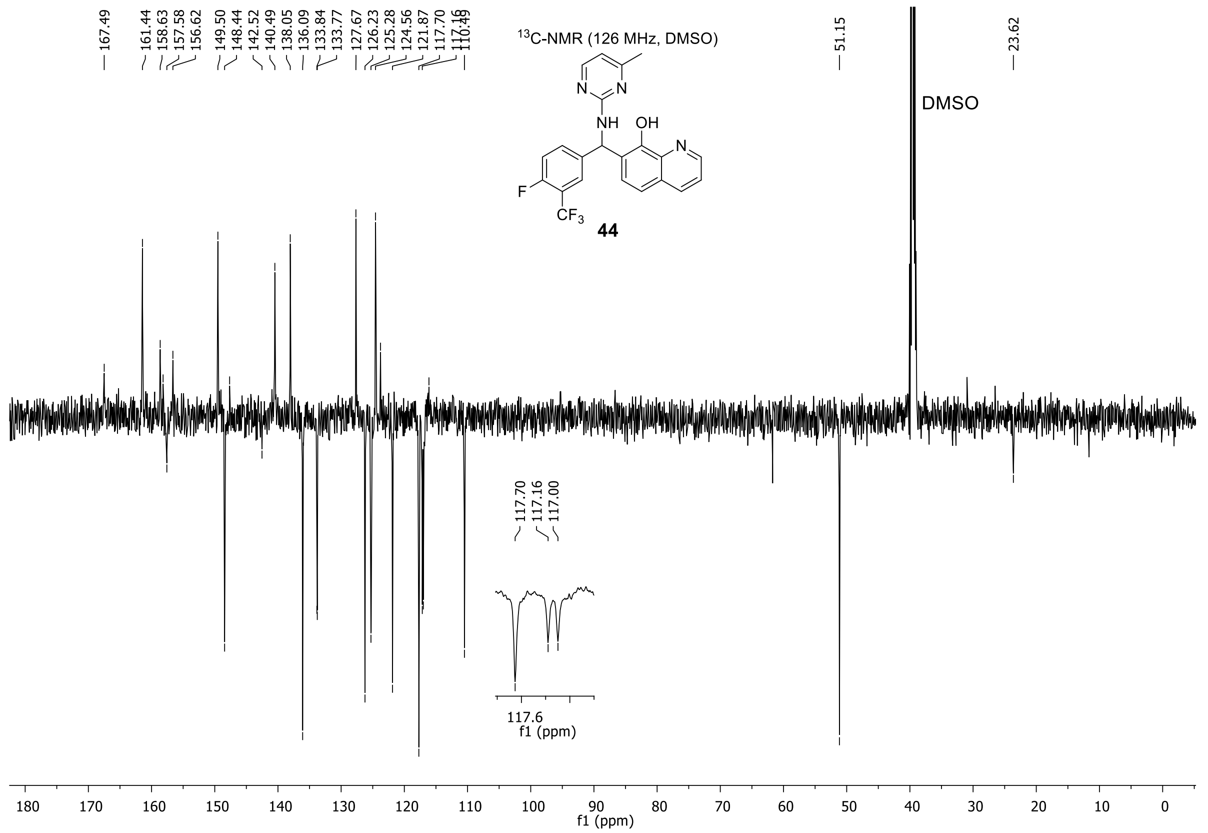
^{13}C-NMR (126 MHz, DMSO)
44
DMSO
f1 (ppm)

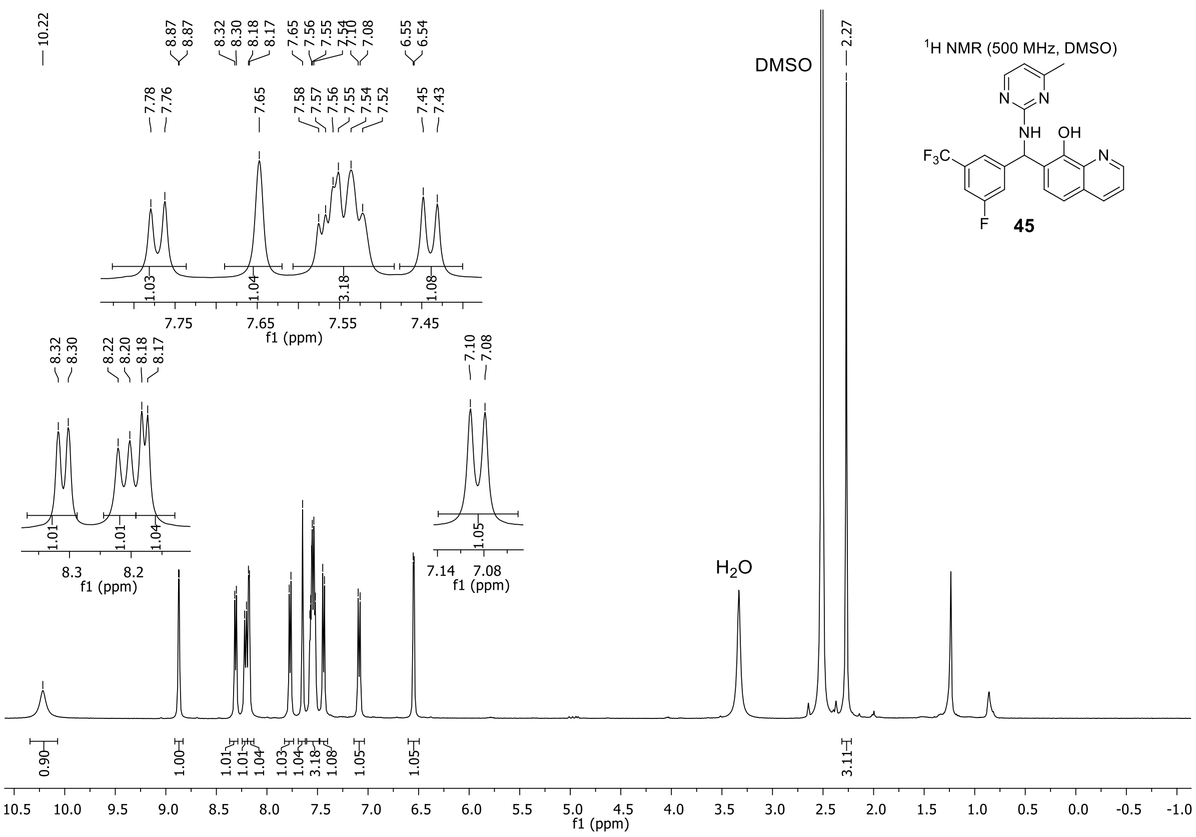
1H NMR (500 MHz, DMSO)
DMSO
H2O
45
f1 (ppm)

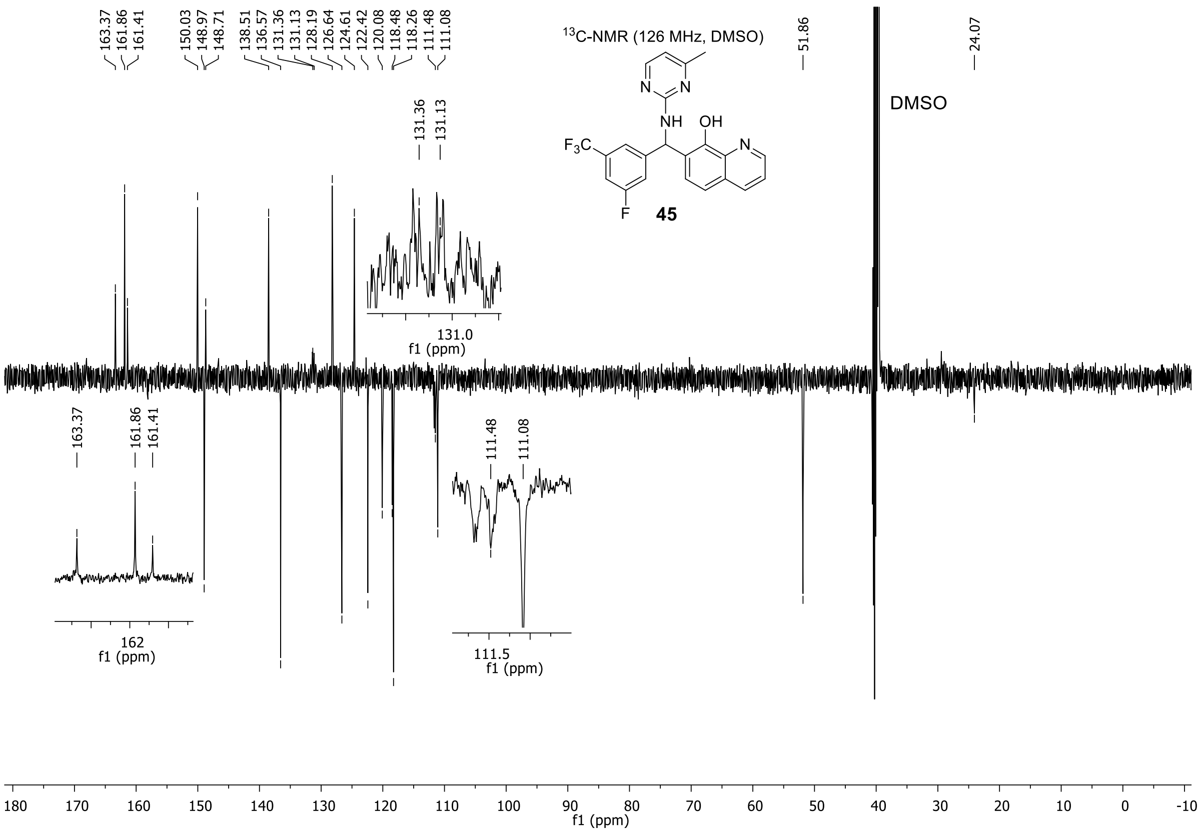
13C-NMR (126 MHz, DMSO)
DMSO
45
f1 (ppm)

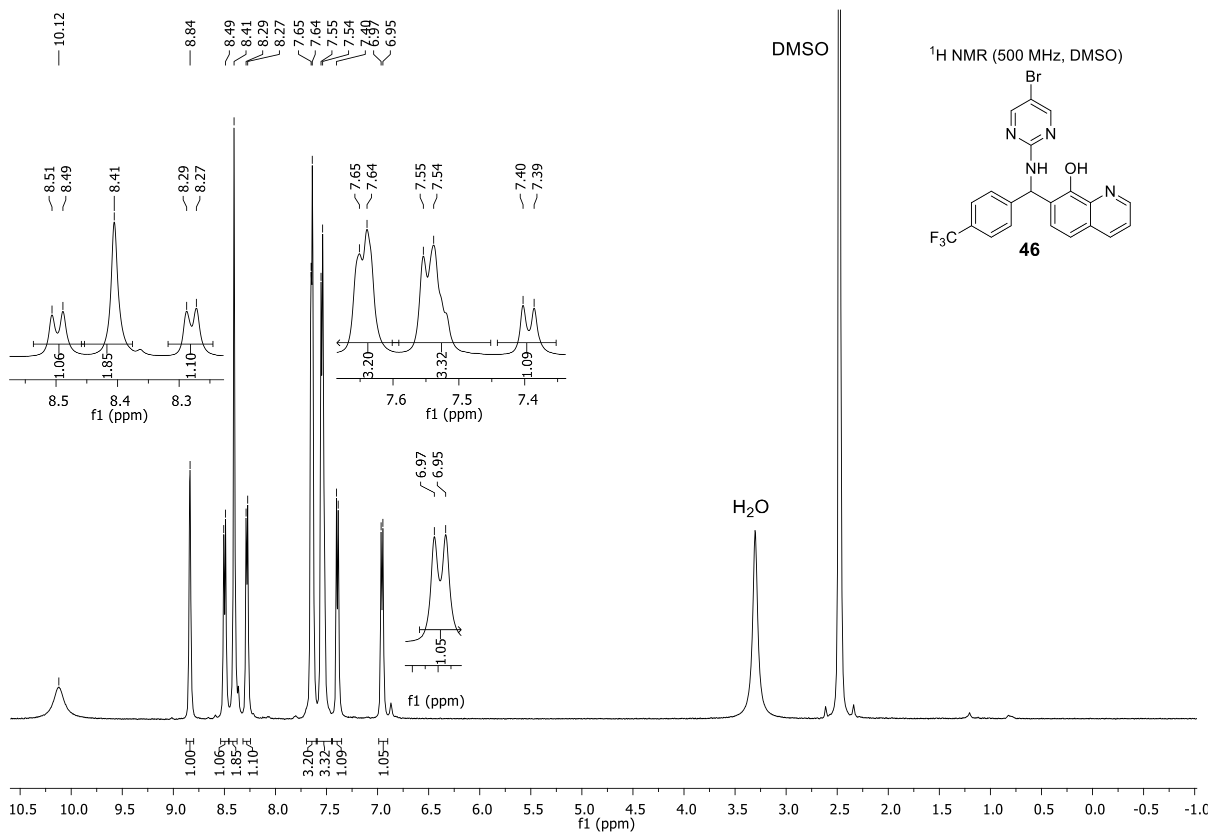
DMSO
1H NMR (500 MHz, DMSO)
H2O
46
f1 (ppm)

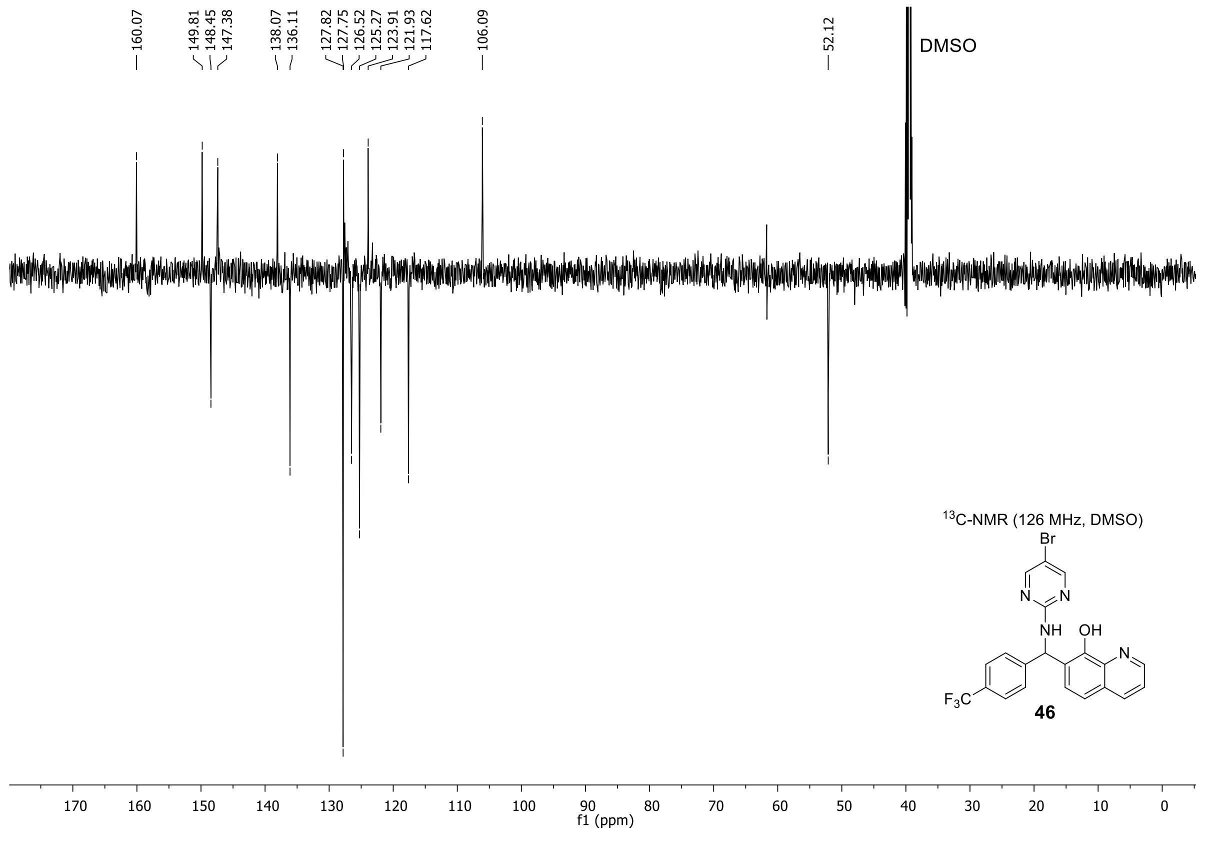
DMSO
13C-NMR (126 MHz, DMSO)
46
f1 (ppm)

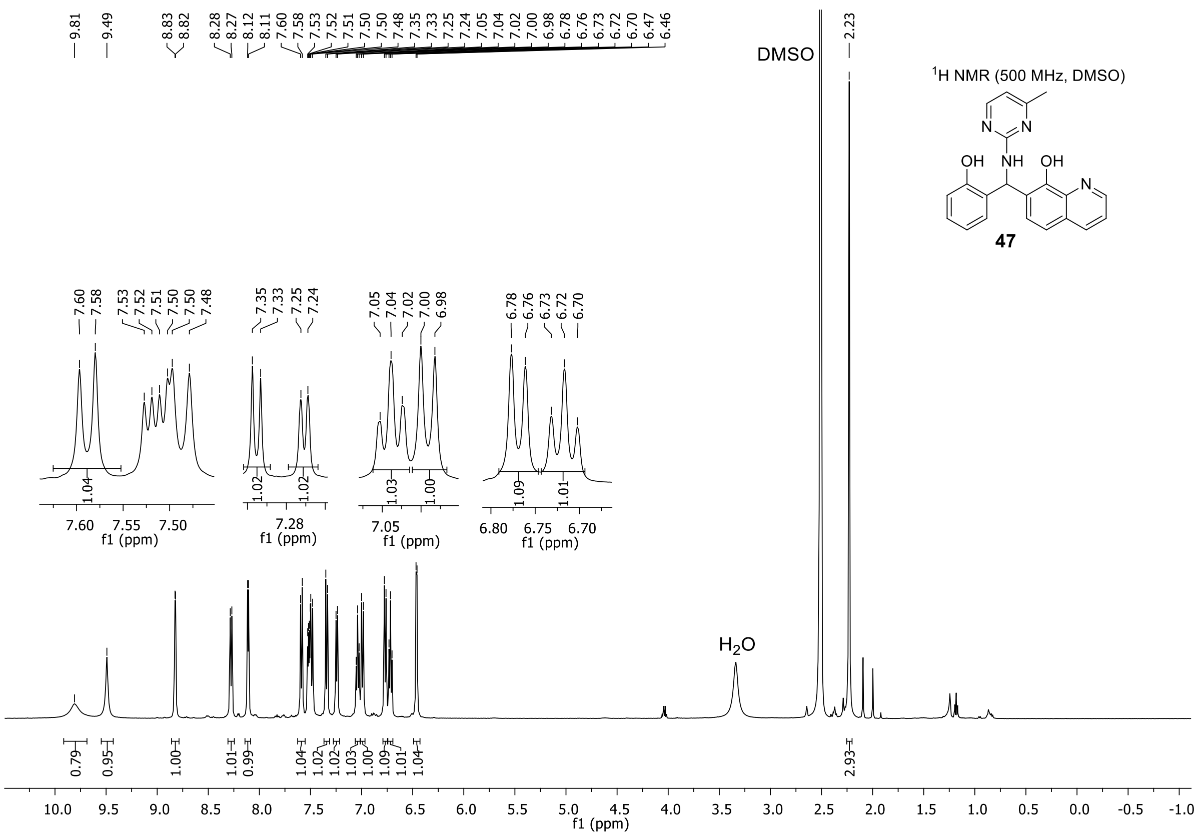
DMSO
^{1}H NMR (500 MHz, DMSO)
OH NH OH
47
H_2O
f1 (ppm)

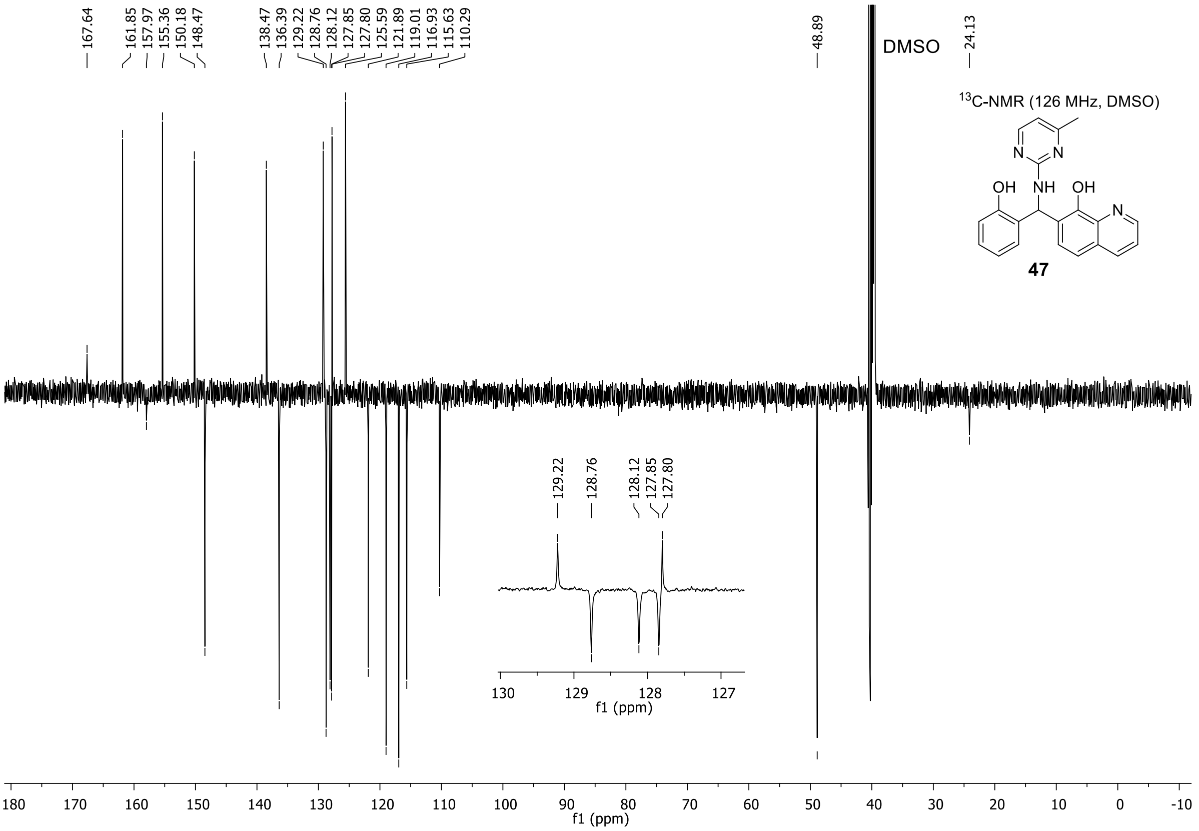
DMSO
^{13}C-NMR (126 MHz, DMSO)
OH NH OH
47
f1 (ppm)

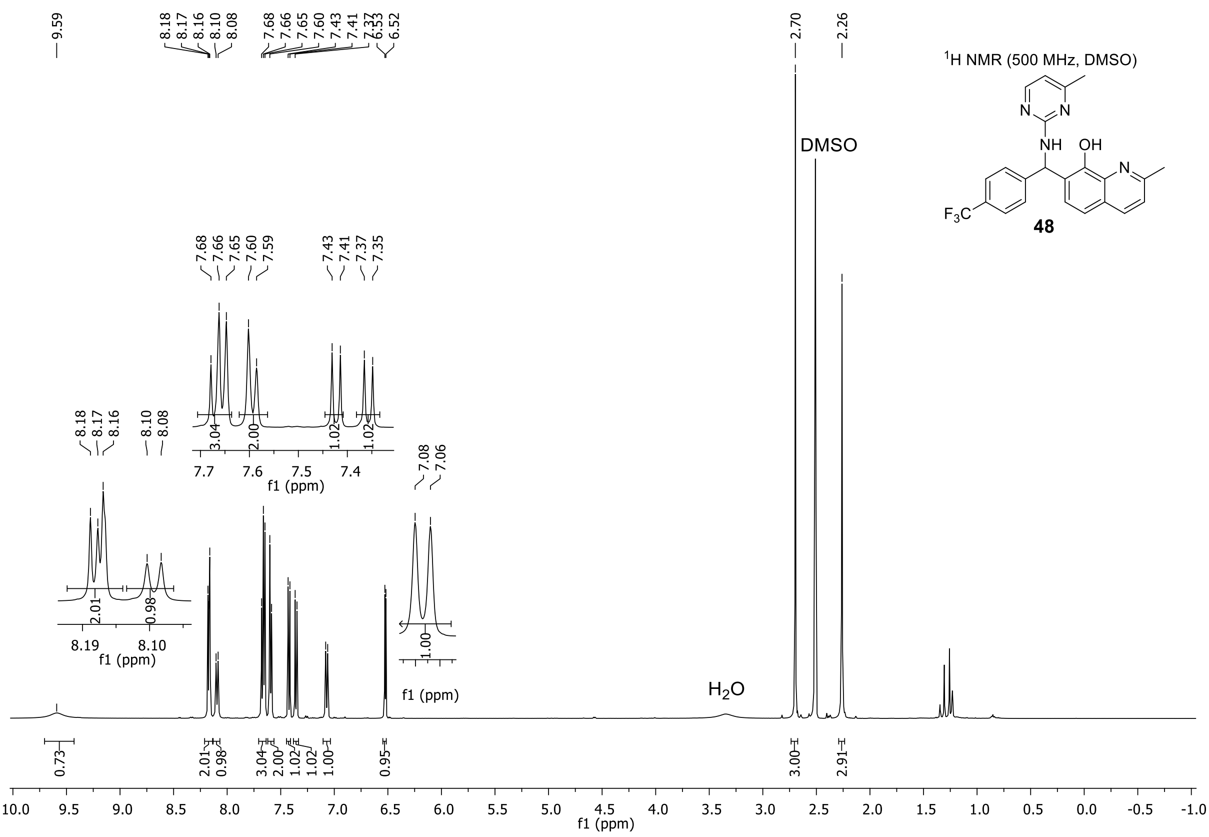

^{1}H NMR (500 MHz, DMSO)
DMSO
H_2O
48
f1 (ppm)

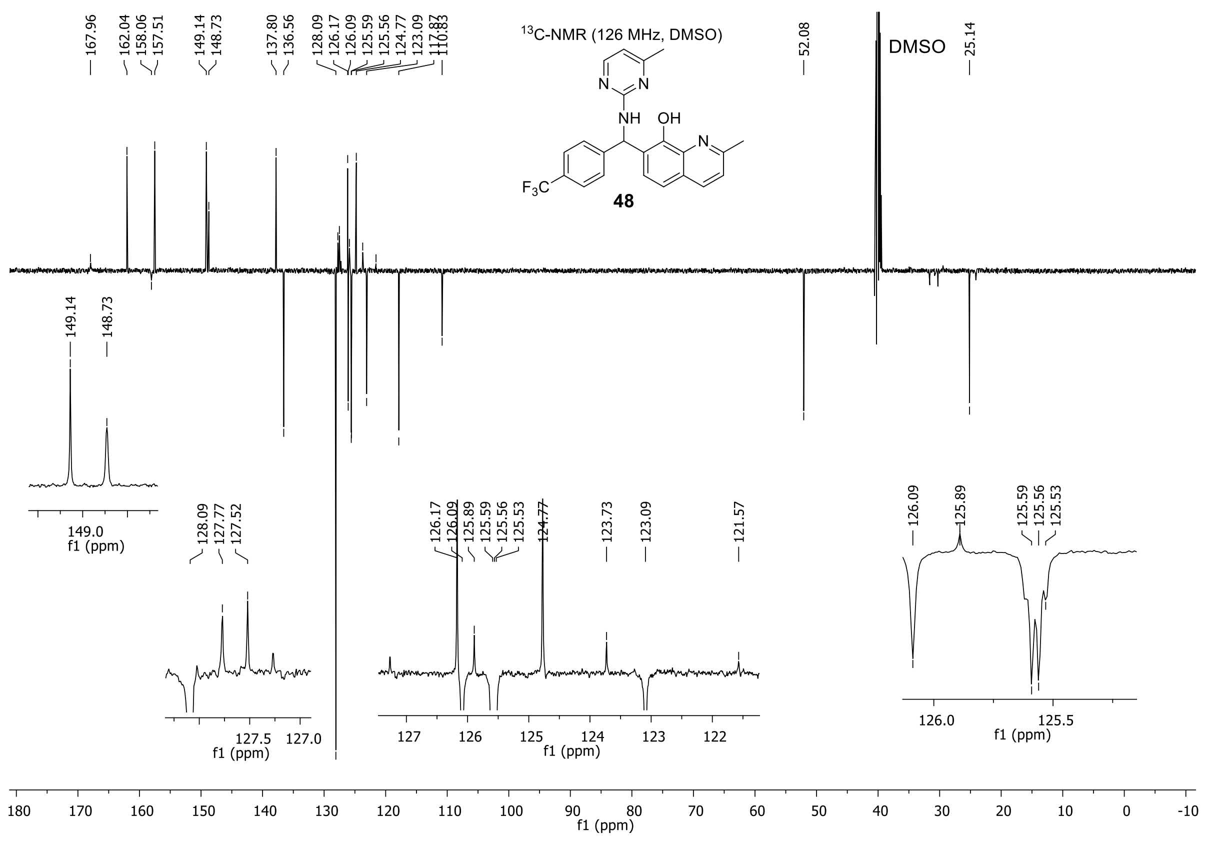

^{13}C-NMR (126 MHz, DMSO)
DMSO
48
f1 (ppm)

II. HRMS spectra:

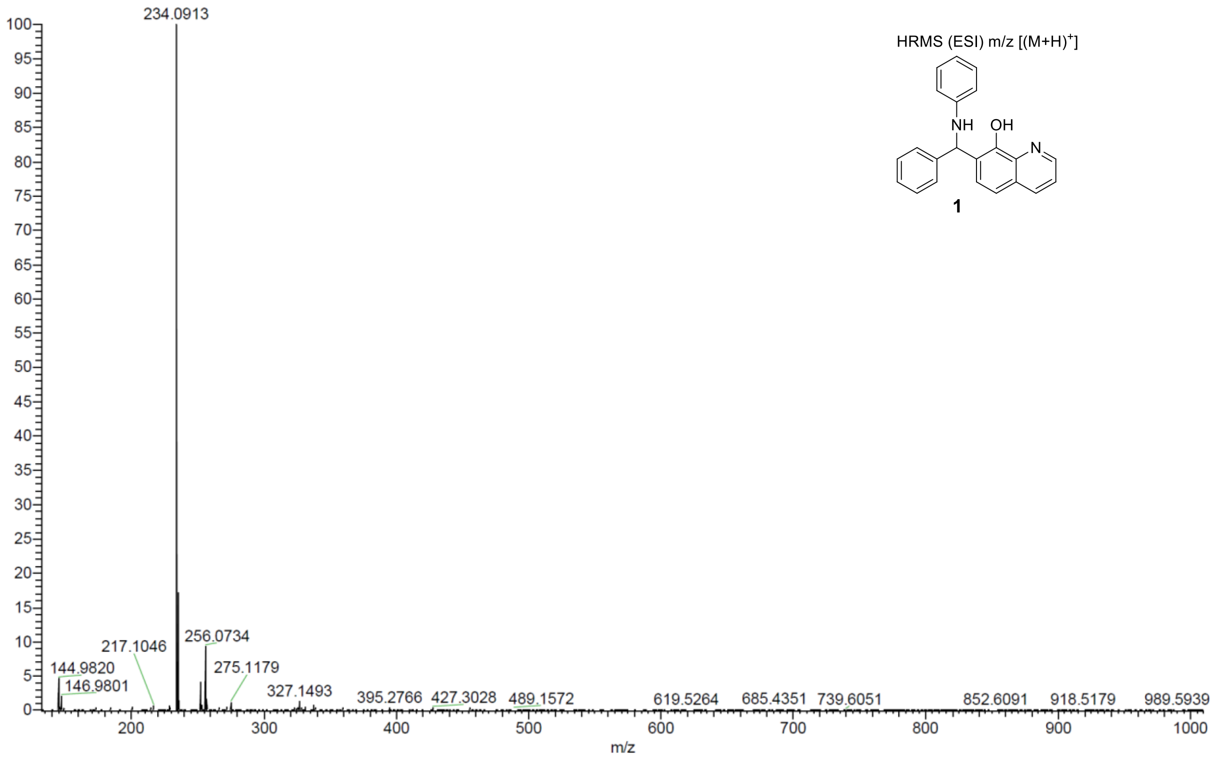

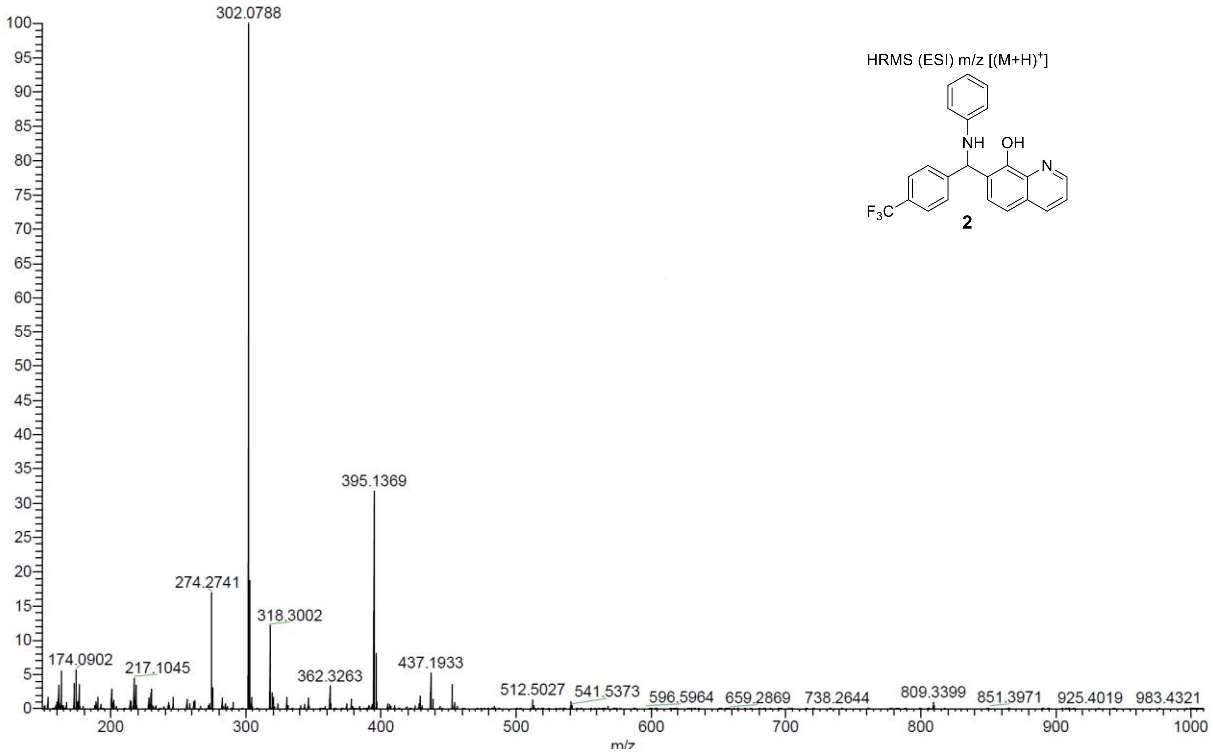

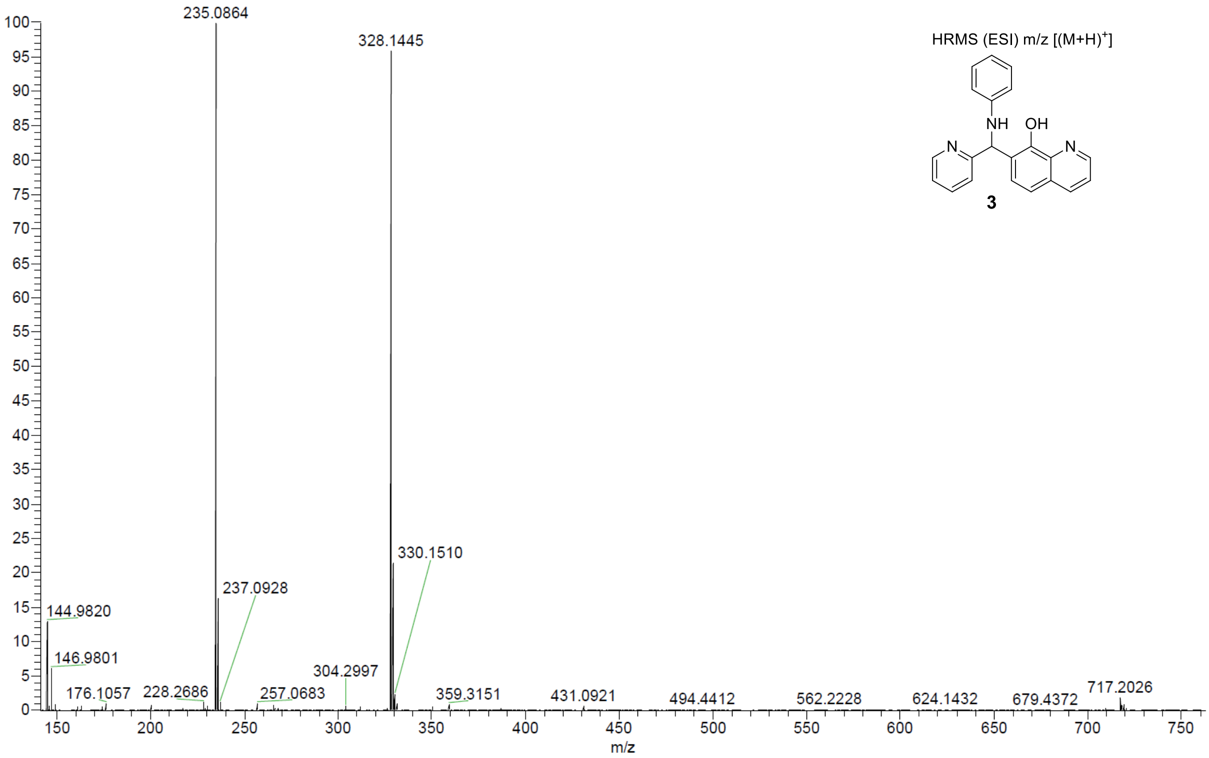
HRMS (ESI) m/z [(M+H)+]
3
235.0864
328.1445
330.1510
237.0928
144.9820
146.9801
176.1057
228.2686
257.0683
304.2997
359.3151
431.0921
494.4412
562.2228
624.1432
679.4372
717.2026
m/z

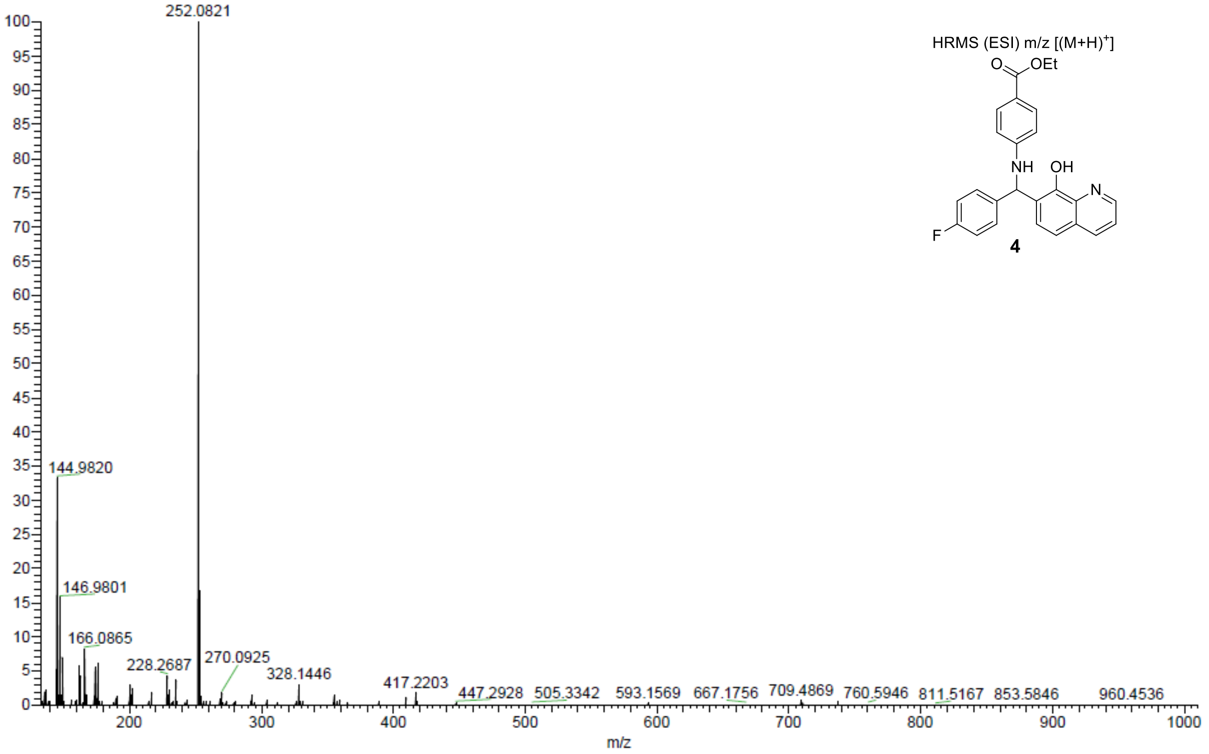
HRMS (ESI) m/z [(M+H)+]
4
252.0821
144.9820
146.9801
166.0865
228.2687
270.0925
328.1446
417.2203
447.2928
505.3342
593.1569
667.1756
709.4869
760.5946
811.5167
853.5846
960.4536
m/z

S49

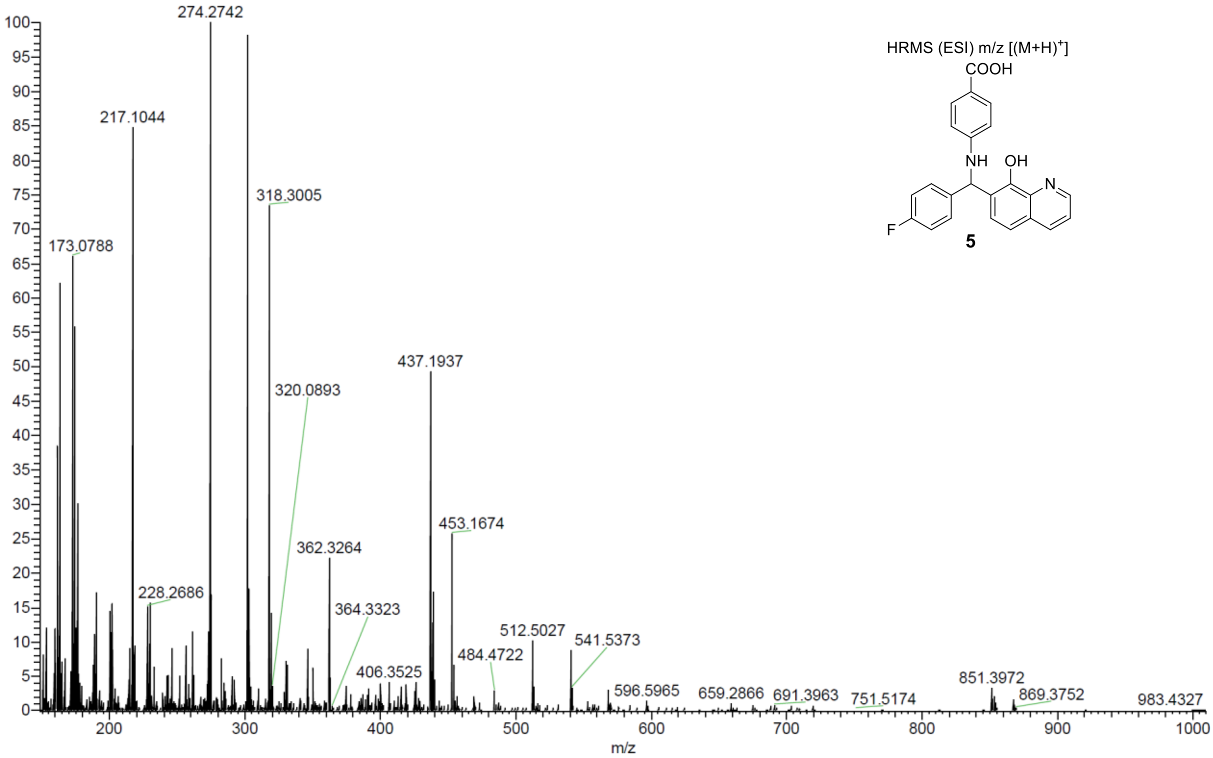
HRMS (ESI) m/z [(M+H)+]
COOH
NH
OH
N
F
5
274.2742
217.1044
318.3005
173.0788
437.1937
320.0893
453.1674
362.3264
228.2686
364.3323
512.5027
541.5373
484.4722
406.3525
851.3972
596.5965
659.2866
691.3963
751.5174
869.3752
983.4327
m/z

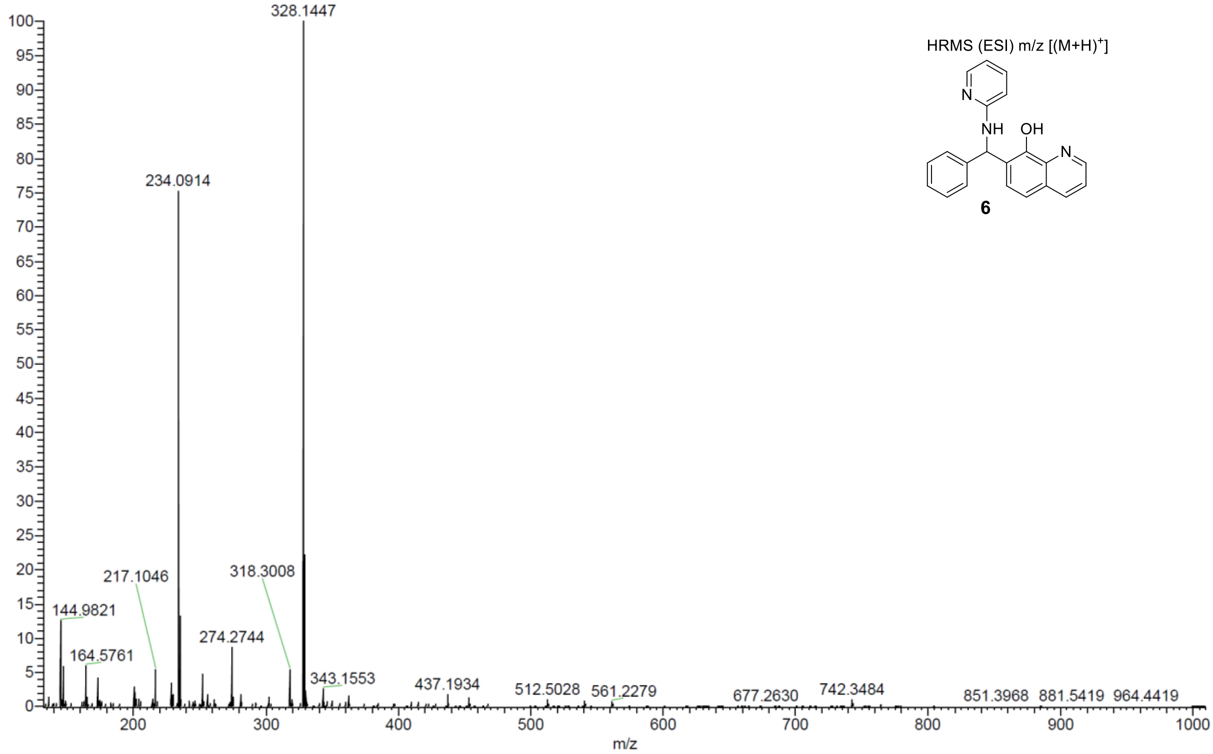
HRMS (ESI) m/z [(M+H)+]
N
NH
OH
N
6
328.1447
234.0914
217.1046
318.3008
144.9821
274.2744
164.5761
343.1553
437.1934
512.5028
561.2279
677.2630
742.3484
851.3968
881.5419
964.4419
m/z

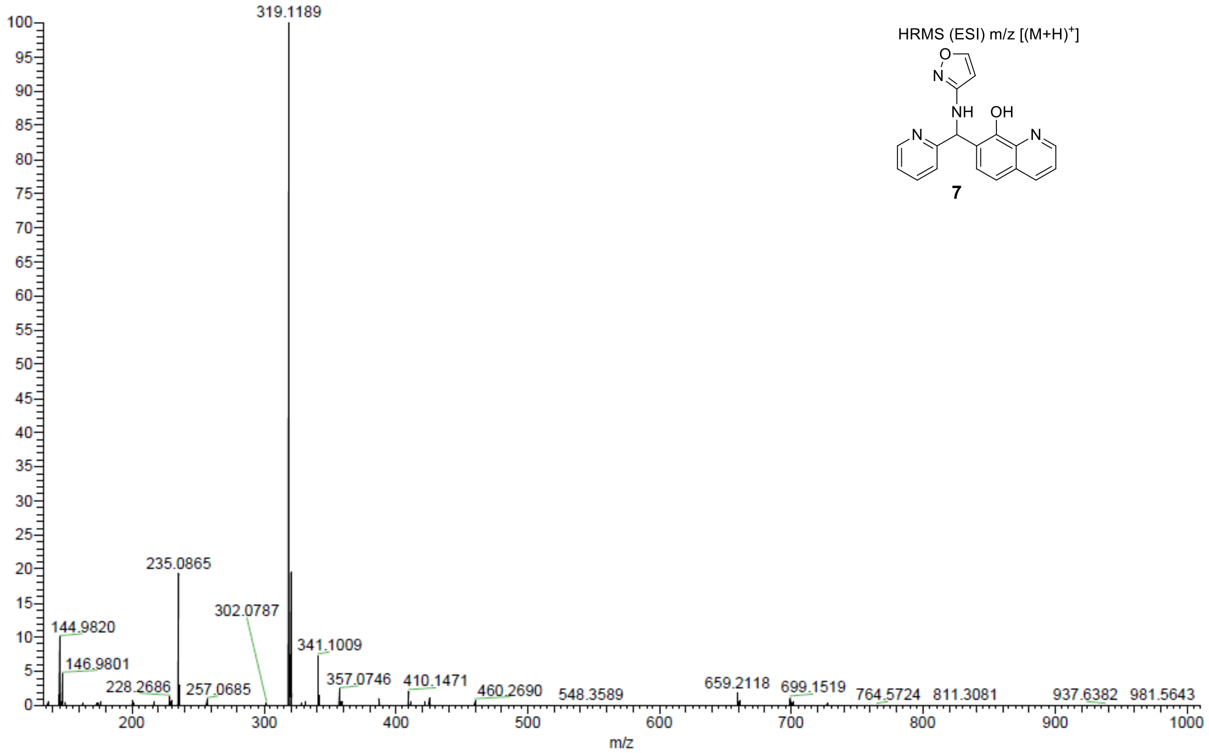
319.1189
HRMS (ESI) m/z [(M+H)+]
7
235.0865
302.0787
144.9820
341.1009
146.9801
228.2686
257.0685
357.0746
410.1471
460.2690
548.3589
659.2118
699.1519
764.5724
811.3081
937.6382
981.5643
m/z

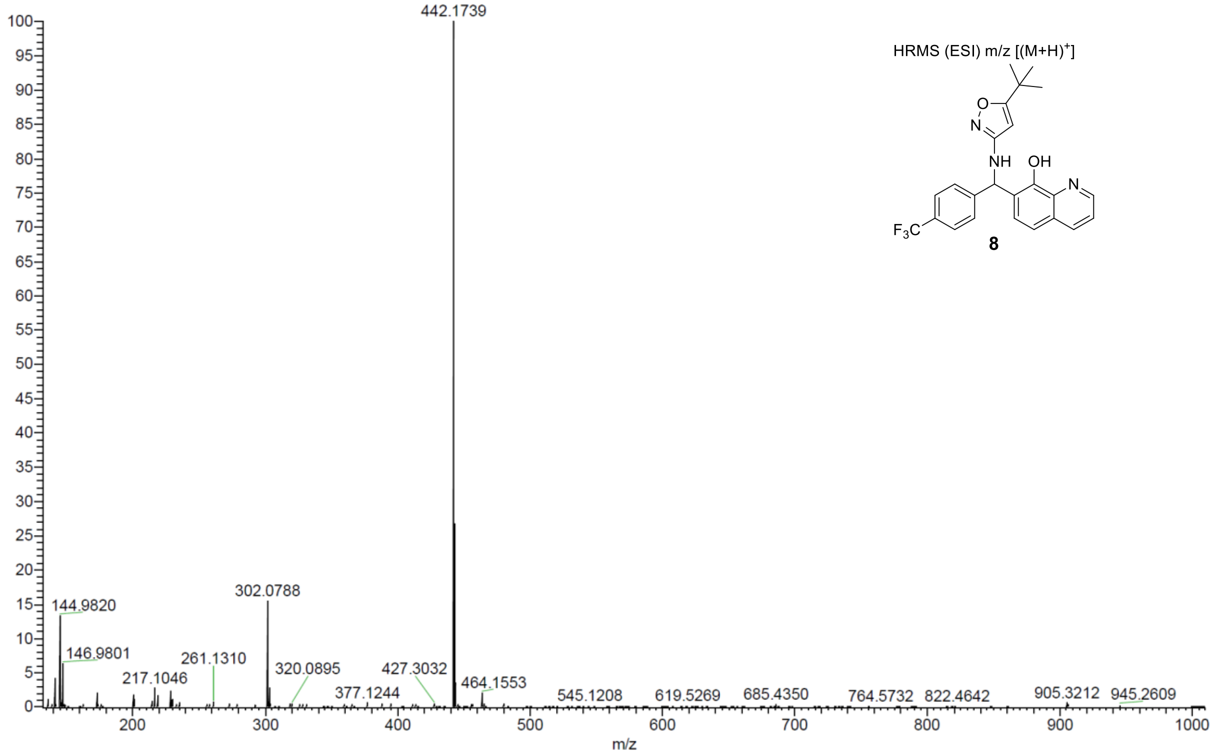
442.1739
HRMS (ESI) m/z [(M+H)+]
8
144.9820
302.0788
146.9801
261.1310
217.1046
320.0895
427.3032
377.1244
464.1553
545.1208
619.5269
685.4350
764.5732
822.4642
905.3212
945.2609
m/z

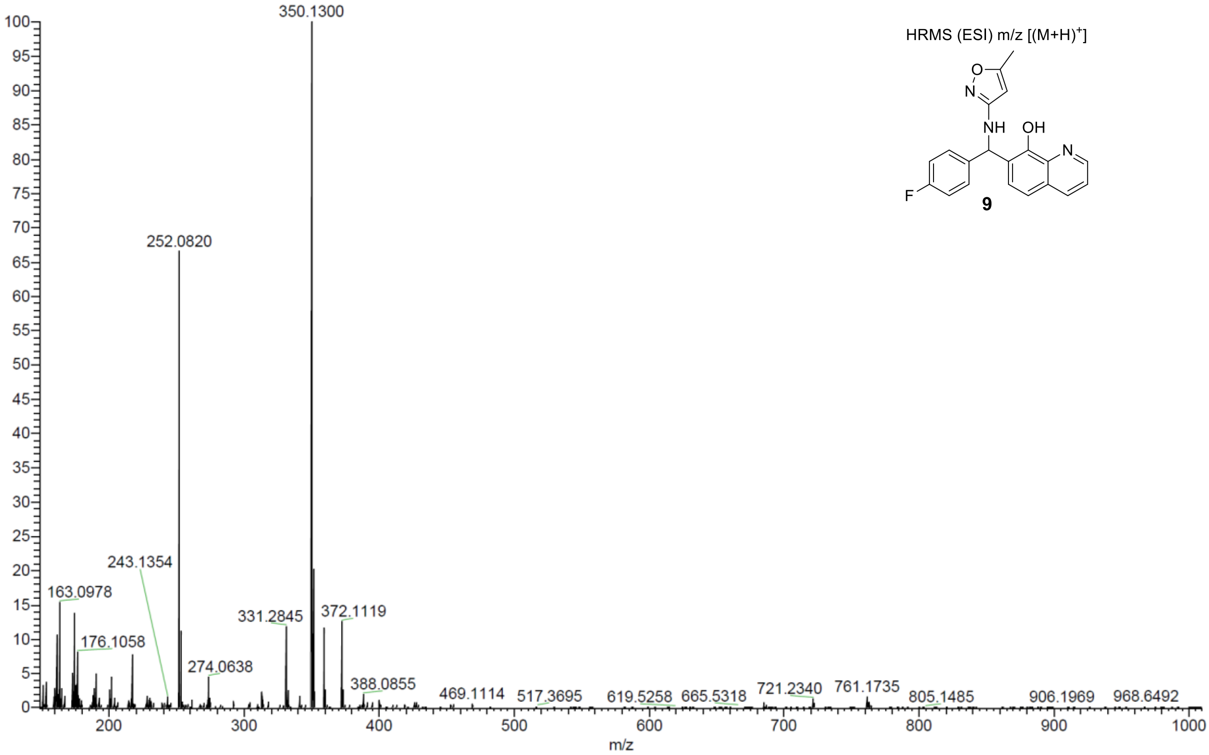

350.1300
252.0820
243.1354
163.0978
176.1058
274.0638
331.2845
372.1119
388.0855
469.1114
517.3695
619.5258
665.5318
721.2340
761.1735
805.1485
906.1969
968.6492
m/z
HRMS (ESI) m/z [(M+H)+]
9

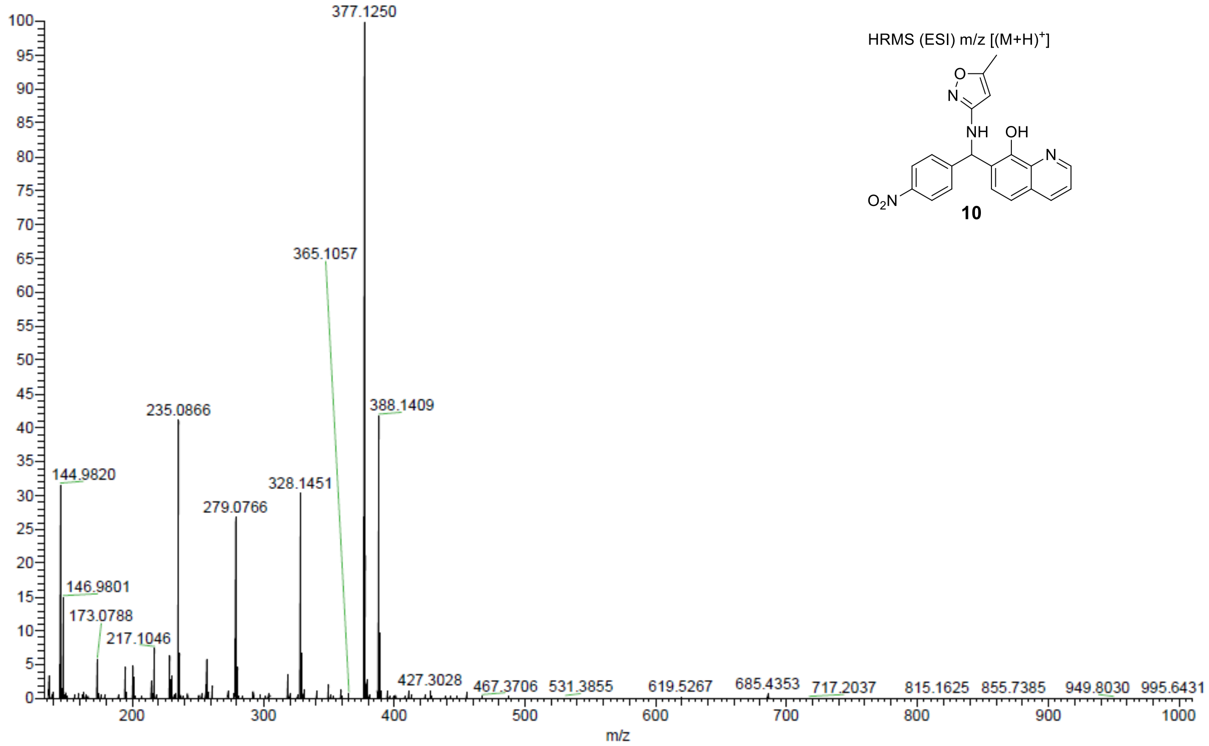

377.1250
365.1057
388.1409
235.0866
144.9820
328.1451
279.0766
146.9801
173.0788
217.1046
427.3028
467.3706
531.3855
619.5267
685.4353
717.2037
815.1625
855.7385
949.8030
995.6431
m/z
HRMS (ESI) m/z [(M+H)+]
10

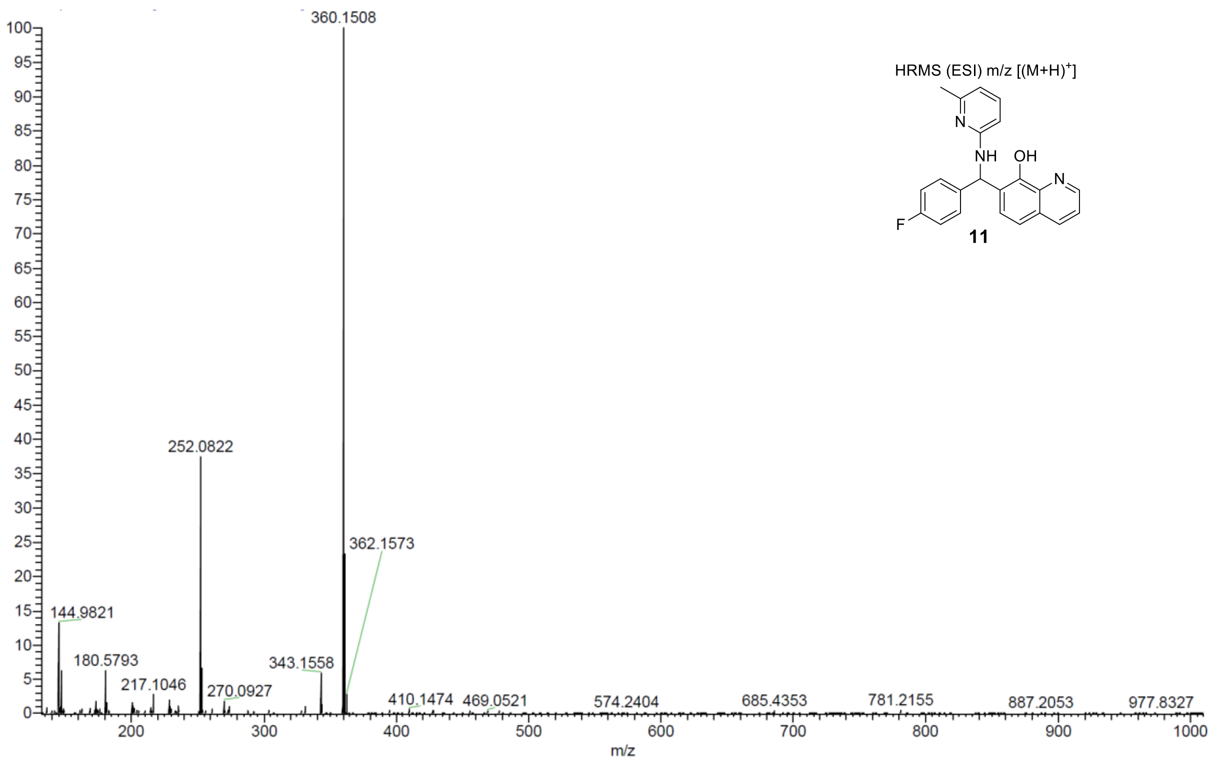
360.1508
HRMS (ESI) m/z [(M+H)+]
NH OH
N
F
11
252.0822
362.1573
144.9821
180.5793
217.1046
270.0927
343.1558
410.1474
469.0521
574.2404
685.4353
781.2155
887.2053
977.8327
m/z

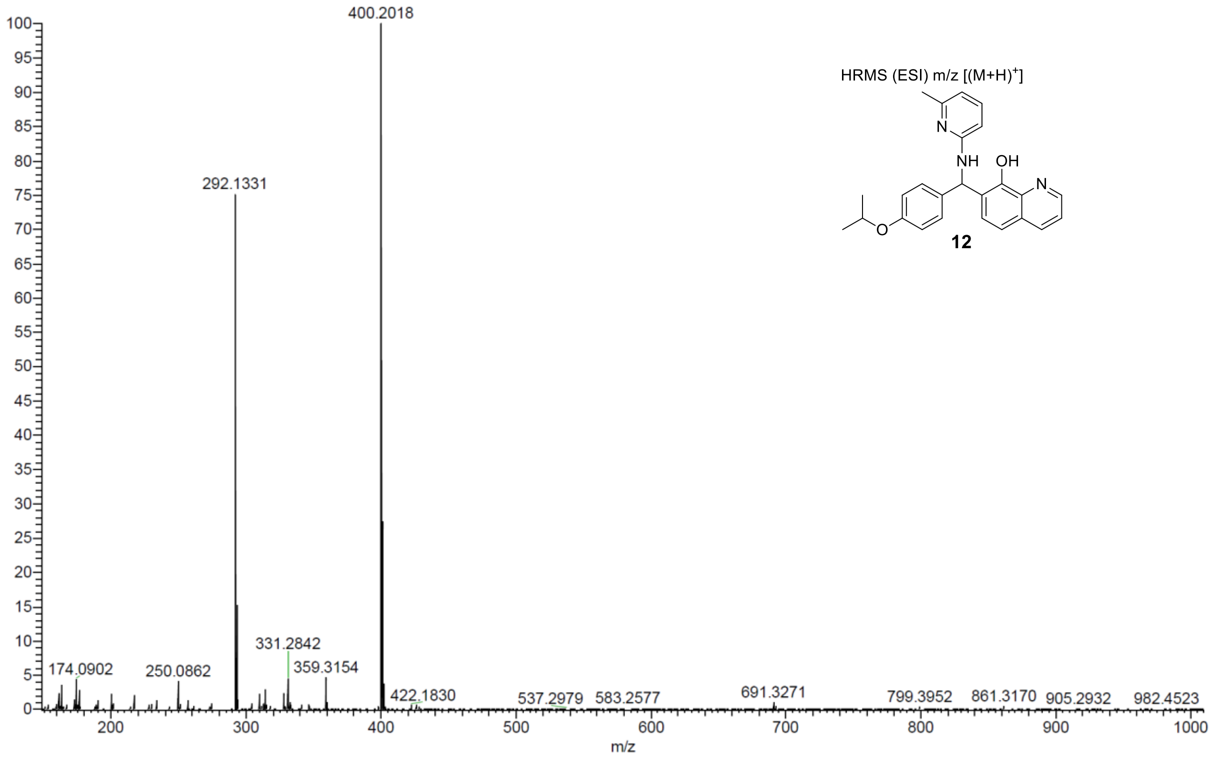
400.2018
HRMS (ESI) m/z [(M+H)+]
NH OH
N
O
12
292.1331
331.2842
359.3154
174.0902
250.0862
422.1830
537.2979
583.2577
691.3271
799.3952
861.3170
905.2932
982.4523
m/z

S53

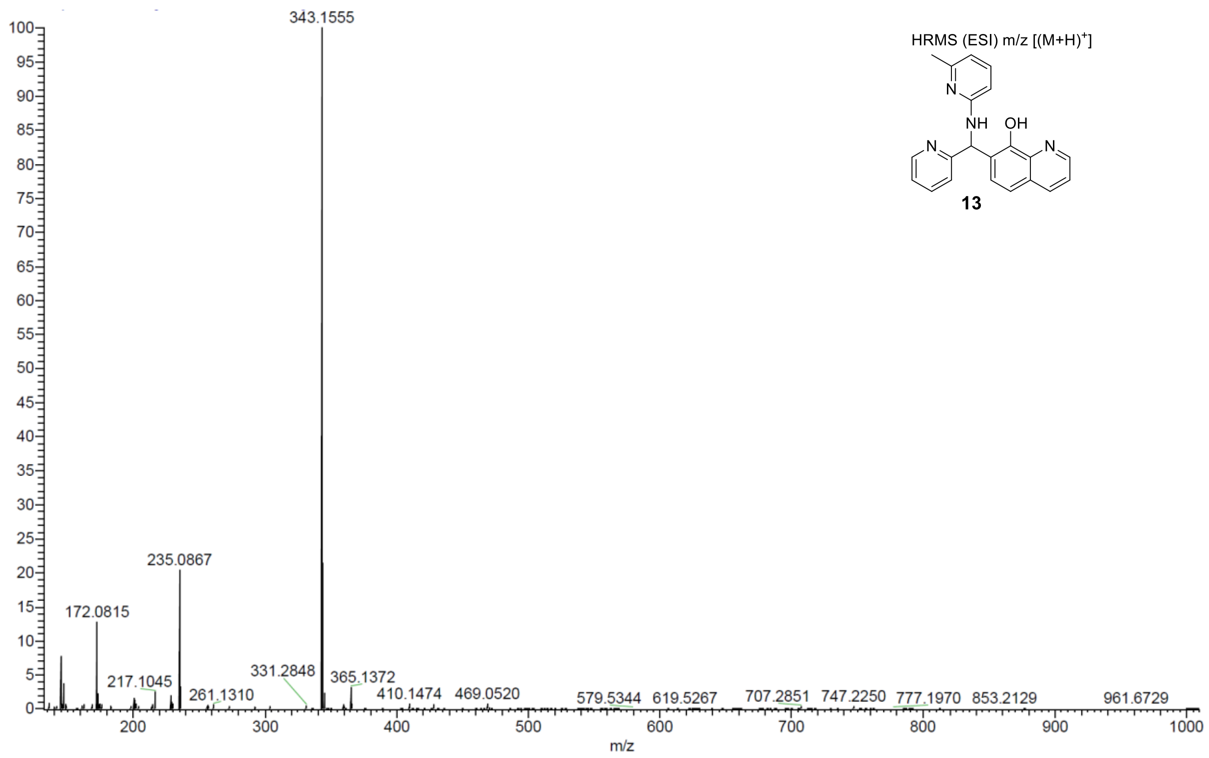
HRMS (ESI) m/z [(M+H)+]
13
343.1555
235.0867
172.0815
217.1045
261.1310
331.2848
365.1372
410.1474
469.0520
579.5344
619.5267
707.2851
747.2250
777.1970
853.2129
961.6729
m/z

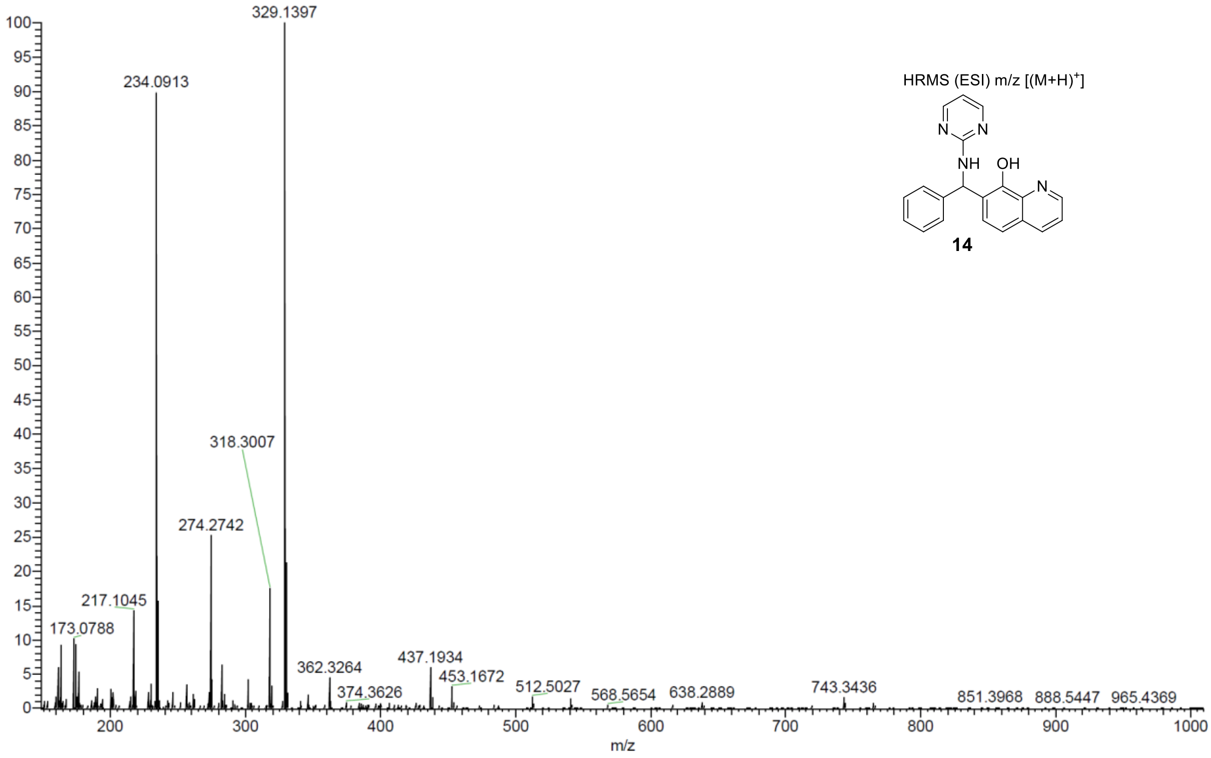
HRMS (ESI) m/z [(M+H)+]
14
329.1397
234.0913
318.3007
274.2742
217.1045
173.0788
362.3264
374.3626
437.1934
453.1672
512.5027
568.5654
638.2889
743.3436
851.3968
888.5447
965.4369
m/z

S54

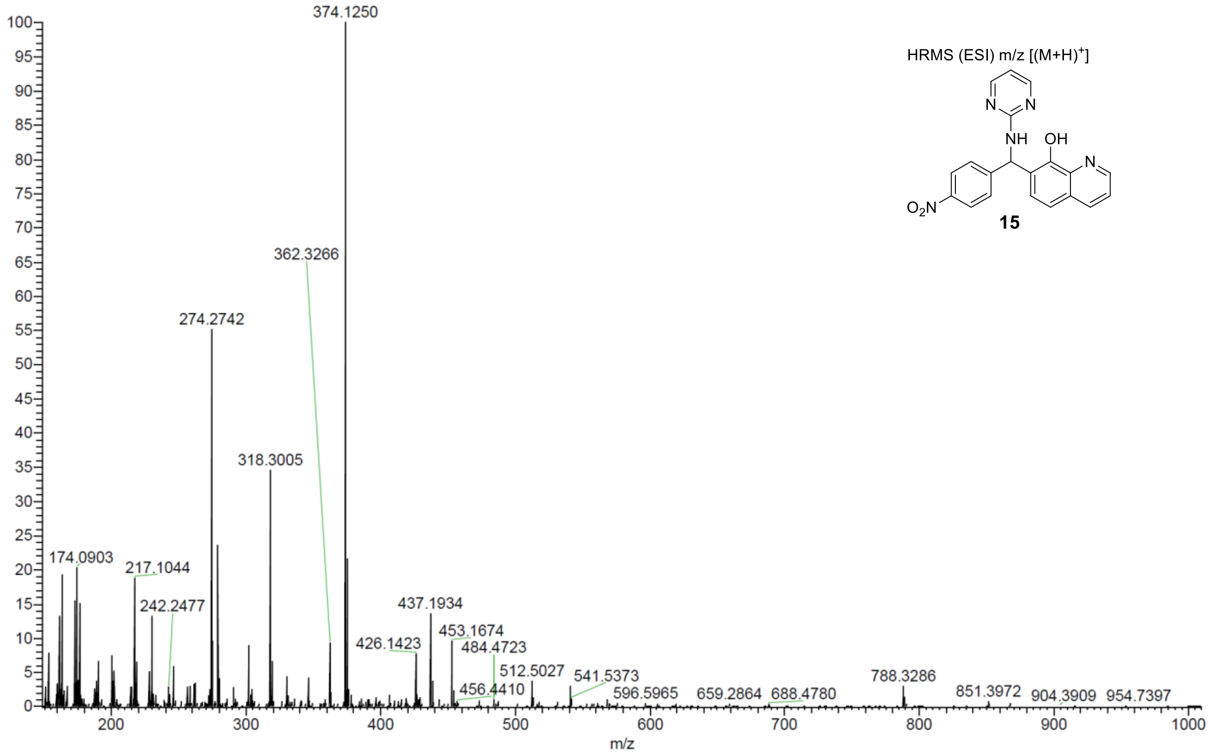

374.1250
HRMS (ESI) m/z [(M+H)+]
362.3266
274.2742
318.3005
174.0903
217.1044
242.2477
437.1934
453.1674
484.4723
426.1423
512.5027
541.5373
456.4410
596.5965
659.2864
688.4780
788.3286
851.3972
904.3909
954.7397
m/z
15

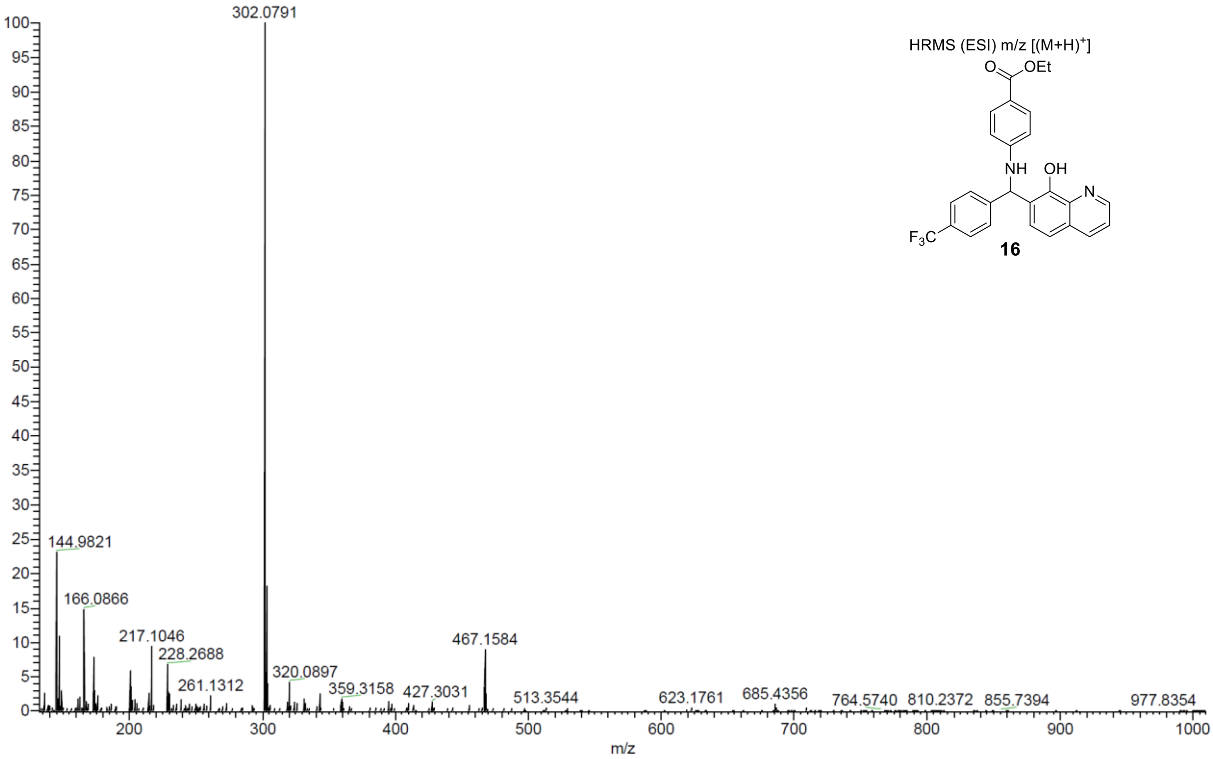

302.0791
HRMS (ESI) m/z [(M+H)+]
144.9821
166.0866
217.1046
228.2688
261.1312
320.0897
359.3158
427.3031
467.1584
513.3544
623.1761
685.4356
764.5740
810.2372
855.7394
977.8354
m/z
16

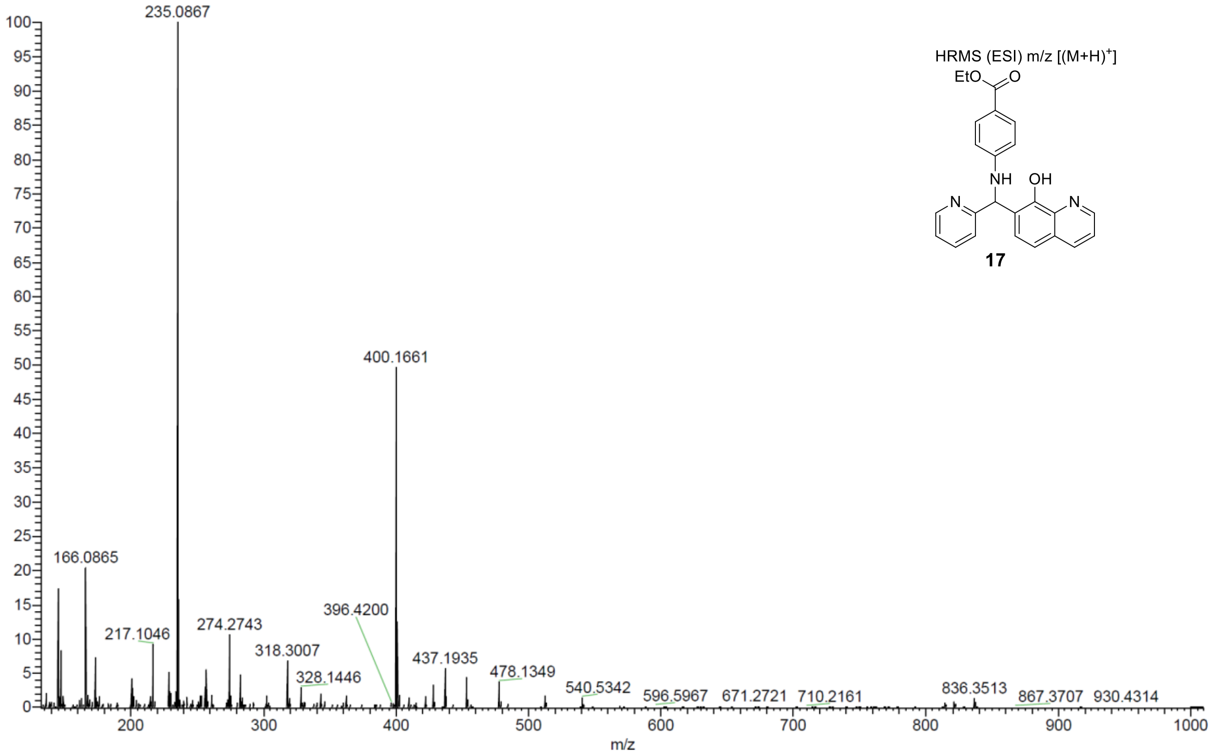
HRMS (ESI) m/z [(M+H)+]
EtO
O
NH
OH
N
N
17
235.0867
400.1661
166.0865
217.1046
274.2743
318.3007
328.1446
396.4200
437.1935
478.1349
540.5342
596.5967
671.2721
710.2161
836.3513
867.3707
930.4314
m/z

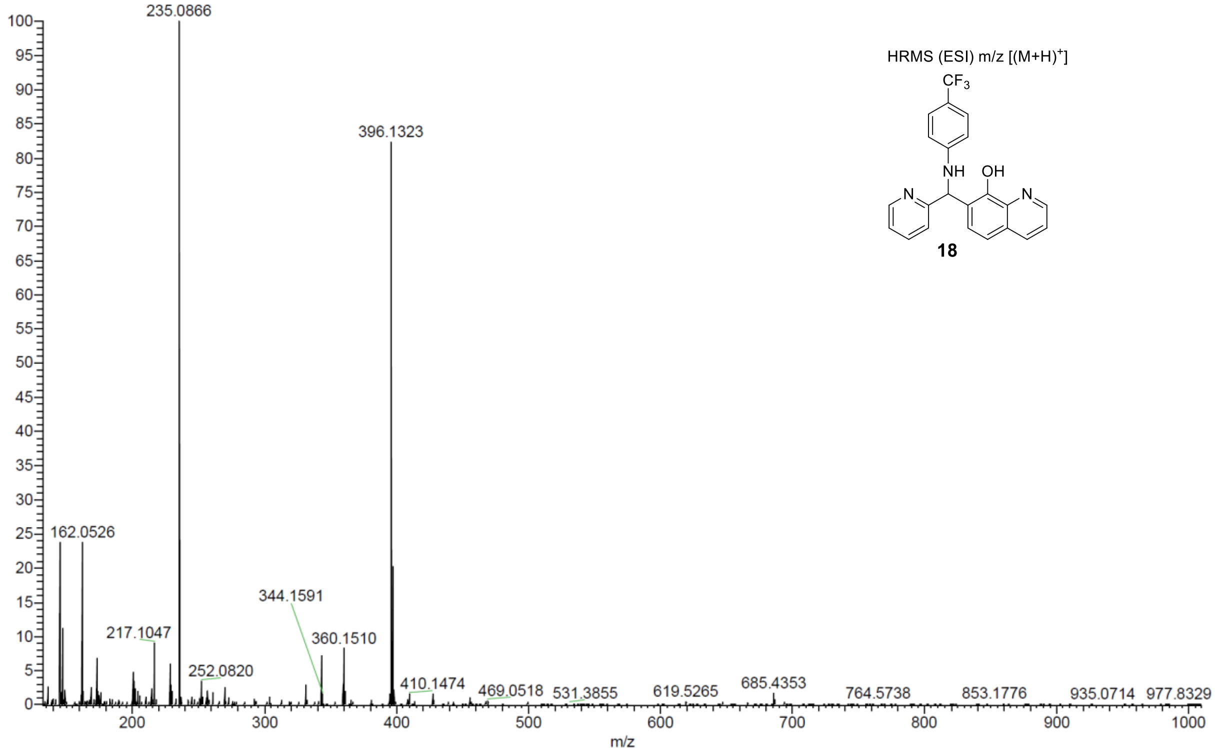
HRMS (ESI) m/z [(M+H)+]
CF3
NH
OH
N
N
18
235.0866
396.1323
162.0526
217.1047
252.0820
344.1591
360.1510
410.1474
469.0518
531.3855
619.5265
685.4353
764.5738
853.1776
935.0714
977.8329
m/z

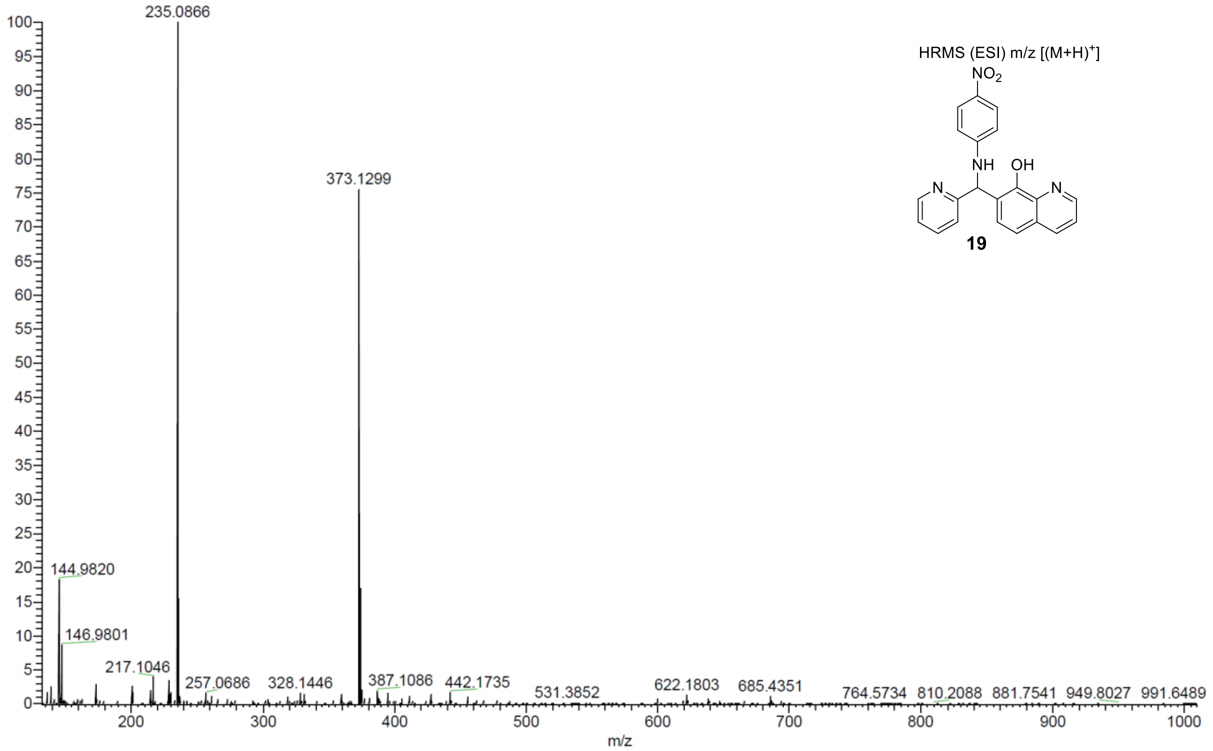
HRMS (ESI) m/z [(M+H)+]
NO2
NH OH
N
N
19
235.0866
373.1299
144.9820
146.9801
217.1046
257.0686
328.1446
387.1086
442.1735
531.3852
622.1803
685.4351
764.5734
810.2088
881.7541
949.8027
991.6489
m/z

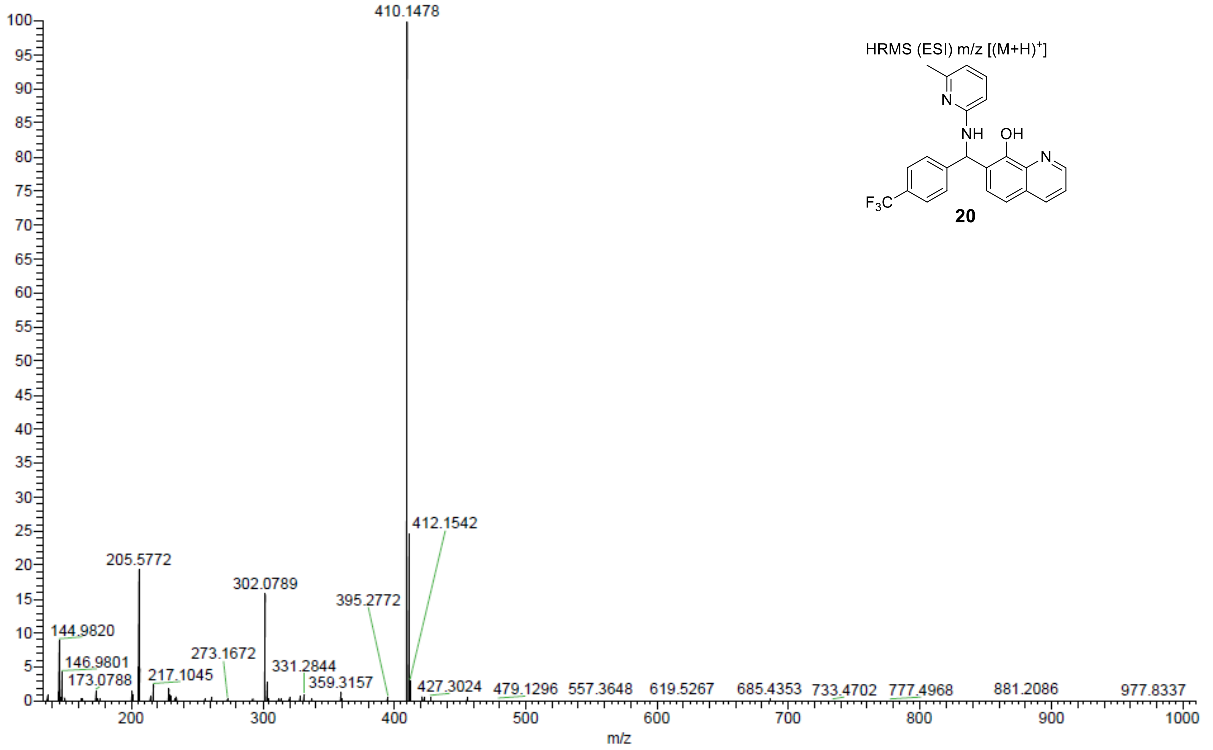
HRMS (ESI) m/z [(M+H)+]
N
NH OH
N
F3C
20
410.1478
412.1542
205.5772
302.0789
395.2772
144.9820
146.9801
173.0788
217.1045
273.1672
331.2844
359.3157
427.3024
479.1296
557.3648
619.5267
685.4353
733.4702
777.4968
881.2086
977.8337
m/z

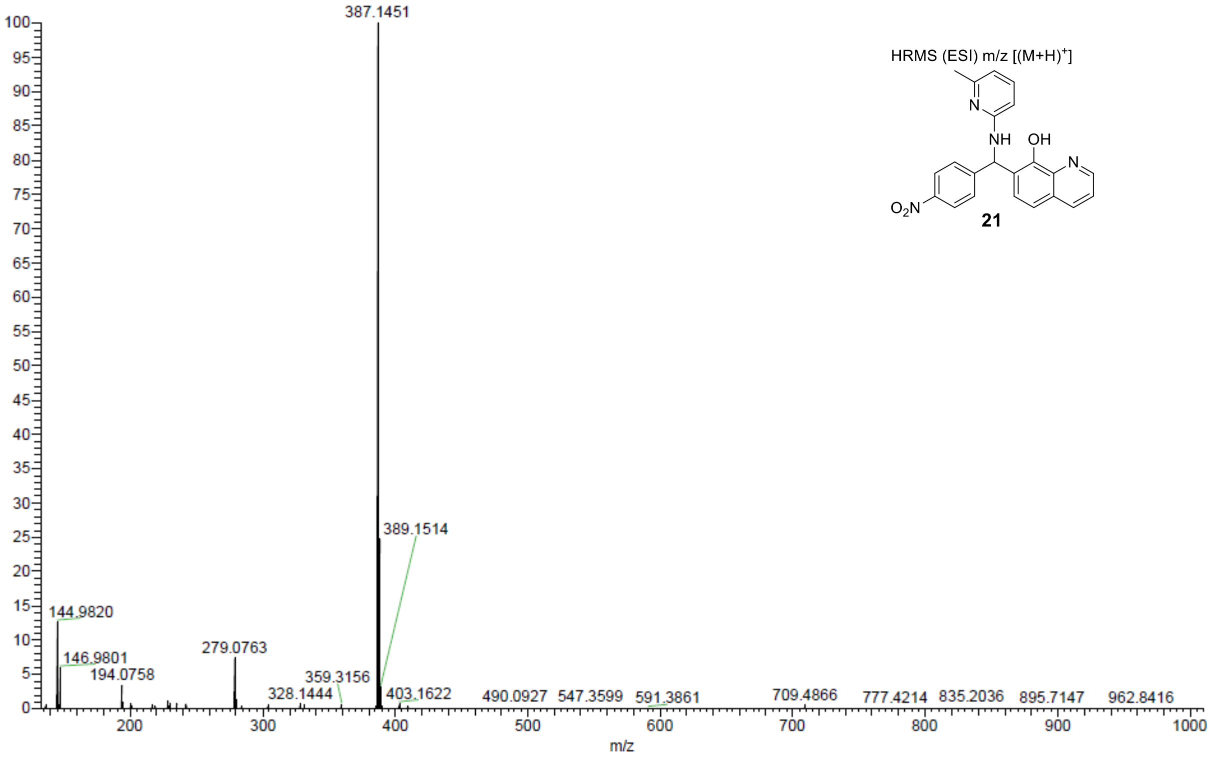
387.1451
HRMS (ESI) m/z [(M+H)+]
NH OH
N
O2N
21
389.1514
144.9820
146.9801
194.0758
279.0763
359.3156
328.1444
403.1622
490.0927 547.3599 591.3861 709.4866 777.4214 835.2036 895.7147 962.8416
m/z

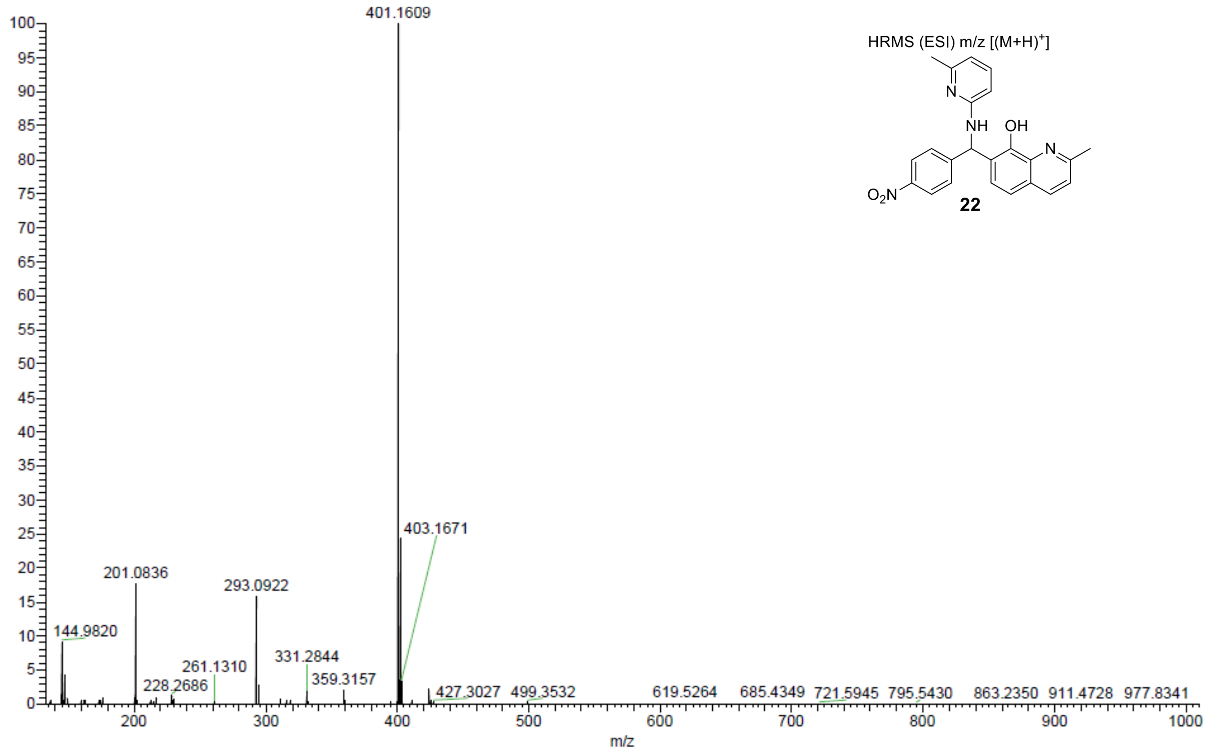
401.1609
HRMS (ESI) m/z [(M+H)+]
NH OH
N
O2N
22
403.1671
201.0836
293.0922
144.9820
331.2844
261.1310
228.2686
359.3157
427.3027 499.3532 619.5264 685.4349 721.5945 795.5430 863.2350 911.4728 977.8341
m/z

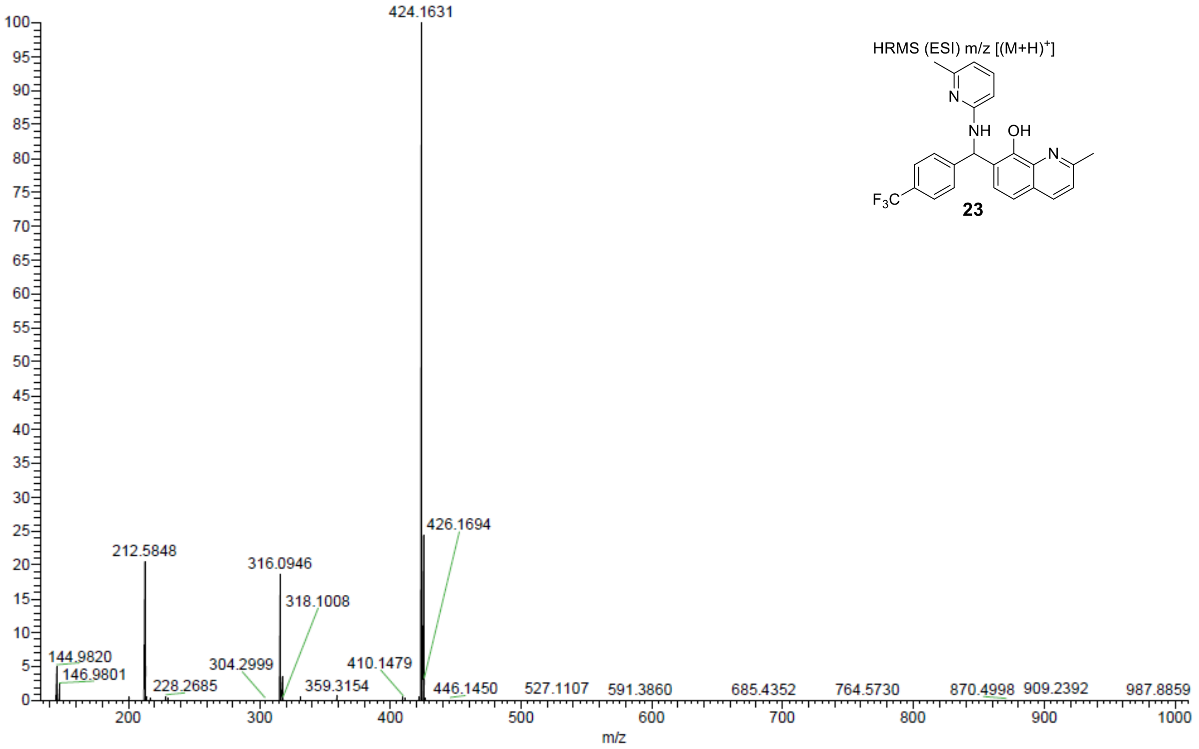
424.1631
HRMS (ESI) m/z [(M+H)+]
NH
OH
N
N
F3C
23
426.1694
212.5848
316.0946
318.1008
144.9820
146.9801
228.2685
304.2999
359.3154
410.1479
446.1450
527.1107
591.3860
685.4352
764.5730
870.4998
909.2392
987.8859
m/z

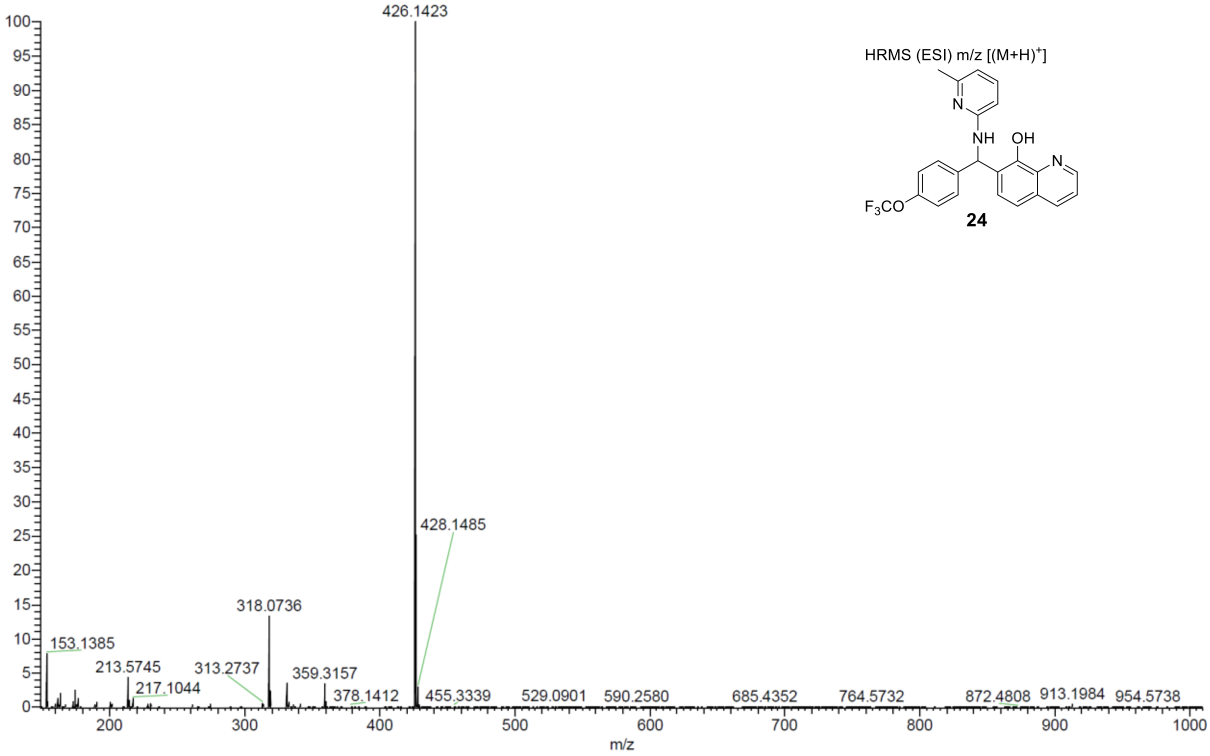
426.1423
HRMS (ESI) m/z [(M+H)+]
NH
OH
N
N
F3CO
24
428.1485
318.0736
153.1385
213.5745
217.1044
313.2737
359.3157
378.1412
455.3339
529.0901
590.2580
685.4352
764.5732
872.4808
913.1984
954.5738
m/z

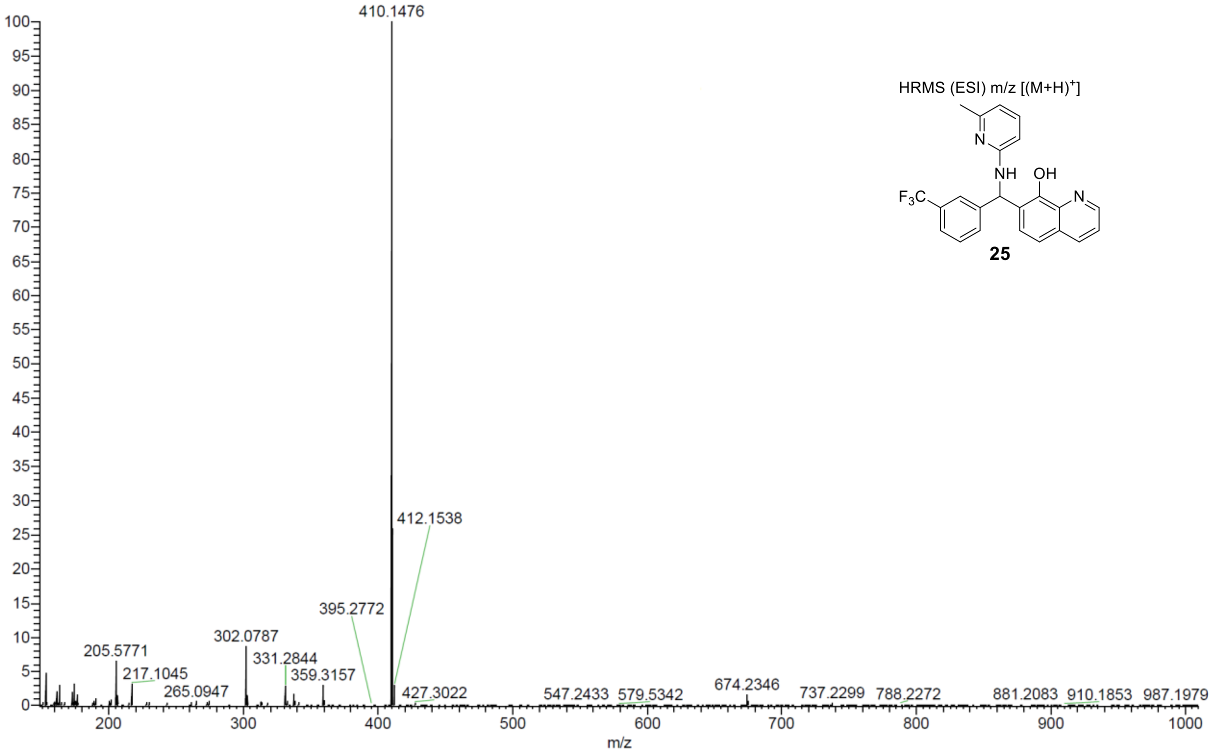
410.1476
HRMS (ESI) m/z [(M+H)+]
NH
OH
N
F3C
25
412.1538
395.2772
302.0787
205.5771
331.2844
217.1045
359.3157
265.0947
427.3022
547.2433
579.5342
674.2346
737.2299
788.2272
881.2083
910.1853
987.1979
m/z

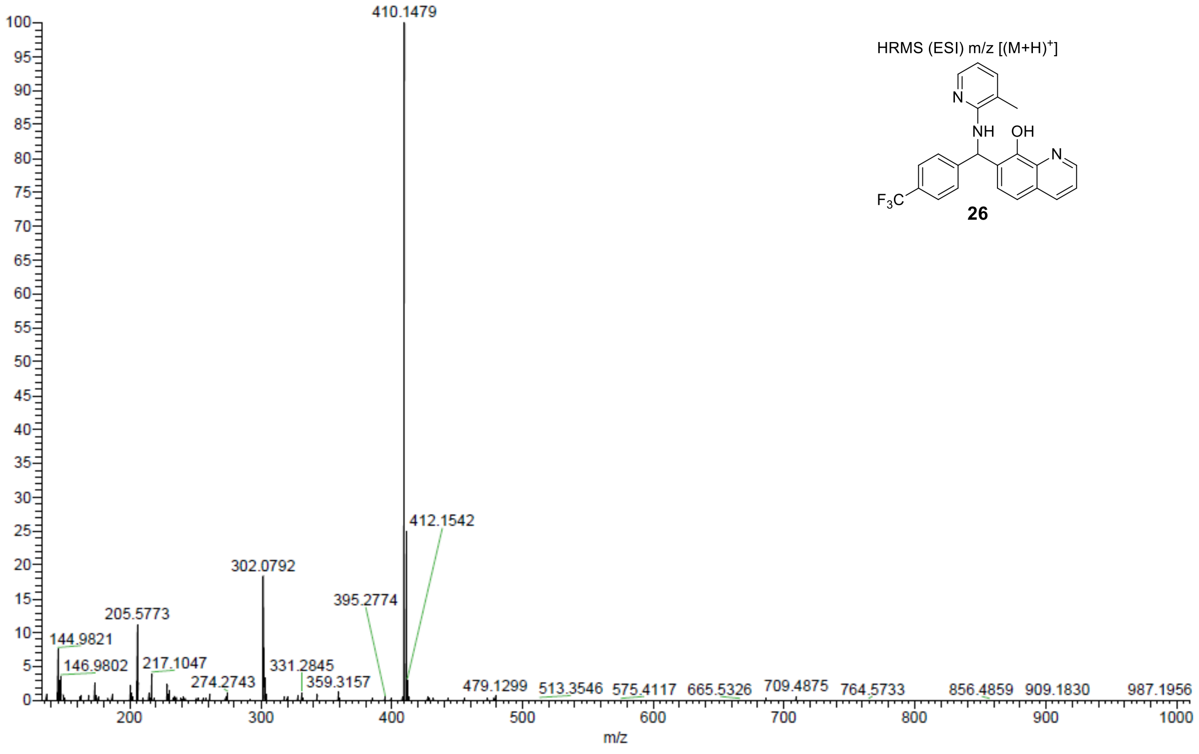
410.1479
HRMS (ESI) m/z [(M+H)+]
N
NH
OH
F3C
26
412.1542
302.0792
395.2774
205.5773
144.9821
146.9802
217.1047
331.2845
274.2743
359.3157
479.1299
513.3546
575.4117
665.5326
709.4875
764.5733
856.4859
909.1830
987.1956
m/z

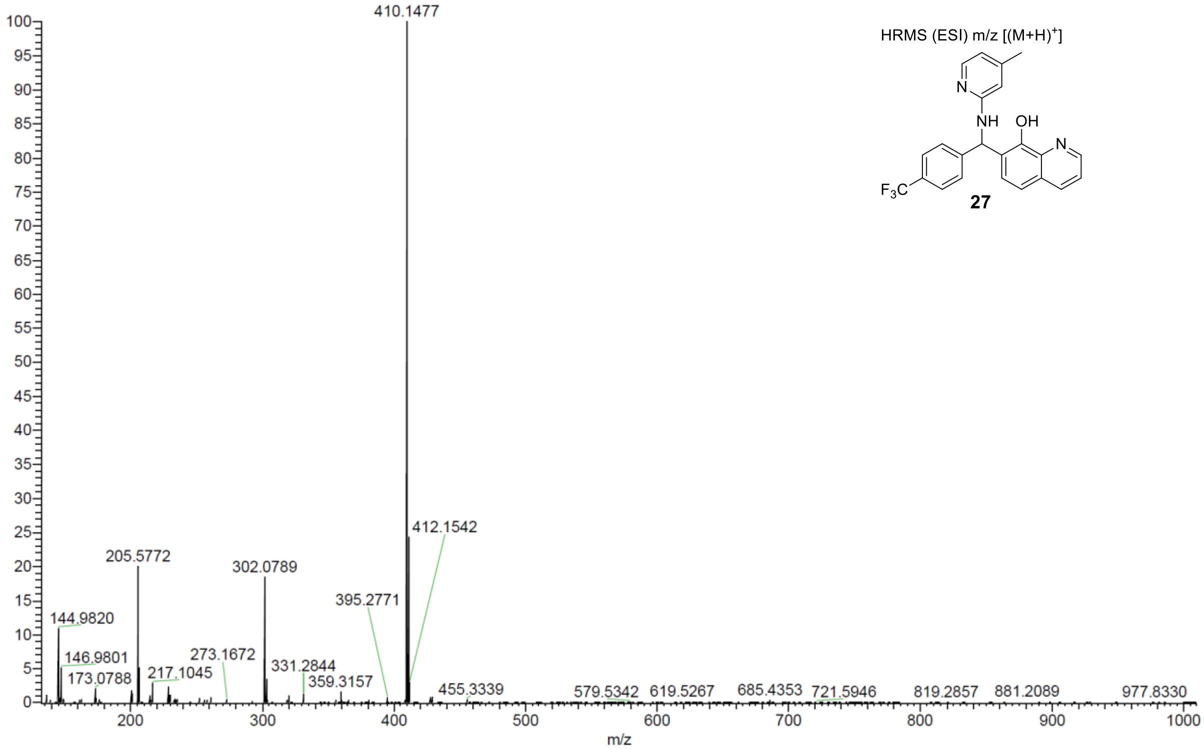
HRMS (ESI) m/z [(M+H)+]
27
410.1477
412.1542
205.5772
302.0789
395.2771
144.9820
146.9801
173.0788
217.1045
273.1672
331.2844
359.3157
455.3339
579.5342
619.5267
685.4353
721.5946
819.2857
881.2089
977.8330
m/z

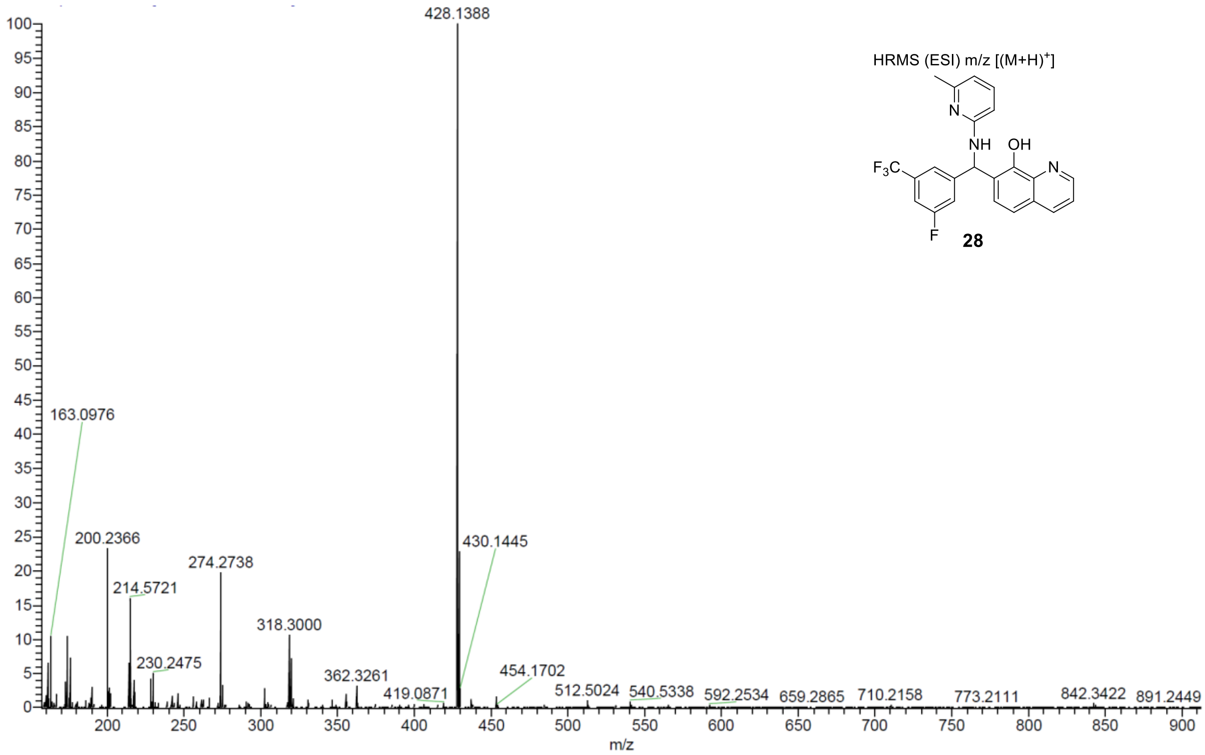
HRMS (ESI) m/z [(M+H)+]
28
428.1388
163.0976
200.2366
214.5721
274.2738
430.1445
318.3000
230.2475
362.3261
419.0871
454.1702
512.5024
540.5338
592.2534
659.2865
710.2158
773.2111
842.3422
891.2449
m/z

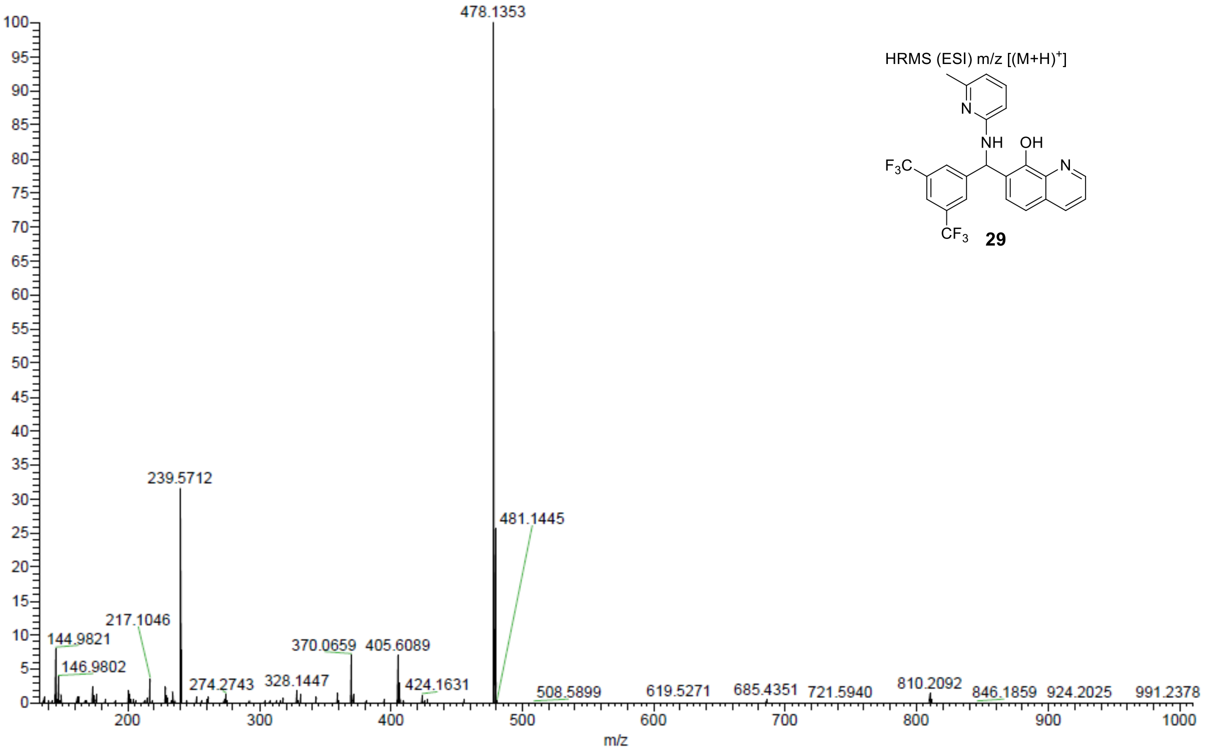
478.1353
HRMS (ESI) m/z [(M+H)+]
NH
OH
N
F3C
CF3
29
239.5712
481.1445
217.1046
144.9821
146.9802
370.0659
405.6089
274.2743
328.1447
424.1631
508.5899
619.5271
685.4351
721.5940
810.2092
846.1859
924.2025
991.2378
m/z

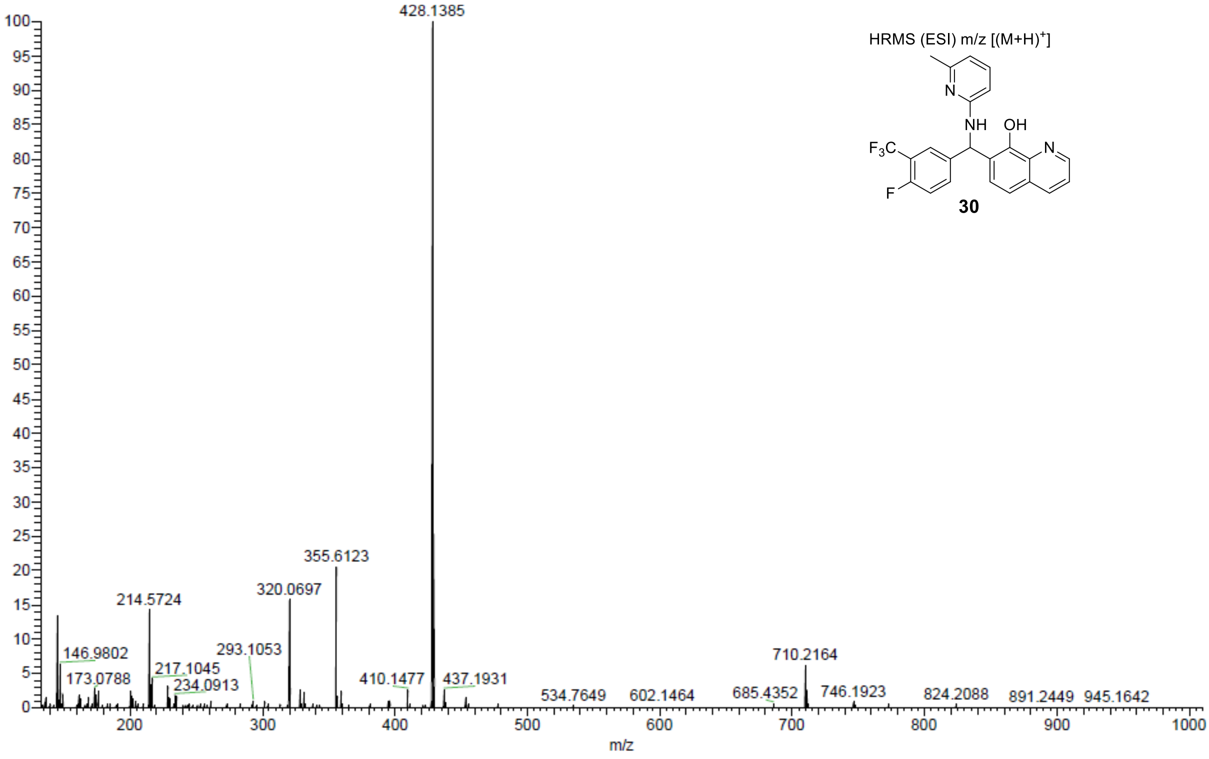
428.1385
HRMS (ESI) m/z [(M+H)+]
NH
OH
N
F3C
F
30
355.6123
214.5724
320.0697
146.9802
293.1053
173.0788
217.1045
234.0913
410.1477
437.1931
534.7649
602.1464
685.4352
710.2164
746.1923
824.2088
891.2449
945.1642
m/z

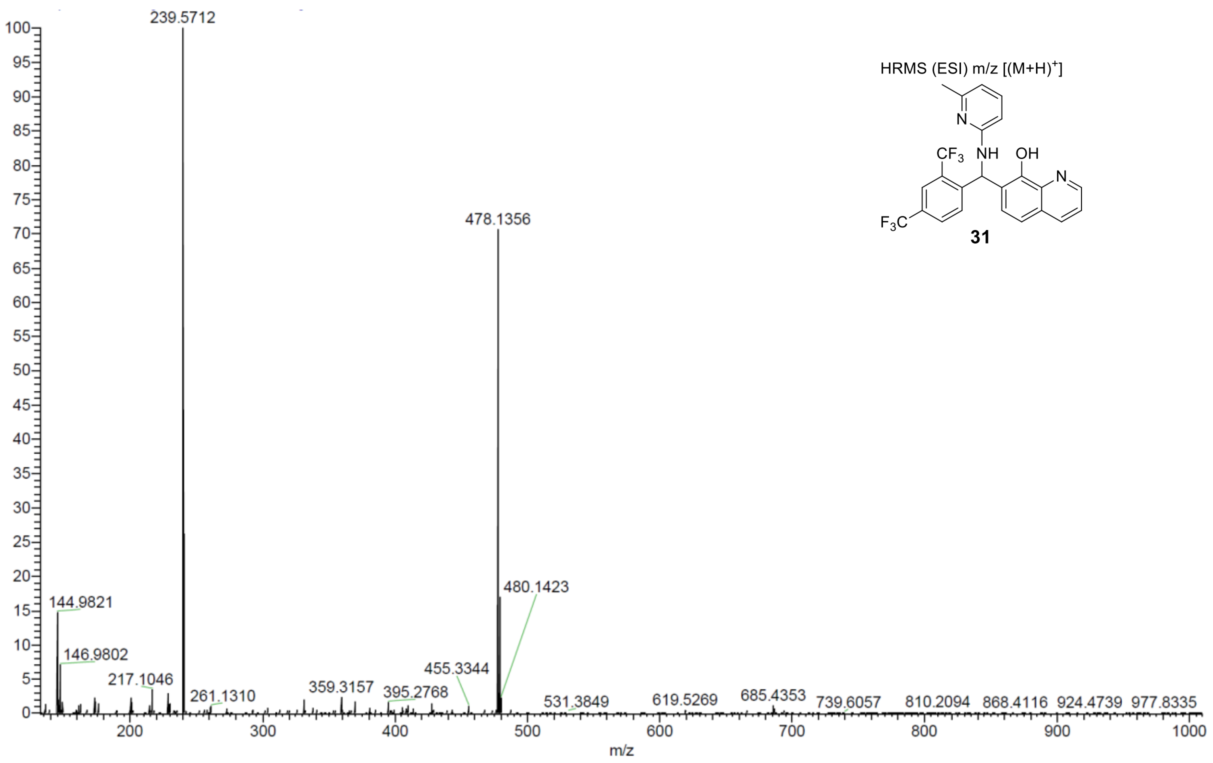
HRMS (ESI) m/z [(M+H)+]
31
239.5712
478.1356
480.1423
144.9821
146.9802
217.1046
261.1310
359.3157
395.2768
455.3344
531.3849
619.5269
685.4353
739.6057
810.2094
868.4116
924.4739
977.8335
m/z

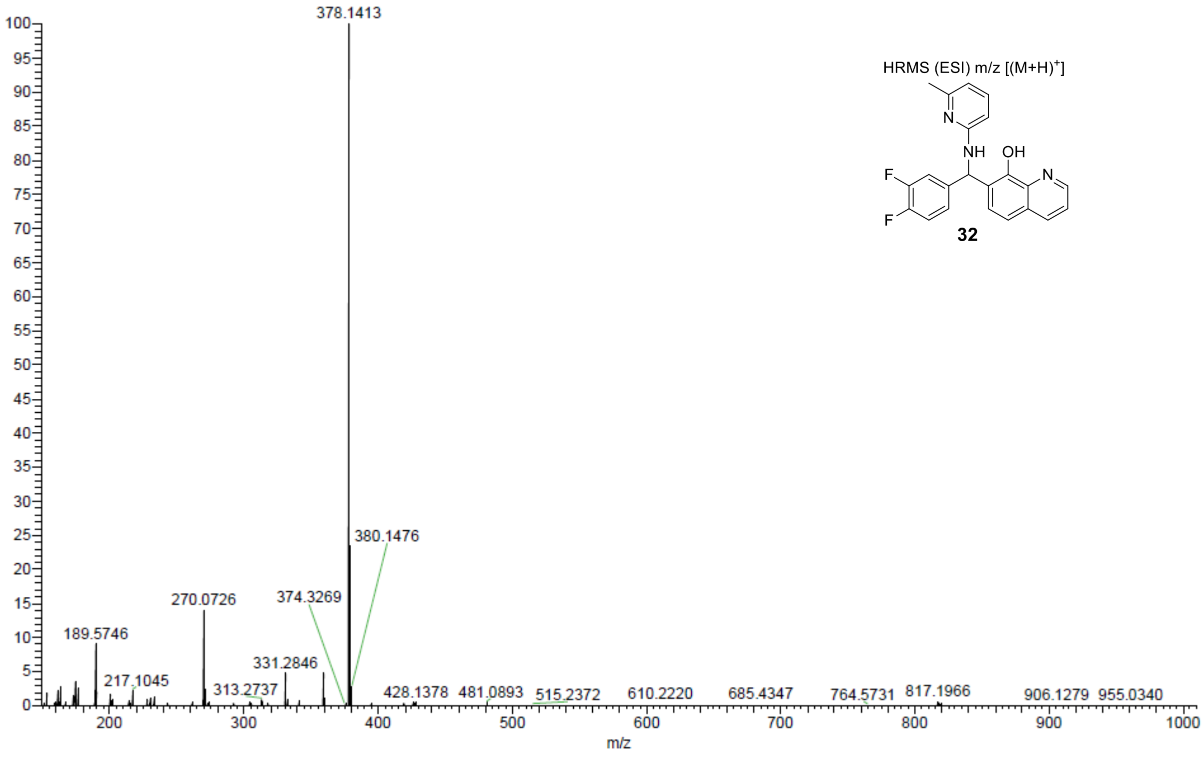
HRMS (ESI) m/z [(M+H)+]
32
378.1413
380.1476
374.3269
270.0726
189.5746
331.2846
217.1045
313.2737
428.1378
481.0893
515.2372
610.2220
685.4347
764.5731
817.1966
906.1279
955.0340
m/z

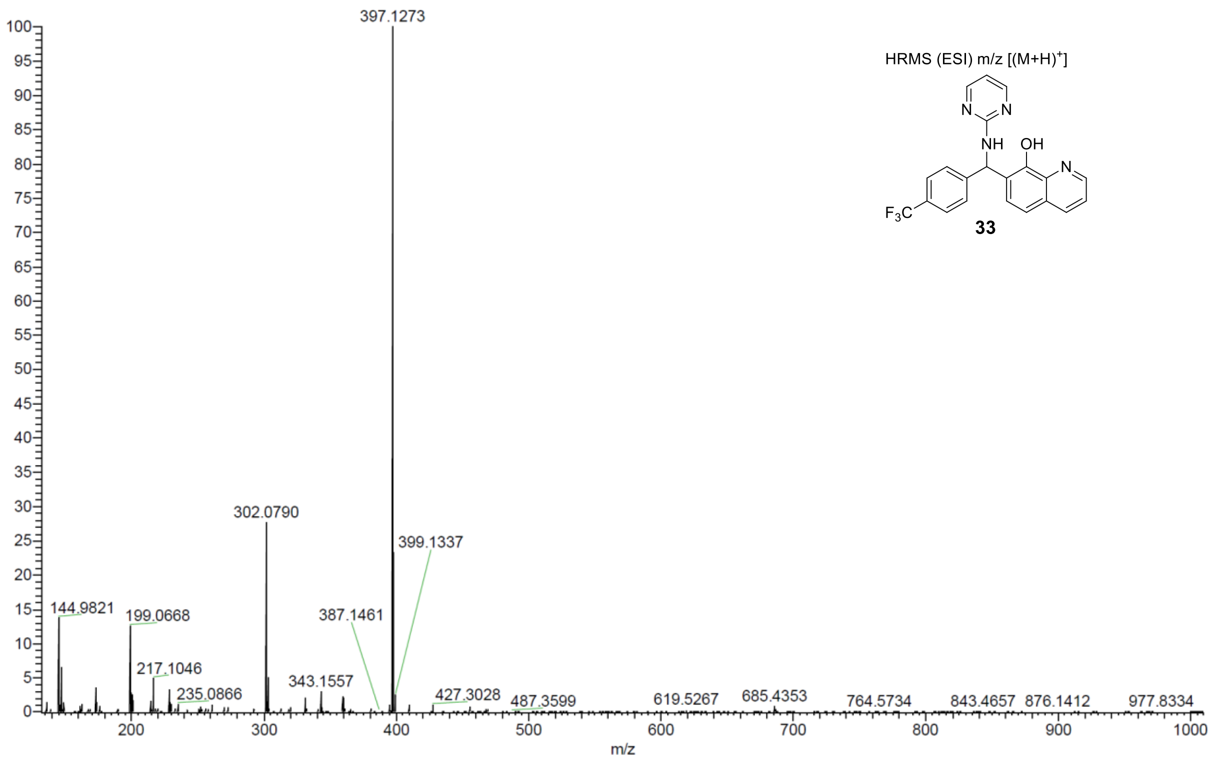

397.1273
HRMS (ESI) m/z [(M+H)+]
33
302.0790
399.1337
387.1461
144.9821
199.0668
217.1046
235.0866
343.1557
427.3028
487.3599
619.5267
685.4353
764.5734
843.4657
876.1412
977.8334
m/z

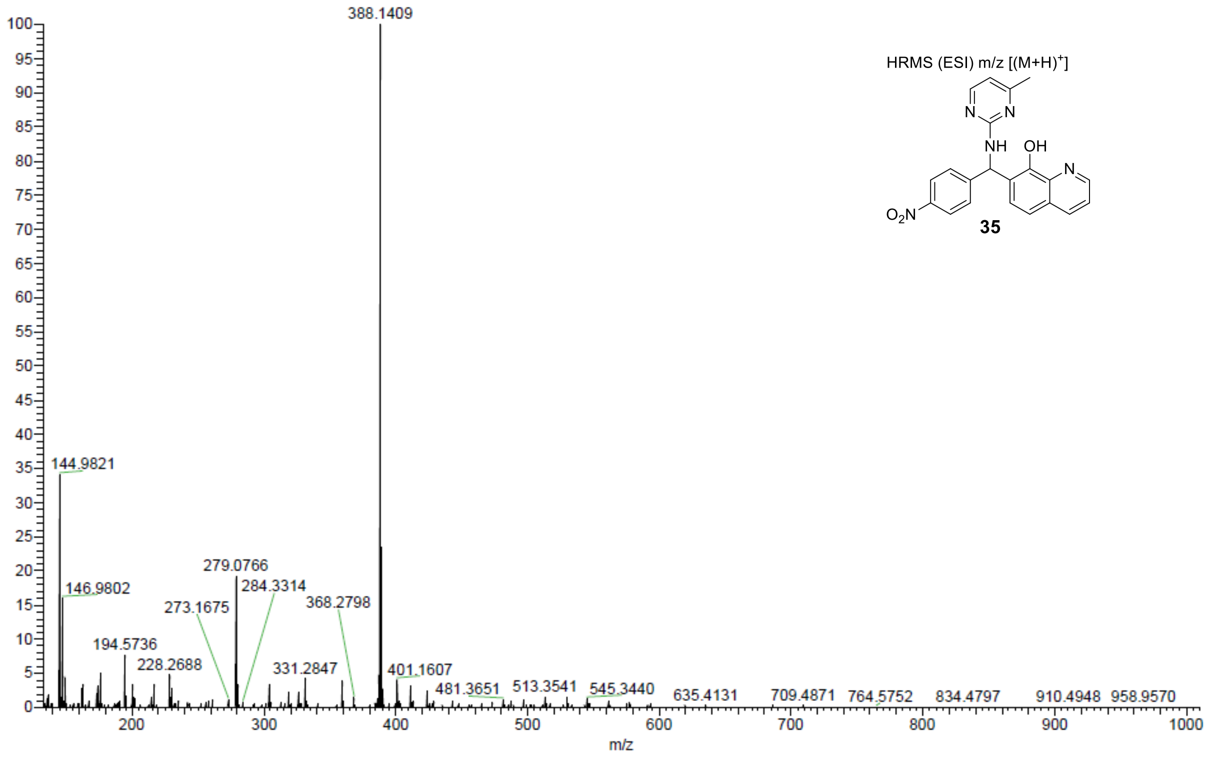

388.1409
HRMS (ESI) m/z [(M+H)+]
35
144.9821
146.9802
279.0766
284.3314
273.1675
368.2798
194.5736
228.2688
331.2847
401.1607
481.3651
513.3541
545.3440
635.4131
709.4871
764.5752
834.4797
910.4948
958.9570
m/z

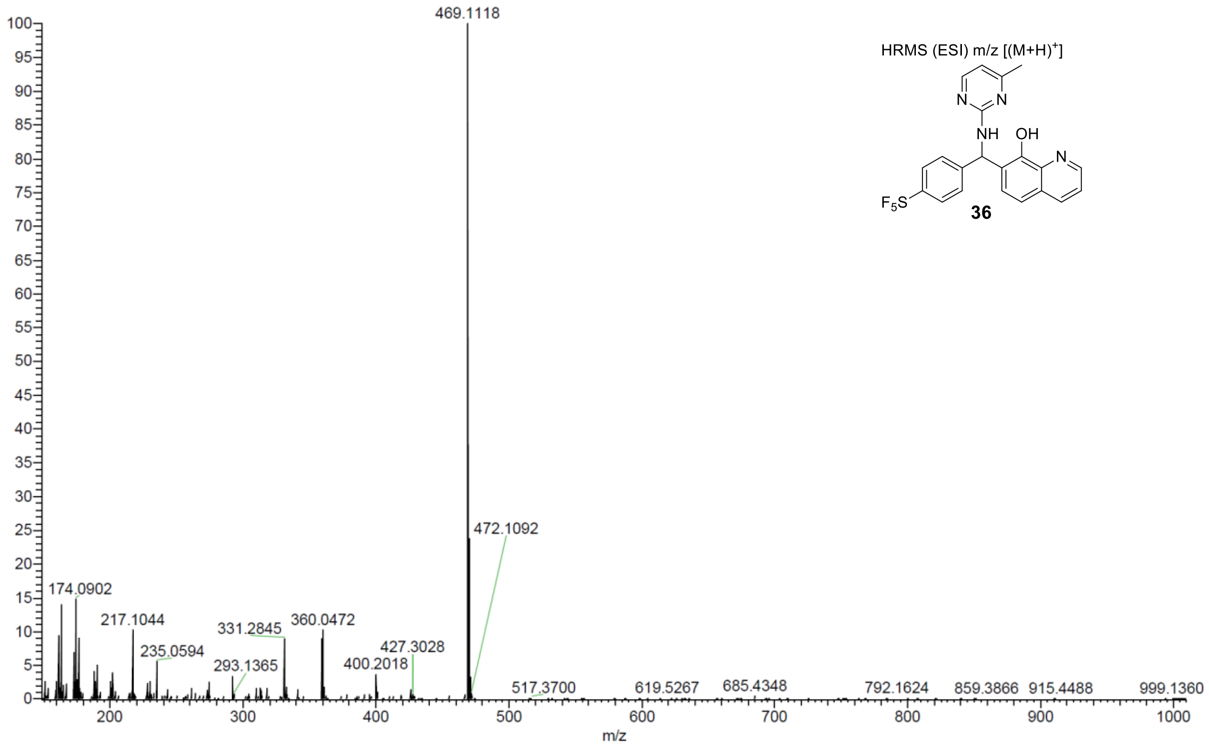
469.1118
HRMS (ESI) m/z [(M+H)+]
36
472.1092
174.0902
217.1044
235.0594
293.1365
331.2845
360.0472
400.2018
427.3028
517.3700
619.5267
685.4348
792.1624
859.3866
915.4488
999.1360
m/z

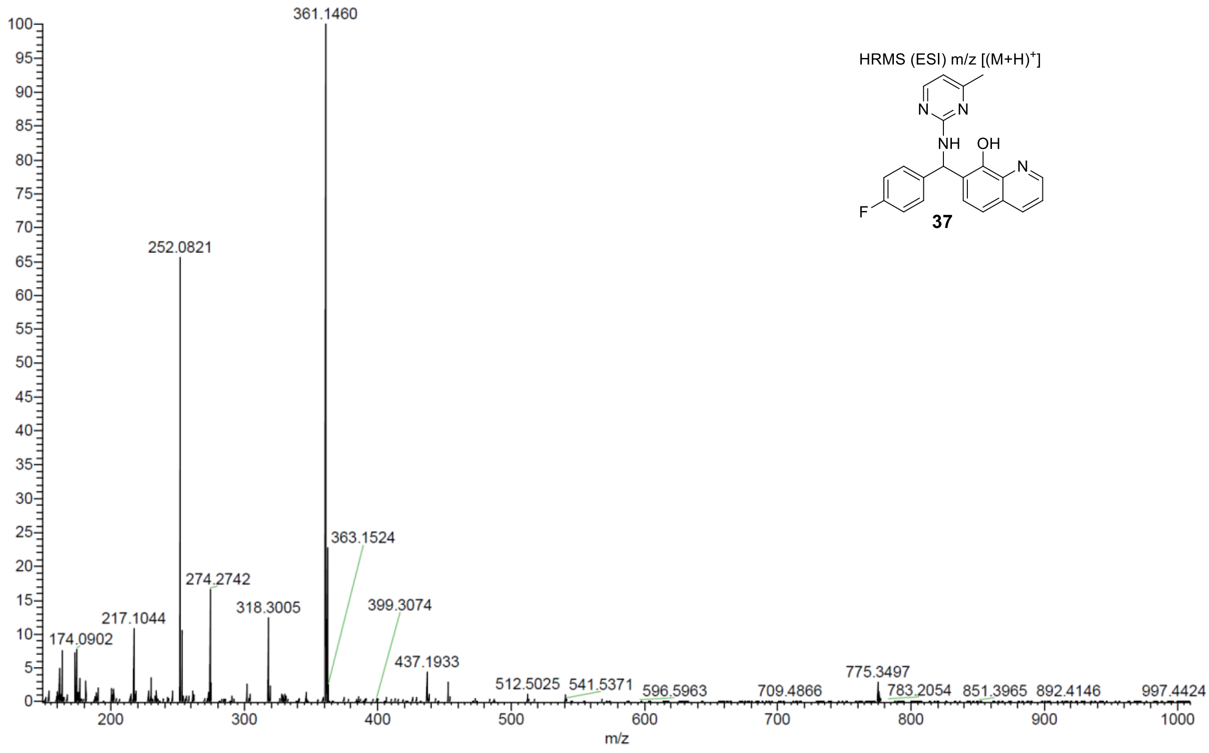
361.1460
HRMS (ESI) m/z [(M+H)+]
37
252.0821
363.1524
274.2742
217.1044
174.0902
318.3005
399.3074
437.1933
512.5025
541.5371
596.5963
709.4866
775.3497
783.2054
851.3965
892.4146
997.4424
m/z

S65

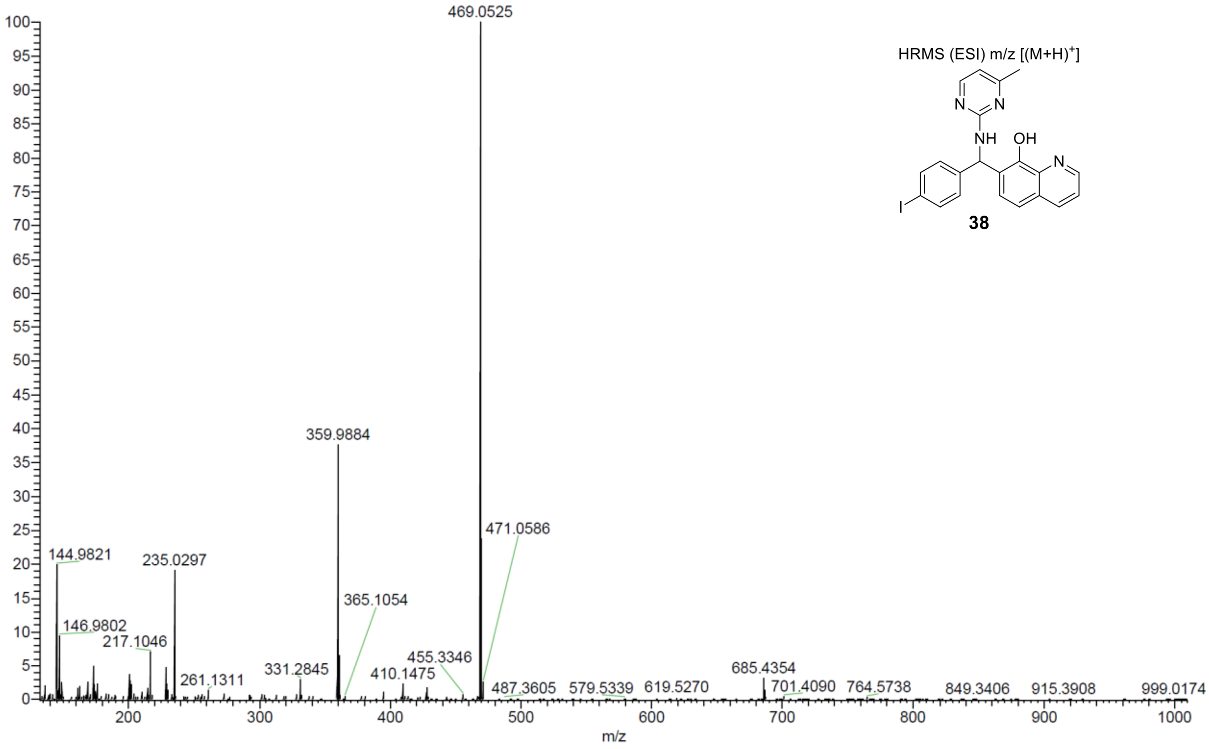

HRMS (ESI) m/z [(M+H)+]
38
469.0525
359.9884
471.0586
365.1054
144.9821
235.0297
146.9802
217.1046
261.1311
331.2845
410.1475
455.3346
487.3605
579.5339
619.5270
685.4354
701.4090
764.5738
849.3406
915.3908
999.0174
m/z

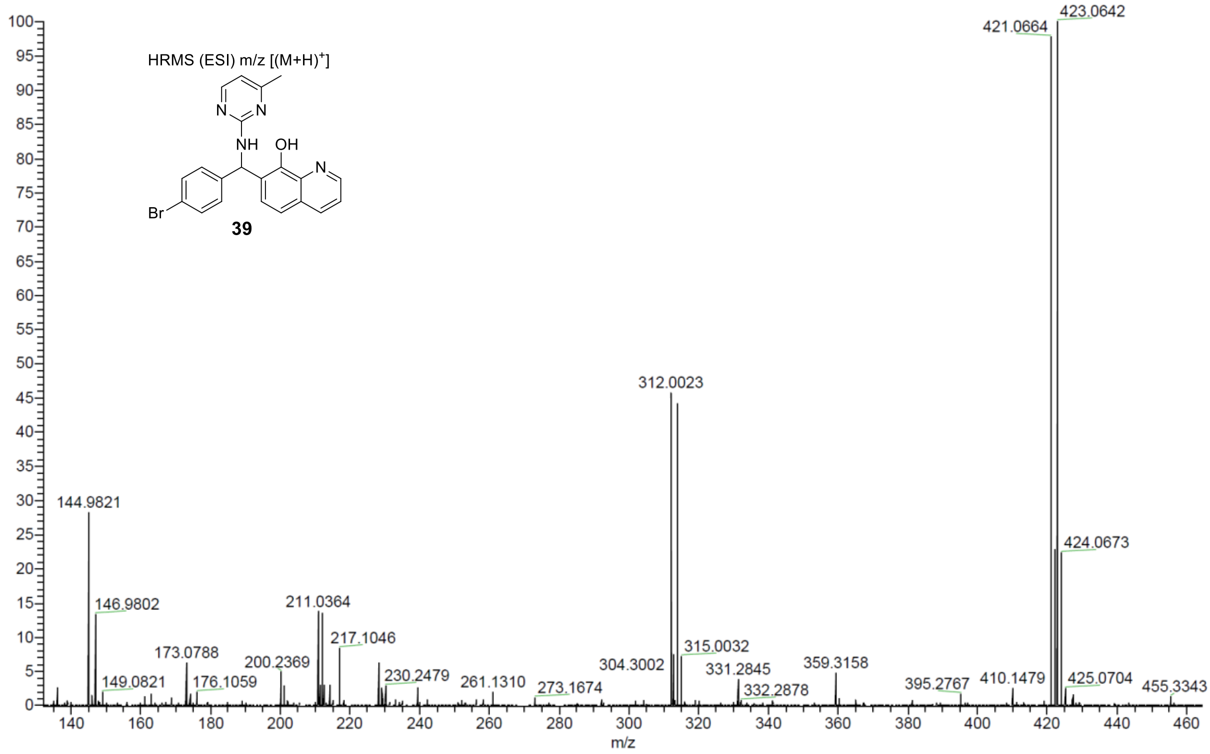

HRMS (ESI) m/z [(M+H)+]
39
421.0664
423.0642
312.0023
144.9821
424.0673
146.9802
211.0364
217.1046
173.0788
200.2369
149.0821
176.1059
230.2479
261.1310
273.1674
304.3002
315.0032
331.2845
332.2878
359.3158
395.2767
410.1479
425.0704
455.3343
m/z

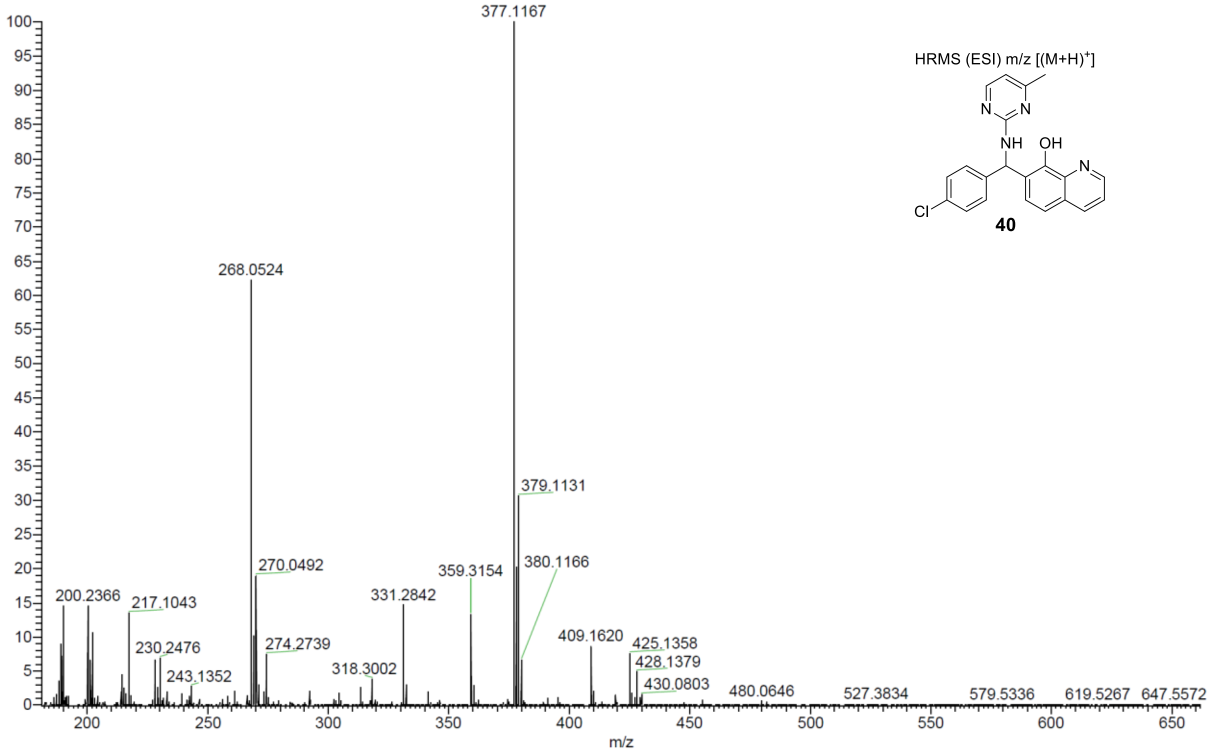
377.1167
HRMS (ESI) m/z [(M+H)+]
268.0524
379.1131
270.0492
359.3154
380.1166
331.2842
200.2366
217.1043
409.1620
274.2739
425.1358
230.2476
428.1379
318.3002
243.1352
430.0803
480.0646
527.3834
579.5336
619.5267
647.5572
m/z
40

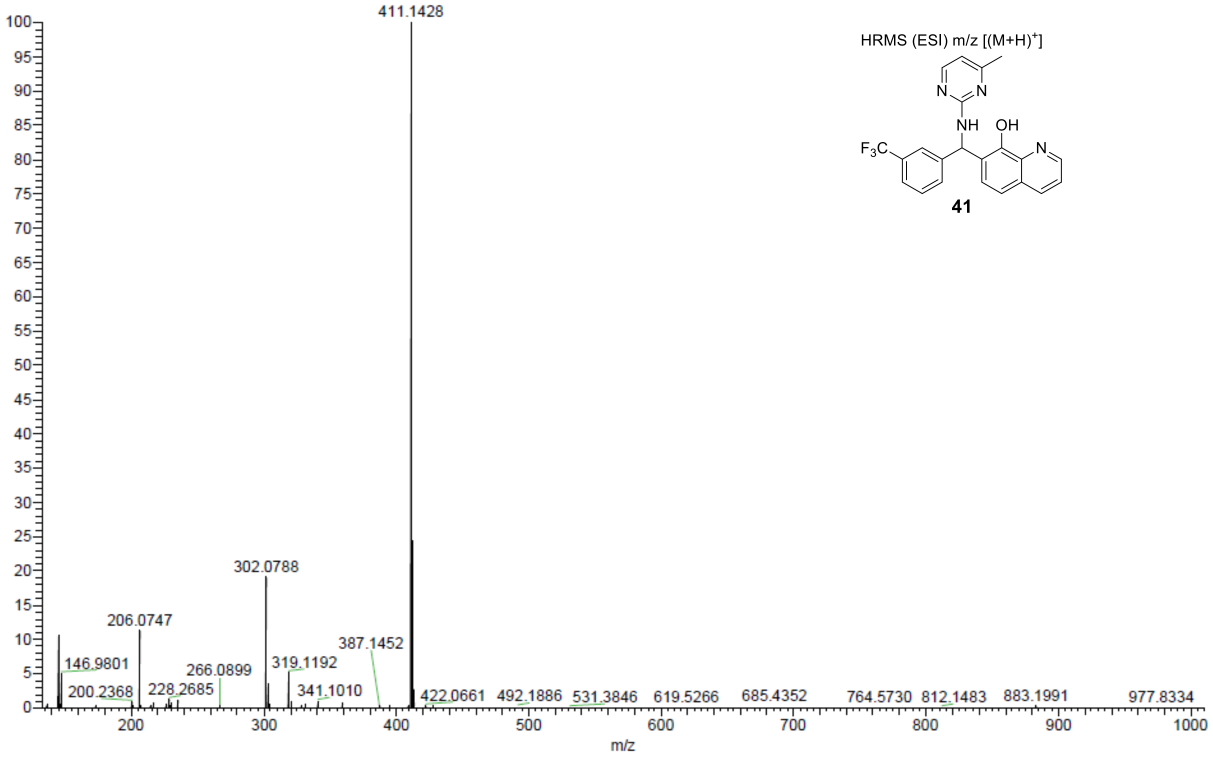
411.1428
HRMS (ESI) m/z [(M+H)+]
302.0788
206.0747
387.1452
146.9801
319.1192
266.0899
341.1010
200.2368
228.2685
422.0661
492.1886
531.3846
619.5266
685.4352
764.5730
812.1483
883.1991
977.8334
m/z
41

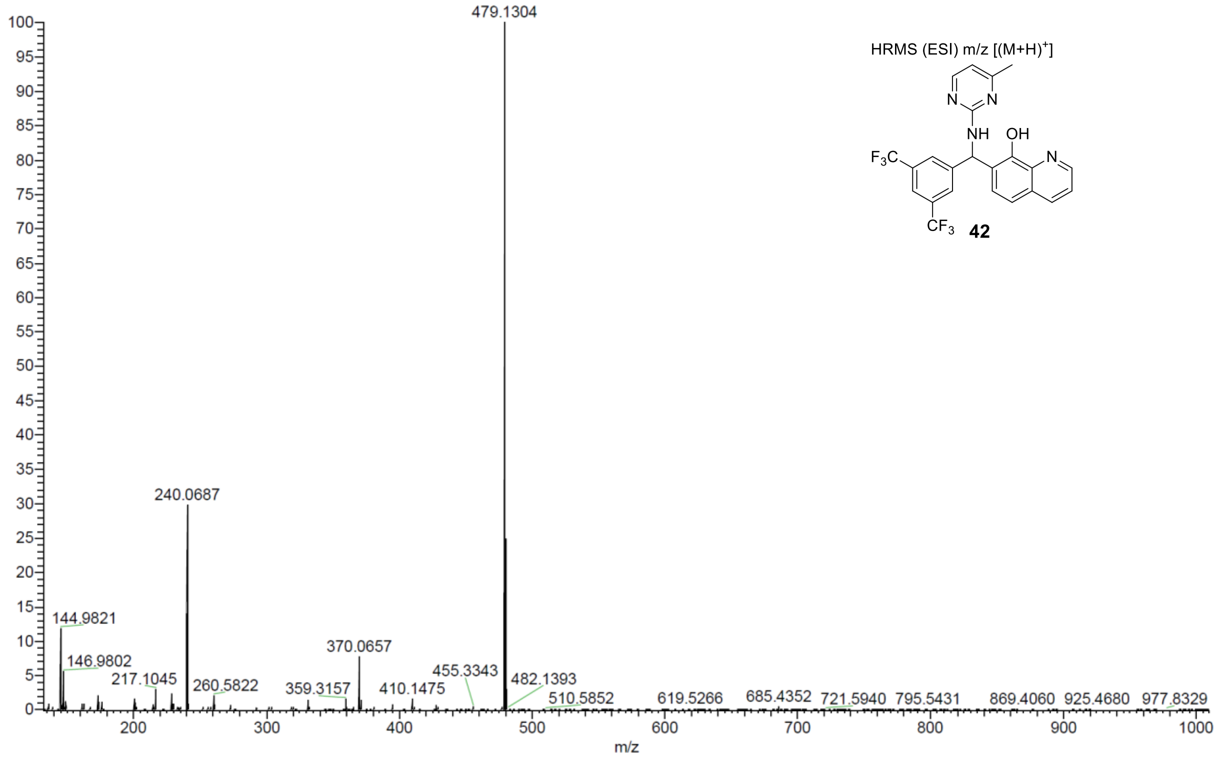

479.1304
HRMS (ESI) m/z [(M+H)+]
42
240.0687
144.9821
146.9802
217.1045
260.5822
359.3157
370.0657
410.1475
455.3343
482.1393
510.5852
619.5266
685.4352
721.5940
795.5431
869.4060
925.4680
977.8329
m/z

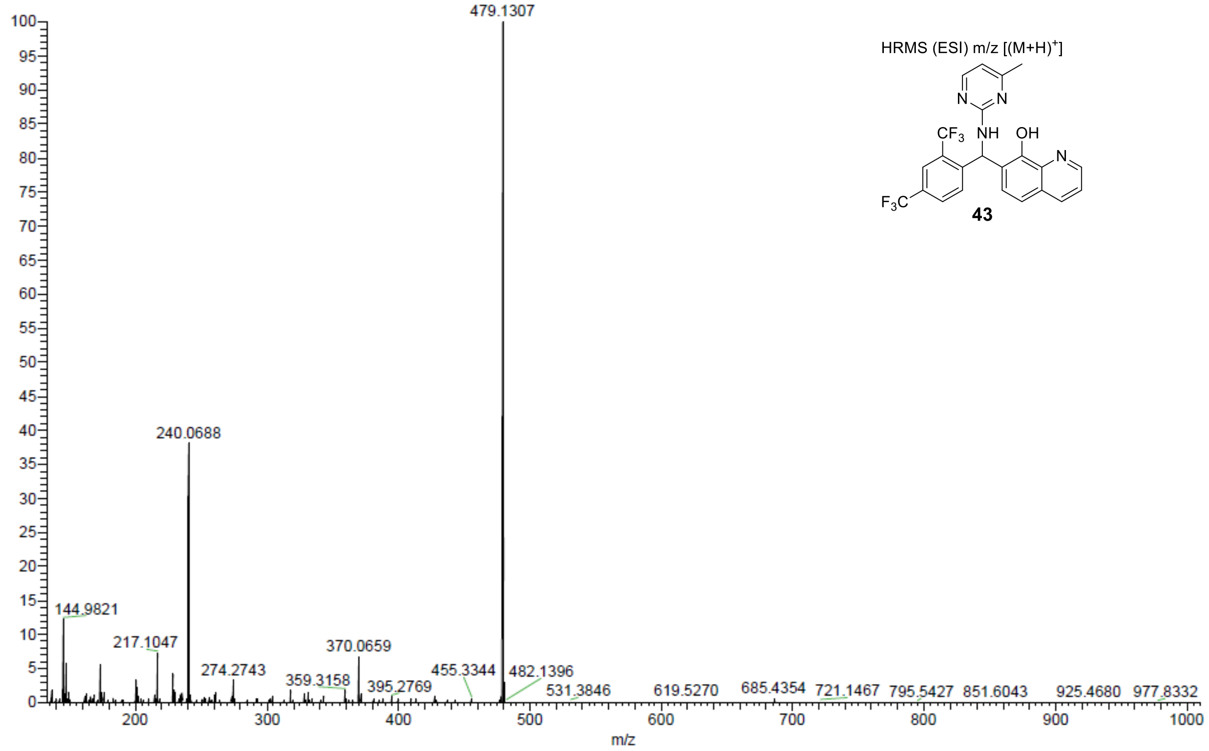

479.1307
HRMS (ESI) m/z [(M+H)+]
43
240.0688
144.9821
217.1047
274.2743
359.3158
370.0659
395.2769
455.3344
482.1396
531.3846
619.5270
685.4354
721.1467
795.5427
851.6043
925.4680
977.8332
m/z

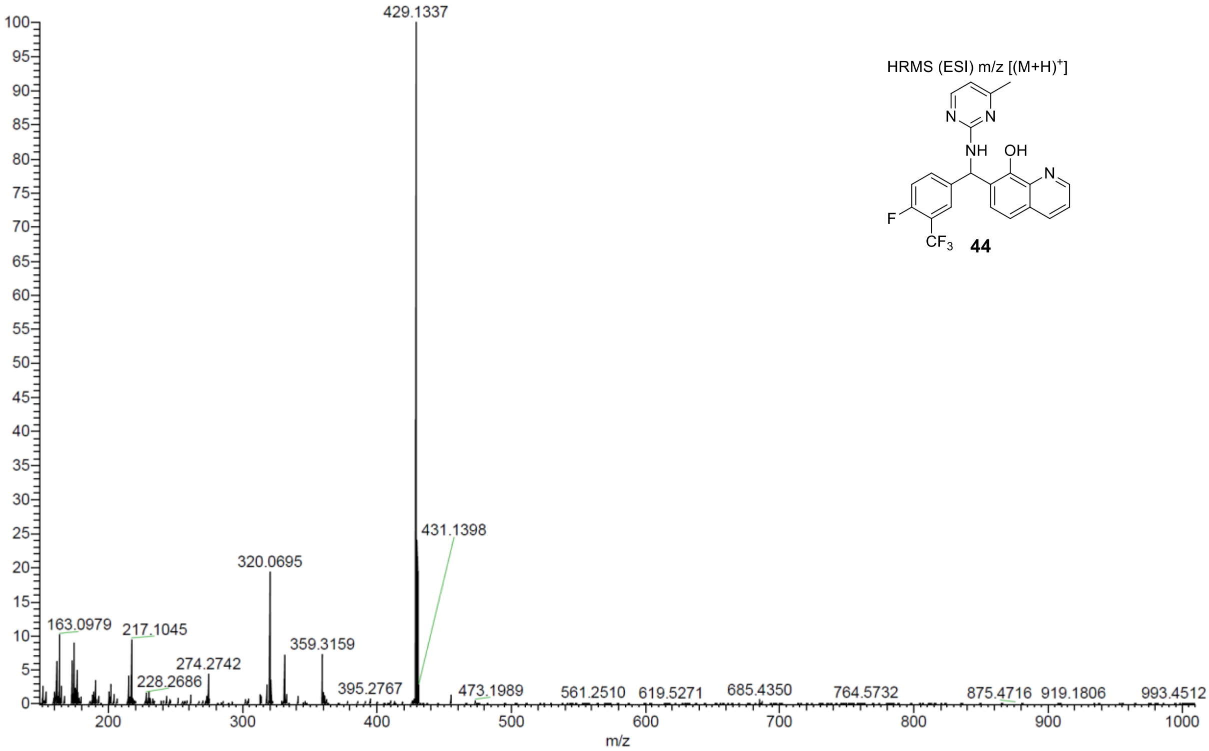
HRMS (ESI) m/z [(M+H)+]
44
429.1337
431.1398
320.0695
163.0979
217.1045
359.3159
274.2742
228.2686
395.2767
473.1989
561.2510
619.5271
685.4350
764.5732
875.4716
919.1806
993.4512
m/z

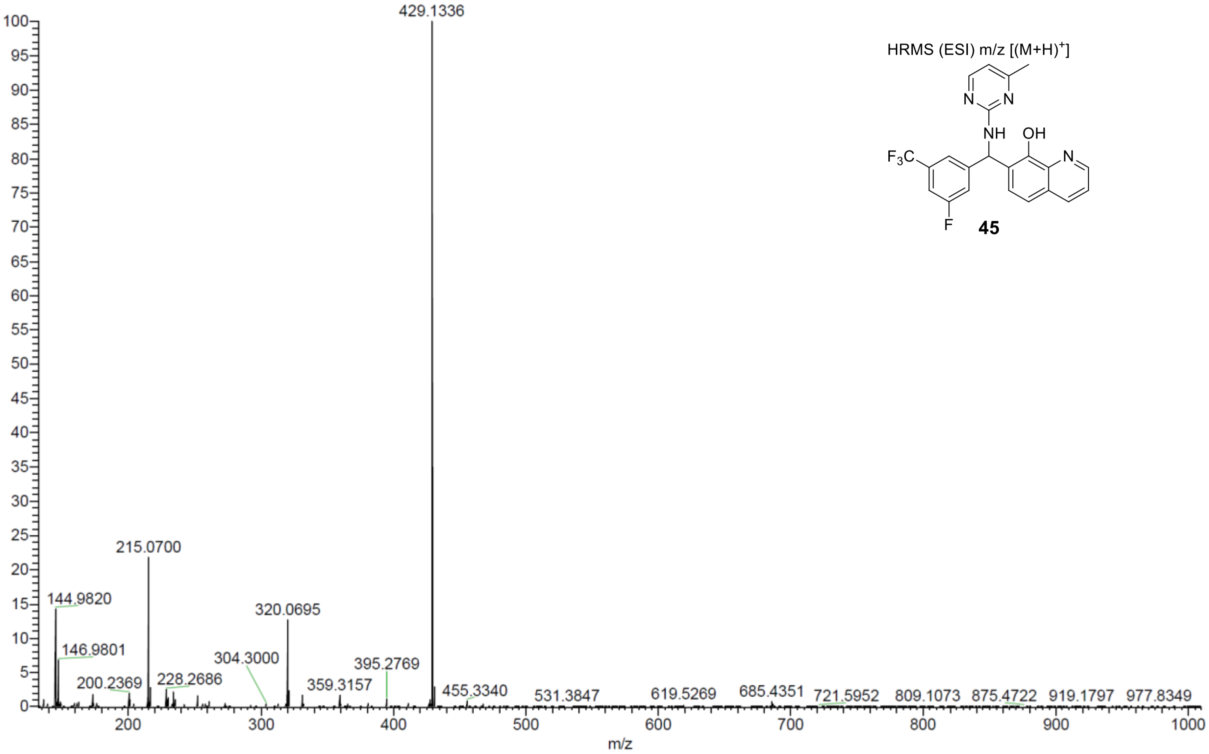
HRMS (ESI) m/z [(M+H)+]
45
429.1336
215.0700
144.9820
320.0695
146.9801
304.3000
395.2769
200.2369
228.2686
359.3157
455.3340
531.3847
619.5269
685.4351
721.5952
809.1073
875.4722
919.1797
977.8349
m/z

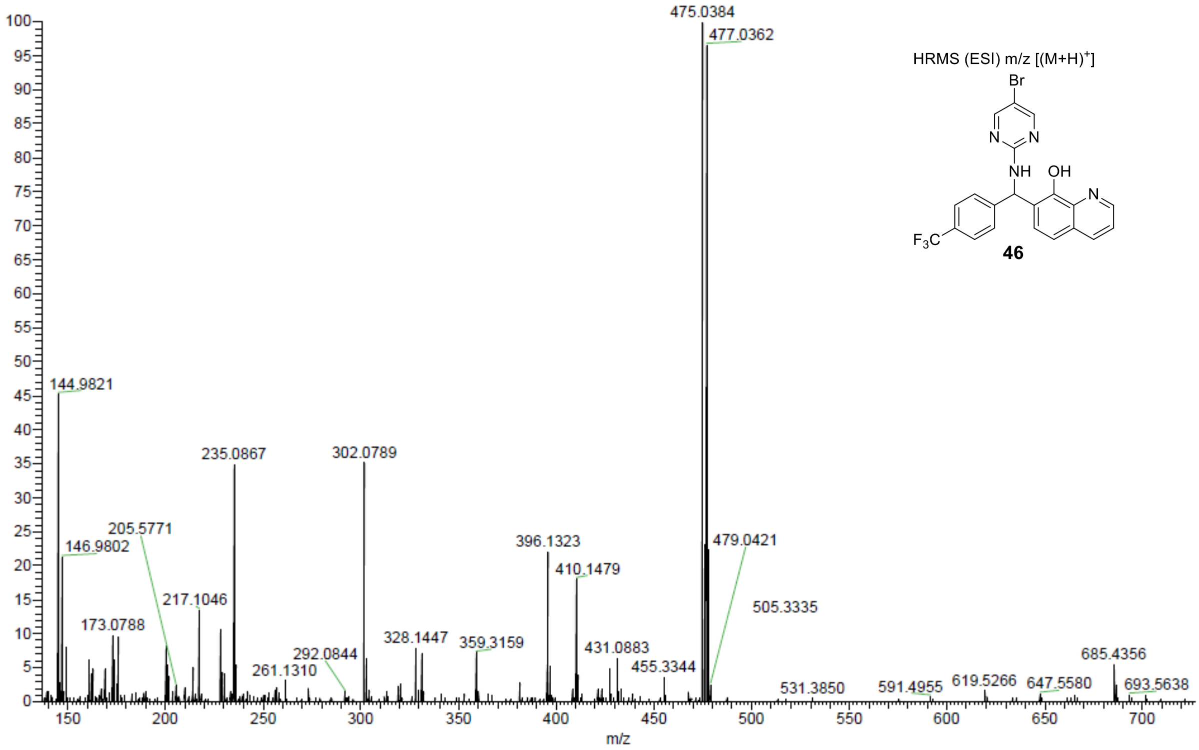
HRMS (ESI) m/z [(M+H)+]
Br
N
N
NH
OH
N
F3C
46
475.0384
477.0362
144.9821
146.9802
205.5771
173.0788
217.1046
235.0867
261.1310
292.0844
302.0789
328.1447
359.3159
396.1323
410.1479
431.0883
455.3344
479.0421
505.3335
531.3850
591.4955
619.5266
647.5580
685.4356
693.5638
m/z

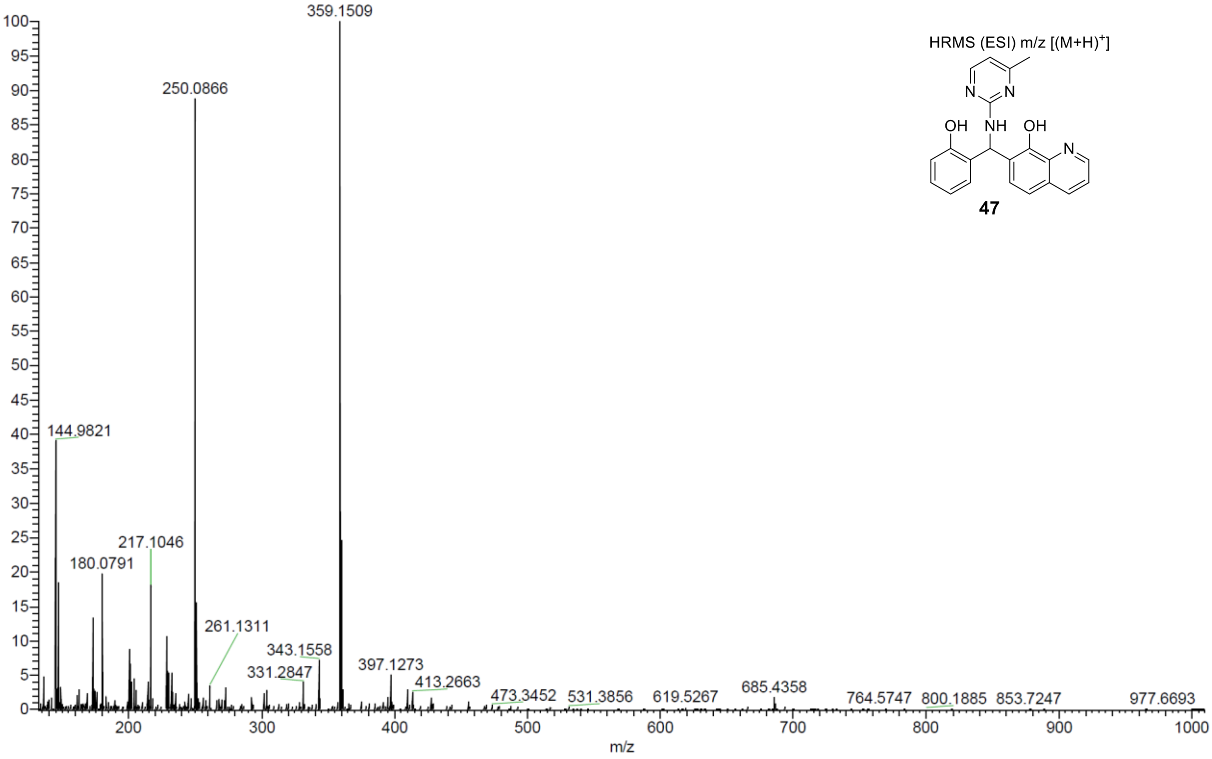
HRMS (ESI) m/z [(M+H)+]
N
N
OH
NH
OH
N
47
359.1509
250.0866
144.9821
180.0791
217.1046
261.1311
331.2847
343.1558
397.1273
413.2663
473.3452
531.3856
619.5267
685.4358
764.5747
800.1885
853.7247
977.6693
m/z

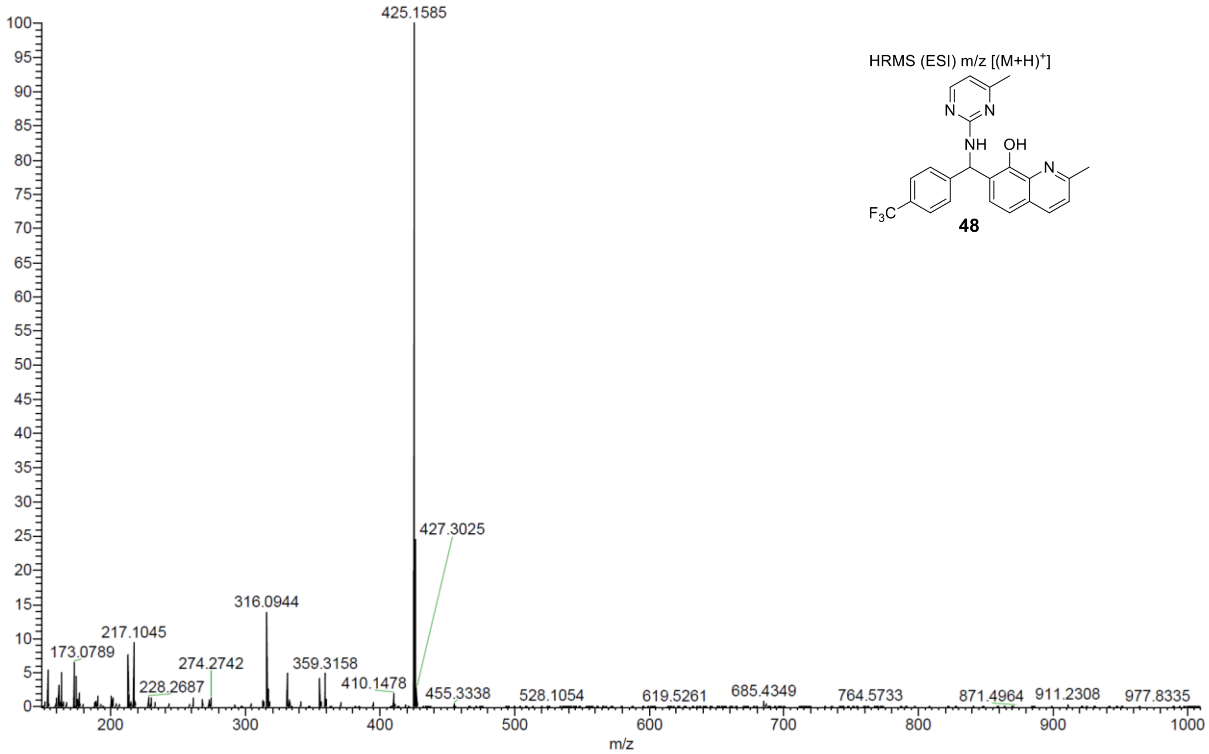
HRMS (ESI) m/z [(M+H)+]
48
425.1585
427.3025
316.0944
217.1045
173.0789
274.2742
359.3158
228.2687
410.1478
455.3338
528.1054
619.5261
685.4349
764.5733
871.4964
911.2308
977.8335
m/z
